# Comparison of Parallel High-Throughput RNA Sequencing Between Knockout of TDP-43 and Its Overexpression Reveals Primarily Nonreciprocal and Nonoverlapping Gene Expression Changes in the Central Nervous System of Drosophila

**DOI:** 10.1534/g3.112.002998

**Published:** 2012-07-01

**Authors:** Dennis J. Hazelett, Jer-Cherng Chang, Daniel L. Lakeland, David B. Morton

**Affiliations:** Department of Integrative Biosciences, Oregon Health & Science University, Portland, Oregon 97239

**Keywords:** TARDBP, neurodegeneration, neuropathy, invertebrate models of human disease, RNA binding protein, genomics

## Abstract

The human Tar-DNA binding protein, TDP-43, is associated with amyotrophic lateral sclerosis (ALS) and other neurodegenerative disorders. TDP-43 contains two conserved RNA-binding motifs and has documented roles in RNA metabolism, including pre-mRNA splicing and repression of transcription. Here, using *Drosophila melanogaster* as a model, we generated loss-of-function and overexpression genotypes of Tar-DNA binding protein homolog (TBPH) to study their effect on the transcriptome of the central nervous system (CNS). By using massively parallel sequencing methods (RNA-seq) to profile the CNS, we find that loss of TBPH results in widespread gene activation and altered splicing, much of which are reversed by rescue of TBPH expression. Conversely, TBPH overexpression results in decreased gene expression. Although previous studies implicated both absence and mis-expression of TDP-43 in ALS, our data exhibit little overlap in the gene expression between them, suggesting that the bulk of genes affected by TBPH loss-of-function and overexpression are different. In combination with computational approaches to identify likely TBPH targets and orthologs of previously identified vertebrate TDP-43 targets, we provide a comprehensive analysis of enriched gene ontologies. Our data suggest that TDP-43 plays a role in synaptic transmission, synaptic release, and endocytosis. We also uncovered a potential novel regulation of the Wnt and BMP pathways, many of whose targets appear to be conserved.

Amyotrophic lateral sclerosis (ALS) is a devastating neurological disease affecting about 2 in 100,000 of the population per year (Beghi *et al.* 2006). This progressive and irreversible disease is characterized by the asymmetric paralysis of the lower limbs and extremities. The prognosis for patients suffering from ALS is grim; 90% will die within 10 years of diagnosis (Beghi *et al.* 2006). In post-mortem samples, ALS patients have intraneuronal aggregates or inclusions composed of hyperphosphorylated, hyperubiquitinated protein (Mitsuyama 1984; Leigh *et al.* 1988; Lowe *et al.* 1988, Migheli *et al.* 1990; Leigh *et al.* 1991; Neumann *et al.* 2006). Six years ago, TDP-43 was identified as one of the primary constituents of these inclusions in ALS and a related neurodegenerative disorder, Fronto-temporal lobar dementia with ubiquitin-positive inclusions (FTLD-U) (Neumann *et al.* 2006), thus spurring a new age of research into the etiology of this devastating family of diseases.

TDP-43 was previously described as a splicing factor (Buratti *et al.* 2001) and a transcriptional repressor (Ou *et al.* 1995; Abhyankar *et al.* 2007). Since then, numerous researchers have sought to assign cellular roles to TDP-43 as a regulator of splicing (Ayala *et al.* 2006, Bose *et al.* 2008), transcription (Ayala *et al.* 2008b), and microRNA biogenesis (Buratti *et al.* 2010), and as a factor that binds and stabilizes neurofilament RNA in the cytoplasm (Strong *et al.* 2007). Consistent with its major function in RNA metabolism, TDP-43 contains two highly conserved RNA binding domains (Ou *et al.* 1995). It also has nuclear import and export motifs and is actively shuttled between the nucleus and cytoplasm in a transcription-dependent manner, consistent with these reported activities (Ayala *et al.* 2008a).

Recent studies have identified potential targets for TDP-43 (Polymenidou *et al.* 2011; Sephton *et al.* 2011; Tollervey *et al.* 2011) and provided important insights into the general role that TDP-43 fills in the nervous system. But there remain some deeply perplexing questions about the basic etiology of TDP-43-opathies. Presently, it remains unclear whether aggregation of TDP-43 into inclusions is detrimental (see Hanson *et al.* 2010), or whether cellular distress is caused by cytoplasmic expression of TDP-43 or by loss of TDP-43 from the nucleus (Lee *et al.* 2012). Overexpression of TDP-43 in a variety of contexts from yeast to mice is certainly detrimental to cells and can cause ALS-like phenotypes at the cellular and organismal level (Johnson *et al.* 2009, Wegorzewska *et al.* 2009; Li *et al.* 2010). The major model that our article is attempting to examine is the proposition that TDP-43 pathology is associated with loss-of-function in the nucleus. Many papers have now been published using overexpression of wild-type and mutant versions of TDP-43 and its various orthologs in a variety of cell-culture and animal models. Generally speaking, overexpression is reported to result in cytoplasmic mislocalization. It remains unclear, however, what the effect of such manipulations are, and what their implications for disease are. For example, does cytoplasmic mislocalization of TDP-43 in an overexpression experiment have the same consequence as loss-of-function in the nucleus? We have attempted to clarify this situation by examining the effect on the transcriptome directly. Here we describe experiments in which we have altered TDP-43 levels by means of genetic manipulations to make a direct comparison of loss-of-function with overexpression. We examined the effect on gene expression and splicing in the nervous system of *Drosophila melanogaster*. Our findings show that overexpression and loss-of-function of TDP-43 have very different consequences with respect to gene expression and splicing, both in terms of the types of genes and the way they are regulated.

## Materials and Methods

### Fly stocks

All fly strains were obtained from the Bloomington stock center (http://flystocks.bio.indiana.edu/) unless otherwise indicated. All animals and genotypes were reared at 25° using standard procedures (Greenspan 2004). The D42-GAL4 motor neuron driver was obtained from the Bloomington stock center and the TBPH loss of function and overexpression and the TPH-GAL4 driver lines were generated in this study as described below.

### Generation of TBPH null mutations

**Figure 1:**
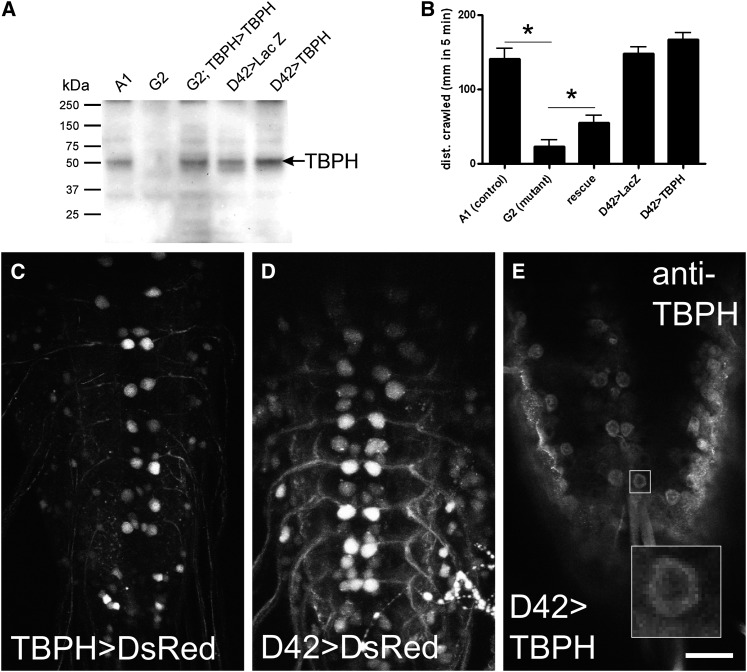
Characterization of fly stocks used in this study. (A) Western blot of larval 3^rd^ instar CNS with anti-TBPH antibody shows absence of TBPH protein in deletion mutants (G2) compared with revertant control (A1) and rescue lines (G2/G2;TBPH-GAL4 > UAS-TBPH). Increased expression of TBPH is seen in the overexpression line (D42-GAL4 > UAS-TBPH) and compared with control (D42 > LacZ). (B) Larval locomotion assay demonstrates that G2 has severely reduced crawling behavior that is partially rescued by driving TBPH with the TBPH promoter (rescue). Overexpression of TBPH in motor neurons (D42-GAL4 > UAS-TBPH) has no effect on larval locomotion. (C–E) TBPH is expressed in motor neurons: (C, D) Confocal z-stack images of larval ventral nervous system driving the fluorescent protein DsRed under control of TBPH-GAL4 and D42-GAL4, respectively, illustrate the expression pattern of the drivers used in this study. Anterior is at top, posterior bottom. (E) TBPH antibody detects overexpressed TBPH in motor neurons of D42-GAL4 > UAS-TBPH overexpressing animals. A single z-section is shown to highlight motor neurons and remove background staining on the surface of the tissue. Inset shows expression is predominantly cytoplasmic. Scale bar = 30 µm for C–E.

Insertion line KG08578 (Bloomington stock center) contains a p-element upstream of TBPH and is lethal, but it complements lethality of an overlapping deficiency, Df[2R]or-BR11. Therefore we out-crossed KG08578 with the *w*^1118^ wild-type strain to remove the lethality until we obtained a new homozygous viable strain. The p-element insertion within this strain was mobilized using standard procedures (Greenspan 2004). In this fashion 2932 chromosomes were screened, of which 161 (5%) lost the white^+^ marker. Of these 5 (0.1% of total) had lethal mutations, and 3 strains, including TBPH[PxG2] (G2), failed to complement the lethality of Df(2R)or-BR11. These three strains also failed to complement Δ142 and Δ23, two previously described alleles of TBPH (Feiguin *et al.* 2009). Additional strains, including TBPH[PxA1] (A1), were also retained from among the nonlethal white-eyed strains as potential controls. Polymerase chain reaction (PCR) primers flanking the adjacent upstream gene (CG4585) and the first large intron of TBPH were used to amplify the deletion. The resulting PCR products were then rapid-ligated into a TA-cloning vector (pCR4, Invitrogen) and sequenced. The deleted region in G2 was determined to be G2:(2R)19749305.0.19751447. There was residual transcription detectable by RT-PCR (not shown) and deep sequencing, but no protein was detected by Western blot of larval CNS tissue (Figure 1A). The predicted transcript should “read through” from the immediate upstream gene, CG4585, which is truncated, thus producing transcripts containing multiple stop codons in all three frames within 54 bp of the breakpoints. Although it is formally possible for TBPH to be spliced into a CG4585 transcript, no such events were detected in our RNA-seq data.

### Generation of GAL4 and UAS lines for rescue and overexpression

For rescue and overexpression of TBPH we used the GAL4/UAS heterologous expression system (Brand and Perrimon 1993). On the basis of the distribution of chromosomal proteins and histone marks available at modENCODE (Kharchenko *et al.* 2010; Hoskins *et al.* 2011) we predicted the likely promoter region of TBPH to be contained within the first large intron, 5′ UTR and upstream sequences between TBPH and the adjacent gene CG4585. We designed primers flanking this region (GGTACCAAGGCTGCTAGAAACGAGGA, CCGCGGCATTTCCATGGCGAGCTAAT) and subcloned the resulting product of PCR amplification from a preparation of Canton-S wild-type genomic DNA into a TA cloning vector (Invitrogen). These primers contained ectopic *Kpn*I and *Sac*II restriction sites, allowing for directional cloning of the insert into the pPTGAL vector. The resulting TBPH-GAL4 plasmid was injected into embryos (Best Gene Inc, Chino Hills, CA), and the resulting transformants were mapped and balanced. To generate rescue constructs of the endogenously expressed TBPH gene, we designed oligonucleotides complementary to regions (GAGCGTGGAACGTACAGTGA, GGTACCACATCATTGGGTGACA) flanking the 5′ and 3′ ends of the coding region, with an introduced *Kpn*I site at the 5′ end of the C-terminal primer. Total RNA was isolated from wild-type adult flies homogenized in Trizol (Invitrogen) and single-stranded cDNA prepared using the superscript II kit (Invitrogen). PCR resulted in three bands of approximate size 1.2 kb, 1.6 kb, and 1.8 kb, which were then TA cloned into pCR4. The inserts were sequenced with vector primers and determined to be consistent with FlyBase transcript annotations “RA” (1.2 kb form) and “RB” through “RF” (1.8 kb form). The 1.8 kb full-length wild-type isoform of TBPH was chosen for rescue and overexpression studies and was subcloned into the pPUAST vector at the *Eco*RI and *Kpn*I sites and used to transform embryos (BestGene). To obtain rescue of TBPH loss-of-function mutants, strains of genotype G2/CyO[GFP]; UAS-TBPH and G2/CyO[GFP]; TBPH-GAL4 were crossed, and the resulting larvae were screened for lack of GFP, 100% of which express TBPH under the control of the proximal TBPH promoter region.

### Locomotion assays

Third instar larvae were removed from food, rinsed briefly in water, and placed on a 90 mm petri plate with 2% heat-sterilized agarose. The path of travel for 3–5 larvae was traced for 5 min. Plates were photographed and the distance traveled analyzed using ImageJ software (http://imagej.nih.gov/ij/). Adult locomotion assays were carried out as described by S. Benzer (1967).

### TBPH antisera and immunofluoresence

Full-length TBPH was amplified by PCR and TA-cloned into an inducible system with His-tag, pET 200D (Invitrogen). The resulting plasmid was transformed into BL21 cells, induced with IPTG (Invitrogen), and then the expressed His-tagged protein was purified on nickel agarose. Protein samples were confirmed as full-length TBPH by the OHSU proteomics core and used for immunization of rabbits (Proteintech, Chicago, IL). The antisera recognized a 58 kDa band in wild type larval CNS on Western blots which was absent from nervous tissue of TBPH mutant larvae (Figure 1). For immunofluorescence, tissue was fixed in Histochoice (AMRESCO, Solon OH) for 10 min at room temperature and incubated overnight at 4° with primary anti-TBPH (1:200) in 2% normal goat serum with 0.2% Triton X-100. After washing, tissue was incubated for 2 hr at room temperature with Alexafluor 647-conjugated goat-anti-rabbit secondary antibody (1: 10,000, Invitrogen/Molecular Probes, Eugene, OR), washed, and mounted in 80% glycerol. Images were captured with a confocal microscope (BioRad Radiance 2100) using excitation wavelengths of 543 nm (DsRed) or 647 nm (Alexafluor 647 secondary antibody) and visualized with an exclusion filter at 570–700 nm (DsRed) or 660–700 nm (Alexafluor).

### Library construction and massively parallel sequencing

Central nervous systems were dissected from 3^rd^ instar wandering larvae, and total RNA was isolated using TRIzol (Invitrogen). Nervous tissue from 20 animals was pooled for each sample. We sequenced samples from two control genotypes, A1, the precise excision line (*n* = 3), and D42-GAL4 > UAS-LacZ (*n* = 3). These were compared with G2 null mutants of TBPH (*n* = 3) and D42-GAL4 > UAS-TBPH (*n* = 3), respectively. For rescue lines, we used G2 homozygous larvae with UAS-TBPH under control of TBPH-GAL4 (*n* = 2). RNA-seq libraries were constructed using Illumina (San Diego, CA) mRNA sequencing kits. Total RNA was subjected to two rounds of oligo-dT purification and then chemically fragmented to approximately 200 bases. Fragmented RNA was used for first-strand cDNA synthesis using random primers and SuperScript II. The second strand was then synthesized using RNaseH and DNA Pol I. Fragment ends were repaired using T4 DNA polymerase, Klenow DNA polymerase, and T4 polynucleotide kinase. A single “A” was added to the 3′ ends of each fragment using Klenow fragment (3′ to 5′ exo minus). Adaptors were ligated to the fragments, the final product was run on a 2% agarose gel, and the region corresponding to 300 bp was then recovered. Templates were enriched by 15 cycles of PCR using Phusion DNA polymerase. The PCR primers also included indexing sequences to “barcode” the samples. PCR was followed by a second gel to separate PCR product from unincorporated primers. Libraries were validated by examining their profiles on an Agilent Bioanalyzer (Santa Clara, CA). Libraries were then diluted according to Illumina’s instructions for application to the flow cell using the Illumina Cluster Station. All 14 samples were randomly assigned to lanes at 2 samples per lane. The flow cell was run for 76 cycles of sequencing on an Illumina GAIIx sequencer with an additional six cycles for the indexing. The output sequences were aligned to a reference *Drosophila* sequence using Illumina’s CASAVA package. The resulting sequence files were uploaded to Genesifter (Geospiza, Seattle, WA) for analysis.

### Bioinformatics

Analysis of predicted binding sites was carried out with release 5.33 of the annotated genome of *Drosophila melanogaster*. Scripts were composed in python (http://www.python.org). Gene regions or other sequence features (*e.g.*, introns or exons) were queried using one or more of three different regular expressions corresponding to published TDP-43 binding sites in mouse or human. The regular expressions were 1:(GT){3,}T{3,}, 2:(GT){2,}(GTA){1}(TG){3,}, and 3:(GT){4,}[^T{3,}], and their reverse complements, corresponding to the canonical TDP-43 motifs (UG)_m_U_n_, (UG)_n_, and the novel (UG)_m_UA(UG)_n_ (Buratti and Baralle 2001; Sephton *et al.* 2011). For searches of single stranded molecules (introns or mRNAs), only the forward regular expressions were used. Differentially expressed genes were compared with predicted targets of TDP-43 binding in R and SQLite.

### Statistical analyses

Genetic rescue of TBPH mutant in genotype G2−/−; TBPH-GAL4 > UAS-TBPH was calculated using 10^5^ samples from a Bayesian model with the JAGS software package (Plummer 2003) to estimate the ratio of survival probabilities between rescue and mutant phenotype. The model was based on multinomial/Dirichlet and binomial/beta conjugate distributions (Gelman and Hill 2006). The genetic frequencies were constrained by an informative prior distribution to be within a few percent of the theoretical Mendelian frequencies. The total progeny (including unobserved embryonic and larval lethality) were estimated from a normal approximation to the binomial distribution using the number of observed adults expressing the dominant visible balancer marker (viable nonmutants). Estimation of gene expression was carried out using Genesifter (Geospiza, Seattle, WA). Gene expression values were normalized for each sample by indexing number of reads per kilobase per mapped million reads of each biological sample (Mortazavi *et al.* 2008; Pepke *et al.* 2009; Wilhelm and Landry 2009) and reported as log_2_ values. There were three biological replicates for each genotype (null G2, control A1, D42 > LacZ, D42 > TBPH) except for rescue (G2; TBPH > TBPH), for which there were two replicates. For pair-wise comparisons of gene expression, *t*-tests were performed on genes with a minimum average quality of 10 reads in at least one genotype. We also report the edgeR *P* values adjusted for multiple pair-wise comparisons (Benjamini and Hochberg 1995). For pathway and ontology analysis, we used DAVID bioinformatics suite at http://david.abcc.ncifcrf.gov/ (Huang *et al.* 2009a, Huang *et al*. 2009b). We used the 7897 genes derived from control gene expression described in the text as our background. Annotation clustering was performed with stringency set to “high.” Enrichment scores represent the negative log of the average *P* value in each cluster. We report enrichment scores of 1.3 or greater (corresponding to log_2_ = 0.05 cutoff value) for categories with at least 5 genes in the result set.

### Analysis of splicing

For analysis of splicing in mutants *vs.* control, both genotypes had a minimum quality of 200 reads and were indexed according to Genesifter’s “correlation coefficient” method (Eisen *et al.* 1998). Genes that had an index less than 0.7 and that also contained predicted TDP-43 binding sites were selected. For specific exon-exon junctions of biological interest, we used a beta binomial conjugate pair to estimate the *P* value for a binomial event having a particular splice pattern (Gelman and Hill 2006). Successes were defined as counts of a specific exon-exon junction of interest and failures as any exon junctions involving the first exon and an alternate second one. Alternatively, failures were defined as all other exon-exon junctions from the same gene. We chose a beta (1/2, 1/2) prior (Jeffreys 1946) to reflect the biologically reasonable assumption that reads across specific exon-exon junctions tend to be either very uncommon or common.

## Results

### Generation of TBPH mutant

The fly ortholog of human TDP-43 is the Tar DNA-binding protein homolog (TBPH). We used imprecise p-element excision to generate TBPH deletion mutants. From this we recovered a balanced lethal line, TBPH[xG2], referred to hereafter as “G2,” that deletes the promoter region, 5′ UTR and most of the first intron. G2 fails to complement a deletion covering the same region as TBPH, and it also fails to complement previously isolated alleles of TBPH (Feiguin *et al.* 2009). Antibodies raised against *Drosophila* TBPH fail to recognize bands of the expected size on Western blots of CNS isolated from homozygous G2 mutant compared with control (Figure 1A). As previously reported for loss-of-function mutations in TBPH (Feiguin *et al.* 2009), G2 homozygous larvae also exhibited severely reduced locomotion in larval crawling assays (Figure 1B). Consistent with our observation that ubiquitous expression of TBPH RNAi is lethal in the late pupa (unpublished data), the G2 allele is 100% pupal lethal. Also, consistent with G2 being a loss-of-function mutation in TBPH, expression of a UAS-TBPH transgene either in motor neurons with the D42-GAL4 transgene (Figure 1E) or under the control of the endogenous promoter region (TBPH-GAL4, Figure 1C) was sufficient to partially rescue lethality (10.7% survival to adult, >9.6:1 ratio of rescue:mutant, 95% confidence, n = 393) and the crawling phenotype in G2 homozygous larvae (Figure 1B). Thus, TBPH-GAL4 likely partially replicates the endogenous expression pattern of TBPH and is an appropriate driver to use as a rescue transgene in our expression profile study. Together these data indicate that the G2 allele retains the coding sequence but does not express protein in the CNS of larvae where it is normally found. Therefore, G2 is a null allele or at least a severe hypomorph of the TBPH gene.

### Overexpression of TBPH is toxic and causes locomotion defects

Many studies have used overexpression of human TDP-43 (hTDP-43) to model ALS. In particular, expression of hTDP-43 in motor neurons cause climbing deficits in adult flies (Li *et al.* 2010). To compare the effects of loss-of-function with overexpression, we drove expression of TBPH pan-neuronally or in more restricted sets of cells. TBPH mis-expression had broadly deleterious effects, causing early lethality with a variety of different cell-type specific drivers (data not shown). Pan-neuronal expression of TBPH was highly toxic, leading to 100% lethality with 90% lethality as first instar larvae (unpublished observations). By contrast, overexpression of TBPH in motor neurons (D42 > TBPH) had no effect in larval stages (Figure 1B), and adult flies exhibited significant climbing deficits relative to controls within a week of eclosion (supporting information, Figure S1). By the end of the second week of adult life, D42 > TBPH flies were unable to scale the sides of a test tube in the same assay (Figure S1). Thus overexpression of TBPH, the fly ortholog of TDP-43, is toxic to neurons. Immunocytochemistry of larval nervous tissue overexpressing TBPH using the D42-GAL4 driver showed extensive expression in the cytoplasm of motor neurons (Figure 1E). Although the staining shows clear enrichment of expression in the cytoplasm, we were unable to detect endogenous TBPH to determine whether overexpression leads to loss of endogenous TBPH from the nucleus.

### Loss of TBPH results in increased transcript abundance in the CNS

**Figure 2:**
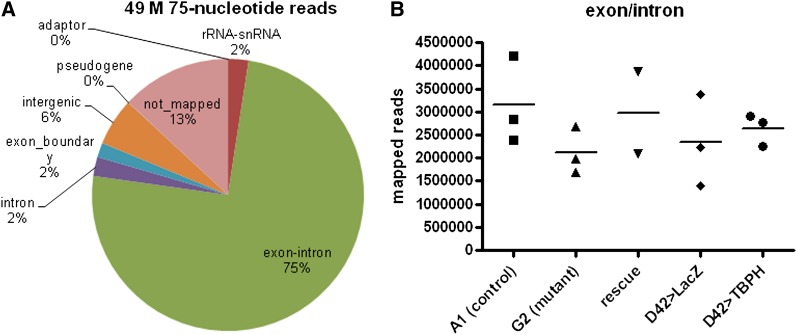
Summary of read counts and read types from RNA-seq. (A) The proportion of reads mapping to each class of RNA. Exon-intron includes reads that mapped to exon regions but may overlap introns. (B) Total number of reads mapping to exons (exon-intron category as described in panel A) for each biological sample. Horizontal bars indicate the mean.

To determine the effects of loss of TBPH function on transcript abundance in the larval nervous system, we performed RNA-seq on genotypes in which we manipulated endogenous TBPH expression levels, first by removing and then restoring expression in loss-of-function animals (rescue). We obtained approximately 49 million reads with an average of 3.5 million reads per sample. Seventy-five percent of the reads mapped to exons, as expected for a sample consisting of poly-A(+) selected RNA. Two percent of the reads were mapped to exon/exon boundaries indicative of splicing. rRNA/snRNA accounts for only 2% of the reads, suggesting a very efficient selection process, as these are the most abundant species in total RNA. These statistics are summarized in Figure 2A. We estimated wild-type gene expression by combining the six control samples (two genotypes), resulting in a count of 7897 genes, about half of the Drosophila genome. We pooled six samples in this way only to simulate the depth of coverage in other pair-wise comparisons in the study that involve five or six samples. Two different genotypes were used as controls in this study to reduce genetic background differences between the loss-of-function and overexpression comparisons. The A1 line, a p-element revertant (see *Materials and Methods*) has the same genetic background as G2, and we used it for the loss-of-function control genotype. GAL4 expression itself has been reported to promote a transcriptional response in Drosophila (Liu and Lehmann 2008), so some fraction of the genes may result from irrelevant GAL4 activity. Therefore, we used D42-GAL4 > UAS-LacZ as a control for ectopic GAL4 transcriptional activation and overexpression of foreign protein in the overexpression experiment.

In TBPH loss-of-function CNS, we detected 910 differentially expressed (DE) genes, of which 112 exhibited a 2-fold or greater change (Table 1 and expression data in Table S1). Of these 910 DE genes, the majority, 681 genes (75%), were upregulated compared with control with 229 genes (25%) downregulated. Likewise, the majority of 2-fold differentially expressed genes were upregulated (85 genes). To determine which of these 910 differentially expressed genes were affected due to the loss of TBPH expression, we replaced TBPH in the G2 mutants by expressing TBPH under the control of its endogenous proximal promoter region. We then compared the CNS expression profile of these rescue larvae with A1 controls, and identified all the genes in this set that were not differentially expressed in the rescue experiment but that were differentially expressed in the mutants. Of the 910 differentially expressed genes identified in mutant larvae, 398 (44%) were completely rescued, and an additional 76 genes (8%) were partially rescued. These 474 genes likely represent the best-candidate genes whose expression depends on TBPH. The proportion of upregulated and downregulated genes in this group was similar to all differentially regulated genes in mutant animals: 346 (73%) were upregulated and 128 (27%) were downregulated in mutants. We interpret these data to mean that our rescue experiment results in a partial restoration of endogenous TBPH activity. This interpretation is supported by the partial rescue of larval crawling behavior (Figure 1C) and lethality.

**Table 1:** Summary of RNA-seq results

Condition	Upregulated	Downregulated	Total
TBPH null (G2 *vs.* A1)	681	229	910
TBPH null > 2-fold change	85	27	112
Rescued and partially rescued*^a^*			
(G2;TBPH > TBPH)	346	128	474
Null with TBPH binding site	308	92	400
Null, rescued with TBPH binding site	134	47	181
Overexpression (D42 > TBPH *vs.* >LacZ)	159	464	623
Overexpression > 2-fold change	23	28	51
Overexpression with TBPH binding site	33	237	270
Null + overexpression*^a^*^,^*^b^*	48	9	79

In control CNS, 7897 genes were expressed. Genes in row 9 do not sum to total because some genes in this set changed in the same direction between null and overexpressed.

a The direction of change refers to the original null-mutant gene expression data set.

b The mathematical intersection of null and overexpression datasets.

When TBPH was overexpressed in motor neurons under the control of the D42-GAL4 transgene, we observed 623 differentially expressed genes, with 51 genes showing changes that were 2-fold or greater (Table 1, Table S2). In contrast to the loss-of-function data, the majority (464) of differentially expressed genes were downregulated (74%), and only 159 (25%) were upregulated compared with control. These statistics are summarized in Table 1. These data suggest that modulating TBPH expression in the nervous system dramatically alters gene expression, and that TBPH plays a direct or indirect role in transcription or message stability. To our surprise however, only 79 genes were shared between the mutant loss-of-function and overexpression datasets. Of these 79 genes, 57 (72%) were regulated in opposite directions—48 (84% of 57) were increased in mutants and decreased in overexpression CNS.

### Identification of genes with putative TBPH binding sites

**Figure 3:**
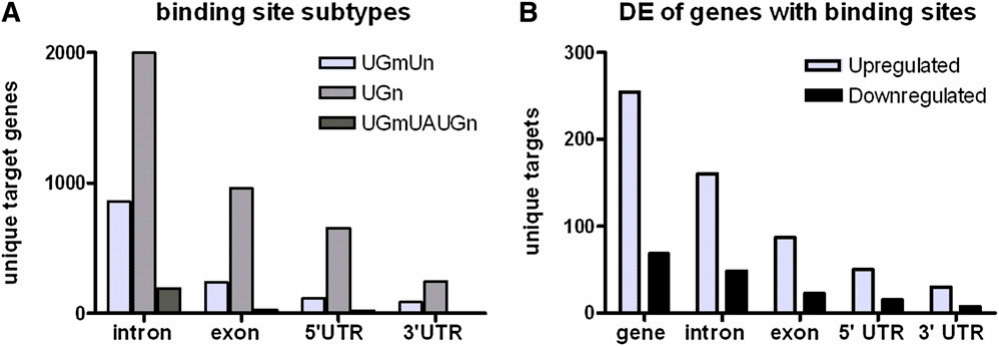
Genomic distribution of TBPH binding sites. Genes with more than one TBPH target binding sequence are counted only once. (A) Distribution of three reported TBPH binding motifs (described in *Materials and Methods*) among different RNA structural subdomains. (B) Distribution of TBPH binding sites among upregulated and downregulated genes in RNA-seq of TBPH knockout.

TDP-43 is an RNA-binding protein with two conserved RNA-binding motifs. The high degree of homology between the two RNA-binding motifs of Drosophila TBPH and human TDP-43 and the finding that they can functionally substitute for each other (Feiguin *et al.* 2009; Lu *et al.* 2009; Li *et al.* 2010) suggest that the target binding sequence is also conserved. Therefore, to predict direct mRNA binding targets of TBPH and relate these predictions to our expression data, we surveyed the Drosophila genome for potential TBPH binding sites. Using previously identified TDP-43 binding site sequence motifs (see *Materials and Methods*), we identified 3742 putative target genes, about 24% of 15,065 annotated genes. Of the 3742 predicted targets, 3018 (81%) were expressed in control CNS (7897 genes), constituting a significant (*P* < 1e−369) enrichment of putative TBPH target genes in the nervous system over genomic background. We compared these genes with the list of TDP-43 targets in mammalian nervous tissue as previously identified by CLIP-seq (Sephton *et al.* 2011). Using one-way BLAST against Drosophila proteins, we identified 2612 unique Drosophila orthologs (17% of genome) that mapped to 3647 of the 4338 genes from the Sephton *et al.* (2011) study (using expect cutoff 1e−7). Of these 2612 Drosophila orthologs, only 1053 also had predicted TBPH binding sites. Of these, 1007 (96% of 1053, *P* = 4.48e−172) were expressed in controls, suggesting a very high enrichment for orthologs of mammalian TDP-43 targets in the larval nervous system. The majority of predicted binding sites in Drosophila were found in introns rather than exons, 5′ UTRs, and 3′ UTRs (Figure 3A). We did not detect any bias within these classes of RNA for different motifs.

Next, we compared our predicted RNA targets to the expression data. We identified 400 genes (44% of 910) that were differentially expressed in TBPH loss of function. This number constitutes significant enrichment (*P* = 5.44e−5) compared with 38% of genes expressed in nervous tissue with binding sites. Similarly, 44% of upregulated genes in loss-of-function mutants contain TBPH binding sites (*P* = 7.7e−5). For the genes that were differentially expressed in overexpression samples, although there was no overall enrichment for binding sites, there were 200 genes (43%) that were downregulated and had binding sites, representing a significant enrichment (*P* = 1.16e−2) compared with all CNS genes with binding sites. Twenty-six of the genes that contained putative TBPH binding sites were identified in both loss-of-function and overexpression data sets. The expression levels of 20 out of the 26 genes changed in opposite directions comparing knockout *vs.* overexpression (Table S3) (*i.e.*, upregulated in mutants and downregulated in overexpression or vice versa).

We also identified 181 differentially expressed genes in loss-of-function samples that contained binding sites and were either fully rescued or partially rescued (Table S4A) by TBPH expression. Of these 181 genes, we identified a subset of 52 genes that were orthologous to one or more previously identified TDP-43 binding targets in CLIP-seq studies (Sephton *et al.* 2011). These genes are listed with the mammalian orthologs in Table 2.

**Table 2:** Conserved TDP targets whose differential expression in G2 mutants was restored in rescue experiment

Flybase_ID*^a^*	Mouse TDP Target*^b^*	Molecular Function*^c^*	Biological Process*^d^*
Rescued expression, downregulated in mutant			
Adar	NM_001111055, NM_001111056, NM_001111057, NM_031006, NM_012894	RNA-binding protein	RNA editing
baz	NM_031235	Phosphatidylinositol binding	Asymmetric division, planar polarity, synapse assembly, other
CG15822	NM_001134514	No data	
CG17754	NM_001047093	No data	Phagocytosis
CG32226	NM_001037191	Membrane sugar binding	
CG33214	NM_017211	Golgi membrane protein	
CG5214	NM_001006981	Succinyl transferase	Tricarboxylic acid cycle
chrb	NM_080906	No data	Cell death, signal transduction
dlg1	NM_031639, NM_019621, NM_022282, NM_022599, NM_022940, NM_012788	Guanylate kinase, egfr binding	Synaptic transmission, basal protein localization, other
MED1	NM_001134361	Mediator complex	RNA Pol II cofactor
pgant2	NM_001100863, NM_001012109	N-acetylgalactoseaminotransferase	Golgi, posttranslational modification
Pk61C	NM_031081	Kinase	Receptor mediated signaling, cell-growth
Sema-1b	NM_001108526	Receptor	Axon guidance
TBPH	NM_001011979	RNA-binding protein	Regulation of splicing
Rescued expression, upregulated in mutant			
Amph	NM_022217,NM_053959	Synaptic vesicle protein	Exocytocis; neurotransmitter secretion
AP-1γ	NM_134460	Clathrin adaptor complex	Neurotransmitter secretion; Notch signaling
att-ORFA	NM_001100860	No data	
cact	NM_030867	Transcription factor binding	Nervous system development, environmental insult, other
CG3308	NM_001109252	Endodeoxyribonuclease	
CG33181	NM_001108742	No data	
CG34127	NM_134336, NM_053868	Neurexin family protein binding	Neurogenesis, phagocytosis
CG34353	NM_001163168, NM_001163169	No data	
CG4293	NM_001024984	Arginase	
CG4400	NM_001106731, NM_001009605	SIN3-type complex	Assembly of HDAC complexes
CG6287	NM_031620	Phosphoglycerate dehydrogenase	Serine biosynthesis
CG8223	NM_001005543	No data	
CG9705	NM_152790, NM_001170542	Transcription factor	Unknown
Clic	NM_031818	Chloride intracellular channel	Adult lifespan, oxidative stress
dve	NM_001109306	Transcription factor	Copper import, morphogenesis
Exn	NM_001136241	Rho-GTP exchange factor	Regulation of neurotransmitter secretion
form3	NM_001106437	Actin binding	Tracheal development
Hph	NM_001004083	Oxidoreductase	Response to DNA damage and hypoxia
l(1)G0289	NM_001108422	No data	
MAPk-Ak2	NM_001025761, NM_001164043, NM_178102	Kinase	Cell adhesion
Nap1	NM_133402, NM_001024789, NM_053561, NM_001012170	Histone binding	Nucleosome assembly, regulation of transcription
nvy	NM_001108657	Transcription factor	Axon guidance, dendrite morphogenesis, muscle
opa	NM_001108391, NM_001108392	Transcription factor	dpp/BMP signaling, eye development, germ cell migration, midgut
Rab35	NM_001013046	GTPase	Signaling, cytokinesis
Rala	NM_053821	GTPase	Cell morphogenesis, Notch regulation, other
rb	NM_001107532, NM_001107646	Regulates ubiquitination	Endocytosis, synaptic vesicle coating, pigment granule biogenesis, lysosomal organization
RpS15Aa	NM_053982	Small ribosomal subunit	Translation, mitotic spindle organization
Sap-r	NM_013013	Lysosomal protein, activator of lysosomal enzymes	dsRNA transport, sphingolipid metabolism
sax	NM_024486	dpp/BMP type I receptor	Morphogenesis, NMJ morphogenesis, other
Sh	NM_012971, NM_023954	Voltage-gated K+ channel	axon potential
skpA	NM_001007608	SCF ubiquitin ligase complex	Centrosome dup., neurogenesis, cell cycle
Smox	NM_019191	Transcription factor	TGF-beta signaling, axon guidance, dendrite morphogenesis, neuron development, cell cycle
spin	NM_001144991, NM_001039208	No data	PCD, dpp signaling, CNS remodel, glial migration/differentiation, NMJ remodeling
Su(var)3–9	NM_001100542	H3-K9 methyltransferase	Gene silencing
Synd	NM_017294, NM_001009966		Vesicle endocytosis, neurotransmitter secretion
Timp	NM_001109393, NM_012886	Metalloprotease inhibitor	Basement membrane organization
twin	NM_001108355	CCR4-NOT complex	mRNA polyA shortening, transcript stability
UGP	NM_001024743	Transferase	Carbohydrate metabolism

a Genes whose expression was rescued in G2 mutants when driving TBPH with the TBPH promoter region under the GAL4 system.

b Identity of mouse orthologous mRNAs found to be bound to TDP-43 in CLIP-seq experiment (Sephton *et al.* 2011).

c Summary of most informative gene ontology terms for molecular function.

d Summary of most informative biological process annotations.

### TBPH target genes are enriched for synaptic transmission, neurotransmitter secretion, and endocytosis

**Figure 4:**
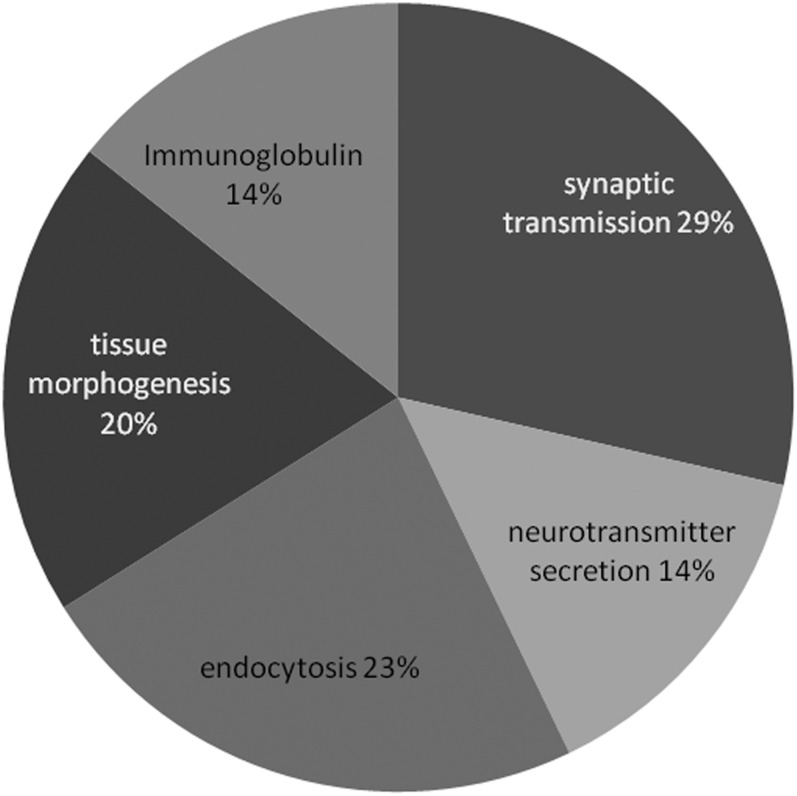
Clusters of related annotation terms enriched in rescued genes with binding sites. Frequency of different categories of clusters is represented by a single descriptive term as a function of the number of genes in each cluster. Percentages indicate proportion of enriched terms, not all genes. For details on the identities of the genes and the individual terms and significance, see Figure S2 and Figure S3.

To determine the biological role of TBPH function in the central nervous system, we focused on the set of genes whose differential expression could be rescued or partially rescued in mutant genotypes by expression of transgenic TBPH. To look for enrichment in gene ontology annotations, we used the publicly available bioinformatics tools at DAVID (http://david.abcc.ncifcrf.gov/). To eliminate inherent bias from using nervous tissue, we used the 7897 genes (Table S4C) from control genotypes as our background instead of the whole genome. Specific biological themes were evident in the enriched ontology terms; therefore, we used annotation clustering to organize enriched terms and identify the sets of genes relevant to each cluster. This analysis revealed enrichment of synaptic transmission, neurotransmitter secretion, and endocytosis (Figure 4 and Figure S2). A significant number of ion channels were also differentially expressed in G2 mutants (Figure S3, cluster 3, enrichment score: 2.29), ligand- or neurotransmitter-gated ion-channels (Figure S3, cluster 6, enrichment score 2.00), and neuropeptide receptors (Figure S3, cluster 13 enrichment score 1.55). Clustered annotation data for G2 mutants are displayed in Figure S3. We also performed this analysis on the list of rescued genes that were orthologous to previously identified orthologs of mammalian TDP-43 targets (Sephton *et al.* 2011; see Table 2). The enrichment results were similar to the set of all rescued genes (data not shown), consistent with the hypothesis that the orthologous genes identified in our study are conserved TDP-43 target genes. Therefore, instead of presenting this analysis separately, the orthologous targets are highlighted red in Figure S2 and Figure S3. We did not detect any pathway enrichment by KEGG analysis, but three important BMP pathway genes, *Smad on X* (*Smox*), *skpA*, and *saxophone*, were upregulated in mutants and rescued in G2; TBPH > TBPH animals.

### Overexpressed TBPH leads to novel changes in gene expression with some functional overlap with mutants

**Figure 5:**
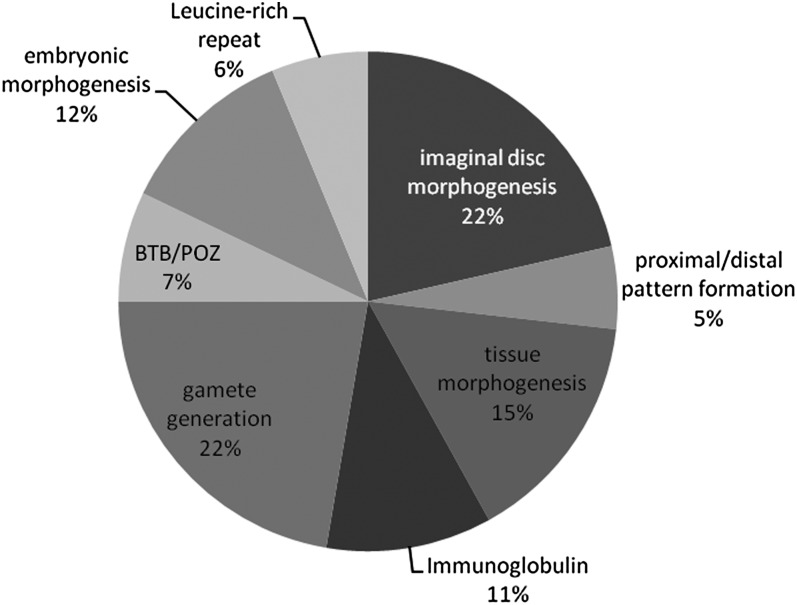
Clusters of related annotation terms enriched in TBPH overexpression DE genes with binding sites. Frequency of different categories of clusters is represented by a single descriptive term as a function of the number of genes in each cluster. Percentages indicate proportion of enriched terms, not all genes. For details on the identities of the genes and the individual terms and significance, see Figure S2 and Figure S3.

Similar to our analysis of loss-of-function altered genes, we also focused on 270 genes with TBPH binding sites (Table S4B) whose expression was altered when TBPH was overexpressed in motor neurons. As with the prior analysis, we clustered annotations to identify overlapping sets of functionally related genes. Once again, we identified enrichment of genes involved in tissue and epithelial sheet morphogenesis (cluster 3, enrichment score 2.76). Another cluster of terms related to larval metamorphosis and pupal or imaginal disc development (enrichment score 3.63) included many of the same genes (Figure 5 and Figure S4). More than a few of these genes were also shared with loss of function (*e.g.*, *u-shaped*, *steamer duck*, and *domeless*). Most shared genes—including these three—were upregulated in mutants and repressed in overexpression (Table S3). There were additional categories present in the overexpression and absent in mutants (Figure 5 and Figure S4): imaginal disc development (enrichment score 3.63), proximal-distal patterning (enrichment score 2.79), oogenesis and gamete production (enrichment score 2.32), stem cell differentiation and asymmetric division (enrichment score: 1.56), and Leucine-rich repeat proteins (enrichment score: 1.81). Immunoglobulin domain proteins were enriched in mutants (Figure 4 and Figure S2, cluster 8) and overexpression (Figure 5 and Figure S4, cluster 4). We also performed the KEGG pathway analysis for the differentially expressed genes in overexpression D42 > TBPH samples. The Wnt pathway was enriched (*P* = 0.0014, corrected *P* = 0.05 [Benjamini and Hochberg 1995]), including the Wnt receptor *arrow*/LRP-5, CG7913/PP2A, Rho-kinase or *rok*/ROCK2, *supernumerary limbs* or *slmb*/beta-TrCP, *armadillo* (beta-catenin), cAMP-dependant protein kinase 1 or *Pka-C1* (PKA), and *Smad on X* or *Smox* (SMAD3). When we considered only conserved TDP-43 targets, WNT pathway genes were clearly enriched (*P* = 3.4e−4, corrected *P* = 7.2e−3 [Benjamini and Hochberg 1995]). One of the targets, *Smox*, is an effector of *dpp*/BMP signaling. Other *dpp* pathway genes *decapentaplegic* and *saxophone* were affected in loss of function or overexpression of TBPH, although the pathway as a whole was not enriched for. Wnt/BMP pathway genes were upregulated in mutants and downregulated in overexpression samples, suggesting that TDP-43 modulates these pathways by altering expression levels.

### Analysis of altered splicing

TDP-43 is described as a splicing factor (Buratti *et al.* 2001; Buratti and Baralle 2001), so we also identified genes with altered splicing. In an attempt to identify splicing changes that were directly regulated by TBPH, we restricted our analyses to the set of genes with TBPH binding sites. There were 30 genes in mutants and 50 genes in the overexpression samples (Table 3) that met these criteria. All but 7 of the genes with altered splicing in mutants returned a higher splice index (reduced altered splicing) in the rescue samples, suggesting that the changes in splicing were due to loss of TBPH. The overall expression levels of the majority of all 80 genes with altered splicing in mutants and overexpression samples were unchanged, although expression of individual exons varied. Eight genes were misspliced in both loss-of-function and overexpression samples. These genes were *cacophony (cac)*, *CG17341*, *strawberry notch (sno)*, *CG3744*, *toucan (toc)*, *Rho kinase (rok)*, *division abnormally delayed (dally)*, and *sticks and stones (sns)*. We also clustered annotations of misspliced genes and discovered some functional overlap with differentially expressed genes. One cluster of enriched terms was related to nervous system development and cell projection morphogenesis (enrichment score 2.23). Six misspliced genes associated with cell or projection morphogenesis terms are *Rho kinase*, *Netrin-B (NetB)*, *Cadherin-N (CadN)*, *hamlet (ham)*, *Insulin-like receptor* (*InR*), *division abnormally delayed*, and the ATP-dependant chromatin assembly factor large subunit *Acf-1*. Three genes annotated with regulation of dendrite morphogenesis were *warts* (*wts*), *hamlet*, and *Cadherin N*. In Kegg pathway analysis, the Wnt pathway was again enriched (*P* = 0.041, corrected *P* = 0.42), which includes the genes Casein kinase II beta subunit (*Ckiibeta)*, *division abnormally delayed*, thus reinforcing the importance of this pathway as a potential target of regulation by *TBPH*/TDP-43 activity.

**Table 3:** Candidate splicing targets of TBPH

Gene_Identifier	Splice_Index_KO	Splice_Index_rsc	Splice_Index_OX
CG15628	NS	NS	0.012231
cac	0.035131	0.096865	0.610466
CG17341	0.374411	NS	0.120439
CG2747	NS	0.440132	0.150027
sno	0.559002	NS	0.246462
CG3744	0.623733	NS	0.283057
Rab8	NS	NS	0.294291
Pde9	0.337865	0.584513	NA
CG8179	NS	NS	0.3732
NetB	NS	NS	0.381535
toc	0.382134	0.399532	0.499912
CG31352	0.40346	0.616599	NS
CG1607	NS	NS	0.42337
InR	NS	NS	0.437603
CG6051	0.44685	NS	NS
axo	NS	0.653959	0.461114
CG34318	NS	0.539601	0.463593
CG10353	NA	NS	0.485735
CG42404	0.489271	0.282117	NS
ssp3	NA	0.623646	0.489643
CG32372	NS	0.692738	0.49013
pgant5	0.493896	NS	NS
CG32479	NS	NS	0.503011
CG14322	NA	0.341229	0.50317
tai	NS	NS	0.510347
CG9318	NS	0.660374	0.51649
wts	NS	NS	0.527354
CG34394	0.530439	0.666341	NS
smi35A	0.531367	0.67484	NS
CG11399	0.537233	NS	NS
CG10011	NS	0.561528	0.539412
CG14764	NS	NS	0.547603
CG15356		0.320978	0.548626
CG9919	0.550806	0.431502	NS
mAcR-60C	0.560996	0.511291	NS
CadN	NS	NS	0.564077
Su(dx)	0.573514	NS	NS
Surf4	NA	NS	0.574626
CG4080	0.586081	0.558437	NS
CG31150	NS	NS	0.604517
CG31739	NS	NS	0.605173
Ark	NS	NS	0.615061
CkIIbeta	NS	0.671316	0.61684
Trn	NS	NS	0.621424
CG6966	0.62369	NS	NS
rok	0.626402	0.406334	0.656452
NaCP60E	0.636625	NS	NS
ham	NS	NS	0.639029
CG3078	NS	NS	0.645845
CRMP	0.646375	0.612456	NS
CG7028	NA	NS	0.651044
htt	0.652084	NS	NS
CG34113	NA	NS	0.658926
dally	0.661246	0.669615	0.677181
l(3)05822	0.661522	NS	NS
sns	0.683637	NS	0.662555
CG12084	NA	NS	0.670823
Acf1	NS	0.500329	0.670858
dbo	NS	NS	0.6765
Nak	NS	NS	0.676703
CG11880	NS	NS	0.676724
ppa	NS	NS	0.677093
H	NS	NS	0.678118
Pkn	0.678824	NS	NS
CG15735	0.685029	NS	NS
CG6509	0.685753	0.671619	NS
CG8726	NS	NS	0.687488
CG14614	NS	NS	0.690319
CG13204	NS	NS	0.691471
Socs16D	NS	0.610877	0.69148
S	0.691913	NS	NS
spir	NS	NS	0.693973
CG34449	0.699088	NS	NS

Based on splice index comparing TBPH[G2] mutant with control A1 (Splice Index KO), rescue (Splice Index rsc), and D42-lacZ control with D42-TBPH (Splice Index OX). A lower splicing index number indicates less correlation between genotypes and hence a greater degree of aberrant splicing (see *Materials and Methods*). Only those targets with TBPH binding sites are included.

NA, insufficient data for the genotype; NS, splice index value did not meet the cutoff criteria (0.7).

We tabulated the frequency of individual species of exon-exon junctions as additional evidence for different splice forms in different genotypes (Table S5). We calculated the expected frequency of an exon junction and the likelihood of the observed junction reads relative either to alternative junctions or relative to all other mapped exon junctions for that gene. In some instances, a gene that had a low splicing index in G2 mutants but not in overexpression samples might nonetheless have significant exon junctions when examined this way. One such gene is *Apaf-1-related-killer* (*Ark*), which was only detected in the overexpression splicing index but yielded significant findings in both the mutant and overexpression for a pair of mutually exclusive alternative exon junctions (see Table S5). The most common splicing events that we detected by comparison of junction reads were those in which multiple exons were spliced out of the middle of genes, such that the first one or two exons were joined to the last or next-to-last exons, producing a transcript with little or no coding sequence. Exon junctions of this type were frequently observed in the controls but absent from mutant nervous tissue. Interestingly, three such genes, *strawberry notch*, *rho kinase*, and *Pkn*, were found to be either upregulated in mutants or downregulated in overexpression, suggesting a potential novel mechanism by which TBPH regulates these transcripts. None of these putative short transcripts is currently annotated in public databases.

### TDP-43 regulates the expression and splicing of genes associated with neurological disorders

We compared our lists of differential expression with human disease databases via “Homophila” (Chien *et al.* 2002). The results of this comparison are presented in Table S6. A large number of genes, including 211 genes from G2 mutant (plus 8 misspliced) and 142 genes (plus 21 misspliced) from D42 > TBPH overexpression were associated with human diseases. To narrow our list to the most interesting candidates, we selected the rescued genes, overexpression set genes, and misspliced genes associated with neurological disorders, and then classified them as developmental, neuropathy, movement disorder, sensory or “other” (*e.g.*, epilepsy), with some genes falling into more than one category. Twenty-three (23) direct homologs or paralogs of such genes were associated with human neurological disorders.

Among the 23 genes with Drosophila orthologs, 10 are linked to developmental disorders, the majority associated with some type of mental retardation or microcephaly. There were 9 genes associated with degenerative neuropathies including, notably, *tau*, which is both upregulated in G2 mutants and downregulated in overexpression samples. *tau* is the Drosophila homolog of the well-known neuropathy causing microtubule-associated protein MAPT (Heidary and Fortini 2001). Overexpression of *tau* is sufficient to cause neurodegeneration in a variety of contexts, including Drosophila (Jackson *et al.* 2002; Chee *et al.* 2006; Chen *et al.* 2007), and it interferes with Wnt pathway activity (Jackson *et al.* 2002). Another microtubule-associated protein *futsch*, the MAP1B homolog, was recently shown to be regulated by TBPH in flies (Godena *et al.* 2011). Consistent with the *tau* result, *futsch* was downregulated in our overexpression samples, whereas no change was detected in G2 mutants.

Six genes were associated with movement disorders, including Charcot-Marie-Tooth disease and Parkinson’s disease (PD). One of the largest effects we observed in TBPH mutants was the downregulation of L-dopa decarboxylase (*Ddc*; expression = −3.52), an enzyme whose activity is significantly reduced in the substantia nigra of PD patients (Lloyd and Hornykiewicz 1970; Gjedde *et al.* 2006). TDP-43 is reported to be deposited in Parkinson’s in 7% of cases and 19% of cases of PD with dementia (Nakashima-Yasuda *et al.* 2007), so it is plausible that TDP-43 pathology affects PD-related disease processes. TDP-43 overexpression also enhances toxicity of α-synuclein in dopaminergic neurons in mice (Tian *et al.* 2011), but TDP-43 has not yet been implicated as a requirement for dopamine production. The strong downregulation of *Ddc* in our mutants suggests that TDP-43 dysregulation or mislocalization could directly impact this pathway and thus contribute to the development of Parkinson’s. Thus, our observations of human-disease genes in TBPH knockout and overexpression indicate that some TBPH targets are orthologs of genes associated with developmental disorders of the nervous system, age-related neuropathies, and motor neuropathies. This list of genes comprises attractive candidates for follow-up studies.

## Discussion

### Implications for loss-of-function *vs.* overexpression of TDP-43 as models of ALS

There are two prevailing models for how TDP-43 dysfunction might be involved in pathogenesis of ALS/FTD-U. In one, cytoplasmic aggregates of TDP-43 are toxic, leading to altered neuronal function or survival by interfering with basic cellular function or by activation of specific cell-death pathways. Mislocalization of exogenous protein to the cytoplasm is sufficient to induce ALS-like phenotypes *in vivo* (Ritson *et al.* 2010) or degeneration and cell death in culture (Ritson *et al.* 2010). Consistent with these experiments, in our hands, overexpression of TBPH with a strong motor neuron driver induced locomotion defects (Figure 1B) and also resulted in TBPH being predominantly expressed in the cytoplasm (Figure 1E). In the second model, cytoplasmic aggregates of TDP-43 lead to depletion of TDP-43 from the nucleus as the primary lesion, resulting in loss of TDP-43 function, which ultimately causes motor neuron loss. In support of this hypothesis, overexpression of TDP-43 in primary cell culture induces aggregates, and mutant versions lacking a nuclear import signal demonstrate the ability to sequester endogenous TDP-43 to the cytoplasm (Winton *et al.* 2008). More recently, it was shown that seeding aggregation of endogenous TDP-43 with a multimer repeat of its hnRNPA1/A2 binding region was sufficient to induce its aggregation and aberrant phosphorylation in the cytoplasm of cultured cells (Budini *et al.* 2012), but no evidence was found for toxicity from such aggregations. In addition, some human disease alleles of TDP-43 that have a tendency to aggregate behave as hypomorphs when tested in model organisms (Lu *et al.* 2009; Estes *et al.* 2010). Thus, the genotypes presented in this article mimic overexpression and loss-of-function assays presented by other investigators.

For the interpretation of our data, it is important to recall that the TBPH-GAL4 promoter line expresses in a subset of neurons, and therefore, comparing CNS samples from mutants and rescues is likely to result in heterogeneous effects with respect to cell autonomy and also to include unaffected cells. Likewise, the overexpression experiments have a mixture of cell types. In both cases, differential expression analysis is complicated by the fact that some genes may be widely expressed, and hence, significant effects in a subset of cells will appear small (dilution), whereas moderately expressed genes that are restricted to the cells of interest could appear to have outsized effects. In addition, our p-element excision line contains a lesion in the 3′ half of *CG4585*, a gene adjacent to *TBPH*. *CG4585* encodes a poorly conserved putative phosphotransferase with highest expression in the larval midgut and adult ovary (Chintapalli *et al.* 2007). There is low expression in larval CNS and none detectable in adult CNS (Chintapalli *et al.* 2007), so some effects observed in larval CNS in TBPH G2 mutants may be attributable to the loss of function of *CG4585*. We addressed this by restricting our downstream analysis to genes that were rescued upon TBPH expression.

The results from the current study show that loss of function and overexpression of TBPH lead to mostly nonoverlapping cellular changes. The predominance of upregulated genes in the loss-of-function samples and downregulated genes in the overexpression samples initially suggested that the overexpression simply represented a gain-of-function phenotype, but the finding that only 79 genes out of 1533 differentially expressed genes were common in the two genotypes suggests that different cellular programs are being activated in these two situations.

### TBPH regulates ion channels, synaptic transmission, and key developmental signaling pathways

Two broad conclusions may be reached about endogenous function of TBPH from these RNA-seq data, one about the molecular function of TBPH, and the second about its role in cell biology. The majority of genes differentially expressed in loss-of-function mutants are upregulated and these are enriched for TBPH binding sites. Similarly, in the overexpression samples, downregulated genes predominate, and these are also enriched for binding sites. These findings suggest that TBPH negatively regulates the expression of a wide variety of genes. This regulation is likely to due to direct binding DNA or pre-mRNA. As a note of caution, when one excludes genes lacking binding sites, 20% of genes are still downregulated in mutants (or about 10% upregulated in overexpression, respectively). Therefore other mechanisms could also operate. Evidence for stabilization of long transcripts was described previously (Polymenidou *et al.* 2011; Sephton *et al.* 2011) and might account for some of the downregulation in mutants. In our study the mean size of downregulated genes with binding sites in mutants (19.7 kb) was larger than upregulated genes (16.2 kb) or all genes with TBPH binding sites (16.7 kb). It remains to be seen how TDP-43, which has many potential interacting partners, stabilizes some transcripts and decreases others.

The enriched clusters of annotations in both the loss-of-function and splicing set indicate that *TBPH*/TDP-43 regulates a number of ion channels and other genes involved in synaptic transmission and neurotransmitter release. Many of these genes and annotation terms (highlighted in red in Figure S2, Figure S3, and Figure S4) appear to be conserved targets of TDP-43 in studies of mammalian tissue (Sephton *et al.* 2011). A significant number of genes involved in stem cell maintenance, differentiation, and asymmetric division were also identified among the differentially expressed genes in overexpression, consistent with TBPH playing some role in neuronal differentiation and death.

Elements of the TGF-beta and Wnt signaling pathways, including *Smad on X*, *arrow*, *armadillo*, and the bmp receptor homolog *saxophone*, were upregulated in mutants and downregulated in our rescue samples. TGF-beta signaling pathways regulate growth of the neuromuscular endplate via retrograde signal from the muscle to the neuron [reviewed by Collins and Diantonio (2007)]. These genes are strong candidates for TDP-43 effectors as both *Smad* and *sax* have TBPH binding sites in their pre-mRNAs and were identified targets of TDP-43 in vertebrate cells as well (Sephton *et al.* 2011). If this finding is validated in subsequent genetic and molecular studies, it would suggest that TDP-43 plays a role in the modulation of synaptic homeostasis via the *dpp*/BMP signaling cascade. In support of such a role, the endplate of the larval neuromuscular junction is reduced in complexity and number of synapses in TBPH mutants (Feiguin *et al.* 2009). Another interesting candidate in the Wnt group is *rho kinase* (*rok*). Wnt3-induced neurite retraction is mediated by Rho-kinase (Kishida *et al.* 2004), suggesting that Rho-kinase/ROCK may regulate arborization downstream of Wnts. Sensory neurons of the larval epidermis show severely reduced complexity and length in the dendritic arbor with loss of TBPH, and both human and Drosophila TDP-43 expression drive overgrowth of this structure in the same cells, suggesting that TDP-43 levels play an important role in regulating the growth of the dendritic arbor (Li *et al.* 2010). Although this process could potentially be effected by *dpp*/*Smad*, we also identified other strong candidates for effector genes in TDP-mediated dendritic arborization. Two genes identified in the endocytosis/membrane group, *Syndapin* and *amphiphysin*, are localized to the postsynapse and regulate aspects of post-synaptic development and function ( Mathew *et al.* 2003; Kumar *et al.* 2009). In addition, we identified dendritic arborization genes *warts*, *hamlet*, and *Cadherin N* as putative splicing targets of TBPH.

Thus, our findings are consistent with a model in which TDP-43 regulates nerve transmission, process outgrowth, and synaptic homeostasis by regulation of transcript abundance and alternative splicing of key genes in important signaling pathways, including Wnt and TGF-beta. Moreover our results indicate that TDP-43 may be important for post-synaptic refinement and maintenance as well as for axon terminal differentiation on the basis of functional annotations, and they help explain why phenotypes may be observed in both compartments. One or more of our identified candidates associated with these terms may ultimately be shown to be critical for mediating TDP-43 activity in these cell types and compartments.

### Altered splicing in TBPH mutants

TDP-43 is a splicing factor with roles in splicing of the cystic fibrosis transmembrane conductance regulator (CFTR) gene (Buratti *et al.* 2001; Ayala *et al.* 2006). It was demonstrated to affect the splicing of the spinal motor neuron (*SMN*) gene, (Bose *et al.* 2008), and the human apoAII gene (Mercado *et al.* 2005). Our analysis identified a number of potentially important target genes, including the Drosophila ortholog of the Ca_v_2.1 channel *cacophony* (*cac*) and the muscarinic acetyl choline receptor (*mAchR-60C*). Mutants of *cac* in Drosophila have been reported to have reduced endplate growth at the neuromuscular junction (Xing *et al.* 2005) similar to TBPH mutants (Feiguin *et al.* 2009), and *cac* is likely to play an important role in the regulation of presynaptic vesicle fusion, thus making it an attractive candidate for follow-up studies on the perturbation of neuromuscular junction function in TBPH mutants. Orthologs of *cacophony* have also been identified as targets of TDP-43 binding in RIP-seq data (Sephton *et al.* 2011) and TDP-43 loss-of-function studies (Polymenidou *et al.* 2011).

### Comparison with other expression-profiling studies

Several massively parallel sequencing or microarray studies have been carried out previously by others. Sephton *et al.* (2011) identified RNA targets bound by TDP-43 *in vivo* by RIP-seq. We found that our differentially expressed genes were statistically enriched in orthologs of many of the same targets identified in that study, which supports a conserved role for TDP-43 in the CNS. Also, Polymenidou *et al.* (2011), using RNA-seq of TDP-43 knockdown in mouse CNS, showed a 3:2 proportion of upregulated genes to downregulated genes, a similar ratio to that seen with our data. This observation suggests that biological effects we observed were similar despite large evolutionary distances. In fact, one interesting aspect of TDP-43 biology is that many of the effects of human TDP-43 expression in model systems recapitulate the effects of overexpression of endogenous orthologs, despite the low degree of conservation of the C-terminus from the perspective of an Altschul alignment (Altschul *et al.* 1990). Here we have provided evidence that Drosophila TBPH regulates processes similar to those reported previously for mammalian TDP-43 (Polymenidou *et al.* 2011; Sephton *et al.* 2011), especially with respect to regulators of synaptic release. Moreover, a significant fraction of the genes regulated by TBPH in synaptic transmission and tissue morphogenesis was found to be conserved targets of TDP-43, most strikingly a set of genes in the *wingless*/Wnt and *decapentaplegic*/BMP pathways. Thus, our data provide support for functional conservation of *TBPH*/TDP-43 and are consistent with the hypothesis that TDP-43 evolved early in animal evolution to play a fundamental role in the differentiation and cellular function of complex nervous systems. It is important to note, however, that TDP-43 is widely expressed in both mammals and insects; therefore, our findings represent a fractional view of its role from an organismal perspective. Also, as our experiments address only changes in messenger RNA, other potential roles in the metabolism of noncoding RNA are excluded from this analysis.

In conclusion, we have identified a relatively small group of genes that are probably directly regulated by the action of TBPH. Consistent with this hypothesis and published phenotypes, a number of these targets are well-characterized promoters of motor neuron differentiation, function, and survival. Surprisingly, a large majority of genes affected in knockout were unaffected in overexpression and vice versa, supporting different mechanisms underlying behavioral and cellular defects of these two genetic models. Further studies using a combination of genetics, behavior, and electrophysiology will be necessary to validate these candidates and to elucidate more precisely how they mediate the biological effects of TDP-43 expression or loss of function in motor neurons. Such studies in flies and other model systems are needed to determine the causes of motor neuron vulnerability to these perturbations and to provide clues to future therapies.

## Supplementary Material

Supporting Information
