# Supplementary material for: Comparison of Parallel High-Throughput RNA Sequencing Between Knockout of TDP-43 and Its Overexpression Reveals Primarily Nonreciprocal and Nonoverlapping Gene Expression Changes in the Central Nervous System of Drosophila
Source: G3 (Bethesda). 2012 Jul 1;2(7):789–802. doi: 10.1534/g3.112.002998 (PMC3385985; doi:10.1534/g3.112.002998)
Supplement: Supporting Information [file supp_2_7_789__index.html]

Supporting Information 

# Comparison of Parallel High-Throughput RNA Sequencing Between Knockout of TDP-43 and Its Overexpression Reveals Primarily Nonreciprocal and Nonoverlapping Gene Expression Changes in the Central Nervous System of Drosophila

## Supporting Information for Hazelett *et al.*, 2012

**Files in this Data Supplement:**

- Supporting Information - Figures S1-S4 and Tables S1-S6 (PDF, 2.1 MB)
- Figure S1 - Overexpression of TBPH in motor-neurons causes adult climbing deficits (PDF, 98 KB)
- Figure S2 - Clusters of annotations of rescued genes (PDF, 585 KB)
- Figure S3 - Clusters of annotations of DE genes from G2 TBPH homozygous mutants (PDF, 643 KB)
- Figure S4 - Clusters of annotations of DE genes for overexpression of TBPH in motor neurons (PDF, 619 KB)
- Table S1 - Gene Expression changes in G2 mutants, rescued genes only (PDF, 167 KB)
- Table S2 - Gene Expression changes in D42>TBPH (PDF, 189 KB)
- Table S3 - List of 26 genes whose expression changed in both loss-of-function (LOF) and gain-of-function (GOF) genotypes and that contained putative TBPH binding sites (PDF, 70 KB)
- Table S4 - Entrez gene identifiers for all genes used in Ontology analyses (PDF, 119 KB)
- Table S5 - Analysis of splice-junction reads in mutant vs. control (PDF, 64 KB)
- Table S6 - Differentially expressed genes with homology to human neurological disease association (PDF, 61 KB)
