# Supplementary material for: Comparison of Parallel High-Throughput RNA Sequencing Between Knockout of TDP-43 and Its Overexpression Reveals Primarily Nonreciprocal and Nonoverlapping Gene Expression Changes in the Central Nervous System of Drosophila
Source: G3 (Bethesda). 2012 Jul 1;2(7):789–802. doi: 10.1534/g3.112.002998 (PMC3385985; doi:10.1534/g3.112.002998)
Supplement: Supporting Information [file supp_2.7.789_002998SI.pdf]

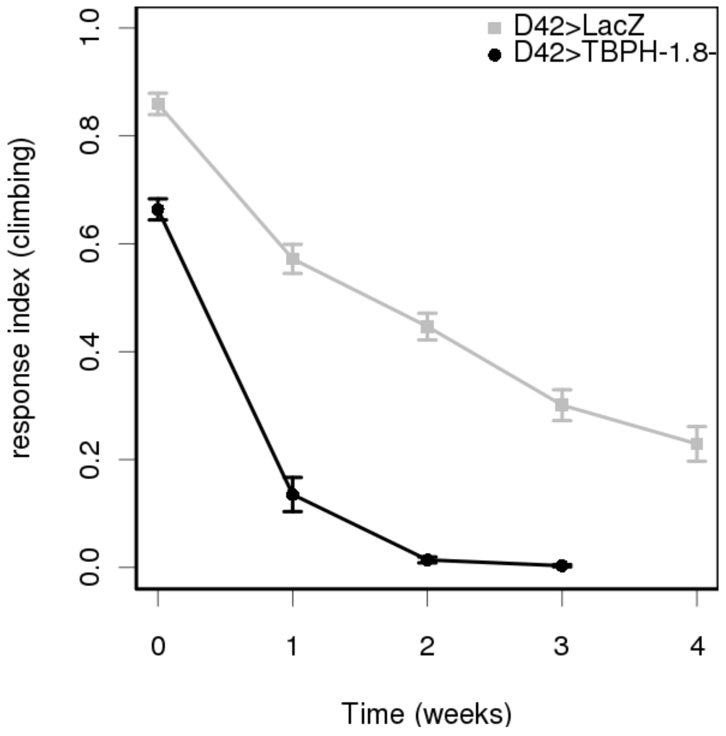

Figure S1 Overexpression of TBPH in motor-neurons causes adult climbing deficits. In the current study, these two genotypes, D42>LacZ (grey squares) and D42>TBPH (black circles) were compared to assess the effect of overexpression of TBPH in motorneurons. At each time point assayed, TBPH expression caused a significantly reduced ability to climb compared to controls ($p<0.01$) Climbing assays were performed as described in (Benzer, 1967).

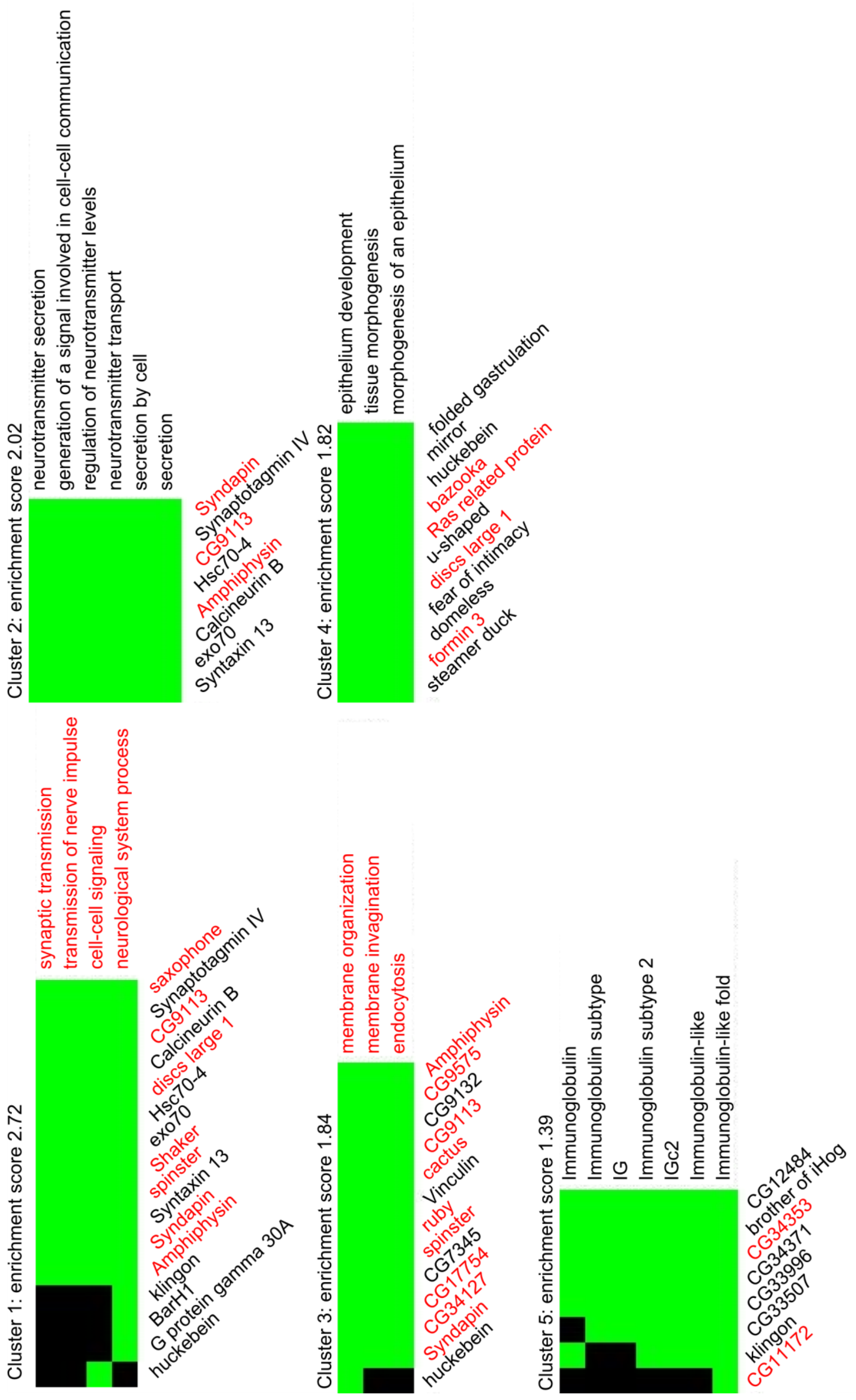

Figure S2 Clusters of annotations of rescued genes. The terms represented on the vertical axis co-occur in the gene sets represented on the horizontal axis, with enrichment scores reflecting the average log p-value for the group (Huang et al., 2009). Green squares indicate positive association reported between the gene and the annotation, black squares indicate no association yet reported. Red highlighted genes are orthologs of mammalian TDP targets identified by CLIP-seq (Sephton et al., 2011). Red highlighted terms were also enriched among TBPH-regulated Drosophila orthologs of vertebrate targets.

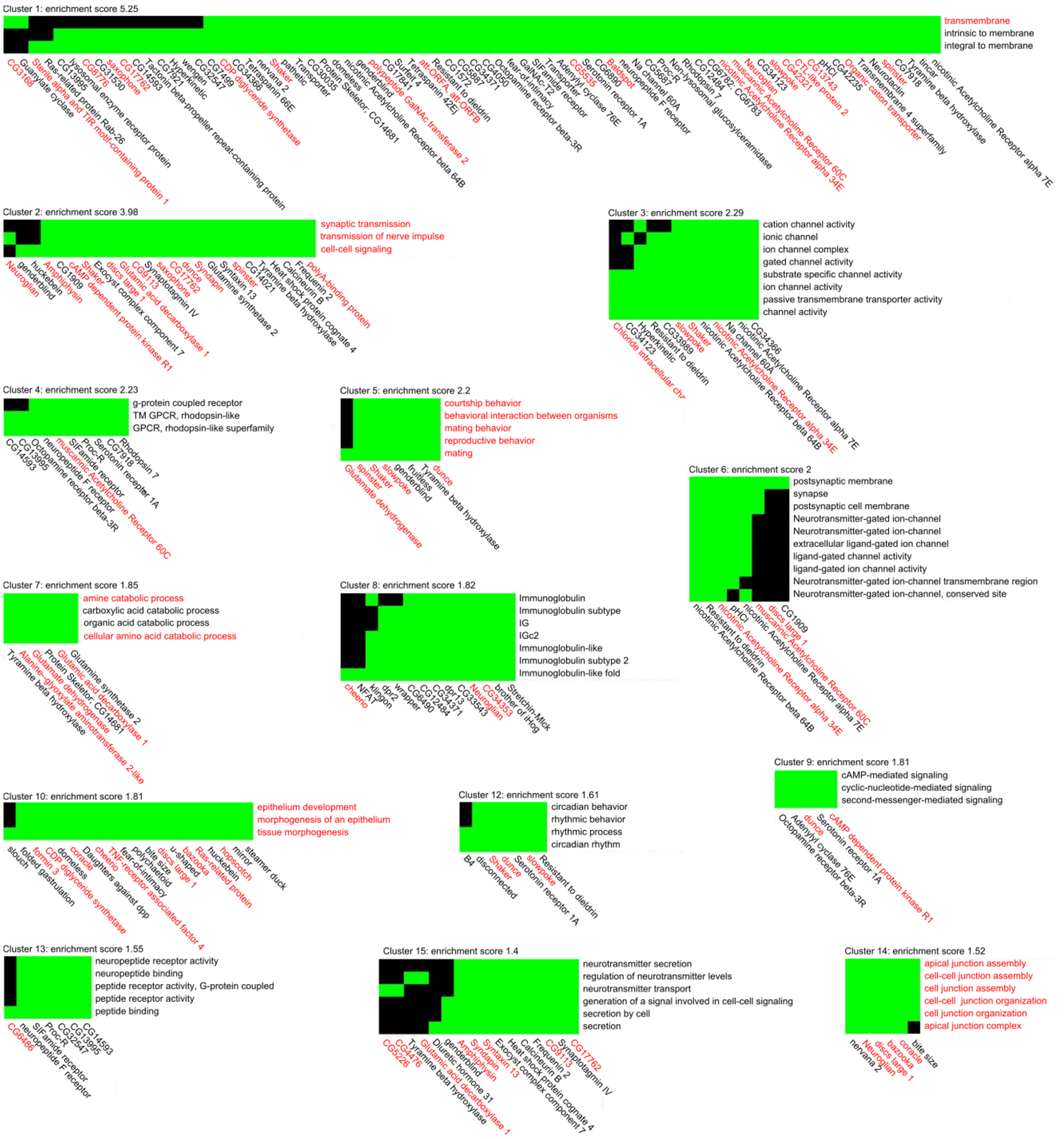

Figure S3 Clusters of annotations of DE genes from G2 TBPH homozygous mutants. The terms represented on the vertical axis co-occur in the gene sets represented on the horizontal axis, with enrichment scores reflecting the average log p-value for the group (Huang et al., 2009). Green squares indicate positive association reported between the gene and the annotation, black squares indicate no association yet reported. Red highlighted genes are orthologs of mammalian TDP targets identified by CLIP-seq (Sephton et al., 2011). Red highlighted terms were also enriched among TBPH-regulated Drosophila orthologs of vertebrate targets.

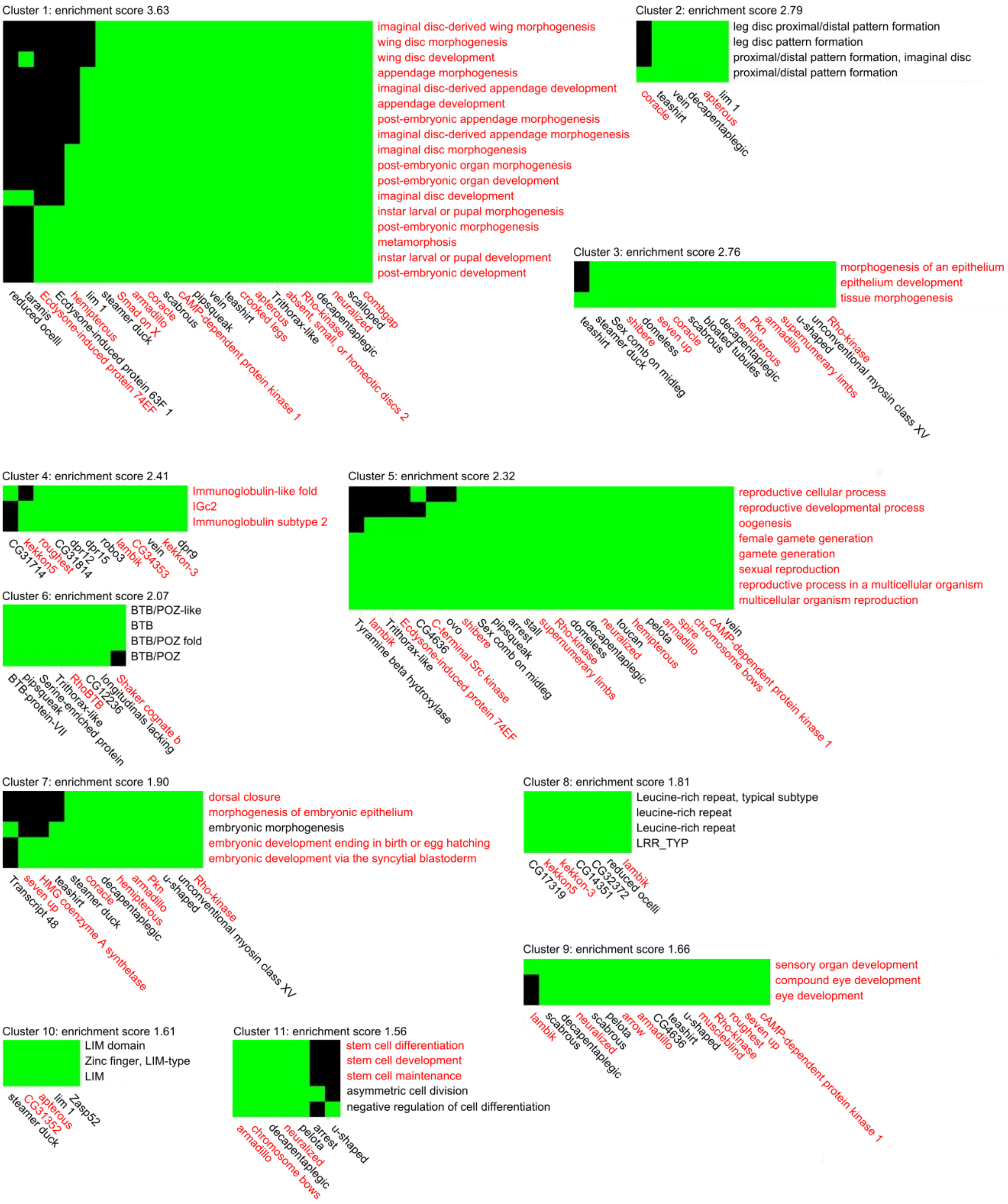

Figure S4 Clusters of annotations of DE genes for overexpression of TBPH in motor neurons. The terms represented on the vertical axis co-occur in the gene sets represented on the horizontal axis, with enrichment scores reflecting the average log p-value for the group (Huang et al., 2009). Green squares indicate positive association reported between the gene and the annotation, black squares indicate no association yet reported. Red highlighted genes are orthologs of mammalian TDP targets identified by CLIP-seq (Sephton et al., 2011). Red highlighted terms were also enriched among TBPH-regulated Drosophila orthologs of vertebrate targets.

Table S1 Gene Expression changes in G2 mutants, rescued genes only.

Flybase_ID	Entrez_ID	A1	G2	Ratio	Direction	p_value	adj_P_Val
Hsp70Aa	48581	-0.62	4.02	25.06	Up	0.047647	NA
Mur18B	32912	-1.82	2.29	17.30	Up	0.00162	8.28E-10
CG33191	251270	-1.82	1.78	12.16	Up	0.004289	NA
CG42393	7354474	-0.82	2.78	12.14	Up	0.006049	1.93E-06
CG6372	38986	-1.29	2.03	10.00	Up	0.046938	3.25E-13
CG3984	41821	5.00	1.97	8.17	Down	0.006599	5.05E-22
CG7084	42606	-0.10	2.86	7.75	Up	0.01693	7.90E-10
CG6912	41820	4.73	1.86	7.33	Down	0.009779	5.93E-28
CG31663	33363	6.25	3.73	5.73	Down	0.000233	1.87E-58
Ude	42953	-0.52	1.93	5.47	Up	0.041034	NA
CG12496	31226	0.72	2.80	4.20	Up	0.040112	0.038228
CG31115	318597	2.32	0.32	4.00	Down	0.034834	0.001154
mwh	38131	0.15	2.11	3.89	Up	0.011536	0.000107
stops	43683	2.71	0.81	3.74	Down	0.020156	1.58E-05
Cyp12c1	40037	0.68	2.58	3.73	Up	0.008916	0.000168
Ddc	35190	6.02	4.20	3.52	Down	8.03E-05	1.08E-26
CG32204	317913	5.18	6.86	3.20	Up	0.000261	2.76E-26
Ance-4	35909	0.71	2.37	3.17	Up	0.049128	0.003194
CG12355	2768954	1.20	2.84	3.12	Up	0.040599	0.101571
CG1924	32180	1.28	2.91	3.09	Up	0.022844	NA
CG4546	41922	0.72	2.30	2.99	Up	0.018114	0.000446
Rpt6R	43635	0.48	2.06	2.98	Up	0.036028	0.038104
CG9812	37709	1.79	3.35	2.96	Up	0.00975	0.000145
CG10869	33337	0.48	2.04	2.94	Up	0.015332	0.010392
CG5491	43227	3.39	4.89	2.83	Up	0.000506	4.47E-12
CG6678	42581	2.75	1.27	2.79	Down	0.037883	0.096767
CG32195	317907	2.66	4.09	2.71	Up	0.006231	2.63E-08
Obp99a	43488	8.83	7.45	2.61	Down	9.84E-05	4.35E-31
lbl	42541	0.90	2.24	2.53	Up	0.020222	0.043097
dro5	38409	2.53	3.83	2.48	Up	0.038687	0.000212
CG1681	32299	2.48	3.77	2.45	Up	0.005266	7.21E-05
CG4839	34348	0.79	2.05	2.40	Up	0.000429	0.236896
CG7800	40963	2.39	3.62	2.34	Up	0.024575	0.000277
CG32495	318053	5.77	4.55	2.33	Down	0.022823	NA
plx	40703	3.64	2.43	2.32	Down	0.013398	1.84E-08
FucTB	34260	1.84	0.63	2.31	Down	0.044129	NA
CG5704	48613	2.13	0.98	2.22	Down	0.022554	0.168479
asparagine-synthetase	2768965	5.34	6.47	2.19	Up	6.04E-05	9.41E-12
GS	32775	6.04	4.93	2.15	Down	0.002856	NA

CG4210	41832	1.87	0.77	2.14	Down	0.009741	NA
CG14082	40088	3.62	2.54	2.12	Down	0.009672	0.00206
MtnA	41202	5.93	4.87	2.09	Down	0.033243	1.46E-10
hkb	40549	2.42	3.48	2.09	Up	0.025104	0.041523
Fuca	3772574	4.59	5.65	2.09	Up	0.011602	1.74E-08
CG11714	3772566	4.59	5.65	2.09	Up	0.011602	NA
stv	39518	3.83	4.89	2.08	Up	0.020985	2.71E-06
RpS5b	41807	3.39	4.42	2.04	Up	0.019594	2.59E-05
CG32442	318031	3.49	2.46	2.04	Down	0.023831	0.018108
GstD3	48336	4.00	2.98	2.03	Down	0.020253	0.000432
CG12994	50350	1.66	2.67	2.01	Up	0.028956	0.23397
CG4660	31542	3.70	4.70	1.99	Up	0.002318	6.40E-05
lama	38610	5.79	4.81	1.98	Down	0.000906	1.09E-09
CG12003	38183	2.47	1.49	1.97	Down	0.041798	0.096767
CG42640	44513	2.23	1.27	1.95	Down	0.036189	0.110598
CG7386	38719	2.23	3.20	1.95	Up	0.017625	0.055547
CG32845	318243	1.16	2.13	1.95	Up	0.044242	NA
mRpS11	42061	2.63	3.59	1.94	Up	0.032741	0.088316
CG10425	43043	3.38	4.29	1.89	Up	0.038887	0.023524
CG13142	34434	1.66	2.56	1.87	Up	0.047215	NA
CG32857	318252	3.12	4.02	1.87	Up	0.014244	NA
ry	41605	2.91	3.81	1.86	Up	0.001791	0.085004
Skeletor	3771796	3.97	3.08	1.85	Down	0.01303	NA
Skeletor	3772559	3.97	3.08	1.85	Down	0.01303	NA
CG8132	41121	4.00	3.11	1.85	Down	0.010685	0.000117
Sox21a	39567	2.27	3.14	1.83	Up	0.014648	0.226839
CG13897	38090	4.70	5.52	1.76	Up	0.005451	4.57E-05
Sp7	40918	4.48	5.26	1.72	Up	0.003129	0.001035
Cyp6v1	33056	4.32	3.54	1.71	Down	0.037145	0.000839
mRpS18A	326141	3.59	4.37	1.71	Up	0.009219	0.03304
CG8928	32558	2.91	3.68	1.70	Up	0.023425	0.224879
CG7607	39263	3.26	4.03	1.70	Up	0.021684	0.001233
CG1545	32036	2.98	3.75	1.70	Up	0.022481	0.185298
rtv	32056	1.97	1.21	1.69	Down	0.02246	NA
CG32687	31980	2.58	3.33	1.68	Up	0.047176	0.116604
CG14210	32958	3.40	4.15	1.68	Up	0.008163	0.321152
CG11562	33200	2.61	3.36	1.67	Up	0.004382	0.204857
wgn	32849	3.95	4.69	1.67	Up	0.001873	0.002382
plx	40704	7.39	6.66	1.65	Down	0.024184	1.84E-08
CG5793	42505	2.78	3.49	1.64	Up	0.039913	0.138717
NFAT	32321	6.80	6.09	1.64	Down	0.005507	4.63E-07
CG15822	38273	3.86	3.15	1.63	Down	0.007874	0.009904

CG34325	32656	3.93	4.64	1.63	Up	0.001944	0.118087
B-H1	32724	3.95	3.25	1.63	Down	0.03526	0.039318
Fibp	40163	3.17	3.87	1.63	Up	0.042017	0.331298
CG4484	39055	4.72	4.02	1.62	Down	0.006238	0.000303
CG12582	40524	5.05	5.75	1.62	Up	0.00123	0.000821
CG1265	38517	3.24	2.54	1.62	Down	0.022864	0.065374
CG9095	32447	1.67	2.36	1.61	Up	0.004679	NA
CG8038	38889	3.68	3.00	1.61	Down	0.000686	0.074307
CG33786	3772640	4.30	4.98	1.61	Up	0.034986	NA
CG33785	3772344	4.30	4.98	1.61	Up	0.034986	NA
CG10898	41384	2.75	3.43	1.61	Up	0.047605	0.085004
mTerf3	40279	2.84	3.52	1.60	Up	0.022521	0.115059
wun2	53558	4.32	4.99	1.59	Up	0.001048	0.012494
ventrally-expressed-protein-D	117331	2.50	1.84	1.59	Down	0.019978	NA
CG42235	43136	4.94	5.60	1.58	Up	0.031781	0.001678
mRpL33	50381	2.21	2.87	1.57	Up	0.015111	NA
CG12728	31526	3.38	4.03	1.57	Up	0.033918	NA
Ac76E	40180	3.65	4.29	1.56	Up	0.010835	0.012359
CG5973	34023	1.57	2.21	1.56	Up	0.034923	NA
CG30392	246587	3.46	4.10	1.56	Up	0.018509	NA
CG31547	40663	6.85	7.50	1.56	Up	0.004023	0.000924
CG3308	42527	4.90	5.54	1.56	Up	0.03303	0.009532
Mdr49	36428	4.14	4.78	1.56	Up	0.01267	0.101641
Tsp42Ej	35620	4.07	4.70	1.55	Up	0.035375	0.296582
DNApol-eta	40438	3.72	4.34	1.54	Up	0.034143	0.24497
CG17292	34152	2.66	3.28	1.54	Up	0.033903	NA
CG33502	2768875	3.47	4.09	1.53	Up	0.000915	NA
CG12177	32326	3.07	3.68	1.53	Up	0.014878	0.100583
CG8326	32738	3.78	4.39	1.53	Up	0.024056	0.11879
CG9114	32460	4.17	4.78	1.52	Up	0.038583	0.033143
UGP	39065	4.73	5.33	1.52	Up	0.046125	0.009532
CG32432	40310	6.07	5.47	1.52	Down	0.013973	0.000259
Scp2	42015	2.76	2.16	1.51	Down	0.016838	NA
CG12746	40672	3.97	4.57	1.51	Up	0.025353	0.055679
CG11577	40084	5.20	5.79	1.51	Up	0.006348	0.014408
CG1927	38262	4.54	5.14	1.51	Up	0.004388	0.151389
Rh50	38589	3.76	4.35	1.51	Up	0.040207	0.006029
spn-A	43577	4.13	3.54	1.50	Down	0.049609	0.159309
CG16863	34784	3.52	2.94	1.50	Down	0.003112	0.155887
Ada1-2	318992	3.34	3.92	1.50	Up	0.017295	NA
xmas-1	44660	3.80	4.37	1.49	Up	0.018407	0.116528

TBPH	37781	6.97	6.41	1.48	Down	0.024987	1.52E-05
Ir62a	3885628	2.52	1.96	1.47	Down	0.031439	NA
CG32281	317953	3.30	3.86	1.47	Up	0.049016	0.265832
Ilp2	39150	5.48	4.92	1.47	Down	0.03675	0.001233
Cdk7	31441	4.64	5.19	1.47	Up	0.008875	0.08987
CHMP2B	38599	3.68	4.24	1.47	Up	0.043362	0.224078
CG9896	37652	2.47	3.02	1.46	Up	0.006961	NA
CG6171	41872	4.95	5.50	1.46	Up	0.004081	0.24497
RpI135	33210	4.99	5.54	1.46	Up	0.017992	0.033508
CG4984	37017	4.37	4.92	1.46	Up	0.033847	NA
PHDP	37788	1.61	2.15	1.46	Up	0.0264	NA
Syx13	39485	5.37	4.84	1.45	Down	0.012964	0.081678
run	33059	5.58	6.11	1.45	Up	2.08E-05	0.021595
CG32786	318210	3.49	4.02	1.45	Up	0.021204	NA
CG32372	38845	6.22	6.75	1.45	Up	0.029087	0.009431
neb	35293	5.09	5.63	1.45	Up	0.007342	0.02346
CG10949	35310	4.03	4.56	1.44	Up	0.012155	0.194738
CG18210	32469	4.27	3.75	1.44	Down	0.027657	0.03304
dpr13	3885598	4.53	5.06	1.44	Up	0.006411	0.210277
Hsp83	38389	11.29	11.82	1.44	Up	0.03774	0.000277
CG34106	4379891	2.33	2.85	1.44	Up	0.019715	NA
CG42668	42411	3.58	3.06	1.43	Down	0.030606	NA
CG32549	32822	6.03	6.55	1.43	Up	0.019216	0.046914
CG6044	37558	4.24	3.72	1.43	Down	0.006123	0.06999
form3	3346238	5.17	5.68	1.43	Up	0.003591	0.072992
CG34163	5740407	1.72	2.24	1.43	Up	0.036284	NA
f-cup	41677	3.91	4.42	1.43	Up	0.019635	0.339684
ECSIT	40732	3.83	4.34	1.43	Up	0.029851	0.221934
RNaseX25	38885	4.86	5.36	1.42	Up	0.020624	0.047892
Hr78	40378	5.95	6.45	1.42	Up	0.020004	0.028848
CG11910	43074	6.79	6.29	1.41	Down	0.011207	0.00198
CG12182	38324	4.00	4.50	1.41	Up	0.029207	0.296582
CG8315	36758	3.74	4.23	1.40	Up	0.023182	0.154832
Spc25	41585	3.77	4.25	1.40	Up	0.042351	NA
Amph	36383	4.41	4.88	1.39	Up	0.043932	0.275556
PGRP-LE	32534	3.85	4.32	1.39	Up	0.021957	0.299592
CG17739	36333	6.04	5.57	1.39	Down	0.01765	0.096767
stck	40999	5.16	5.63	1.39	Up	0.003959	NA
fog	33148	5.76	6.23	1.38	Up	0.003711	0.020909
CG10069	37427	5.20	5.67	1.38	Up	0.019111	0.08987
CG42399	33176	4.45	4.92	1.38	Up	0.045165	0.135094
chrb	39284	8.40	7.93	1.38	Down	0.006531	0.000302

CG1236	40708	5.09	5.55	1.38	Up	0.001213	0.19408
CG10702	35181	4.74	5.20	1.38	Up	0.004616	0.039449
CG15353	50191	5.16	4.70	1.38	Down	0.038835	0.001233
Timp	41248	2.59	3.05	1.37	Up	0.006019	NA
CG3735	37803	4.15	4.61	1.37	Up	0.032263	0.150456
CG17691	3355069	4.46	4.92	1.37	Up	0.039391	0.173441
Tsp66E	39017	5.74	6.20	1.37	Up	0.005103	0.104675
Ast-CC	34538	3.81	3.35	1.37	Down	0.025275	NA
Shawl	5740840	4.19	3.74	1.36	Down	0.022376	0.253844
sll	42115	4.86	5.31	1.36	Up	0.028613	0.104111
blow	35694	5.70	6.14	1.36	Up	0.004801	0.058488
CG9123	32462	5.17	5.61	1.36	Up	0.002958	0.237545
CG4400	32243	5.75	6.19	1.36	Up	0.009638	0.075906
CG5116	43081	4.65	5.09	1.36	Up	0.019542	0.081445
CG33214	40382	7.36	6.91	1.36	Down	0.013977	0.001006
CG12065	31798	6.94	7.38	1.36	Up	6.94E-05	0.03304
CG7772	32776	2.31	2.75	1.36	Up	0.034363	NA
CG5532	37761	5.17	4.73	1.36	Down	0.049171	0.013653
Mocs2	43017	3.62	4.06	1.36	Up	0.037099	NA
mirr	39441	5.94	6.37	1.35	Up	0.011402	0.039449
exo70	38959	4.80	5.23	1.35	Up	0.021261	0.289507
PCID2	39306	5.12	4.69	1.35	Down	0.005642	0.116084
CG33199	326329	5.51	5.94	1.35	Up	0.021231	NA
Atg5	31666	4.70	5.13	1.35	Up	0.038091	0.052119
CG8229	35898	5.51	5.94	1.35	Up	0.01926	0.008315
CG12576	33135	6.29	6.72	1.35	Up	0.000659	0.065903
scb	36692	5.94	6.37	1.34	Up	0.040915	0.022813
CG12104	38187	4.46	4.88	1.34	Up	0.023133	NA
CG17385	36603	5.90	6.33	1.34	Up	0.025258	NA
ATPsyn-Cf6	42759	6.27	5.85	1.34	Down	0.034014	0.170544
CG7277	33777	5.25	5.67	1.34	Up	0.00203	0.26852
Nfl	43782	5.34	5.76	1.34	Up	0.008671	0.224607
Lerp	43223	5.40	5.82	1.34	Up	0.017676	0.132346
CG7582	43492	2.76	3.17	1.33	Up	0.030215	NA
Csl4	34548	3.47	3.88	1.33	Up	0.008374	NA
Klp3A	31240	6.71	6.30	1.33	Down	0.010035	0.016135
CG5021	39025	5.07	5.48	1.33	Up	0.004589	0.226839
CG2126	43748	3.35	3.76	1.33	Up	0.012453	NA
CG4098	39854	4.73	5.14	1.33	Up	0.022651	NA
CG5147	40002	5.29	4.88	1.33	Down	0.039857	0.048322
CG10508	40342	6.54	6.95	1.33	Up	0.014882	0.014425
Syx6	40373	5.32	5.73	1.33	Up	0.032081	

kat-60L1	40715	6.08	6.49	1.33	Up	0.013325	0.160573
Fer1	2768661	4.67	5.08	1.33	Up	0.010187	0.191272
CG15651	37375	4.87	5.27	1.32	Up	0.034791	NA
CG5590	43325	6.43	6.03	1.32	Down	0.005916	0.016695
Jra	36057	5.19	5.59	1.32	Up	0.030928	0.168479
VhaM9.7-b	40389	4.82	5.22	1.32	Up	0.047265	NA
CG34127	40912	5.09	5.49	1.32	Up	0.037106	0.20893
CG13108	34240	4.72	5.12	1.32	Up	0.013656	NA
SelR	41309	4.98	5.38	1.31	Up	0.01165	NA
Nep4	42449	7.41	7.01	1.31	Down	0.000923	0.00991
CG11180	37330	4.83	5.22	1.31	Up	0.014102	0.295161
CG3011	31524	6.37	6.76	1.31	Up	0.047205	0.135586
Spn6	49803	4.54	4.92	1.31	Up	0.045003	0.329516
CG8116	41035	3.81	3.43	1.31	Down	0.021762	NA
CG7188	38936	6.13	6.51	1.30	Up	0.029443	0.13033
CG6565	34749	4.97	5.35	1.30	Up	0.025928	0.26508
SIFR	42530	4.19	3.81	1.30	Down	0.026354	NA
CG16753	38365	3.91	4.29	1.30	Up	0.044282	NA
salr	34568	6.38	6.76	1.30	Up	0.021516	0.151237
CG8379	41060	5.23	5.61	1.30	Up	0.01499	NA
CG9531	33902	3.75	3.37	1.30	Down	0.021172	NA
cact	34969	6.25	6.63	1.30	Up	0.007955	0.29018
egl	37757	5.00	5.37	1.30	Up	0.044811	NA
CG3368	43292	6.85	7.22	1.30	Up	0.00078	0.204857
pit	42595	6.03	6.40	1.30	Up	0.0126	0.209738
CG5181	34018	5.43	5.06	1.30	Down	0.04082	0.116528
SAK	40384	5.33	5.70	1.29	Up	0.015908	NA
crim	39321	3.44	3.81	1.29	Up	0.021965	NA
dve	37546	5.96	6.33	1.29	Up	0.005206	0.233345
Eip71CD	39675	5.12	4.75	1.29	Down	0.013615	0.09237
Sh	32780	5.82	6.18	1.29	Up	0.042161	0.020026
wda	42750	4.30	4.66	1.29	Up	0.048334	NA
mRpL14	31222	4.13	4.49	1.29	Up	0.042329	NA
Tollo	44497	5.31	4.94	1.29	Down	0.007832	0.169634
dpr2	3346227	2.44	2.80	1.28	Up	0.010273	NA
magu	36048	5.05	4.69	1.28	Down	0.006403	NA
CG11317	43676	5.81	6.16	1.28	Up	0.012135	0.135288
CG7339	39310	4.35	4.70	1.28	Up	0.013541	NA
CG13248	40254	6.18	5.83	1.28	Down	0.031411	0.065502
Nelf-E	38982	4.70	5.05	1.28	Up	0.022342	0.350458
pain	37985	5.17	5.52	1.27	Up	0.001303	NA
CG10189	35234	3.33	3.68	1.27	Up	0.003837	NA

CanB	44317	6.85	7.20	1.27	Up	0.03154	0.288419
Pk61C	38017	7.66	7.31	1.27	Down	0.0238	0.009904
att-ORFA	42429	3.81	4.16	1.27	Up	0.020656	NA
CG7745	36210	4.52	4.86	1.27	Up	0.0468	NA
crl	44054	5.35	5.69	1.27	Up	0.018134	NA
CG31999	43777	4.89	4.55	1.27	Down	0.019124	0.058488
CG40045	3355079	6.54	6.88	1.27	Up	0.042339	NA
bbc	36496	6.75	7.09	1.26	Up	0.045139	0.224078
foi	38976	6.60	6.94	1.26	Up	0.003194	0.24497
CG6751	36150	5.97	6.31	1.26	Up	0.017457	0.204569
Fbxl4	32378	5.17	4.83	1.26	Down	0.035628	0.296298
CG12413	43382	3.76	3.42	1.26	Down	0.03864	NA
CG3253	37861	4.34	4.68	1.26	Up	0.031798	NA
Fdh	41311	7.08	7.41	1.26	Up	0.032958	0.182525
CG42458	2768945	4.19	3.85	1.26	Down	0.02702	NA
CG17574	36415	4.32	4.64	1.26	Up	0.041039	NA
CG34195	37018	2.70	3.03	1.26	Up	0.046011	NA
CG3726	31525	4.98	5.31	1.25	Up	0.037443	NA
CG1402	31687	4.32	4.65	1.25	Up	0.031937	NA
CG12822	35737	5.62	5.94	1.25	Up	0.014699	NA
Sulf1	53437	5.80	6.12	1.25	Up	0.016031	0.143232
CG31915	319025	4.58	4.89	1.25	Up	0.011425	NA
CG5288	39031	4.98	5.30	1.25	Up	0.034309	NA
Exn	39901	5.44	5.76	1.25	Up	0.009464	0.325554
CG11191	35765	5.57	5.88	1.24	Up	0.036687	NA
ldbr	38900	5.46	5.78	1.24	Up	0.040951	NA
Aats-cys	36784	5.68	6.00	1.24	Up	0.009876	NA
CG32528	32990	5.08	5.39	1.24	Up	0.048799	NA
Psi	36889	8.59	8.28	1.24	Down	0.018049	0.072881
Hydr2	33532	6.17	6.48	1.24	Up	0.037415	NA
REG	32274	7.23	7.54	1.24	Up	0.003733	0.331982
Scgalpha	34135	4.77	5.08	1.24	Up	0.008772	NA
CG34260	5740551	4.62	4.93	1.24	Up	0.023719	NA
CG12262	38864	6.58	6.27	1.24	Down	0.010739	0.045152
hd	40642	3.68	3.99	1.24	Up	0.027412	NA
CG9436	35586	4.11	4.41	1.24	Up	0.040524	NA
Syt14	40544	4.97	4.66	1.23	Down	0.038823	0.204857
CG10366	35258	4.71	5.01	1.23	Up	0.009626	NA
CG17754	31873	7.02	6.72	1.23	Down	0.009864	0.13033
CG5823	42105	5.42	5.72	1.23	Up	0.021641	0.248311
Hmr	31988	5.83	6.13	1.23	Up	0.018436	NA
CG42337	40335	4.52	4.81	1.23	Up	0.011569	0.273226

XRCC1	31451	4.83	5.12	1.23	Up	0.007842	0.311549
Hsc70-5	36583	7.54	7.83	1.23	Up	0.013538	NA
Lip4	34450	6.02	6.31	1.22	Up	0.024495	NA
Rala	31332	5.59	5.88	1.22	Up	0.036386	NA
Vps45	41153	4.33	4.62	1.22	Up	0.026277	NA
CG6712	34631	5.20	5.49	1.22	Up	0.037521	NA
Sdic1	43984	4.54	4.83	1.22	Up	0.016682	NA
ush	33225	5.51	5.79	1.22	Up	0.015133	NA
CG3560	32586	5.28	5.56	1.22	Up	0.012233	NA
CG1789	31812	4.25	4.53	1.22	Up	0.027686	NA
MED1	40403	7.85	7.57	1.22	Down	0.00734	0.178288
Arc1	36595	8.89	8.61	1.22	Down	0.047083	0.071523
nvy	37886	6.71	6.99	1.21	Up	0.016199	NA
CG6287	34554	6.07	6.35	1.21	Up	0.047581	NA
ergic53	44679	6.79	7.06	1.21	Up	0.033977	NA
Aats-lys	31904	7.72	8.00	1.21	Up	0.008459	NA
Clic	32349	6.14	6.41	1.21	Up	0.007097	NA
CG8602	38808	6.51	6.24	1.21	Down	0.04403	0.168858
CG5343	34429	4.78	5.05	1.21	Up	0.003521	NA
CG34371	37679	7.44	7.17	1.21	Down	0.019022	0.059006
Ggamma30A	45234	8.33	8.60	1.21	Up	0.00659	NA
CG13784	34003	5.94	6.21	1.20	Up	0.016125	NA
l(1)G0289	31964	7.20	7.47	1.20	Up	0.019807	NA
baz	32703	6.22	5.95	1.20	Down	0.003991	0.065272
klg	42707	7.51	7.78	1.20	Up	0.021615	NA
Aats-val	45783	6.91	7.18	1.20	Up	0.012694	NA
Rab26	40359	5.53	5.79	1.20	Up	0.001393	NA
CG9636	40967	7.15	7.42	1.20	Up	0.029257	0.347539
AP-1gamma	31842	7.05	7.31	1.20	Up	0.049497	NA
MAPk-Ak2	44573	6.49	6.75	1.20	Up	0.027776	NA
CG11396	40296	4.48	4.22	1.20	Down	0.042361	NA
MED11	40042	4.84	4.58	1.20	Down	0.011481	0.236896
NPFR1	40754	3.84	4.10	1.20	Up	0.003066	NA
Gbeta5	31744	5.58	5.83	1.19	Up	0.015182	NA
RpS14a	47218	9.39	9.65	1.19	Up	0.040083	0.317467
geminin	35563	6.30	6.55	1.19	Up	0.015139	NA
Mer	32979	4.83	5.08	1.19	Up	0.001263	NA
grau	45871	4.99	5.24	1.19	Up	0.005611	NA
CG1637	32019	6.27	6.52	1.19	Up	0.02527	NA
GalNAc-T2	32836	6.26	6.52	1.19	Up	0.037906	NA
CG15618	33001	6.13	5.88	1.19	Down	0.02534	0.292735
p	41025	6.81	7.06	1.19	Up	0.004703	NA

CG11926	33689	4.85	4.60	1.19	Down	0.024696	NA
CAP-D2	43491	7.16	6.92	1.19	Down	0.048453	NA
CG5214	41360	7.47	7.22	1.19	Down	0.035739	0.104912
CG12592	41263	5.93	6.18	1.18	Up	0.035484	0.067867
7B2	40644	7.40	7.64	1.18	Up	0.022045	NA
CG8378	36299	6.57	6.81	1.18	Up	0.014082	NA
CG12239	31509	7.65	7.41	1.18	Down	0.026647	0.116528
Hnf4	44544	3.97	3.73	1.18	Down	0.009187	NA
CG32226	40229	7.51	7.27	1.18	Down	0.005332	0.189433
CG4577	33291	7.88	7.64	1.18	Down	0.008905	0.157082
HspB8	32955	6.44	6.68	1.18	Up	0.002243	NA
scf	38145	6.65	6.89	1.18	Up	0.00263	NA
dlg1	32083	8.02	7.78	1.18	Down	0.005125	0.182336
Sod2	36878	6.64	6.40	1.18	Down	0.035369	0.264135
CG6506	32766	4.20	3.96	1.18	Down	0.011712	NA
CG5871	42518	6.94	6.71	1.18	Down	0.02985	0.285009
CG14812	31137	3.76	3.99	1.18	Up	0.020684	NA
dome	32976	7.24	7.47	1.18	Up	0.018949	NA
RpS14b	47219	8.54	8.77	1.17	Up	0.001696	NA
CG3004	31903	6.15	5.92	1.17	Down	0.015639	NA
pgant2	33556	6.67	6.44	1.17	Down	0.024948	NA
CG6769	32770	5.52	5.75	1.17	Up	0.001558	NA
ferrochelatase	43757	5.04	5.27	1.17	Up	0.01943	NA
Adam	36037	5.74	5.97	1.17	Up	0.007047	NA
CG6512	39922	6.99	7.22	1.17	Up	0.025557	NA
xmas-2	44271	6.39	6.17	1.17	Down	0.000928	NA
Hop	33202	8.16	8.39	1.17	Up	0.036117	NA
Aats-thr	45784	8.20	8.43	1.17	Up	0.024972	NA
CG42390	42600	5.20	5.43	1.17	Up	0.000484	NA
CG8841	36336	5.74	5.96	1.17	Up	0.014236	NA
CG15646	32501	5.27	5.49	1.17	Up	0.009272	NA
CG15111	37200	5.84	6.07	1.17	Up	0.038562	NA
ham	35135	7.35	7.13	1.17	Down	0.042123	0.308393
CG9346	37381	6.01	5.79	1.17	Down	0.023752	NA
CG9253	35379	6.54	6.76	1.16	Up	0.0026	NA
Smox	31738	8.31	8.53	1.16	Up	0.044535	NA
CG31635	33928	6.49	6.71	1.16	Up	0.016871	NA
CG13995	33851	5.87	6.09	1.16	Up	0.036236	NA
CG3719	44736	5.16	5.38	1.16	Up	0.019642	NA
CG17337	35507	6.10	6.32	1.16	Up	0.016465	NA
boi	31229	5.09	4.87	1.16	Down	0.010207	NA
Adar	31130	6.21	5.99	1.16	Down	0.043778	NA

CG34317	5740224	5.15	5.36	1.16	Up	0.02048	NA
CG7950	43579	5.15	5.36	1.16	Up	0.02048	NA
Rrp1	33500	8.13	8.34	1.16	Up	0.003718	NA
shrb	35933	6.73	6.94	1.16	Up	0.041807	NA
CG1553	35730	6.63	6.84	1.16	Up	0.044575	NA
Ykt6	31706	6.18	6.39	1.16	Up	0.008528	NA
Vrp1	37521	5.87	6.08	1.16	Up	0.033444	NA
CG11448	31090	6.91	7.12	1.16	Up	0.025539	NA
rb	31381	6.77	6.97	1.15	Up	0.003607	NA
CG9171	33807	4.87	4.66	1.15	Down	0.042627	NA
Syt4	40876	8.53	8.74	1.15	Up	0.029357	NA
CG9132	2768881	6.06	6.27	1.15	Up	0.005226	NA
RpL18A	36985	10.07	10.28	1.15	Up	0.014954	NA
sti	39429	8.03	7.83	1.15	Down	0.015908	0.301703
Nlp	43560	8.82	9.03	1.15	Up	0.044512	NA
SIDL	41833	5.71	5.51	1.15	Down	0.032601	NA
tinc	42148	8.65	8.85	1.15	Up	0.041053	NA
Vinc	31201	6.38	6.59	1.15	Up	0.009122	NA
spin	45380	6.98	7.18	1.15	Up	0.025716	NA
CG33181	318916	5.73	5.93	1.15	Up	0.008862	NA
CG33096	326251	6.28	6.08	1.15	Down	0.013788	NA
sax	35731	5.76	5.96	1.15	Up	0.045167	NA
disco-r	64875	5.76	5.56	1.15	Down	0.021436	0.334537
ns4	35338	5.51	5.71	1.15	Up	0.001301	NA
CG16896	37977	5.52	5.72	1.15	Up	0.041874	NA
CG34353	5740590	5.56	5.76	1.15	Up	0.041968	NA
Bap55	36956	7.10	7.29	1.14	Up	0.004659	NA
Sema-1b	37007	7.24	7.05	1.14	Down	0.041922	0.285009
Hsc70-4	41840	12.16	12.36	1.14	Up	0.036502	NA
CG11927	33687	5.67	5.47	1.14	Down	9.57E-05	0.320212
Elp3	33649	5.19	5.38	1.14	Up	0.04858	NA
MED19	39987	6.64	6.46	1.14	Down	0.044932	NA
RpS15Aa	44150	9.45	9.64	1.14	Up	0.026626	NA
Sap-r	43662	9.59	9.77	1.14	Up	0.033072	NA
CG9170	32563	7.45	7.64	1.14	Up	0.027964	NA
CG2915	35754	7.05	7.23	1.14	Up	0.025429	NA
Hph	40633	5.89	6.08	1.14	Up	0.032973	NA
CG1518	33082	8.13	8.32	1.14	Up	0.013822	NA
CG7185	38937	9.06	8.87	1.14	Down	0.026995	0.205585
CG12424	36673	7.46	7.27	1.14	Down	0.019694	0.210774
Sesn	37755	5.02	5.20	1.14	Up	0.028161	NA
Sesn	37755	5.02	5.20	1.14	Up	0.028161	NA

CG6049	40369	5.80	5.62	1.13	Down	0.043121	0.298784
Sam-S	48552	8.15	8.33	1.13	Up	0.022739	NA
CG14213	32966	7.46	7.64	1.13	Up	0.043031	NA
CG11504	43557	6.56	6.38	1.13	Down	0.005324	NA
Ssrp	37767	8.13	8.31	1.13	Up	0.005496	NA
Rca1	33959	5.99	6.17	1.13	Up	0.036156	NA
CG4502	34002	6.72	6.89	1.13	Up	0.04501	NA
Set2	32301	7.61	7.44	1.13	Down	0.02468	NA
CG12484	37310	6.14	5.97	1.13	Down	0.043498	NA
ecd	38291	6.50	6.33	1.13	Down	0.021521	NA
RnpS1	41147	7.38	7.20	1.13	Down	0.037634	NA
Surf4	41864	6.17	6.34	1.13	Up	0.004833	NA
CG4293	31001	5.52	5.69	1.13	Up	0.019972	NA
skpA	31016	7.46	7.63	1.12	Up	0.044819	NA
Rtc1	32338	4.34	4.51	1.12	Up	0.042441	NA
CG4674	41320	5.09	5.25	1.12	Up	0.046093	NA
twin	42880	7.40	7.56	1.12	Up	0.037373	NA
CG31855	318983	5.24	5.40	1.12	Up	0.012446	NA
gfzf	40858	6.69	6.86	1.12	Up	0.007355	NA
CG5745	42498	5.43	5.60	1.12	Up	0.042093	NA
CG15744	2768909	7.41	7.25	1.12	Down	0.012221	NA
dod	33111	6.69	6.85	1.12	Up	0.049093	NA
KdelR	34427	6.91	7.07	1.12	Up	0.035286	NA
CG9147	33855	4.66	4.82	1.12	Up	0.025106	NA
CG10347	32141	5.37	5.53	1.12	Up	0.047009	NA
CG10627	39434	5.75	5.59	1.12	Down	0.009976	NA
CG5946	39336	5.72	5.87	1.11	Up	0.037411	NA
Taf1	40813	7.85	7.70	1.11	Down	0.042935	0.311162
l(3)05822	47260	6.45	6.60	1.11	Up	0.020439	NA
CG14782	31105	6.68	6.83	1.11	Up	0.015917	NA
CG8372	34121	5.33	5.18	1.11	Down	0.04695	NA
Ef1alpha100E	43736	11.11	11.25	1.11	Up	0.018765	NA
RpS13	34149	9.84	9.98	1.10	Up	0.04153	NA
Smg6	42994	6.41	6.56	1.10	Up	0.017922	NA
CG6724	34488	5.41	5.55	1.10	Up	0.027683	NA
128up	36288	5.86	6.00	1.10	Up	0.034727	NA
Tcp-1zeta	32518	8.46	8.59	1.10	Up	0.031597	NA
grp	34993	7.80	7.93	1.10	Up	0.043522	NA
CG6236	41857	6.55	6.68	1.10	Up	0.029679	NA
Rab35	33014	7.30	7.43	1.10	Up	0.036234	NA
CG9705	39875	8.22	8.35	1.09	Up	0.041692	NA
Nup154	34527	6.75	6.88	1.09	Up	0.009063	NA

bur	45830	6.84	6.97	1.09	Up	0.023329	NA
CG2025	32143	6.86	6.73	1.09	Down	0.018124	NA
CG7878	40959	6.45	6.57	1.09	Up	0.045138	NA
CG7332	32885	6.45	6.57	1.09	Up	0.005166	NA
Nap1	37798	8.69	8.81	1.09	Up	0.032857	NA
RpL7A	31588	10.76	10.88	1.08	Up	0.027734	NA
wuho	31566	6.22	6.33	1.08	Up	0.025997	NA
Cctgamma	42029	8.66	8.77	1.08	Up	0.041267	NA
Ptpmeg	38059	7.67	7.78	1.08	Up	0.022273	NA
D1	41095	9.80	9.70	1.07	Down	0.048673	NA
Tom7	35899	5.45	5.35	1.07	Down	0.028487	NA
Synd	42467	6.15	6.25	1.07	Up	0.030868	NA
opa	40605	6.25	6.33	1.06	Up	0.024776	NA
Su(var)3-9	41843	7.94	8.01	1.05	Up	0.045205	NA
CG8223	41045	8.15	8.21	1.04	Up	0.018312	NA

Terms and abbreviations: Gene_Identifier, the flybase symbol of the corresponding differentially expressed gene, A1_mean, the mean expression level of A1 p-element revertant controls, G2_mean, the mean expression level of TBPH[G2] allele (homozygous), Ratio, the fold-change of expression in G2 compared to A1, Direction, the direction of the change, p_value, the genesifter p value (see methods for parameters), edgeR p value adjusted for multiple hypothesis testing (Benjamini & Hochberg 1995). NA, the gene was not differentially expressed using edgeR analysis.

Table S2 Gene Expression changes in D42>TBPH

Flybase ID	Entrez	LacZ	TBPH	Ratio	Direction	P Value	adj_P_Val
Tpr2	45556	-1.30	3.08	20.91	Up	0.000449	NA
CG17669	37125	-0.97	3.37	20.31	Up	0.00117	2.24E-16
CG3984	41821	4.40	0.21	18.27	Down	0.000125	1.17E-24
Cyp4p2	35946	3.45	-0.43	14.67	Down	0.000221	NA
CG12607	38547	2.18	-1.62	13.99	Down	0.000269	4.53E-09
Magi	41820	4.26	0.59	12.75	Down	0.000447	2.13E-30
snoRNA:Psi28S-2263	40157	3.37	-0.10	11.04	Down	0.012306	NA
bgm	45524	1.65	-1.62	9.65	Down	0.002793	2.82E-08
CR40734	5740371	-1.30	1.47	6.83	Up	0.049309	NA
CR41548	5740812	-0.44	2.31	6.74	Up	0.038967	NA
CR41539	5740187	-0.64	2.05	6.44	Up	0.033255	NA
CR41544	5740694	0.16	2.71	5.84	Up	0.024901	NA
CG42382	40957	-0.77	1.66	5.4	Up	0.026403	0.007291
CG30015	41822	2.05	-0.32	5.18	Down	0.018011	NA
Pepck	37131	3.78	1.51	4.83	Down	0.032896	8.35E-15
CG14277	34129	1.90	-0.10	3.99	Down	0.006754	0.005358
brp	42344	2.69	0.71	3.95	Down	0.012885	2.45E-09
CG42306	40588	1.82	3.79	3.93	Up	0.01895	1.32E-11
CG6701	42443	-0.11	1.86	3.91	Up	0.003875	0.015887
Catsup	37712	3.32	1.53	3.46	Down	0.006132	0.000188
Nplp4	50190	1.14	2.85	3.28	Up	0.035451	0.026685
Msp-300	35199	0.23	1.87	3.13	Up	0.000972	0.121946
CG30187	246509	2.35	0.77	2.99	Down	0.035893	0.40095
Dhc36C	35061	2.31	0.78	2.89	Down	0.007782	0.31854
CG14946	34627	2.57	1.10	2.78	Down	0.014636	0.00177
CG2976	42294	3.28	1.82	2.75	Down	0.024819	NA
Gug	39621	1.02	2.46	2.72	Up	0.019709	NA
CG4892	53502	2.60	4.02	2.66	Up	0.022874	NA
CG13897	38090	4.70	3.31	2.61	Down	0.011887	1.71E-06
phr	35735	5.76	4.43	2.51	Down	0.001317	2.45E-14
CG31728	34731	2.99	1.70	2.43	Down	0.045172	0.098217
CG8814	42049	4.51	3.23	2.43	Down	0.04415	9.15E-07
Ir62a	3885628	3.09	1.81	2.42	Down	0.026837	NA
CG13082	35244	1.13	2.40	2.41	Up	0.018851	0.118273
CG31797	32767	2.73	1.48	2.39	Down	0.000822	0.059971
Gdi	39567	2.95	1.72	2.34	Down	0.020901	NA
CG3788	37644	3.09	4.29	2.3	Up	0.015163	7.55E-06
snoRNA:Me28S-A576	40701	0.49	1.69	2.29	Up	0.010014	NA
Doc3	40266	4.38	3.21	2.26	Down	0.00105	2.96E-05
s-cup	36371	3.08	1.90	2.26	Down	0.023767	NA

CG42796	41017	2.64	1.47	2.25	Down	0.027202	NA
CG34458	40868	4.54	5.70	2.25	Up	0.028068	6.22E-11
CG31643	43126	2.86	1.69	2.24	Down	0.042358	0.343689
5740512	5740512	0.86	2.02	2.23	Up	0.038367	NA
CG6424	32419	3.42	2.26	2.22	Down	0.01045	0.021966
Ady43A	44750	4.90	6.00	2.15	Up	0.006933	NA
CG33271	32084	0.67	1.73	2.1	Up	0.034625	NA
cni	53522	1.46	2.53	2.09	Up	0.031436	NA
stl	37619	1.93	2.96	2.04	Up	0.048928	0.077571
CG42801	4379858	3.04	2.03	2	Down	0.016104	NA
for	47878	1.89	2.89	2	Up	0.028341	0.332967
Gadd45	35646	1.32	2.30	1.97	Up	0.017649	NA
CG5160	34015	1.25	2.21	1.96	Up	0.040538	NA
akirin	40427	4.16	3.20	1.95	Down	0.000369	0.00666
CG14471	35525	2.30	1.34	1.94	Down	0.038649	0.53413
CG12911	36081	3.51	4.46	1.93	Up	0.023607	0.00128
Egfr	39566	8.20	7.25	1.93	Down	0.043681	6.81E-14
CG8552	41202	6.81	7.75	1.92	Up	0.026908	1.64E-10
CG13272	35013	2.27	1.33	1.92	Down	0.007575	0.392587
Socs36E	43583	2.40	1.48	1.9	Down	0.018836	NA
CG42700	36330	2.01	1.09	1.9	Down	0.017414	NA
CG8679	40205	6.63	5.73	1.87	Down	0.004442	NA
Catsup	37709	2.77	1.87	1.86	Down	0.000189	0.362167
Chd1	39048	4.61	3.72	1.86	Down	0.009781	NA
CG14502	42055	2.60	3.48	1.84	Up	0.033918	0.420993
CG5830	39748	7.49	6.61	1.84	Down	0.012054	4.55E-09
CG16741	40728	2.55	3.42	1.83	Up	0.03618	0.069552
Gmd	39238	3.55	2.69	1.82	Down	0.002921	NA
nopo	37083	2.79	1.93	1.82	Down	0.048664	NA
CR18854	32148	6.95	6.09	1.82	Down	0.009063	2.93E-07
Dap160	39662	2.93	2.10	1.78	Down	0.007585	NA
Gal	31761	2.74	3.57	1.78	Up	0.014134	0.116775
CG34398	5740462	3.96	3.13	1.77	Down	0.015492	0.05469
CG9951	41766	2.66	1.84	1.76	Down	0.042753	NA
TotX	31193	3.51	2.70	1.75	Down	0.028059	0.102051
mir-307-as	42879	2.33	1.52	1.75	Down	0.002714	NA
lectin-21Cb	31271	5.95	6.75	1.74	Up	0.007816	NA
CG15674	37463	2.53	1.74	1.73	Down	0.00619	NA
Acox57D-p	37445	2.06	2.84	1.73	Up	0.03783	0.191724
Ir31a	31718	5.04	4.25	1.73	Down	0.028432	0.001299
CG3732	40297	1.82	1.03	1.72	Down	0.03864	NA
osp	44110	5.32	6.10	1.72	Up	0.001689	1.17E-06
CG12594	41416	6.25	5.48	1.7	Down	0.002161	7.55E-06

Cbp53E	40422	2.96	3.73	1.7	Up	0.007211	0.125953
CG13966	35284	1.78	2.53	1.68	Up	0.047131	NA
CG18853	246511	4.43	3.68	1.68	Down	0.009838	NA
mir-278	42945	3.81	4.55	1.67	Up	0.007221	0.002298
gammaTub37C	42271	2.33	1.59	1.67	Down	0.046855	NA
Fibp	41819	3.72	2.98	1.67	Down	0.025113	NA
Adgf-E	37551	1.28	2.02	1.67	Up	0.03402	NA
salm	36991	4.12	3.39	1.66	Down	0.010389	0.126957
CG30392	246587	4.34	3.61	1.66	Down	0.000586	0.015333
Obp44a	45326	8.19	7.46	1.66	Down	0.013578	1.34E-06
CG42866	42866	3.23	3.95	1.65	Up	0.001948	NA
sec31	35877	7.89	7.17	1.65	Down	0.006275	NA
Herp	39854	5.24	4.52	1.64	Down	0.000345	0.003947
CG15100	50388	4.59	3.88	1.64	Down	0.020081	NA
Mip	39933	4.29	3.58	1.64	Down	0.021901	0.062798
CG42750	35094	3.15	2.43	1.64	Down	0.007505	NA
CG1827	35989	3.48	4.19	1.64	Up	0.016807	0.054096
E2f2	39263	3.18	3.88	1.64	Up	0.046982	1.66E-05
Fbp2	34259	4.94	4.24	1.63	Down	0.046694	0.003947
RpL27A	41092	2.13	2.82	1.62	Up	0.03443	0.384406
pdm2	32441	1.88	2.57	1.61	Up	0.032088	0.4678
l(2)05714	46066	4.16	4.84	1.6	Up	0.010277	0.048475
CG8740	35866	3.26	2.58	1.6	Down	0.003126	NA
mRpS2	33688	4.41	3.74	1.59	Down	0.023328	0.420993
CG42268	42268	3.40	4.07	1.59	Up	0.031103	0.273177
CG3394	37887	2.19	2.86	1.59	Up	0.006181	0.228537
l(2)35Bg	40916	2.98	3.65	1.59	Up	0.017085	NA
CG9140	40424	7.10	6.44	1.58	Down	0.004544	5.30E-05
Tom20	43235	3.39	2.73	1.58	Down	0.035311	0.451935
CG5946	31249	3.83	3.16	1.58	Down	0.048722	NA
CG32640	318135	3.52	4.18	1.57	Up	0.026336	NA
Ast	42947	5.00	4.35	1.56	Down	0.041968	0.007838
Zasp52	36740	3.20	2.56	1.56	Down	0.048659	NA
membrin	38614	3.33	2.69	1.56	Down	0.029299	NA
CG13800	38305	4.81	4.17	1.56	Down	0.040561	0.351735
CR30009	39517	3.23	2.59	1.56	Down	0.047788	NA
CG7366	41710	3.84	4.48	1.55	Up	0.017267	NA
v(2)k05816	31502	4.48	3.85	1.55	Down	0.017021	NA
ventrally-expressed-protein-D	117331	2.47	3.11	1.55	Up	0.014476	NA
Hen1	36301	1.64	2.27	1.55	Up	0.010345	NA
CG9932	32469	4.87	4.24	1.55	Down	0.044803	0.118082
CG8950	43751	7.70	7.08	1.54	Down	0.001276	0.000272

Pp2A-29B	32028	4.51	3.88	1.54	Down	0.049109	0.182235
RpL40	37227	3.17	2.55	1.54	Down	0.041095	NA
CG7264	50001	2.14	2.76	1.54	Up	0.007733	NA
CG1273	38522	2.53	1.91	1.53	Down	0.036546	NA
calypso	41384	3.20	2.58	1.53	Down	0.044129	NA
Mis12	38762	3.13	2.52	1.53	Down	0.047486	NA
tacc	41502	2.71	3.32	1.53	Up	0.007768	NA
CG16970	34740	6.21	5.59	1.53	Down	0.030011	0.00666
CG13575	38352	5.52	4.91	1.53	Down	0.020993	NA
Sin3A	39317	5.18	5.79	1.53	Up	0.025495	0.000283
Mitf	3885647	4.27	4.88	1.53	Up	0.006326	NA
cenG1A	48336	3.79	4.40	1.52	Up	0.048313	0.070744
Hr51	36702	3.13	2.53	1.52	Down	0.023511	NA
CG7777	36237	4.01	3.40	1.52	Down	0.001274	0.040622
imd	38957	3.85	3.25	1.52	Down	0.001311	NA
CG15828	44002	3.00	3.60	1.52	Up	0.017302	NA
CG8086	34131	5.08	4.48	1.51	Down	0.049406	0.186158
Rpp30	31237	3.17	2.58	1.51	Down	0.035714	NA
CR14578	40059	6.08	6.67	1.51	Up	0.017083	0.00013
tim	32401	4.40	3.80	1.51	Down	0.035079	0.142297
CG9510	34681	4.78	4.19	1.51	Down	0.010824	0.098217
CG14593	35535	3.45	2.86	1.51	Down	0.003644	NA
Ret	32930	7.54	6.96	1.5	Down	0.026735	0.003947
CG1814	35984	4.86	4.27	1.5	Down	0.033477	0.405891
CG31344	31344	4.68	4.09	1.5	Down	0.010613	NA
Indy	40049	5.74	5.15	1.5	Down	0.015006	NA
Idgf3	34981	6.97	6.39	1.5	Down	9.21E-05	0.001386
CG33213	338395	4.37	3.79	1.5	Down	0.031199	0.290773
A16	32468	6.30	5.72	1.5	Down	0.026148	0.026147
CG5924	34307	3.30	3.88	1.49	Up	0.045936	0.405891
CG8475	40430	3.88	3.31	1.49	Down	0.049432	0.262212
Tango1	42835	4.05	4.62	1.48	Up	0.02415	NA
CG10341	31429	3.56	2.99	1.48	Down	0.011545	NA
CG1969	43530	4.00	3.44	1.48	Down	0.009633	NA
CG1265	38517	2.60	3.17	1.48	Up	0.01375	NA
CG17036	34679	3.39	3.95	1.48	Up	0.018873	0.384406
CaBP1	41616	6.59	6.03	1.47	Down	0.020286	NA
GABA-B-R1	34878	4.43	3.87	1.47	Down	0.033725	NA
CG9948	41626	4.17	3.61	1.47	Down	0.040022	NA
JIL-1	43551	5.16	5.71	1.46	Up	0.002952	0.011397
CR32385	42503	4.13	4.67	1.46	Up	0.006713	0.170104
mdy	32610	4.13	3.59	1.46	Down	0.010821	0.331745
daw	33474	5.73	5.19	1.46	Down	0.001721	0.102708

CG33339	33339	4.54	4.01	1.45	Down	0.000285	NA
snmRNA:128	3771829	3.35	2.82	1.45	Down	0.037279	NA
santa-maria	41317	4.46	3.93	1.44	Down	0.020717	0.234793
CG6201	42339	4.19	4.72	1.44	Up	0.033606	0.125953
CG34370	50405	3.93	3.41	1.43	Down	0.015952	NA
CaBP1	39962	7.99	7.47	1.43	Down	0.048012	0.004571
dpp	33432	3.99	3.47	1.43	Down	0.044814	0.347364
CG12187	38337	5.29	4.78	1.43	Down	0.011323	0.509133
CG13330	36501	6.46	5.95	1.43	Down	0.002787	NA
CG9319	35337	4.06	4.58	1.43	Up	0.04172	NA
CG32500	2768879	3.81	3.30	1.43	Down	0.001814	NA
CG32857	318252	3.65	3.14	1.42	Down	0.01842	NA
CG31729	31729	5.50	4.99	1.42	Down	0.033985	0.254171
Catsup	37708	4.92	5.43	1.42	Up	0.007056	0.026985
CG3407	33606	3.81	4.31	1.42	Up	0.043826	0.267378
CG13901	45706	2.92	2.42	1.41	Down	0.041665	NA
tim	38501	4.82	4.32	1.41	Down	0.028118	NA
CG5660	39023	3.87	3.37	1.41	Down	0.038606	NA
ush	42440	3.76	4.25	1.41	Up	0.046118	0.43007
CG31954	31543	6.80	6.31	1.4	Down	0.044103	NA
botv	38793	7.02	6.53	1.4	Down	0.036859	NA
Ppcdc	37882	5.27	4.78	1.4	Down	0.006221	NA
Pmi	3772100	5.06	4.57	1.4	Down	0.002972	NA
CG13868	37302	7.52	7.03	1.4	Down	0.009833	0.026985
BicC	41473	4.43	3.95	1.4	Down	0.007827	NA
aret	34648	6.21	5.73	1.39	Down	0.010663	NA
FucTC	3355171	3.58	3.11	1.39	Down	0.047457	NA
Gmd	42351	3.70	4.18	1.39	Up	0.02754	NA
Socs44A	35786	5.44	4.96	1.39	Down	0.010176	0.378194
fusl	33765	4.13	3.66	1.39	Down	0.040061	NA
mir-184	42925	5.25	4.77	1.39	Down	0.044097	NA
CG10543	41340	6.04	5.57	1.38	Down	0.044889	NA
CG7120	38954	4.88	4.41	1.38	Down	0.018226	0.427281
CG6043	34732	3.88	3.41	1.38	Down	0.009849	NA
prom	42310	7.14	6.67	1.38	Down	0.010623	NA
CG9314	40339	5.61	5.15	1.38	Down	0.030702	0.389559
CG9130	39611	3.82	4.28	1.38	Up	0.028969	0.267378
osp	34850	6.09	5.63	1.37	Down	0.01311	0.099178
Jheh2	41355	3.82	4.27	1.37	Up	0.018599	0.194671
EndoG	43293	2.90	3.36	1.37	Up	0.026149	NA
mbl	36945	7.63	7.18	1.37	Down	0.017529	0.020587
CG3376	37884	6.27	5.82	1.37	Down	0.030479	0.12581
Fuca	3772574	5.49	5.04	1.37	Down	0.047797	0.40095

CG11714	3772566	5.49	5.04	1.37	Down	0.047797	NA
Hydr1	35930	4.93	4.48	1.37	Down	0.041762	NA
Pkc53E	40124	5.67	6.11	1.36	Up	0.000282	0.110654
Cf2	33692	6.39	5.94	1.36	Down	0.020281	0.419474
CG1675	36022	4.33	3.88	1.36	Down	0.013017	NA
Mmp2	38523	7.15	6.70	1.36	Down	0.030197	0.125953
CG42266	42266	4.46	4.90	1.36	Up	0.00995	0.188392
CG8486	34112	6.93	6.49	1.36	Down	0.049041	0.2488
CG4133	36570	3.70	4.14	1.36	Up	0.005257	0.527191
FucTD	44126	1.92	2.36	1.36	Up	0.040167	NA
PGRP-LD	3771920	4.22	3.78	1.35	Down	0.00332	NA
CG31688	40999	5.60	5.16	1.35	Down	0.041068	0.317133
CG42750	42750	4.70	4.27	1.35	Down	0.010674	0.429966
CG11912	40524	5.71	5.28	1.35	Down	0.006572	0.288112
hig	35949	7.60	7.17	1.34	Down	0.013471	0.113908
rok	43916	7.02	6.60	1.34	Down	0.022776	0.18697
CG5446	34655	5.39	5.81	1.34	Up	0.000201	0.444587
Ugt36Bc	32606	4.90	4.48	1.34	Down	0.045213	0.311492
CG31251	318645	3.34	3.77	1.34	Up	0.043324	NA
CG17742	38787	6.04	5.62	1.34	Down	0.014831	0.289351
ck	36656	5.26	4.84	1.34	Down	0.04025	NA
CG11180	37330	4.69	5.11	1.34	Up	0.049615	0.339929
dpr3	3346208	4.29	3.87	1.34	Down	0.010102	NA
CG15643	32489	3.61	3.19	1.33	Down	0.015692	0.542998
cpx	32490	5.39	4.97	1.33	Down	0.002076	0.431609
Rx	41835	4.60	4.18	1.33	Down	0.025915	NA
NHP2	44005	4.82	5.23	1.33	Up	0.047945	NA
rig	32233	6.35	5.94	1.33	Down	0.002674	0.408399
blue	39939	5.06	4.64	1.33	Down	0.035186	NA
Shawl	32113	4.54	4.95	1.33	Up	0.043883	0.102578
CG13784	34003	6.18	5.77	1.33	Down	0.022977	0.223418
CG8678	35396	4.87	4.46	1.33	Down	0.013731	0.529443
GC	38194	5.63	6.03	1.33	Up	0.019519	0.312127
mre11	34565	4.97	4.57	1.33	Down	0.04395	0.376261
CG1695	33046	5.84	5.44	1.32	Down	0.023696	0.165713
DNApol-gamma35	3772064	5.32	5.72	1.32	Up	0.045621	NA
CG33649	3772218	5.32	5.72	1.32	Up	0.045621	NA
tweek	53586	5.52	5.12	1.32	Down	0.005346	0.529944
tutl	38657	3.50	3.90	1.32	Up	0.045415	NA
CG9008	34779	3.79	3.39	1.32	Down	0.005822	NA
snmRNA:357	3772300	7.13	7.53	1.32	Up	0.03277	NA
CG17082	3355133	5.43	5.83	1.32	Up	0.048565	0.311492
CG34381	31743	3.11	2.71	1.32	Down	0.041236	NA

hk	41578	3.25	2.85	1.32	Down	0.041486	NA
Skeletor	3771796	3.67	3.27	1.32	Down	0.033858	NA
Skeletor	3772559	3.67	3.27	1.32	Down	0.033858	NA
CG31714	34341	4.98	4.58	1.31	Down	0.047241	NA
Oatp26F	33927	3.66	3.27	1.31	Down	0.023559	NA
CG7741	36208	4.20	4.60	1.31	Up	0.046852	NA
rdo	35077	4.57	4.18	1.31	Down	0.037158	NA
mfas	41455	8.19	7.80	1.31	Down	0.0272	0.081752
Prosalpha1	45780	6.51	6.12	1.31	Down	0.014568	NA
Rab14	44870	5.19	5.57	1.31	Up	0.000291	0.104046
CG10664	38885	5.02	4.63	1.31	Down	0.036964	0.467953
CG6520	36967	5.20	5.58	1.31	Up	0.046791	0.161705
ine	33659	4.76	4.38	1.3	Down	0.024958	NA
d	31610	5.06	4.67	1.3	Down	0.003864	NA
CG10249	36668	5.94	5.56	1.3	Down	0.035681	NA
rdgBbeta	39582	3.14	2.76	1.3	Down	0.042324	NA
srpk79D	41113	3.69	3.31	1.3	Down	0.033283	0.467325
CG2976	33682	5.13	4.75	1.3	Down	0.003785	0.295955
CG3975	38629	7.62	7.99	1.3	Up	0.01812	NA
CG34353	5740590	5.77	5.40	1.3	Down	0.047338	NA
CG10431	35157	4.24	4.62	1.3	Up	0.040184	NA
TepII	45970	4.62	4.25	1.29	Down	0.005991	NA
CG34380	40323	5.20	4.83	1.29	Down	0.028805	NA
Rgk2	37132	4.40	4.03	1.29	Down	0.015394	NA
CG32815	318224	6.06	5.69	1.29	Down	0.008855	0.439892
CG13993	43682	4.78	4.41	1.29	Down	0.017918	0.434994
snRNA:U2:38ABa	32882	4.58	4.21	1.29	Down	0.017974	NA
CG14590	48903	4.53	4.90	1.29	Up	0.01625	NA
Smc5	326215	5.30	5.67	1.29	Up	0.049318	NA
ics	38735	5.08	5.45	1.29	Up	0.037363	0.496872
CG3021	53546	4.66	4.30	1.29	Down	0.005782	NA
CG34371	32922	4.53	4.16	1.29	Down	0.006553	NA
dpr9	2768670	5.76	5.39	1.29	Down	0.040939	NA
CG32039	317834	3.16	3.53	1.29	Up	0.040899	NA
CG11455	39331	4.47	4.83	1.29	Up	0.017375	0.509969
CG10338	35793	4.98	5.34	1.28	Up	0.013648	NA
CG15270	34886	6.12	5.76	1.28	Down	0.015258	0.191724
d4	42505	3.21	3.57	1.28	Up	0.029713	NA
Lrr47	34449	4.12	3.76	1.28	Down	0.048854	NA
CG31772	33601	6.31	5.95	1.28	Down	0.047919	0.31614
CG33631	31934	3.06	3.41	1.28	Up	0.01364	NA
dpr12	50320	5.63	5.27	1.28	Down	0.00499	NA
CG31953	31953	7.15	6.79	1.28	Down	0.002421	NA

CG33923	38930	3.29	3.64	1.27	Up	0.018837	NA
CG17912	35004	7.30	6.95	1.27	Down	0.031361	0.431609
CG4945	38845	6.54	6.19	1.27	Down	0.03219	NA
Alg10	32076	4.88	4.53	1.27	Down	0.018449	NA
sec71	34785	6.66	6.31	1.27	Down	0.039804	NA
Ir41a	38817	6.34	5.99	1.27	Down	0.00139	NA
Ca-alpha1D	42045	4.56	4.90	1.27	Up	0.010761	NA
CG3570	37934	3.73	4.08	1.27	Up	0.019854	NA
CG12214	36072	5.72	5.38	1.27	Down	0.038534	0.466225
bft	34639	5.92	6.27	1.27	Up	0.025019	0.267378
fau	41994	4.00	3.66	1.27	Down	0.0243	NA
CG6614	38752	7.13	6.79	1.27	Down	0.005838	0.428626
Pde1c	34594	6.48	6.13	1.27	Down	0.009011	0.392587
CG15630	31970	5.00	4.66	1.27	Down	0.003348	0.503575
Aldh-III	41571	5.06	4.72	1.27	Down	0.023438	NA
nxf2	41644	5.80	5.46	1.26	Down	0.008273	NA
CG30460	36924	6.03	5.70	1.26	Down	0.029185	NA
ACXD	33110	6.88	6.54	1.26	Down	0.010771	0.383025
CG31244	31244	5.16	4.82	1.26	Down	0.049993	NA
CG1648	40794	6.19	5.85	1.26	Down	0.031008	NA
CG8617	36594	5.51	5.17	1.26	Down	0.021017	NA
CG42389	34987	7.42	7.09	1.26	Down	0.039772	0.35522
Neu3	3772109	6.75	7.08	1.26	Up	0.021074	NA
CG34393	33534	3.74	3.41	1.26	Down	0.038898	NA
CG9510	3771738	3.66	3.33	1.26	Down	0.048218	NA
CG9515	3771965	3.66	3.33	1.26	Down	0.048218	NA
CG10880	42726	5.46	5.14	1.26	Down	0.010068	0.33168
yellow-c	34879	4.11	4.44	1.25	Up	0.016643	NA
Acp53Ea	37382	6.34	6.01	1.25	Down	0.014166	0.472786
CG33123	53472	7.55	7.23	1.25	Down	0.000752	0.509133
zuc	246582	6.60	6.28	1.25	Down	0.014265	NA
CG33116	32625	7.43	7.11	1.25	Down	0.013976	0.302478
CG10492	35177	6.96	6.65	1.25	Down	0.038244	0.54266
Ip259	41376	5.23	4.91	1.25	Down	0.002889	NA
ush	33225	5.58	5.26	1.25	Down	0.045819	0.529944
Atf-2	37978	7.61	7.30	1.24	Down	0.016068	0.43007
Fmrf	36147	7.26	6.94	1.24	Down	0.033813	0.376025
sr	42402	5.94	5.63	1.24	Down	0.046221	NA
CG10912	40265	4.72	4.41	1.24	Down	0.012446	0.451935
l(2)tid	43506	5.79	5.47	1.24	Down	0.042428	0.431609
Slbp	43448	5.33	5.02	1.24	Down	0.011306	NA
Acp26Aa	37218	5.50	5.19	1.24	Down	0.020264	NA
mRpL17	43278	4.39	4.70	1.24	Up	0.049459	NA

Pkg21D	40147	5.11	4.79	1.24	Down	0.010338	NA
CG17273	32788	6.01	5.70	1.24	Down	0.009054	NA
Gef26	31798	7.04	6.73	1.24	Down	0.024021	0.451935
wb	32970	7.38	7.07	1.24	Down	0.029728	NA
CG31778	31565	6.97	6.67	1.24	Down	0.015478	NA
CG31646	40948	5.77	5.46	1.24	Down	0.010022	NA
CG1317	38300	7.13	6.83	1.24	Down	0.004766	NA
CG9641	40659	5.29	4.99	1.23	Down	0.001328	NA
Spred	36643	7.92	7.62	1.23	Down	0.022701	0.355773
CG33145	41403	5.60	5.30	1.23	Down	0.024149	0.296358
ACXB	33108	6.91	6.61	1.23	Down	0.009807	NA
dp	31964	7.48	7.18	1.23	Down	0.032315	NA
Adh	36580	5.94	6.24	1.23	Up	0.026749	0.392587
CG3558	48421	6.80	6.50	1.23	Down	0.040649	NA
Ptpmeg	41636	6.53	6.23	1.23	Down	0.025797	0.455822
lectin-22C	42295	6.14	5.84	1.23	Down	0.01827	NA
Hmx	42110	5.93	5.64	1.23	Down	0.004251	NA
CG17660	33347	6.48	6.78	1.23	Up	0.004263	0.244993
CG33932	338392	6.18	5.88	1.23	Down	0.002773	NA
Rpp20	3772007	6.18	5.88	1.23	Down	0.002773	NA
CG1516	39627	4.92	4.62	1.23	Down	0.042946	NA
CG4577	33291	8.17	7.87	1.23	Down	0.02946	0.472786
Zasp52	38978	9.89	9.60	1.23	Down	0.041866	NA
CG42303	42303	3.70	3.41	1.22	Down	0.040607	NA
FK506-bp2	41441	6.47	6.18	1.22	Down	0.015847	NA
hipk	39732	7.12	6.83	1.22	Down	0.032088	0.320696
CG9330	40131	6.59	6.30	1.22	Down	0.049176	NA
CG5028	118436	6.53	6.25	1.22	Down	0.021803	NA
crol	45931	6.51	6.22	1.22	Down	0.013911	NA
Sos	34912	5.49	5.21	1.22	Down	0.011383	NA
svp	41491	6.25	5.97	1.22	Down	0.019667	NA
Ire1	42358	6.23	5.95	1.22	Down	0.049362	NA
CG1667	36016	3.27	3.55	1.22	Up	0.025731	NA
CG1134	38472	3.69	3.41	1.21	Down	0.025845	NA
RYBP	37601	6.36	6.08	1.21	Down	0.010602	NA
Hmgs	44154	6.23	5.96	1.21	Down	0.030339	NA
Iap2	32373	7.56	7.28	1.21	Down	0.013725	NA
CG6498	39686	7.84	7.56	1.21	Down	0.043239	NA
oys	37306	8.77	8.50	1.21	Down	0.049926	NA
Syt1	38716	5.60	5.32	1.21	Down	0.003163	NA
dos	32135	7.04	6.76	1.21	Down	0.024332	NA
Hrs	33565	6.75	6.48	1.21	Down	0.015042	NA
PHDP	32684	7.10	6.83	1.21	Down	0.031428	0.515358

CG13295	38692	3.53	3.80	1.21	Up	0.038774	NA
mir-2b-1	42175	6.23	5.95	1.21	Down	0.013821	NA
tau	326116	6.96	6.69	1.21	Down	0.009074	NA
CG5287	34711	3.43	3.71	1.21	Up	0.036686	NA
Nnf1b	31658	6.87	6.60	1.21	Down	0.027361	NA
Catsup	37781	7.11	7.38	1.21	Up	0.028224	NA
CG2955	38195	10.79	10.52	1.21	Down	0.003165	0.297552
pelo	34286	5.63	5.36	1.21	Down	0.02782	NA
Tom7	35899	5.32	5.59	1.21	Up	0.037214	NA
Ced-12	34633	6.11	6.38	1.21	Up	0.046891	NA
jing	37954	7.14	6.87	1.21	Down	0.017555	NA
Pka-C1	34284	8.74	8.47	1.2	Down	0.034984	NA
CG42307	7354423	5.62	5.35	1.2	Down	0.005506	NA
FKBP59	38809	5.62	5.35	1.2	Down	0.005506	NA
BtbVII	38376	7.84	7.58	1.2	Down	0.013073	0.51454
ade3	31628	5.94	5.67	1.2	Down	0.049187	NA
Trp1	39524	5.87	5.60	1.2	Down	0.005581	NA
Acer	34189	4.90	4.64	1.2	Down	0.043338	NA
shep	38605	9.40	9.14	1.2	Down	0.014671	0.522769
mir-12	33076	5.13	4.86	1.2	Down	0.02667	NA
CG3808	40074	4.55	4.29	1.2	Down	0.049816	NA
Sr-CI	38003	7.80	7.54	1.2	Down	0.000685	NA
CG42399	31225	5.98	5.72	1.2	Down	0.015245	NA
CG34452	40876	8.65	8.39	1.2	Down	0.025907	NA
alien	34225	6.10	6.36	1.2	Up	0.024126	NA
snRNA:U1:21D	31656	5.59	5.33	1.2	Down	0.044501	NA
Side	41869	4.42	4.68	1.2	Up	0.019455	0.278074
vir-1	32426	6.54	6.28	1.2	Down	0.012518	NA
spen	42345	7.25	6.99	1.2	Down	0.030805	NA
esc	35098	10.30	10.04	1.2	Down	0.03717	0.53406
CG4341	33276	7.93	7.67	1.2	Down	0.021041	0.431609
CG42271	2768892	6.32	6.06	1.2	Down	0.006285	NA
CG10866	38461	2.20	2.46	1.2	Up	0.049036	NA
aop	45467	7.58	7.33	1.19	Down	0.049269	NA
Atg1	42504	6.76	7.01	1.19	Up	0.041476	0.537672
Ntf-2r	35101	3.92	4.18	1.19	Up	0.010746	NA
CG3662	42179	5.28	5.54	1.19	Up	0.028375	0.165713
nol	32077	8.01	7.75	1.19	Down	0.027794	NA
app	39399	6.50	6.25	1.19	Down	0.018482	NA
CG1600	40687	10.58	10.32	1.19	Down	0.00691	NA
CG5543	41247	8.02	7.76	1.19	Down	0.0087	0.507438
wb	35236	5.24	5.49	1.19	Up	0.011216	NA
ns4	35338	5.78	6.03	1.19	Up	0.033897	NA

pr	41592	6.69	6.44	1.19	Down	0.048718	NA
CG2269	36056	9.26	9.01	1.19	Down	0.015012	0.451935
CG30007	38756	10.64	10.40	1.19	Down	0.001514	NA
robo3	33314	7.93	7.69	1.19	Down	0.032163	NA
snoRNA:Psi28S-1180	44018	7.55	7.30	1.19	Down	0.012109	NA
betaggt-II	40972	7.05	7.29	1.19	Up	0.039923	0.434994
dao	34891	5.48	5.23	1.19	Down	0.018119	0.293868
CG34212	40704	7.06	7.31	1.19	Up	0.038359	0.305174
Su(var)2-HP2	42954	7.73	7.48	1.18	Down	0.025537	NA
CG42404	41842	6.86	6.62	1.18	Down	0.047398	NA
CG6209	45021	4.52	4.28	1.18	Down	0.01107	NA
A16	41120	9.56	9.32	1.18	Down	0.006984	NA
CG11658	39319	4.31	4.06	1.18	Down	0.021354	NA
Smg5	34804	7.85	7.61	1.18	Down	0.012911	NA
CG42748	32976	7.42	7.18	1.18	Down	0.009628	NA
Dyb	36362	7.02	6.79	1.18	Down	0.010156	NA
iPLA2-VIA	43728	5.32	5.56	1.18	Up	0.040924	NA
ia2	31795	7.27	7.04	1.18	Down	0.004803	NA
CG42561	42561	7.61	7.38	1.18	Down	0.013864	NA
CG13284	36375	6.43	6.20	1.18	Down	0.00585	NA
CG4502	39135	6.93	6.70	1.18	Down	0.047974	NA
CG11050	42634	5.46	5.69	1.17	Up	0.02701	0.529944
Alr	32991	4.49	4.26	1.17	Down	0.017587	NA
CG9527	38420	8.67	8.44	1.17	Down	0.016645	NA
syd	43905	6.89	6.66	1.17	Down	0.042469	NA
CG31919	40249	7.80	7.57	1.17	Down	0.015213	NA
RpL24	34754	10.36	10.13	1.17	Down	0.001901	NA
CG10260	31247	7.09	6.86	1.17	Down	0.046907	NA
clumsy	32933	5.97	5.74	1.17	Down	0.038029	NA
CG17377	46158	5.88	5.65	1.17	Down	0.001968	NA
Pi3K68D	42793	6.43	6.21	1.17	Down	0.037253	NA
CG32452	318035	4.82	4.60	1.17	Down	0.029157	NA
SoxN	44275	9.52	9.30	1.17	Down	0.023553	0.439892
CG11035	43434	6.52	6.29	1.17	Down	0.037376	NA
CG31352	261629	7.86	7.63	1.17	Down	0.005146	NA
CG8001	38224	5.85	5.63	1.17	Down	0.037784	NA
CG32396	43453	5.90	5.67	1.17	Down	0.012514	NA
CG3156	30994	3.97	3.75	1.17	Down	0.024241	NA
CG13995	33851	5.92	5.70	1.17	Down	0.024341	NA
CG7879	38184	5.84	6.06	1.17	Up	0.003682	0.307188
Fbxl4	32378	5.19	4.97	1.17	Down	0.040393	NA
ken	49713	6.53	6.31	1.17	Down	0.00303	NA
tRNA:K5:84ABb	31489	4.55	4.33	1.17	Down	0.045376	NA

Catsup	37750	4.15	3.93	1.17	Down	0.037343	NA
CG4707	37935	5.38	5.15	1.17	Down	0.034506	NA
CG31665	33361	7.53	7.30	1.17	Down	0.027805	NA
CG7686	36146	7.08	6.86	1.17	Down	0.020465	NA
lwr	31701	5.09	4.87	1.17	Down	0.045416	NA
CG13330	44014	8.14	7.92	1.17	Down	0.014044	NA
CG1600	35687	8.67	8.45	1.16	Down	0.04612	NA
GRHR	33132	7.55	7.33	1.16	Down	0.024127	NA
CG9098	33840	7.31	7.10	1.16	Down	0.032015	NA
Sfp33A2	42473	7.15	6.93	1.16	Down	0.005995	NA
CG31813	31813	6.41	6.20	1.16	Down	0.04682	NA
Hex-t1	32849	4.37	4.15	1.16	Down	0.047447	NA
edl	39588	3.40	3.62	1.16	Up	0.021335	NA
CG8814	41768	6.89	6.67	1.16	Down	0.027794	NA
Acp53Ea	37381	5.90	6.11	1.16	Up	0.042258	NA
CG17129	38080	2.09	1.87	1.16	Down	0.03852	NA
debcl	32430	4.42	4.20	1.16	Down	0.01702	NA
Su(Tpl)	32217	7.00	6.79	1.16	Down	0.016797	NA
CG31357	326135	6.22	6.01	1.16	Down	0.041794	NA
CG40045	3355079	6.64	6.85	1.16	Up	0.016158	0.480176
LSm7	53562	8.46	8.25	1.16	Down	0.043653	NA
CG13343	36539	5.37	5.58	1.16	Up	0.023846	NA
snoRNA:Psi28S-2263	34568	6.57	6.36	1.16	Down	0.007577	NA
nimC4	43277	7.09	6.88	1.16	Down	0.002066	NA
Acp53Ea	37384	6.31	6.10	1.16	Down	0.005883	NA
CG8441	36792	4.26	4.47	1.16	Up	0.032233	NA
CG15765	31513	7.46	7.25	1.16	Down	0.007461	NA
aru	31290	6.04	5.83	1.16	Down	0.037909	NA
psq	36118	8.46	8.25	1.16	Down	0.009171	NA
E2f2	31523	6.42	6.21	1.16	Down	0.029643	NA
CG10253	36669	6.39	6.18	1.16	Down	0.00782	NA
Nhe2	42958	8.78	8.57	1.16	Down	0.027935	NA
CG7149	40654	9.34	9.13	1.16	Down	0.033057	NA
arr	44279	6.96	6.76	1.15	Down	0.049788	NA
Rho1	40792	6.80	6.59	1.15	Down	0.007161	NA
CG31871	32278	6.51	6.31	1.15	Down	0.013947	NA
wah	41911	8.36	8.16	1.15	Down	0.033249	NA
CG3589	42169	8.52	8.31	1.15	Down	0.011013	NA
CG11377	37152	5.33	5.13	1.15	Down	0.021994	NA
rdo	40258	3.66	3.45	1.15	Down	0.00489	NA
Nup54	36360	5.95	5.75	1.15	Down	0.044708	NA
SCAR	34519	7.30	7.10	1.15	Down	0.019055	NA
CG1753	33081	6.22	6.02	1.15	Down	0.025507	NA

Dscam	31050	6.26	6.06	1.15	Down	0.024422	NA
CG4970	32953	10.32	10.12	1.15	Down	0.004274	NA
Cpsf160	41365	6.54	6.34	1.15	Down	0.038497	NA
Gpdh	32379	7.00	6.80	1.15	Down	0.02404	NA
CG13705	48481	5.62	5.82	1.15	Up	0.013055	NA
CG9531	32935	7.34	7.15	1.15	Down	0.015081	NA
CG12042	43901	7.76	7.56	1.15	Down	0.013469	NA
flfl	42267	8.07	7.88	1.15	Down	0.000288	NA
Acp29AB	32561	8.01	7.81	1.14	Down	0.014684	NA
CG13097	34184	5.69	5.50	1.14	Down	0.021774	NA
mdy	32611	2.89	3.09	1.14	Up	0.012586	NA
drl	41343	9.60	9.40	1.14	Down	0.043145	NA
Sec61alpha	42993	4.71	4.91	1.14	Up	0.047558	NA
CG4806	37948	5.45	5.64	1.14	Up	0.037286	NA
aop	40431	8.78	8.59	1.14	Down	0.022144	NA
crol	34592	9.92	9.72	1.14	Down	0.00821	NA
dikar	43576	7.40	7.21	1.14	Down	0.035109	NA
Fsn	33133	4.65	4.83	1.14	Up	0.044879	NA
snoRNA:Me28S-G3255b	40461	7.65	7.46	1.14	Down	0.033262	NA
Catsup	37761	5.39	5.21	1.14	Down	0.035649	NA
loqs	34751	7.57	7.39	1.13	Down	0.042821	NA
trsn	36110	5.00	5.18	1.13	Up	0.049492	NA
lbk	36788	6.73	6.55	1.13	Down	0.012045	NA
CG33969	41443	8.19	8.01	1.13	Down	0.031234	NA
CG16812	34722	6.06	5.88	1.13	Down	0.030237	NA
RpL37A	44783	8.95	8.77	1.13	Down	0.033344	NA
RpL9	45928	8.10	7.92	1.13	Down	0.014404	NA
spir	32854	7.25	7.07	1.13	Down	0.041228	NA
fusl	38943	4.77	4.95	1.13	Up	0.023626	NA
AGO1	43353	6.19	6.02	1.13	Down	0.004653	NA
Jon25Bii	35758	7.17	7.00	1.13	Down	0.015264	NA
CG12194	33685	4.66	4.84	1.13	Up	0.04748	NA
Amy-d	32536	7.10	7.27	1.13	Up	0.024985	NA
Smox	31738	8.53	8.35	1.13	Down	0.014364	NA
CG33013	41398	7.64	7.47	1.13	Down	0.003063	NA
Catsup	37862	7.18	7.01	1.13	Down	0.0036	NA
PNUTS	33526	7.34	7.17	1.13	Down	0.006367	NA
CG42741	40139	4.31	4.48	1.13	Up	0.030533	NA
CG31814	318958	4.19	4.02	1.13	Down	0.034266	NA
CG9304	39688	5.40	5.23	1.13	Down	0.007543	NA
Pvf3	31629	6.23	6.06	1.13	Down	0.039342	NA
PRL-1	34952	7.70	7.87	1.13	Up	0.047632	NA

eIF-4a	33835	11.35	11.18	1.12	Down	0.007014	NA
CG32066	39218	7.05	6.88	1.12	Down	0.031178	NA
sca	36411	6.76	6.59	1.12	Down	0.03484	NA
tRNA:D2:69F	32125	6.54	6.38	1.12	Down	0.011039	NA
CG8833	39535	5.00	4.83	1.12	Down	0.005782	NA
oaf	33435	5.82	5.99	1.12	Up	0.045374	NA
I-2	39156	5.55	5.38	1.12	Down	0.024126	NA
gprs	36862	6.31	6.15	1.12	Down	0.044979	NA
Rca1	38700	7.15	6.99	1.12	Down	0.006886	NA
Poxn	41721	7.89	8.05	1.12	Up	0.037118	0.536973
Dscam	31044	4.55	4.39	1.12	Down	0.020911	NA
emb	34167	8.48	8.64	1.12	Up	0.030563	NA
mir-2c	42936	6.08	5.92	1.11	Down	0.010046	NA
stai	35627	5.26	5.42	1.11	Up	0.003471	NA
Thiolase	32690	7.75	7.60	1.11	Down	0.046568	NA
l(2)37Cb	42808	5.35	5.19	1.11	Down	0.025098	NA
Dek	47906	8.03	8.19	1.11	Up	0.036215	NA
aub	47384	5.77	5.62	1.11	Down	0.017217	NA
CG33464	2768843	8.14	7.99	1.11	Down	0.030078	NA
CG4669	41025	6.78	6.93	1.11	Up	0.040794	NA
tsh	35430	7.04	6.89	1.11	Down	0.023726	NA
CG31883	32616	6.58	6.43	1.11	Down	0.015304	NA
Ced-12	37464	6.94	6.79	1.11	Down	0.021769	NA
Aldh	32114	4.96	4.82	1.11	Down	0.036328	NA
snoRNA:Me28S-C3227b	40739	10.69	10.55	1.11	Down	0.001874	NA
Sir2	40562	6.51	6.36	1.11	Down	0.029894	NA
gpp	31151	10.46	10.32	1.11	Down	0.025169	NA
Opbp	246618	4.79	4.64	1.11	Down	0.012645	NA
CG12567	35509	7.19	7.05	1.11	Down	0.024839	NA
CG7971	38206	9.54	9.40	1.1	Down	0.045289	NA
Acp26Ab	37205	8.45	8.32	1.1	Down	0.04559	NA
Vha16-1	44307	8.71	8.57	1.1	Down	0.014843	NA
snRNA:U5:38ABa	32881	6.06	5.92	1.1	Down	0.04218	NA
mus201	41168	6.75	6.62	1.1	Down	0.043698	NA
tRNA:CR31602	31602	6.90	6.77	1.1	Down	0.018435	NA
snoRNA:Me18S-C1831	44000	8.73	8.60	1.1	Down	0.018979	NA
Gr64c	32256	7.23	7.10	1.09	Down	0.045078	NA
CG4983	40682	6.14	6.27	1.09	Up	0.03334	NA
CG3625	40155	7.94	7.82	1.09	Down	0.01687	NA
rl	41085	6.94	7.06	1.09	Up	0.00371	NA
CG42540	38562	6.33	6.20	1.09	Down	0.006548	NA

CG11866	38400	5.98	5.86	1.09	Down	0.014074	NA
CG10151	36639	6.76	6.64	1.09	Down	0.001704	NA
CG42342	7354466	7.86	7.74	1.09	Down	0.018692	NA
AGO2	39683	8.80	8.92	1.09	Up	0.000791	NA
CG42306	31581	7.09	6.98	1.08	Down	0.010146	NA
Amy-p	32524	5.75	5.64	1.08	Down	0.000328	NA
Egfr	39899	8.02	7.90	1.08	Down	0.025891	NA
His2B:CG17949	32627	7.57	7.69	1.08	Up	0.025126	NA
CG4230	33743	6.15	6.04	1.08	Down	0.003061	NA
Pkn	35950	6.98	6.87	1.08	Down	0.045645	NA
Catsup	37876	6.19	6.30	1.08	Up	0.016836	NA
bur	39099	7.49	7.38	1.08	Down	0.038098	NA
Trl	2768981	9.00	8.89	1.07	Down	0.038693	NA
cutlet	44637	4.27	4.37	1.07	Up	0.045913	NA
CG12907	44548	10.85	10.75	1.07	Down	0.021864	NA
alpha4GT1	38744	6.80	6.70	1.07	Down	0.045622	NA
CkIIalpha-i3	43130	6.60	6.50	1.07	Down	0.013431	NA
crc	32941	7.86	7.76	1.07	Down	0.001983	NA
lama	42265	7.94	7.85	1.07	Down	0.039376	NA
RpL19	37995	10.77	10.68	1.07	Down	0.039261	NA
Thor	32557	6.39	6.30	1.07	Down	0.00368	NA
snoRNA:Me28S-G2596	5740396	6.94	7.03	1.07	Up	0.04682	NA
snmRNA:184	3772671	6.94	7.03	1.07	Up	0.04682	NA
CG1371	36053	7.32	7.40	1.06	Up	0.04553	NA
CG17612	39935	6.37	6.29	1.05	Down	0.039049	NA
zf30C	34292	8.07	8.00	1.05	Down	0.048358	NA
cg	36571	8.51	8.46	1.04	Down	0.031872	NA
CG7248	40933	7.19	7.15	1.03	Down	0.011376	NA

Terms and abbreviations: Gene_Identifier, the flybase symbol of the corresponding differentially expressed gene, control_mean, the mean expression level of D42>LacZ, overexpression_mean, the mean expression level of D42>TBPH, Ratio, the fold-change of expression in D42>TBPH compared to D42>LacZ, Direction, the direction of the change, p_value, the genesifter p value (see methods for parameters), edgeR p value, the p value calculated in edgeR for the corresponding gene, also adjusted for multiple hypothesis testing (Benjamini & Hochberg, 1995). NA, the gene was not differentially expressed using edgeR analysis.

Table S3 List of 26 genes whose expression changed in both loss-of-function (LOF) and gain-of-function (GOF) genotypes and that contained putative TBPH binding sites.

Gene_Identifier	LOF	Ratio	p_value	GOF	Ratio	p_value
CG9812	+	2.96	0.00975	-	1.86	0.000189
Irc	+	2.67	0.010363	-	2.43	0.04415
wgn	+	1.67	0.001873	-	1.16	0.047447
plx	-	1.65	0.024184	+	1.19	0.038359
CG10249	+	1.56	0.030863	-	1.3	0.035681
CCHa2r	+	1.45	0.035987	-	1.51	0.003644
ltl	+	1.45	0.029087	-	1.27	0.03219
CG18210*	-	1.44	0.027657	-	1.55	0.044803
CG17211	-	1.39	0.008802	+	1.27	0.025019
stck	+	1.39	0.003959	-	1.35	0.041068
CG12065*	+	1.36	6.94E-05	-	1.24	0.024021
Tbh	+	1.3	0.009052	-	1.73	0.028432
CG3902	+	1.25	0.0101	+	1.51	0.017083
ush	+	1.22	0.015133	-	1.25	0.045819
CG13784	+	1.2	0.016125	-	1.33	0.022977
l(1)G0289	+	1.2	0.019807	-	1.23	0.032315
CG1578	+	1.2	0.030473	-	1.21	0.024332
CG9009	-	1.19	0.033497	-	1.2	0.012518
cora	+	1.19	0.00021	-	1.1	0.04559
CG4577	-	1.18	0.008905	-	1.23	0.02946
dome	+	1.18	0.018949	-	1.18	0.009628
Smox	+	1.16	0.044535	-	1.13	0.014364
CG13995	+	1.16	0.036236	-	1.17	0.024341
CG13928	-	1.16	0.011025	-	1.21	0.003165
Hmgs	+	1.15	0.022268	-	1.21	0.030339
CG34353	+	1.15	0.041968	-	1.3	0.047338

LOF Ratio, TBPH[G2] knockout expression relative to control A1 strain; GOF Ratio, D42-GAL4>UAS-TBPH expression relative to D42-GAL4>UAS-LacZ.

Table S4 Entrez gene identifiers for all genes used in Ontology analyses.

A: Entrez identifiers of DE in mutant , rescued or partially rescued, contains TBPH bind site (181 genes)

37981,3772221,42541,31001,37555,36340,39441,43662,33814,35554,36673,33135,32976,32080,36616,35731,43895,39106,31105,44736,42059,38490,39990,44936,37196,32469,318243,40403,33386,37546,36862,44544,43844,37788,37435,39485,37786,648
75,41059,39583,31964,37940,33225,37892,36930,39056,38273,31542,34554,42891,39017,37012,42581,31965,3772232,38976,38468,39284,33363,45040,37781,31408,32349,43563,47260,42015,41843,43265,47173,32009,41121,42477,37070,35181,33292,42
226,34826,32703,33807,40382,43579,39567,31767,38845,40170,38959,39530,43178,2768881,34093,37798,40835,32994,31201,31157,31795,32943,3346227,40633,32243,40666,37882,41605,33783,42429,35911,37401,34003,43800,37886,31842,40059,3204
5,48581,39311,38104,38195,35286,44317,36782,36383,31955,43950,44154,36896,44573,42049,40102,32321,37583,40229,40590,33291,31525,41360,40342,34002,45012,39054,31332,39860,42880,47767,3772312,36232,36048,35494,42747,39903,34114,325
79,40605,31130,31016,32135,33148,44497,39390,3346207,40330,38610,35620,40754,43765,32037,31718,35730,37119,31212,37661,36634,32083,32087,32273,33299,38484,40310,32822,32724,31980,39389,38255,42105,34969,42216,32447,34767,33953,43
560,40955,40999,35765,43676,38545,43756,37709,37652,43440,34170,43630,36030,326116,43223,33851,318721,31214,34835,42467,318916,41320,34639,34803,39543,42881,41266,32836,3346167,3346238,35507,32849,42547,41309,31873,32217,42707,3
6712,39132,53425,44679,33991,33765,36694,40912,45880,5740840,31451,44660,42702,36395,37755,117369,38017,39518,37161,41864,38895,40205,42066,35997,44258,39901,37132,31798,45380,40359,31309,31381,37310,37011,39049,37567,40959,5344
6,41674,37757,31839,30991,31612,47729,40305,41035,42527,3346150,36595,31402,3885573,31386,40549,42940,31588,3885598,31792,36681,50350,33928,5740318,32656,5740349,31591,34560,35737,50253,42832,38994,32576,33638,38434,32955,36248,
40879,44297,40922,36084,40335,42600,31687,42595,34181,32799,36513,32775,32098,37679,40056,31490,33283,41095,36415,44150,37205,43683,45922,34068,41677,36753,38059,42455,5740224,39090,39730,32337,35135,41840,33260,35535,39065,4313
6,37521,42530,45234,3771985,40876,32780,42411,40715,39954,32343,36496,40703,40704,37007,37419,32056,34993,41045,36603,43562,42196,38262,33495,36488,38222,32123,35444,35697,36121,38731,31441,32426,5740176,3771796,3772559,33014,31
666,31738,45875,36668,31229,34287,33556,35293,39873,39875,317913,53437,36526,38589,40180,35768,37129,42148,44359,48311,2768945,42836,318743,43197,41248,5740590,41062,5740551,4379891

Conserved with Sephton *et al*., (2011, 109 genes):

34731,42049,39567,40701,36371,41017,37619,4379858,47878,34015,40427,35525,36330,40205,37709,39748,32148,5740462,42879,31718,35284,41819,35094,35866,40424,43235,36740,38305,31502,36301,32469,43751,32028,34740,38352,39317,36237,34
131,40059,32401,35535,32930,35984,31344,40049,32468,42835,31429,41616,34878,43551,33339,42339,39962,33432,38337,31729,37882,41473,34648,33765,38954,34732,39611,34850,36945,37884,33692,38523,34112,44126,40999,35949,43916,32606,334
6208,32233,39939,34003,35396,38194,38657,34779,3355133,41578,3771796,3772559,34341,36208,35077,41455,31610,36668,38629,5740590,45970,37132,43682,48903,2768670,50320,31953,35004,38845,36072,34639,34594,31970,41644,36924,40794,365
94,34987,3772109,33534,3771738,3771965,35177,41376,33225,37978,36147,42402,31798,32970,31565,40948,36643,33108,31964,48421,42295,42110,33291,38978,42303,39732,45931,34912,41491,37601,44154,32373,39686,38716,32135,326116,31658,37
781,38195,34286,37954,34284,38376,31628,38605,31225,40876,32426,33276,2768892,45467,42504,32077,39399,41247,41592,36056,38756,33314,40704,41842,39319,34804,32976,36362,43728,31795,42561,43905,40249,31247,44275,261629,38224,43453,
30994,33851,37935,33361,44014,33132,42473,31813,32849,39588,32217,53562,43277,37384,31513,31290,36118,31523,42958,44279,40792,32278,41911,42169,37152,34519,33081,31050,32953,48481,43901,42267,32561,41343,40431,34592,40461,36788,4
1443,34722,45928,32854,43353,35758,32536,31738,41398,33526,318958,34952,36411,36862,38700,34167,42936,35627,32690,47906,35430,32616,32114,31151,35509,37205,44307,41168,31602,44000,32256,40155,41085,38562,7354466,31581,39899,3374
3,35950,39099,2768981,44548,38744,43130,32941,37995,36571

B: DE in overexpression, contains TBPH binding site (270 genes)

34731,42049,39567,40701,36371,41017,37619,4379858,47878,34015,40427,35525,36330,40205,37709,39748,32148,5740462,42879,31718,35284,41819,35094,35866,40424,43235,36740,38305,31502,36301,32469,43751,32028,34740,38352,39317,36237,34
131,40059,32401,35535,32930,35984,31344,40049,32468,42835,31429,41616,34878,43551,33339,42339,39962,33432,38337,31729,37882,41473,34648,33765,38954,34732,39611,34850,36945,37884,33692,38523,34112,44126,40999,35949,43916,32606,334
6208,32233,39939,34003,35396,38194,38657,34779,3355133,41578,3771796,3772559,34341,36208,35077,41455,31610,36668,38629,5740590,45970,37132,43682,48903,2768670,50320,31953,35004,38845,36072,34639,34594,31970,41644,36924,40794,365
94,34987,3772109,33534,3771738,3771965,35177,41376,33225,37978,36147,42402,31798,32970,31565,40948,36643,33108,31964,48421,42295,42110,33291,38978,42303,39732,45931,34912,41491,37601,44154,32373,39686,38716,32135,326116,31658,37
781,38195,34286,37954,34284,38376,31628,38605,31225,40876,32426,33276,2768892,45467,42504,32077,39399,41247,41592,36056,38756,33314,40704,41842,39319,34804,32976,36362,43728,31795,42561,43905,40249,31247,44275,261629,38224,43453,
30994,33851,37935,33361,44014,33132,42473,31813,32849,39588,32217,53562,43277,37384,31513,31290,36118,31523,42958,44279,40792,32278,41911,42169,37152,34519,33081,31050,32953,48481,43901,42267,32561,41343,40431,34592,40461,36788,4
1443,34722,45928,32854,43353,35758,32536,31738,41398,33526,318958,34952,36411,36862,38700,34167,42936,35627,32690,47906,35430,32616,32114,31151,35509,37205,44307,41168,31602,44000,32256,40155,41085,38562,7354466,31581,39899,3374
3,35950,39099,2768981,44548,38744,43130,32941,37995,36571

Conserved (85 genes):

40424,38352,32930,35984,42835,34878,39962,34850,36945,33692,38523,35949,43916,32233,38194,36208,5740590,48903,35004,34594,3772109,37978,32970,36643,31964,48421,42303,39732,45931,34912,41491,44154,39686,326116,37781,37954,34284,3
1628,2768892,42504,41247,38756,39319,43728,42561,43905,40249,31247,261629,38224,30994,44014,42473,39588,32217,43277,31290,44279,40792,32278,31050,43901,32561,34592,36788,45928,32854,35758,31738,41398,34167,42936,32616,31151,3550
9,37205,31602,32256,41085,31581,39899,35950,43130,37995,36571

C: Controls (7897 genes)

45524,35351,35946,42257,41259,39626,317923,31503,33274,38015,32419,37302,43671,47878,39529,40916,31162,317915,32034,40020,3771872,3772576,31674,34191,34383,38495,38818,39096,43820,45880,33292,37644,33635,40963,40268,37991,36348,
38638,318129,33324,37131,43227,38470,35211,43126,32778,36595,36838,33659,42787,41403,41031,318098,42339,37015,43593,36532,38473,35911,39795,37941,37590,43184,36806,33749,34894,36615,31641,38180,318135,41339,43177,43067,41036,320
36,41640,41368,43413,38432,246458,246608,50442,41160,37929,41155,41520,32610,34627,39333,42002,40345,43183,39075,33474,33874,32199,38058,37128,32327,34317,33569,318956,261621,32592,39232,31358,32401,31923,34037,39020,32399,37469,
36596,34679,43440,37004,37709,31408,43136,41831,5740766,326156,34127,36865,39582,35913,5740276,38741,32299,34819,43867,41297,32329,32148,45879,31591,42788,5740455,34565,318136,37614,4379858,318160,37295,35047,42426,261611,36573,
35380,3772574,3772566,40205,32035,37458,42581,40004,35735,36958,44887,42970,42432,31898,246587,3772393,3772088,33839,37182,35708,41202,326170,36740,40266,34038,38583,37534,33349,43583,36011,31340,34732,36016,32154,37229,39740,36
244,41887,38484,38501,43895,42530,45326,50350,32285,7354475,50001,35886,33657,38434,261626,39056,33934,37619,317953,38296,38297,33432,41513,39752,36601,42049,37435,40088,32087,33170,39748,43243,249663,39165,317907,40612,32353,50
044,31111,43275,31650,35745,31656,39071,35013,37949,33200,42981,33727,261623,42930,5740424,39954,35989,36924,40666,45396,39543,42455,3772646,3771806,36243,32488,326116,5740275,3354987,41308,38507,44013,36634,34786,36586,39055,31
8825,317857,35875,36849,50466,3346150,317852,38719,37940,246442,44058,34673,45875,35090,33641,38410,39264,37575,36501,7354376,7354377,7354378,7354381,31103,34322,40598,37545,40071,40918,39048,43530,35746,41518,33726,34260,33534,
38183,41767,317877,43551,41967,38972,33718,39049,39991,35513,41134,42893,37227,35417,39077,37748,41851,35104,318799,250710,34827,31099,41452,42697,43756,326264,42940,36076,35940,32283,246651,41144,38942,40051,39396,3355069,57401
95,42361,32694,33386,31417,59239,34117,39567,37106,38994,36957,38663,44098,34639,36081,41919,41221,35396,40083,34289,41033,40333,3772042,34611,37083,32680,37399,36292,32821,37166,40450,31718,41473,39231,34176,45780,31094,34351,41
252,43043,5740596,38443,34878,37887,40897,39517,39085,42335,32326,37294,40524,31136,41605,318631,38751,38813,39422,36668,246386,37560,41222,34530,32426,30972,31858,40492,41248,32093,38954,37822,31251,38610,44910,41070,38290,3606
0,36528,39237,38517,35264,33695,31402,43429,42676,35061,32672,41238,41112,35231,42560,36550,35622,40287,31743,33047,246573,41982,35687,5740325,31212,42242,41381,37219,42683,42860,39317,33540,42723,38104,37147,36009,37548,36753,45
588,39106,32558,38315,40608,59261,41044,32874,36965,42701,38472,44010,37663,326141,37137,34181,41821,34067,32469,246595,41278,40732,318251,31497,46391,34477,2768870,38518,36396,40102,43582,42216,318573,5740503,40713,31385,34623,
46386,34237,41894,40059,42832,40279,49090,40085,32724,43748,36588,41136,3346141,36651,40922,32072,31021,31198,2768992,3346208,246620,42801,41469,40816,35126,31391,42294,4379889,34064,42523,34087,3885628,2768998,246514,44297,404
91,117366,33613,32767,38390,42576,43014,38869,33022,2768668,42280,31055,318992,43825,43318,36368,34023,38889,326109,318203,41746,140439,32296,31695,31461,43414,40579,34645,318930,40001,38305,42890,39138,35180,32053,49077,246420,
36427,40031,2768965,42078,40575,45706,33354,35284,37223,33812,4379917,32476,43707,38252,50381,246533,38235,34152,318260,36776,47253,42783,40180,35221,46085,38454,37999,32665,35125,44057,7354414,50395,35153,32098,35620,43805,3591
6,318252,3355048,3772540,36862,41148,42527,38364,34652,40156,37376,40721,32064,40373,34952,31526,34792,35656,31843,59178,34528,42134,37641,41394,34106,42851,40486,34981,50402,50149,246511,37028,31480,32980,32354,31606,40153,4282
9,37697,42762,246582,34440,32620,34598,41191,33931,35554,34039,42477,35291,31326,42356,40422,43104,31729,38906,3346202,41742,42390,38337,38502,39854,33953,32272,53558,41626,3346192,46032,33530,35984,32447,42295,34961,42702,43324,
35003,37025,36017,38535,39139,31949,31857,39316,39550,36379,2768718,31186,42061,32549,37134,2769000,31777,37567,38012,35271,34490,32748,35704,39389,43514,40703,39871,3355016,31550,35309,36392,37968,31189,36284,37648,35238,43506,
43608,37445,53446,30988,42045,34090,39504,32251,41863,43326,31509,34184,39262,43002,39383,3772691,3771923,41157,37453,42929,31070,33284,48373,39306,32504,37985,38731,41017,34485,36248,41726,39409,31520,31935,5740349,59163,32485,
318031,39461,32626,34577,41758,34505,31702,7354433,31761,34434,30983,39238,33308,33600,34839,41644,5740879,5740394,34625,33347,33705,318224,40429,41820,43577,2769004,35353,3885598,31169,3346207,37342,36712,41450,37783,33476,409
31,41771,38957,37191,41937,40619,33175,32850,32079,44831,37944,36757,44858,3346227,43256,32135,5740347,44258,42620,31056,41396,36197,36909,32532,33700,326201,50003,44002,37429,33230,36364,33556,50170,36268,41480,35369,41300,3904
4,41384,32244,43483,35987,42003,31614,42564,41387,Entrez_ID,45524,35351,35946,42257,41259,39626,317923,31503,33274,38015,32419,37302,43671,47878,39529,40916,31162,317915,32034,40020,3771872,3772576,31674,34191,34383,38495,38818,3
9096,43820,45880,33292,37644,33635,40963,40268,37991,36348,38638,318129,33324,37131,43227,38470,35211,43126,32778,36595,36838,33659,42787,41403,41031,318098,42339,37015,43593,36532,38473,35911,39795,37941,37590,43184,36806,33749,
34894,36615,31641,38180,318135,41339,43177,43067,41036,32036,41640,41368,43413,38432,246458,246608,50442,41160,37929,41155,41520,32610,34627,39333,42002,40345,43183,39075,33474,33874,32199,38058,37128,32327,34317,33569,318956,26
1621,32592,39232,31358,32401,31923,34037,39020,32399,37469,36596,34679,43440,37004,37709,31408,43136,41831,5740766,326156,34127,36865,39582,35913,5740276,38741,32299,34819,43867,41297,32329,32148,45879,31591,42788,5740455,34565,
318136,37614,4379858,318160,37295,35047,42426,261611,36573,35380,3772574,3772566,40205,32035,37458,42581,40004,35735,36958,44887,42970,42432,31898,246587,3772393,3772088,33839,37182,35708,41202,326170,36740,40266,34038,38583,37
534,33349,43583,36011,31340,34732,36016,32154,37229,39740,36244,41887,38484,38501,43895,42530,45326,50350,32285,7354475,50001,35886,33657,38434,261626,39056,33934,37619,317953,38296,38297,33432,41513,39752,36601,42049,37435,4008
8,32087,33170,39748,43243,249663,39165,317907,40612,32353,50044,31111,43275,31650,35745,31656,39071,35013,37949,33200,42981,33727,261623,42930,5740424,39954,35989,36924,40666,45396,39543,42455,3772646,3771806,36243,32488,326116,
5740275,3354987,41308,38507,44013,36634,34786,36586,39055,318825,317857,35875,36849,50466,3346150,317852,38719,37940,246442,44058,34673,45875,35090,33641,38410,39264,37575,36501,7354376,7354377,7354378,7354381,31103,34322,40598
,37545,40071,40918,39048,43530,35746,41518,33726,34260,33534,38183,41767,317877,43551,41967,38972,33718,39049,39991,35513,41134,42893,37227,35417,39077,37748,41851,35104,318799,250710,34827,31099,41452,42697,43756,326264,42940,36
076,35940,32283,246651,41144,38942,40051,39396,3355069,5740195,42361,32694,33386,31417,59239,34117,39567,37106,38994,36957,38663,44098,34639,36081,41919,41221,35396,40083,34289,41033,40333,3772042,34611,37083,32680,37399,36292,3
2821,37166,40450,31718,41473,39231,34176,45780,31094,34351,41252,43043,5740596,38443,34878,37887,40897,39517,39085,42335,32326,37294,40524,31136,41605,318631,38751,38813,39422,36668,246386,37560,41222,34530,32426,30972,31858,404
92,41248,32093,38954,37822,31251,38610,44910,41070,38290,36060,36528,39237,38517,35264,33695,31402,43429,42676,35061,32672,41238,41112,35231,42560,36550,35622,40287,31743,33047,246573,41982,35687,5740325,31212,42242,41381,37219,4
2683,42860,39317,33540,42723,38104,37147,36009,37548,36753,45588,39106,32558,38315,40608,59261,41044,32874,36965,42701,38472,44010,37663,326141,37137,34181,41821,34067,32469,246595,41278,40732,318251,31497,46391,34477,2768870,38
518,36396,40102,43582,42216,318573,5740503,40713,31385,34623,46386,34237,41894,40059,42832,40279,49090,40085,32724,43748,36588,41136,3346141,36651,40922,32072,31021,31198,2768992,3346208,246620,42801,41469,40816,35126,31391,4229
4,4379889,34064,42523,34087,3885628,2768998,246514,44297,40491,117366,33613,32767,38390,42576,43014,38869,33022,2768668,42280,31055,318992,43825,43318,36368,34023,38889,326109,318203,41746,140439,32296,31695,31461,43414,40579,34
645,318930,40001,38305,42890,39138,35180,32053,49077,246420,36427,40031,2768965,42078,40575,45706,33354,35284,37223,33812,4379917,32476,43707,38252,50381,246533,38235,34152,318260,36776,47253,42783,40180,35221,46085,38454,37999,
32665,35125,44057,7354414,50395,35153,32098,35620,43805,35916,318252,3355048,3772540,36862,41148,42527,38364,34652,40156,37376,40721,32064,40373,34952,31526,34792,35656,31843,59178,34528,42134,37641,41394,34106,42851,40486,34981
,50402,50149,246511,37028,31480,32980,32354,31606,40153,42829,37697,42762,246582,34440,32620,34598,41191,33931,35554,34039,42477,35291,31326,42356,40422,43104,31729,38906,3346202,41742,42390,38337,38502,39854,33953,32272,53558,41
626,3346192,46032,33530,35984,32447,42295,34961,42702,43324,35003,37025,36017,38535,39139,31949,31857,39316,39550,36379,2768718,31186,42061,32549,37134,2769000,31777,37567,38012,35271,34490,32748,35704,39389,43514,40703,39871,33
55016,31550,35309,36392,37968,31189,36284,37648,35238,43506,43608,37445,53446,30988,42045,34090,39504,32251,41863,43326,31509,34184,39262,43002,39383,3772691,3771923,41157,37453,42929,31070,33284,48373,39306,32504,37985,38731,41
017,34485,36248,41726,39409,31520,31935,5740349,59163,32485,318031,39461,32626,34577,41758,34505,31702,7354433,31761,34434,30983,39238,33308,33600,34839,41644,5740879,5740394,34625,33347,33705,318224,40429,41820,43577,2769004,35
353,3885598,31169,3346207,37342,36712,41450,37783,33476,40931,41771,38957,37191,41937,40619,33175,32850,32079,44831,37944,36757,44858,3346227,43256,32135,5740347,44258,42620,31056,41396,36197,36909,32532,33700,326201,50003,44002
,37429,33230,36364,33556,50170,36268,41480,35369,41300,39044,41384,32244,43483,35987,42003,31614,42564,41387,34143,39726,43362,4379851,35131,37132,42391,33647,37556,117300,41272,42180,39051,41092,3885637,41455,41710,43872,39564,3
4145,32822,35261,36789,41159,42481,32799,33656,40252,31990,43408,39738,34303,42429,41124,42992,35535,36121,3355171,37126,38334,38322,32253,326171,7354403,40999,318053,41823,41877,43521,37882,318938,36012,37246,34356,35585,34226,
40137,33793,49893,32682,36688,246551,40228,40923,32076,33332,35728,32723,31193,43265,39320,33865,41625,43782,43697,40330,117369,31648,42505,40346,39065,30970,48421,40543,33299,32991,43997,3771877,3772432,39559,39294,39393,31880,
43268,35877,35681,38600,39964,326256,43504,30992,40263,36258,34873,40339,45845,38823,40288,36428,39070,40552,39444,246599,41261,37149,38036,37558,33783,42507,44750,33858,32881,34882,39907,31020,33639,43918,5740699,34752,41229,32
144,33924,5740318,32775,30994,41677,37861,42657,32792,36848,34114,2768882,38080,41994,42810,43034,34044,32849,42947,35220,43590,35255,43213,40562,43797,43780,43999,43244,33079,41032,43497,38857,5740282,36454,33780,36762,35068,42
028,36232,42310,2768908,41225,37947,33901,34018,3772603,3772383,43155,38362,3772100,43353,43843,42750,318246,2768976,34446,36488,38911,34543,33765,2768945,41149,40128,40309,37413,40997,34429,33085,41579,37514,38192,48440,42503,3
7890,41807,41002,32468,40669,31498,35901,35094,31572,39627,38895,31502,34171,50320,36383,35832,318096,33501,42206,43193,38917,32813,40871,33844,39566,35310,42622,35525,39999,34109,43098,39774,40348,45285,33826,35043,41420,40016,3
0991,38622,40935,40430,39530,32348,43936,31463,44510,42670,37036,32676,31794,37954,36466,31436,33461,37849,37834,34115,33571,3355011,31249,36976,35878,33190,39486,33179,31558,36051,35322,40833,31536,32075,40745,37937,36656,40555,
37531,38900,36626,35299,2768977,47852,42465,43070,318003,45884,39947,32216,39004,44637,42547,33363,41345,38720,40363,32619,39382,33515,33377,35546,33927,43009,35567,35568,33606,32864,42645,39518,38102,40720,36272,246637,39514,32
166,43149,32489,39516,40356,42014,30979,44498,31168,39078,43777,39129,35111,41768,38693,37520,3355084,42551,246534,42589,37708,39581,33520,42920,31237,36547,47065,50416,40056,3355132,35480,35828,31766,34251,38147,41318,38666,409
53,3772376,35004,38273,45248,326246,44432,43453,41445,45012,39539,43747,42210,38614,36333,35768,36536,38121,32581,31896,34304,2768877,37563,38445,40427,37905,5740462,38496,33759,44392,48571,42588,31943,39989,37419,39156,32958,38
331,326235,50068,38003,34259,41809,39950,31507,34779,40271,38461,41708,37807,36920,38095,41428,42835,36084,37017,50302,33028,34395,44018,33221,5740840,32375,49241,38941,3771779,36868,40961,32397,32994,32081,38879,40009,31728,359
96,36127,43347,36829,37107,38089,31469,33923,42126,326217,34749,36705,39730,42066,43040,246389,41502,40860,43069,40671,41290,32293,35174,33351,37089,318751,41027,34116,45908,246473,39916,36135,42634,40160,40681,42112,36279,31963,
42018,41676,192528,37129,35888,35077,41822,42271,39068,32545,36283,36057,41030,46717,2769003,37232,38810,36246,43015,38182,33909,7354423,38809,33487,39777,39221,42166,41250,40335,43450,40124,43081,35604,42694,34068,32921,37888,4
1547,35885,40400,41121,31005,40042,38522,39252,38615,38657,326180,43630,42245,37037,40826,40019,36438,33908,318886,39533,33652,35689,318035,35898,37555,36202,47384,32342,40061,43875,35941,35694,33133,34634,32788,38407,38029,3699
1,31002,35910,34122,2769005,43092,40551,40644,40706,31178,33882,31934,35190,35424,37364,45922,5740299,33440,38594,32320,41985,326329,42350,43392,32873,45041,41772,31980,35122,35383,34406,31270,31038,31644,36770,39441,34275,35234,
36508,40282,38985,48844,38887,43033,318916,40982,44005,44227,31317,43099,45021,43904,42831,34368,31622,34386,31808,43127,43699,32967,5740816,37412,31406,38624,32319,42877,40241,42203,35124,246650,34435,41000,36030,35107,35408,57
40629,31447,34419,59128,50191,31120,35055,42396,39572,34310,3772580,3772419,36551,34947,47220,35016,38581,43786,40592,35692,41344,38976,44291,38382,43046,40599,31194,32267,39508,35571,40522,38805,3772483,34180,31010,5740446,4051
3,33117,32479,40293,31721,7354383,41254,35918,31429,40690,326208,40704,32037,31736,34591,49580,246488,31126,34998,318794,39426,40342,35773,47611,41153,36526,34621,37791,2769008,43223,3355093,33742,38085,41170,43746,36174,33959,5
0438,34837,40981,31962,38562,36814,35531,37331,35374,31434,34189,318977,42001,40640,43651,41721,33248,39476,34774,37564,35277,40928,39172,35807,34538,39098,40167,38845,31547,317891,41354,43168,39941,5740544,2768669,45249,246467,
37122,40921,37884,39675,32229,43020,43503,43819,39304,317834,35266,34186,35344,31424,41955,31339,33257,31696,37016,42340,34030,35937,48445,40340,41991,53579,37030,40002,49425,40965,43562,5740712,36481,50236,40521,3346176,36758,3
1704,31855,33277,41697,37806,38723,47173,32534,41961,33711,31701,35037,44856,41293,38596,38298,36335,35786,38955,33561,34716,41361,32753,40222,33688,41704,36533,32028,44787,40193,31831,35156,40349,41971,36375,64877,34951,4379850,
37174,32606,39643,5740224,43579,3346162,2768673,5740142,41174,42351,41583,43607,41034,38463,42187,32574,43682,32151,41309,41601,43535,42160,44817,32495,35615,42289,3771796,3772559,32761,40858,39996,34680,39876,3772670,3771960,3
772109,2768920,42883,33231,33626,34560,38814,36641,35029,40374,37571,39976,36654,42300,33399,37867,37712,59150,42226,32322,33285,34483,40323,39023,43729,35699,42784,34912,40280,42004,32324,59223,41990,37879,42298,40130,35866,323
23,40873,38539,44095,36577,39682,32483,42733,37188,42268,50364,40325,42433,3771968,39034,34005,31190,30976,50266,31610,40296,43427,37355,35357,326240,43869,246452,35800,32211,42608,42162,34953,3771920,33431,33141,318833,47729,41
417,37701,34762,248194,318587,251984,32201,33814,31724,33599,40613,37964,31810,31322,35793,53431,35926,41760,31491,41009,40689,35042,32215,39097,35138,33403,33976,33291,33565,37524,33161,34908,32002,34013,37136,39652,3772165,413
06,31382,41163,44289,40147,32586,42865,32986,33361,42037,42956,43610,34121,31540,44448,41882,33957,35970,36395,37874,35158,38992,40587,37135,38101,41242,31965,38543,33935,32629,33952,37173,34963,36996,4379884,43620,41703,53433,34
355,34772,32972,47408,41650,43447,32498,36692,36886,38222,37825,36768,48973,34118,38084,38771,33776,34544,34891,40080,33861,41720,32645,35145,39390,31007,39775,37285,48312,2768881,38050,37818,318193,32120,31185,35528,35118,32865,
31266,35260,34028,32343,32857,33207,41366,7354406,7354408,7354407,40221,41941,31964,34011,42127,41826,44669,39762,35181,32011,36227,32165,37304,36713,38088,37009,5740407,38428,41302,33545,31295,43078,31967,41046,326151,41359,381
96,34412,33193,45815,40152,43490,35296,43991,32677,42807,41311,31873,39132,40988,38038,31744,35338,37332,37368,31441,39939,38181,35227,49892,31912,40471,48986,43990,40567,39336,35623,38357,33197,41192,31780,42189,45234,38306,3901
9,34091,39052,33016,38735,7354399,42359,39629,40872,31384,33703,35753,40257,39813,43029,31839,40862,48441,38464,42594,41671,44154,36905,37245,38169,3346160,37422,44007,34731,36039,34131,33855,35608,32963,37707,34893,36086,31451,3
17825,32088,36623,31380,35340,31581,38612,46035,30995,43800,38069,49803,4379885,53471,36403,39050,50169,39028,38203,318981,41207,40637,37012,36916,39933,34630,41023,37244,38520,37839,39930,40941,38515,41086,7354410,7354409,73544
11,37470,43672,41733,41866,34840,34945,36687,36593,41612,36045,35691,43500,36307,40003,32461,33391,37516,31642,42269,37815,35376,31944,5740790,42463,33975,33425,31568,35683,41040,41571,40310,30998,31320,42690,317926,32742,42362,4
1231,41448,42619,38381,33578,39458,32224,42454,36960,2768719,40254,31023,43505,36702,3771983,3772180,44783,36990,36046,41701,40404,36592,43614,2768858,338392,3772007,32726,318644,41103,43102,41521,37075,35943,50373,32281,42737,3
6778,40724,33234,41552,246439,36792,59247,40948,33644,39053,31054,32903,43831,43317,34268,36644,44054,40794,2768999,33743,39965,37181,33692,31585,42105,38233,38747,34277,32398,41407,33960,326267,42341,42292,39310,38097,2768688,4
1817,39656,42502,44830,42060,42244,41355,34414,41305,34965,41816,43689,40725,43604,33601,246602,50007,41687,48971,33519,40035,31560,34753,42669,42077,5740672,32766,31837,42519,42473,32509,41194,318566,39527,246552,31489,3772064,
3772218,38261,36541,40780,42079,39457,37141,118436,34537,44108,39101,326120,42687,33920,33401,37407,32516,38922,41549,33077,37305,43074,39079,42148,33398,31903,34633,317887,32868,43425,44936,41797,42492,34631,37119,35709,33224,3
1683,33046,31490,3355133,36694,39388,32435,38500,53436,42597,318935,31383,31682,326146,35575,42939,34138,41376,53585,42958,41989,41062,33847,35177,38302,39736,31268,34323,33609,40095,38989,41035,33294,33455,41789,35817,326199,37
71738,3771965,36887,35035,318753,38750,319028,32497,43435,41875,42109,44441,37926,35505,37187,43738,31397,31236,36754,31848,38457,31267,34307,36782,33112,250736,32636,41869,42385,37960,39364,36459,42905,36643,38629,2768687,39888

,33781,39054,40314,36240,40220,41647,31913,38034,34529,38197,40402,38201,38456,33610,40145,35060,39432,44315,35193,39879,34110,41288,33902,40258,35256,35781,31013,3772182,40509,36157,33222,45816,318103,318682,31806,47942,39293,40
649,37161,34003,3771966,3772677,31205,34701,42859,42964,34720,39690,43006,43373,31613,42954,32382,38346,36496,36253,35880,40620,318790,43570,3355116,7354421,31155,32449,36109,35382,32531,34089,42491,5740281,40389,42925,38087,389
44,36889,5740205,43499,31081,38325,42821,32943,37306,41351,39406,326115,41419,34879,36041,31603,40695,36156,32591,42497,319046,44748,41364,31139,32507,36457,53425,37218,39093,38716,32009,317932,40054,36743,35073,39066,36107,3672
5,31915,43436,43789,37198,5740536,32526,41575,38533,36954,37776,37918,42885,38545,35986,31045,38379,41825,40679,41694,35253,47895,37770,32698,39041,32387,36507,44390,3772069,3772329,40944,38411,42185,33734,35715,39850,31950,4192
0,5740408,42178,40714,32317,35222,32988,34169,3885631,318133,40265,48775,3772015,3772216,32279,43199,39204,39919,43489,34282,41189,40163,38733,41639,34453,35023,37044,37652,3771829,39332,33686,40359,43231,36037,35402,47767,37627
,41503,35452,33822,35684,40718,40411,318947,34624,38936,33650,43382,35711,38503,36682,47594,43230,5740414,318595,34250,34357,31361,33564,37761,31639,43913,43713,42726,42918,35920,318249,33118,44110,37587,4379919,36919,43357,4119
5,42843,32597,39363,42192,32294,41425,35386,36529,33717,31158,40945,35419,34966,33529,33757,31344,40097,36080,42026,43563,42633,35259,59173,37836,42919,31835,326139,32982,43757,38601,40159,45320,36475,34449,40358,32202,42366,3998
5,43827,37536,41203,38303,43616,38344,35127,3355160,32973,53502,33473,46456,42993,43169,32916,5740235,33103,32587,42508,43466,38526,43533,41401,41784,48903,33469,42894,41850,43417,42637,39415,37535,33194,2768853,35149,43262,4833
6,43292,43160,39067,31815,37703,38602,39852,326148,44529,34264,38605,41542,39017,32662,38715,37034,43547,31217,39654,43814,36209,43018,43100,39479,37185,40719,43448,42393,34718,31222,41083,32529,39027,40638,35315,33226,42817,3319
6,33114,32686,34315,35179,33033,37978,38964,32885,39549,34930,31731,34374,31899,33255,34132,33007,39671,41362,32102,35537,37988,41133,35934,38224,42254,33000,43423,37591,38695,3772300,41217,35091,42752,37871,31738,5740323,34373,3
7444,43105,38353,43438,41868,33532,37209,32446,39234,34736,35370,35761,31586,40176,38504,40893,38369,37749,31601,33524,49713,41446,317898,35737,41176,43529,42728,32791,43502,35969,34985,41538,39588,33135,40260,33932,36799,33992,3
1145,43669,33655,45460,39135,36128,42389,40925,53428,31745,33356,42230,33948,31143,31244,32290,43916,37431,40177,35390,41804,38161,34286,31348,35559,31807,36717,35712,44279,41204,34393,39987,39137,36513,34803,3885647,38352,43495,
33526,37296,38928,39768,39547,33341,41951,35953,36563,36914,33241,42291,33563,35442,45840,5740590,31035,38286,31605,53569,32679,42937,32312,40036,33119,31089,37068,42195,43652,32242,38433,37449,37410,31732,33282,41126,35159,4231
3,39679,33076,40139,39944,31171,37277,32341,43473,34426,35865,31841,32107,47718,40384,42058,38455,33346,33434,46662,32969,44030,37375,36772,42973,35511,35355,40149,36881,40863,42805,31388,37164,32334,31221,42221,42543,34982,41263
,36963,40975,35738,31717,38430,39860,2768674,33680,38756,35688,33132,32572,32989,43508,38019,50058,42520,31697,44046,33951,42302,42414,43555,32506,31801,33541,38345,31083,33714,42357,31612,31119,32674,37155,37730,31826,36048,3701
4,36146,33521,39284,35892,32358,318636,43093,36269,38451,32008,43724,36447,38760,32113,31589,38148,43735,38532,43008,41550,53442,36984,42900,32212,39552,36038,326165,37533,32625,42325,32230,43982,41313,42996,38807,38178,35286,415
33,32520,38816,44339,32888,41104,31525,246496,38103,43345,38229,38377,45785,43004,35198,42462,33498,42934,37981,36594,42850,31500,32300,32295,33648,31110,42279,39557,246410,33128,32183,42590,34568,38116,37196,32013,47219,36560,39
717,35785,36499,246459,32063,37973,34969,43690,35297,3885643,46201,33516,31866,37463,38155,32437,40557,246421,31524,34445,36606,39847,34095,41190,40912,35257,35083,32568,35364,43228,45525,31016,246618,35944,40998,32047,42754,368
30,32222,246629,33210,33191,39133,50253,37896,39917,3355164,4379834,31735,34380,35098,42518,117414,38730,37040,3355136,31157,43179,2768843,34450,42897,38787,36150,31318,41819,36079,2768879,42561,34385,34622,32480,40938,43552,504
03,35375,40073,35671,40134,43901,38389,32976,31881,42380,36285,32335,32050,43236,42834,41161,39410,41709,32940,34747,36696,43792,38677,40181,40111,41927,37385,41266,46008,41911,40300,40665,34784,31739,35192,39554,37935,47757,3679
4,36810,35540,41636,44233,32523,33643,35347,31851,31681,33298,33914,33396,37047,35609,38801,36273,44127,32593,43399,43391,38534,38514,32790,45883,39450,38478,36357,43783,35744,35697,36825,35490,36652,42030,42031,39314,43492,35121
,38969,42243,31596,246445,37323,49805,37637,2768916,43580,35891,33973,40687,3771973,39625,39894,32259,33304,35248,40959,38779,37462,43042,31401,32688,43767,43532,33369,32350,41060,33236,34097,39551,32768,39212,43170,42449,43828,3
7916,42936,35673,33627,34311,42999,318210,39108,43892,39521,39693,46194,35560,34715,43830,31142,41304,38808,38185,32080,31945,35488,38890,41597,33254,42515,40643,35985,31869,41931,35957,43720,36681,3355135,35483,39260,36583,3932
5,31518,37476,34787,40336,37152,37817,33704,43794,35343,37205,36400,39119,34274,49953,40994,33017,38076,42652,36497,34889,41258,36281,34065,40169,39076,46719,43128,31399,32337,43290,31513,31335,41343,39157,39979,59170,36685,35116
,31418,32014,32142,43349,40305,33677,40700,32722,35085,38656,35799,42376,32217,32764,35522,43952,34519,34397,38313,38208,42586,37996,41528,38643,31293,43293,326216,34170,36423,318212,33421,44254,34154,35971,43719,42592,36252,4111
3,36683,38374,42440,4379867,32637,37919,39734,40231,33466,31012,39228,39948,318872,32494,46140,47272,37862,34804,34432,33373,32954,42055,36159,33233,41938,39611,43095,32407,43691,47091,36985,37325,3346235,44861,35420,41459,48805,
47186,53434,44125,38492,35664,43238,32226,42866,42101,40131,34754,246580,34159,32554,41578,31921,43811,53586,39337,32607,37931,35000,32023,34714,35879,31667,53472,33823,34193,35228,37288,40368,36290,40831,41120,38846,39043,42394,
41522,326255,34885,36967,39691,31386,44543,36104,37994,42128,43656,39610,39524,40861,41778,41053,50225,42386,35232,34480,318991,36720,38736,38734,32909,36237,38232,33388,35109,33807,38785,32661,33899,31342,34805,42201,31208,4062
4,43809,41228,46260,49505,35324,44433,40357,37786,37804,42852,33963,38469,34997,31593,33427,40410,42943,41179,37393,38542,49968,33045,33900,33212,31977,33999,38763,37950,36205,34001,42747,40507,33110,42059,32962,32612,32039,38721
,38995,41878,34636,38901,40294,41855,34111,40851,36951,44965,37301,32608,34433,37808,41434,34398,36955,326186,43325,40974,34328,38343,34921,39878,31665,43197,39259,34847,32789,36860,35405,41672,42960,38055,48481,32924,38883,36746
,42504,31887,41247,33139,40459,40453,38397,34004,38413,42358,41788,41071,34618,34242,41656,31098,318843,40967,5740757,43493,34096,50246,41182,42086,47080,35928,318221,39843,37124,33353,41935,47121,42466,34685,34616,33263,36956,35
954,41026,41114,31331,35695,42267,41184,35395,35974,35988,35100,39217,32670,35147,50356,41437,39628,40934,32737,31247,41436,41014,37482,33424,33379,39630,35113,40000,39118,41045,31604,43751,35197,31914,33972,42626,42428,36552,352
18,32841,261634,37702,261631,43003,34996,38421,2768981,44785,39722,39375,46158,35194,36719,33562,34939,33018,35682,40126,251690,34324,36108,36986,32007,326234,33504,38424,36138,36376,39735,34442,38885,37859,36398,36856,38934,384
23,39439,37875,42464,39302,44793,40715,36589,44724,34826,33567,41744,39254,42836,246653,42141,34155,35128,39746,43235,31582,42169,42630,246652,53437,38262,31009,326193,38628,40553,40081,33214,46078,37860,37921,39267,32158,34172,3
6619,34481,38494,36022,41495,32508,40626,43905,33965,38193,32777,44702,40894,33083,318043,41840,34699,37201,43618,32598,36689,46080,37647,317815,45928,36019,318559,43359,33259,40712,43958,3885654,37033,31106,46038,41067,43077,34
608,39413,31842,39478,31387,42137,36731,38070,37084,37339,317952,40431,5740708,3772055,43844,35189,35949,31795,41073,33982,38647,42500,39153,39164,31377,38485,37741,318016,42816,42571,33503,40958,5740853,44128,33597,42290,43329,
33111,32338,32145,36911,7354471,41616,36598,33281,32546,43842,37704,43950,37936,43182,41801,42124,34750,34514,36851,43057,38714,38970,42439,50227,39650,37478,33499,42748,34527,38978,36073,45268,35144,338395,37329,35565,37728,440
59,2768909,32953,34009,31240,32576,40045,36948,40852,37570,35337,44250,33602,36061,48535,39826,50296,43222,31239,38136,42191,38446,44451,35757,40791,248102,37892,35140,38195,34831,32430,43573,40792,36321,42062,41827,32282,38259,3
6492,40190,31121,42764,36068,37628,43348,41785,36295,33857,34924,35298,40879,31597,35053,31708,32514,33237,36415,42689,32938,33983,36102,43103,117419,44260,32640,31805,39645,34548,36574,38915,39759,36144,40664,38646,31631,35267,5
0139,34626,2768951,36025,37803,33367,35756,35426,37467,31090,44620,59218,31398,33283,317935,37216,32518,41193,37401,32406,53444,43517,32770,41743,33227,35155,31588,40399,42600,33689,2768716,32196,32038,42820,35931,31811,32408,31
8079,34677,34962,42572,33849,2768892,43550,42842,33182,33134,34504,40583,34443,32288,33653,38956,38081,31420,46091,43978,42266,38945,35200,40256,37589,32729,32701,31044,36764,34740,42452,42789,50250,38732,32902,36040,40490,37427,
35262,43528,38059,34993,3355120,44672,42662,40800,48785,38063,41377,32703,43774,2768670,42265,39243,37809,37794,43240,40566,32515,34610,38637,44271,35900,41125,35932,40369,36053,41805,39025,32624,318578,33136,33690,35680,39399,2
61620,32537,318600,39213,35381,3885607,326173,35590,33034,31176,33260,33005,41447,32045,32033,39858,35627,38110,37019,36747,43400,50323,44653,40129,35848,36173,39122,44353,40565,38017,33049,32622,36498,35293,40582,42721,43355,39
430,39935,34408,45329,43557,32755,34073,32140,41185,34120,34722,45827,40451,41168,38355,42027,38516,35930,41873,39014,36028,4379907,4379908,41042,246638,39434,3772124,31147,35539,37927,42813,37601,41641,3771751,53556,38982,33739
,40343,41056,46074,49809,38873,40663,42174,40096,7354371,39158,34708,38047,41404,33198,36961,40605,37035,38360,33172,39657,35993,35966,31363,31818,42871,40559,41847,31140,34669,35555,32055,35747,35044,37021,41100,40936,37938,3863
5,42985,34683,35963,33167,43572,42758,42342,40132,37894,38530,3772349,3772641,33343,44100,39694,3885646,40786,31658,33056,42990,37330,36064,37781,37824,43017,38819,44885,37249,40324,33137,36436,33220,36603,41395,35895,41154,3675
9,34287,43019,44573,41838,32660,34594,40960,35288,37925,36168,42498,33883,38888,2768677,43242,36670,40548,40048,3355114,37203,38206,38013,49228,41608,41526,38987,33738,50251,34137,49701,42587,41689,7354380,31153,39150,37414,3592
4,40191,38528,36362,32613,32164,318990,41741,326198,36386,43558,40589,39535,41770,42802,33229,33305,42046,326213,40303,44531,40138,318841,32258,32563,33276,42703,36311,37400,4379861,38100,34135,42593,41443,35917,33853,40273,4234
3,31628,39969,42136,44811,41438,42770,4379817,45318,53473,30975,42215,43428,38378,32373,32149,38109,33235,39875,317824,43594,39536,32898,39724,40437,2768661,38865,35849,37979,33105,32241,40062,39206,43561,32569,38505,41940,31874
7,42882,39255,32116,42828,40240,36210,34488,43384,37397,33043,326119,38669,33654,47082,39251,42735,37078,34284,46234,192507,40970,42283,40174,39104,31373,40360,37261,41842,35929,31346,39180,45368,41340,31907,53562,39513,36111,339
87,44791,31598,38367,42175,31225,31097,33184,40754,34404,31657,50133,35123,37372,3355130,32471,42402,41137,38698,41839,33651,318082,38640,43404,42903,42516,31537,38742,42757,48552,3885575,318231,35564,32169,32605,39099,318077,40
172,37858,43852,45339,42258,41109,40654,40187,42020,44160,43159,38961,44660,34892,44183,36820,32162,32269,34354,35172,40717,41783,38700,38376,34688,41960,44497,38697,42595,38255,31392,34950,40540,39820,3772183,3771756,326245,383
95,38490,38864,31430,31562,43178,34785,31228,33265,40034,43754,33809,36788,32104,39750,37430,5740674,38561,42051,318095,32551,35662,39903,37914,31086,41150,46243,41776,50363,34648,37832,34503,40781,37992,59212,38336,36338,43481,4
0383,44360,39751,34709,35084,2768997,31011,318161,39749,33732,39480,39856,318599,41857,35899,39686,35938,34738,41205,326135,39685,31764,50405,33805,42000,39689,3772476,38487,36995,42840,59246,34409,43283,38488,326232,33938,73544
66,37633,37546,41942,34387,35795,40438,41603,38057,38373,43277,3771951,3772662,39069,41074,3355118,47906,326158,36773,35097,40162,33027,36514,32340,38243,42209,36766,44544,42303,42397,7354473,59260,37071,38033,38300,40049,37934,
32410,31104,326161,43148,41215,37643,31608,39833,38796,32240,38031,31904,40790,32278,37451,33836,36014,41029,40622,38062,40996,41551,37820,64874,31166,33240,39758,44029,35336,318259,38020,31310,36464,33825,34070,43768,32934,4264
1,32671,40932,31521,39914,41427,38648,36239,35397,38001,31523,38769,32886,41983,326192,40178,44554,38593,5740156,41896,44870,41525,37618,326127,37659,3355013,35754,32918,32604,5740700,39086,33943,43164,32596,32866,31230,35702,39
240,38794,42272,34437,40876,35923,42806,42881,39972,32462,38363,36118,40843,39095,47396,5740362,40597,34158,41876,40332,43328,31804,37699,31499,32819,43984,35952,37632,3772719,3771946,32522,43031,43730,37415,38418,43446,38187,35
041,43772,34974,42509,35730,36282,32933,31874,37335,36125,32349,50428,34675,41013,41899,43694,31812,35378,36133,38046,37384,40472,35850,36797,32780,53573,31181,44001,39659,35975,37602,33418,41558,2768660,33204,41022,35331,36585,4
2125,41837,34495,38221,39934,44522,41598,42179,31567,32208,31970,37118,39684,45031,38913,39997,40590,36566,33840,37912,36160,33409,35219,36123,35030,34421,31713,41835,31611,36171,40015,3346237,41871,36420,44701,31174,40074,40361,
42545,38068,47781,43515,39998,326214,35430,318680,35215,36374,35072,38323,31022,34183,32730,40189,39809,38342,34496,43437,40610,38499,41849,35842,45969,37561,33502,39141,42436,31687,33458,7354476,50188,31253,45784,41162,36540,38
375,3355134,40281,40495,35066,36027,34378,40946,41068,36945,36570,36546,37475,40506,41740,36912,31245,39721,48448,34881,37381,44228,32328,43398,32400,31535,318709,35154,40558,31319,31856,32815,36981,31660,34126,35732,37895,42858,
40377,37072,36001,246497,31127,32204,40044,35591,40014,40686,48782,5740551,34320,36271,37966,33761,34362,32330,31163,36372,43946,38611,32771,44786,41423,252479,41398,44275,31771,31577,39883,41844,31571,31260,39147,38692,41341,43
79886,40064,34707,31741,42239,117332,33195,31376,31292,38194,43491,43559,32937,326143,33693,45343,38400,44263,32882,31840,32872,39404,37178,38667,317946,45739,40577,41433,33125,38718,36639,37020,36821,31259,37581,32228,40053,324
78,40787,43812,42005,37344,33219,38219,34681,42143,35524,37768,33835,38905,37723,31150,318706,45906,43576,39321,31543,43026,32552,319048,42490,31684,35677,32457,38694,38786,31042,2768667,41390,33158,40308,42129,31905,31775,36760,
35955,35501,42891,33782,36727,31165,38749,32501,40378,31988,41260,32505,33611,37154,33838,32379,33646,33967,4379854,42927,35882,39250,31071,318857,32315,36260,42876,37873,32683,36360,39522,30996,41147,38493,45338,33989,33348,316
09,32970,3772560,3771728,38151,43130,47869,40683,33140,35478,41374,38540,34231,38655,37754,35102,31026,36908,41993,33649,39770,44155,41456,43835,39268,31647,43546,32854,43660,34321,42646,41675,45398,39484,37946,38258,35837,42839,
41707,59215,32977,41269,39766,48632,41178,38963,38820,35365,246461,42642,33979,38228,40386,39083,35532,37554,42183,42615,39387,47285,319025,34975,41865,33775,33607,39829,37729,37076,37585,36299,40904,33716,33256,46020,40699,4068
2,38006,33301,37063,37767,50008,39111,32594,40508,31948,33719,35507,35787,32027,3885573,37795,40789,42857,34167,44011,42525,39966,36697,31184,46415,40950,36224,317878,31309,3355079,42647,36997,43300,41363,36809,37455,34711,39013,
36655,39769,37967,45683,34196,42663,318911,36921,35362,37664,35762,39438,36926,39936,31817,42285,117353,43637,43927,2768875,42899,39613,35411,44843,41406,40515,39448,41091,32573,32935,31942,37450,39624,3346238,192509,31156,37011
,42749,33303,326128,35784,33939,42064,43856,38260,37511,46040,38079,36054,38979,39400,41638,38649,38086,32124,43673,39844,50392,31483,31160,31618,37785,35713,33402,40844,40195,32776,31798,32109,47894,43215,38529,42370,41118,43241
,34352,31381,50005,36750,41458,318743,42146,47998,42632,31678,36363,39088,40668,32738,42181,34396,39026,31107,326259,34783,31979,34906,38844,31574,36857,249072,34371,36617,42549,33988,37537,41690,35002,42880,33684,42888,32990,318
97,32541,41342,32579,36793,326206,43028,317924,31976,31557,36736,42089,317849,44484,38943,40302,38023,35236,35889,64880,38248,46006,40388,39720,32273,33645,3772312,40289,40168,43571,31351,38227,45668,35527,36722,33921,32987,3607
2,37751,33834,44096,33109,36137,39818,40549,45360,31953,45307,31991,34516,41262,41138,31213,41646,32979,34646,34422,39688,38256,36337,41090,38159,31148,40084,43775,33715,36763,35588,39899,43862,31052,31659,35674,42299,32046,31177
,35453,39270,34428,250735,46027,43676,43153,42624,31175,39561,32585,42371,39874,40723,246457,7354379,33905,41591,38037,38654,3772082,36578,251900,37757,38093,34329,44703,50060,42691,34674,37662,42110,41085,36732,35778,41180,3888
0,31449,40617,36579,43693,318206,33560,38831,37833,41592,44889,326121,34021,39394,40694,31364,34300,35529,50335,41080,43459,36527,38324,47769,36147,36411,35268,35379,31940,2768940,39082,317928,31224,39619,43344,37168,40659,41399,
36442,37932,32631,326205,39772,35311,36309,31987,32635,32946,33215,31844,40933,38959,38176,38051,36749,39263,43163,318824,40711,36698,38438,36676,33397,41268,39683,43469,44355,42553,33604,40436,42309,33964,42617,33078,44014,3487
4,43788,38349,38398,38806,43518,42413,38613,34119,246507,34392,32971,34107,43488,32782,35672,40087,39765,33552,39593,34062,41141,31625,43664,32472,34557,326228,44131,38599,35611,41962,43718,43766,39862,41319,40547,35045,38067,360
56,32544,37500,36748,43923,37117,41461,46058,7354422,42501,5740442,35660,3771985,43943,36408,32448,41206,37018,32595,34550,4379891,45783,36568,41674,31706,32700,42445,40262,42742,42665,41749,36020,32368,36448,34549,4379860,40242
,42846,31747,38177,40487,36397,37989,36432,41165,32458,38326,36706,34263,36255,31553,3772280,3772012,42944,36680,33995,41862,34734,36632,43271,31215,37987,38788,37479,53578,34312,49636,40596,53439,39437,35071,36082,44259,32928,40
670,42282,32910,41628,37733,43750,33307,41541,37811,31475,45399,34405,40433,41569,4379911,32292,37640,35142,42935,39958,37805,37706,42364,40642,37090,34524,31716,41365,43556,38420,41271,31496,40030,34112,40661,36304,47457,36544,4
0092,37334,35678,2768996,33131,43167,37070,36303,34541,38174,32539,41043,317813,43315,42093,49260,36059,41084,43609,31043,38096,32721,40709,31101,38930,36831,53445,41880,42475,32395,37661,36289,36495,41848,32482,36669,37923,3468
4,33293,31833,40929,40105,38523,33289,36341,43793,35240,39483,33777,32716,32377,36203,46059,43507,41570,47874,40841,32820,33223,44380,43460,43662,36878,32077,35425,41158,32150,35399,33019,37062,44437,41393,37144,40653,33991,35950
,31095,31772,32456,42986,31638,326226,32763,33928,31082,41122,33338,43180,41883,33015,31359,38477,43001,37069,40739,36031,36426,42496,37378,33268,41992,34992,43727,40660,36208,32878,326122,40716,39366,40403,41347,33166,31989,3778
0,31544,38744,246605,34423,43471,36494,33435,32615,38226,45064,32005,32245,40391,41836,45976,37029,36826,31448,37842,37238,33225,34222,37379,37073,32163,33636,35486,35499,38149,3346167,31350,32984,39397,32638,41957,38332,7354429,
31700,42220,47763,33094,40165,33629,44173,41058,35316,38002,37734,41939,32101,37679,37586,35176,37328,32589,41282,41431,32114,40633,43833,37310,39265,41370,45871,43838,39940,36393,44643,2768680,34430,43433,40693,36975,32115,3189
18,38981,34245,246682,32691,41279,34294,39902,32955,45931,41078,34283,34775,38338,35342,40164,40127,35070,33245,44540,34372,36042,31037,38429,32133,33977,35925,32073,32094,34595,40581,42922,42672,43087,34542,37886,34710,38983,402
32,41718,40859,33339,319043,32119,40186,44326,41181,32851,32752,34790,32057,40155,39961,40983,38150,38283,36927,39986,32684,42065,32992,31542,39608,37924,41201,31813,40270,37200,43981,43725,36918,32352,36050,42173,31997,38598,317
67,31615,32836,44097,38447,34573,32475,32136,34256,34546,43444,33910,36545,43233,39663,32966,38589,34781,35146,31698,53580,33736,39161,39943,36784,41917,35959,39377,34877,32100,36846,31834,35398,43041,37065,31096,34420,42360,3492
7,31776,34500,36199,40401,41317,34767,38022,43611,38321,41845,39882,33156,34748,47216,44307,43810,40449,32111,59172,39835,43795,32936,33465,45970,40385,31405,41532,34156,36761,3355124,31204,33850,39253,42467,35890,38173,32268,370
07,46326,36044,42273,326197,42460,33518,317859,34079,37290,33911,33760,41745,38513,32090,40842,41921,43472,40545,36542,37769,45247,30973,43374,42150,32547,41226,40060,38199,45655,39612,35788,40907,35485,39745,43267,3355163,42526,
49427,37437,37471,41491,44548,33389,34850,36143,41087,40264,31792,34241,44129,36306,250811,35569,39647,38674,39386,34700,37746,31751,40135,43803,38489,42196,33082,38044,34327,53577,31845,32599,34713,45682,35536,33753,37466,33201,
38091,41197,31960,42470,36959,318645,35927,31141,5740565,35027,37800,41028,42208,42550,32016,36302,36876,32566,31195,37456,45380,32351,42653,32439,39218,42693,31001,39381,31473,36287,44266,35348,36280,39548,33350,32434,32460,329
83,42186,37079,37433,43617,43354,39873,37377,41081,43765,36384,42388,34007,48317,36638,42567,35454,43358,42844,43739,47260,41747,43565,32803,37782,36915,38065,40785,38884,34024,38527,46187,59151,3355143,32981,36134,43101,37006,31
211,35919,33059,261607,44505,43192,40505,40857,39877,42991,37043,34197,36358,39587,33744,35057,36562,32548,31906,33051,32732,42931,37042,40710,41766,36555,35509,47218,39210,31955,42139,2769001,36035,40239,35444,37464,35563,43808,
48071,34403,32974,34592,41460,41294,43023,36645,38074,44272,35789,35339,37172,31144,45574,38007,41320,41673,44819,33496,32275,39408,41391,40585,38135,35653,44172,31459,36974,40576,31474,318979,39175,35748,35440,32786,43319,36576,
40234,40171,38997,37864,43758,33470,39570,36640,40541,41167,33471,35570,3355158,38603,40602,36103,39329,35500,34338,40966,3771905,3772679,33862,33314,32502,47762,37846,39596,42510,41498,33712,40158,45250,35594,45269,36580,33638,
34069,40692,40291,31679,35526,3355089,32243,33916,40464,35991,43401,36381,44728,42531,39301,37116,38595,42803,33774,36699,45894,34539,41617,32646,33372,44438,35001,45401,39455,38800,251645,38184,40457,32071,32584,39446,39331,354
00,32205,37367,37635,32510,34778,38175,34240,40517,35956,318908,42493,39764,40544,37228,32321,41139,38740,37402,32652,35052,32463,43700,33002,43717,31214,39821,3772396,39440,34794,40390,42521,317913,44978,34903,42087,31425,34140,
43165,43166,32262,31130,35847,40249,31793,5740176,42406,36129,32112,35741,44238,35292,3771984,3772658,3771897,41244,32440,37221,43456,37130,35652,34360,35995,39757,4379844,31809,3355125,40224,37386,32256,36229,37237,43276,35921,
31265,41256,38073,39319,34479,40980,34441,39202,32941,3354925,32919,45786,43125,32473,32157,38144,35157,43734,33808,31816,43823,40795,44149,326257,32860,41289,2768961,43096,45374,32754,44801,41930,42812,33264,43310,326177,41860,
39842,38931,31362,318563,34364,5740472,318867,261629,39737,41095,38157,45680,3355129,42314,7354432,39990,33026,41864,33390,36612,37646,32564,42165,37532,41859,3355138,39491,47765,37481,40593,42412,35881,43217,44747,34359,40556,3
772133,34185,36154,34302,38590,34416,38696,42015,32715,32274,41981,33509,33896,34571,35583,39445,38060,43516,32601,33269,32611,33845,31746,35586,32001,38186,53567,31493,37417,30985,32425,36767,38113,34215,41870,40652,41657,37953,
34925,39978,318958,36730,36131,37387,39560,2768685,31781,53438,3772382,43804,40564,59176,37217,5740715,36604,40655,318604,32760,36071,326219,32041,42274,33419,317833,37784,38893,43764,36988,40272,34175,37521,41562,33966,41054,37
005,32106,37465,37727,41143,34979,34276,31079,41173,32209,41907,43906,32225,40146,38968,31030,34559,42499,40144,37872,41177,36110,45467,42177,39340,33640,41235,42679,40398,39669,31017,36978,41145,35422,41995,246397,32968,33025,36
297,43710,36840,42544,32678,41802,40295,42987,37588,3355126,33462,32239,41296,35576,326166,31456,38291,42368,39655,40947,31394,40196,39558,42707,32656,32123,47877,32908,3772640,3772344,33270,36455,37140,39443,31664,33918,32000,3
8249,37272,43005,39417,42845,38257,37389,7354437,34134,43434,36564,34032,35254,32428,42218,33024,35258,33851,31374,32097,40955,43588,34751,31632,31187,37165,43312,37235,31992,33881,39537,33608,37411,37202,37837,37123,37995,42972,
39454,43569,7354420,2768948,38412,31926,40415,31347,39435,37336,40973,32869,31442,41441,36587,34080,39732,31666,37177,33971,44679,318909,246627,43442,35649,41800,32796,33981,53435,31802,47399,3771726,3772080,3772213,34358,57402
50,36310,37066,36288,36329,37840,37970,37038,43924,31329,41779,38765,31838,33512,5740557,7354430,44000,37080,42076,42863,40414,34179,41965,40217,32513,34192,41757,36674,42994,34967,31487,31006,31957,38812,43351,37199,40461,36278,
35195,40987,43385,31129,43787,40530,37230,37993,43158,37443,31762,34281,35922,38327,34896,318207,44851,37841,33710,3772221,3772232,34655,33778,42811,34540,31257,38230,41540,31663,246596,32556,40542,33904,37790,35779,40063,38372,
39594,33906,35512,43476,38078,41350,261329,35306,43944,39311,40850,36791,43826,50050,43284,40835,32467,43736,36058,37801,36170,42484,32474,39955,42862,38160,2768852,42284,36308,34225,41714,37638,39198,43218,42435,41798,33247,429
50,41353,32429,37583,35265,31353,36726,39725,31760,37434,192508,39338,49424,49297,34425,38427,43157,35278,35595,40292,7354428,36571,47283,49638,31555,41072,40448,38254,43836,36233,37292,41852,43377,43633,33962,39651,33441,53560,4
1107,34757,40848,34142,33014,2768848,35790,32725,43415,35325,41082,3355010,36238,40673,5740668,35387,38218,41102,326251,33554,42778,43044,32846,37360,36678,42616,35135,35031,43873,32663,40434,246538,33854,40421,117365,38661,3157
8,34363,42824,40529,38028,38512,43928,36913,33974,33423,37552,40456,31170,43864,38971,5740513,246485,31255,39716,42363,32741,317987,37787,45325,31031,34394,41096,35759,44391,43291,33003,36521,37893,53565,31803,31711,37755,36621,3
5690,42795,36431,32318,32207,32167,32143,45961,42664,44324,35130,31846,44027,46069,32557,41599,50417,3771890,39962,40940,43785,43501,36130,37215,39834,39910,38468,40331,39031,5740131,38932,34829,36506,34735,41910,39429,41312,369
93,43586,38757,40229,34796,39849,42447,39136,42971,39846,41059,37254,34642,39274,34987,35263,3346209,39744,39102,35610,40962,39145,246512,34970,31243,34139,37731,48387,48228,31379,43332,318828,40351,43330,318568,31290,35385,3274
5,38146,326172,35693,36419,39670,34780,39864,36774,42437,40301,42696,43654,192506,37562,35359,33202,31646,32263,38301,31154,39845,37247,39160,37850,42047,34842,37851,38216,33628,37580,45233,42048,40179,34099,45775,40696,35054,437
23,38984,37142,42596,32797,39402,36259,32083,31958,3772305,3772101,50472,37977,39431,35685,43799,39956,43039,42879,36989,34292,33306,33619,39901,251610,43737,317826,32623,42793,41524,33300,42753,35173,34487,34361,36158,35893,353
26,35323,33528,34148,34000,36896,35523,33477,33148,43912,34149,32734,44052,42671,32455,41151,32213,32582,33001,38652,59216,35295,53581,36614,32664,39405,40327,36365,31050,40290,42256,42591,43000,36769,41479,37792,33107,43741,3982
7,5740203,44132,33414,38168,41891,41025,41952,38821,39433,33081,43791,35933,33573,33681,31554,46155,35574,35696,32948,36554,41470,37081,37143,31669,43289,37523,42889,33859,41079,39035,31357,34413,32939,43441,32877,31229,49816,400
90,44205,42984,32666,35654,38580,35026,33903,42145,35305,32907,32078,33098,42736,40514,36463,3355144,35801,42867,31072,33819,38304,32470,41346,34614,37298,39523,38077,37239,36460,40094,31201,38459,37636,38422,39644,39855,35213,47
900,36015,3771934,3772186,38660,42345,41968,50082,35151,37778,39142,33772,33218,31422,31423,43402,42199,32855,38391,318890,31349,3772539,35184,40371,46156,42240,318211,40382,40093,36539,33203,42322,34876,34350,43079,40675,36373,
3885567,45959,39385,326184,326155,32602,31624,31623,35743,50459,39493,42963,33443,31254,42761,34928,3355131,39173,43132,39569,36005,2768883,33492,40250,33493,40546,42237,5740435,41397,39616,31261,41589,36263,43531,31151,43331,34
955,39540,38859,42029,33468,38099,37810,39168,40813,7354465,40201,41867,43352,36964,43484,40047,39226,34153,32740,44915,38223,33824,43513,40424,31579,36569,32316,40698,39697,40133,318059,34325,31527,33898,318164,35101,37289,3122
0,34293,2768886,36361,41416,38904,33837,38793,40225,34696,40708,32668,39159,34160,32757,38415,41200,40846,36795,35939,33394,32153,32889,36653,45382,34956,41208,48224,44115,36277,34980,41566,42034,32787,38341,41310,117331,44496,34
157,53443,33658,39583,42680,42171,36854,42379,42638,33185,44087,36176,38385,37948,40395,35731,37922,42734,41020,34632,37798,42896,30971,43333,36142,34844,39018,38568,42875,48311,33769,43266,42411,42853,326237,7354424,36673,37322,
43541,31849,317860,326157,39904,31000,39103,34665,42446,39032,32141,246578,37156,34388,38898,36609,40032,32993,34719,39350,40439,33162,41372,37176,43938,45280,42995,32015,42253,5740528,34765,32690,246513,43554,326142,32195,38444
,37838,31332,43709,42524,39763,41432,32257,40188,40628,40782,41925,37517,42861,38630,40924,36200,35578,34742,39866,34333,35979,32378,31773,40647,39459,33944,42682,34569,37959,33165,31872,40569,31759,39266,45757,41756,35596,39687,
34002,38748,32233,38752,38881,37774,59253,42982,43816,3355127,36808,41773,48450,32493,33555,42822,34313,36465,45881,42825,36087,31620,38966,39090,31902,39309,39747,36286,41375,33258,44446,41166,33505,31600,36491,32524,41585,4027
8,36242,38937,36406,36421,33687,43016,41913,32459,34098,36561,33035,47730,5740526,46066,5740845,34431,37603,37480,43698,36262,32769,42327,39474,36855,42434,42444,46068,34058,32852,33495,319033,326252,43278,41373,37356,35329,4425
1,33460,37852,35733,41230,43841,38811,39889,35028,37395,45278,36992,39214,39505,33795,36953,35749,43386,40623,35170,32947,42848,39453,32530,32069,40972,35758,35418,35475,117368,41627,42945,32221,39174,41123,40055,35796,36565,4407
1,42115,36445,38287,34526,41286,326210,35421,31192,33232,31630,32049,35843,42517,40173,42898,40657,39376,38214,42874,38645,32627,44150,44900,31014,31617,31003,35679,40091,35279,252554,32019,38153,3772179,3772427,39143,34910,3430
9,40814,41843,32588,38541,33513,33048,47141,42103,35223,34551,38142,42130,31712,41295,42142,38565,32492,317823,42140,33500,36339,34564,33062,40630,32746,35314,31680,38246,38726,38909,7354472,39475,37843,35667,246656,50002,42952,4
2392,37857,43162,42979,36241,40870,40528,36440,33180,42804,42453,34402,33682,38231,38027,35771,326168,41602,31602,34984,43439,42849,33295,42601,32070,41089,42819,33488,32920,40458,33758,39244,3772655,3772367,43229,41997,32838,35
584,33676,42182,43824,5740252,44086,35504,36771,36675,40462,31800,43012,35056,41830,38755,44111,40680,36319,43771,39460,36703,31649,34954,37876,34367,40616,33171,39988,36264,39980,43974,36405,39456,36033,33370,31004,36824,35051,3
9477,37542,43350,32017,38886,36461,44226,34427,38396,7354398,34619,40847,38154,47251,42906,5740389,35129,251466,37631,37869,41943,32793,39424,32616,36049,39151,43387,35977,31015,37148,7354425,40993,31470,31517,39436,39313,41833,
35816,40560,35225,34063,35577,31607,35742,45821,43749,33930,37337,32132,43327,33770,33897,37214,34314,37403,38026,38207,32687,32125,31722,39367,35765,34370,31248,44261,37549,34341,35552,2768944,41152,34177,41349,44736,31911,3639
0,39653,5740396,3772671,37559,42808,33852,35246,33262,31146,31466,40150,32567,44359,49045,41038,53557,40676,33288,36462,38519,38824,32871,34721,47746,38916,32540,31137,43822,42478,36122,37823,42656,38521,44668,38644,36999,43566,3
1105,32490,41360,44317,31179,7354434,39365,38198,31799,318785,41055,39195,33489,261613,41719,31256,43692,45826,33725,41679,40563,31533,42904,35335,53561,34377,33176,41872,32561,35241,40140,35780,34326,39258,31118,41738,53566,374
18,32533,37573,40269,2768929,38452,40170,64875,40141,42204,37579,42854,5740593,42286,41227,42841,38491,39811,117294,32371,32511,31458,38244,40639,39425,41959,40396,40248,192535,32565,31890,33444,31027,38365,35783,43549,43650,500

70,42738,3346177,39241,41666,40157,39428,37082,40412,43294,3346206,42369,36425,32042,34582,36966,37617,41670,39501,34280,40046,32751,32464,32743,37515,44021,36377,39091,44207,44118,43462,39922,38863,35998,43449,34411,39222,42759,
39485,34968,37639,44039,31707,41367,34745,38347,35445,34086,37382,39368,41587,34957,40656,36043,34835,38795,42025,43661,32056,35327,48309,53563,32818,34976,40043,33968,39502,43829,41531,40672,49168,39753,39555,42082,37557,33685,3
9403,40447,42249,41273,38815,34136,37961,43288,40707,39107,40949,44235,41861,32021,33864,39881,40527,36444,43728,41187,47249,37121,33863,34554,36775,31354,43798,39776,37179,34613,38045,326215,35089,42431,36409,41834,34886,38204,3
9089,36584,49804,36718,42826,44839,34384,32930,326150,38762,37233,40952,38891,42743,43560,33108,32765,33986,43083,39857,34686,34012,40697,34875,53554,40678,38082,252671,33721,42649,35239,31481,42695,34484,35289,41219,31450,34330,
31404,31662,42438,34883,43839,41611,33922,42618,38205,39121,42809,43568,49779,36270,34102,40299,37775,38912,35168,35864,42946,38799,32875,33843,43770,41270,38090,34066,35237,35614,42933,38288,37461,38717,34658,39322,35648,37962,4
1392,38399,41094,40261,39810,38156,34486,326152,39520,37204,32639,36336,38094,40233,31075,42063,37866,41615,40143,34353,326153,31740,41648,46121,39884,35701,40079,35846,42057,36658,38668,33261,32122,37965,42838,35330,43426,33514,
39970,41543,35997,35287,33178,3355139,32603,33213,32630,36382,43740,32543,35099,43832,36201,40684,37146,3355145,35119,43080,32168,43091,32536,44072,33374,39447,42056,37528,42091,38388,36340,44274,31519,33536,33486,32517,32394,31
355,34788,33392,32853,34166,34793,43216,38897,35897,31566,318983,40554,44642,41140,42957,35169,32617,32024,42306,35112,38137,38817,40815,32922,41316,31629,36387,31393,31149,38914,37358,43574,42872,34187,42942,50391,46015,40432,45
830,41737,41973,31565,35813,44409,41209,33709,43196,35150,40259,36903,2768836,42661,42869,34999,49070,38021,32301,41565,32095,38974,39169,318236,50105,35686,35207,31703,36018,40733,41457,40793,38032,42184,37750,40607,33507,38798,
35235,42928,33211,35492,38145,2768865,3885565,33044,34593,34888,34656,42158,39374,35384,41265,53546,39420,32442,35851,37447,33445,326262,43548,

A: List of all genes whose expression was rescued or partially rescued in TBPH/dTDP43 mutants by GAL4/UAS expression of TBPH under control of the endogenous promoter. “Conserved”: sublist of orthologs of mammalian TDP targets. B: List of differentially expressed genes in TBPH overexpression experiment. C: Pooled list of control genes from A1 and D42-GAL4>UAS-LacZ expressing strains.

Table S5 Analysis of splice-junction reads in mutant *vs.* control

gene	exon junction	physical map	A1	G2	rescue	D42> LacZ	D42> TBPH	*p* all junctions	*p* alternate exon	
cac	12/13	11,848,314..50,954				4	2			\
	12/15	11,848,314..56,052				4	**0**	0.022		/
	25/26	11,867,517..8,256	4	**2**	3	2	3	0.579		\
	27/28	11,869,894..70,435	11	**3**	14	10	10	0.076	0.163	/
	31/33	11,871,595..3,657	0	**0**	1	2	3	0.183		\
	32/33	11,873,188..3,657	7	**0**	3	4	2	0.033	NA	/
Pde9	1/2	12685781..874	2	**0**	1	1	2	0.15		\
	1/9	12,685,781..723,742	10	**0**	2	4	6	0.009	NA	/
	2/3	12,686,996..7,609	0	**3**	0	0	0	0.001		\
	2/9	12,686,996..723,742	14	**6**	5	4	6	0.4	0.001	/
Unc-115a	8/9	5,523,483..629	1	1	6	1	1			\
	8/10	5,523,484..4,039	0	**5**	0	0	0	0.002	0	/
smi35a	2/3	14,187,153..8,863	0	**1**	0	0	0		0.026	\
	2/13	14,187,153..233,893	10	**3**	3	5	3	0.053		/
sno	2/3	13,090,543..629	0	**3**	0	0	0		0.001	\
	2/16	13,090,543..105,132	2	0	2	0	1			/
rok	2/3	16,520,280..602	6	3	2	1	4			\
	2/11	..30,460	1	**0**	0	0	0		NS	/
Pkn	2/3		4	**1**	1	3	0			\
	2/13		1	**1**	1	1	4			/
	2/16		21	**3**	4	4	9	0.009		/
	2/21		2	**1**	0	1	1			/
CG6509	2/3	11,509,403..499	0	**1**	0	0	0		0.006	\
	2/12	11,509,403..16,508	4	**0**	2	0	4	0.024		/
NetB	9/10	14,642,954..3,380				6	**22**	0.040		
InR	13/14	17,413,731..5,692	13	**1**	2	9	15	0.008		\
	13/16	17,413,731..28,737	1	0	0	0	0			/
CG34318	4/5					6	**0**	0.003		
CG31379	3/4	16,827,889..28,051	1	**4**	0	0	0	0.017	0.002	\
	3/8	16,827,889..30,523	19	**3**	8	19	19	0.007		/
Ark	3/4		9	**0**	3	4	4		0.012	\
	3/7		4	3	9	9	**1**		0.029	/
CkIIbeta	2/3	11,687,551..830	0	**18**	0	0	0	0.000	0.000	\

	2/9	11,687,551..94,025	6	3	2	5	4			/
Trn	11/12	6,190,657..713	14	**3**	10	6	**13**	0.001,0.018		
ham	5/6	18,776,374..471	0	**5**	0	2	0	0.006	0.000	\
	5/7	18,776,374..9,804	**4**	0	0	1	0	0.009		/

Terms and abbreviations: gene, the flybase gene symbol for the corresponding splice target, Exon-junction, reference to the numerical order of exons from left to right as they appear in the genomic sequence regardless of orientation, Physical map, the breakpoints of the alternatively spliced exons, A1 number of reads crossing the junction in control (A1) genotype, G2 number of reads *etc*. in mutant (G2) genotype, rescue, number of reads in rescue (G2-/-; TBPH-GAL4>UAS-TBPH), D42>LacZ, number of reads in D42-GAL4>UAS-LacZ controls, D42>TBPH, number of reads in D42-GAL4>UAS-TBPH overexpression, *p* all junctions, the result of a statistical test using all junction reads (not shown) from the involved gene to normalize (non-inclusive, see methods), *p* alternative exon, similar statistical test using the alternative junction reads as a normalizer (see also methods). The *p* value refers to the boldfaced, underlined junction read-count in the body of the table. If more than one *p* value is listed, reads were significant for the indicated junction in two genotypes and the p values are listed in the same order as the column order of the boldfaced and underlined genotypes. The brackets at right indicate alternative exon-junction pairs from within the same gene.

Table S6 Differentially expressed genes with homology to human neurological disease association

Gene	Dataset	Direction	Ratio	Ortholog	e_value	rev e value	disease
Ddc	KO	Down	3.52	DDC	5.00E-175	8.00E-175	Aromatic L-amino acid decarboxylase deficiency
Tequila	OX	Down	1.86	PRSS12	5.00E-72	3.00E-72	Mental retardation
CG12728	KO	Up	1.57	NHEJ1	0.0008	0.0002	Severe combined immunodeficiency with microcephaly
CG10249	KO, OX	Up,Dn	1.56,1.3	KANK1	1.00E-67	2.00E-67	Cerebral palsy
Myo10A	OX	Down	1.54	MYO15A	0	0	Deafness
Tau	KO, OX	Up,Dn	1.45,1.21	MAPT	1.00E-24	1.00E-24	Dementia
CG3376	OX	Down	1.37	SMPD1	2.00E-120	3.00E-120	Niemann-Pick disease
CG14291	OX	Up	1.36	SGSH	3.00E-133	4.00E-133	Sanfilippo syndrome
mre11	OX	Down	1.33	MRE11A	1.00E-118	2.00E-118	Ataxia-telangiectasia-like disorder
Sh	KO	Up	1.29	KCNA1	5.00E-149	7.00E-149	Episodic ataxia/myokymia syndrome
Tsp3A	OX	Down	1.26	TSPAN7	6.00E-20	1.00E-23	Mental retardation
Spred	OX	Down	1.23	SPRED1	2.00E-19	2.00E-19	Neurofibromatosis
Htt	KO_SPLICE	Up	1.21	HTT	8.00E-20	1.00E-19	Huntington disease (3)
Mer	KO	Up	1.19	NF2	6.00E-148	7.00E-148	Meningioma
dlg1	KO	Down	1.18	DLG3	0	0	Mental retardation
CG3822	OX	Down	1.16	GRIK2	0	0	Mental retardation
Rst	OX	Down	1.16	KIRREL3	8.00E-63	4.00E-63	Mental retardation
Cac	KO_SPLICE, OX_SPLICE	Up,Dn	1.15,1.1	CACNA1S	0	0	Hypokalemic periodic paralysis
Sd	OX	Up	1.13	TEAD1	3.00E-128	1.00E-128	Sveinsson choreoretinal atrophy
Taf1	KO	Down	1.11	TAF1	0	0	Dystonia-Parkinsonism
Cap	OX	Up	1.08	SMC3	0	0	Cornelia de Lange syndrome 3
Marf	OX	Down	1.08	MFN2	0	0	Charcot-Marie-Tooth disease
CG31739	OX_SPLICE	Up	1.02	DARS2	1.00E-151	1.00E-151	Leukoencephalopathy with brainstem and spinal cord involvement and lactate elevation

Explanation of terms: In the column marked Dataset, KO refers to the differentially expressed genes in TBPH G2 mutant vs. control, OX to D42>TBPH vs. D42>LacZ control. KO_SPLICE and OX_SPLICE refer to differentially spliced genes in G2 vs. control and D42>TBPH vs. control, respectively. The direction and magnitude of change relative to control are given in the Direction and Ratio columns. The Human gene column is the proposed homolog/ortholog followed by its BLAST e-value (obtained from homophila, see text) and reverse blast e-value. The final column gives the human gene's disease associations.
