# Supplementary material for: Comparison of Parallel High-Throughput RNA Sequencing Between Knockout of TDP-43 and Its Overexpression Reveals Primarily Nonreciprocal and Nonoverlapping Gene Expression Changes in the Central Nervous System of Drosophila
Source: G3 (Bethesda). 2012 Jul 1;2(7):789–802. doi: 10.1534/g3.112.002998 (PMC3385985; doi:10.1534/g3.112.002998)
Supplement: Supporting Information [file supp_2.7.789_FigureS1.pdf]

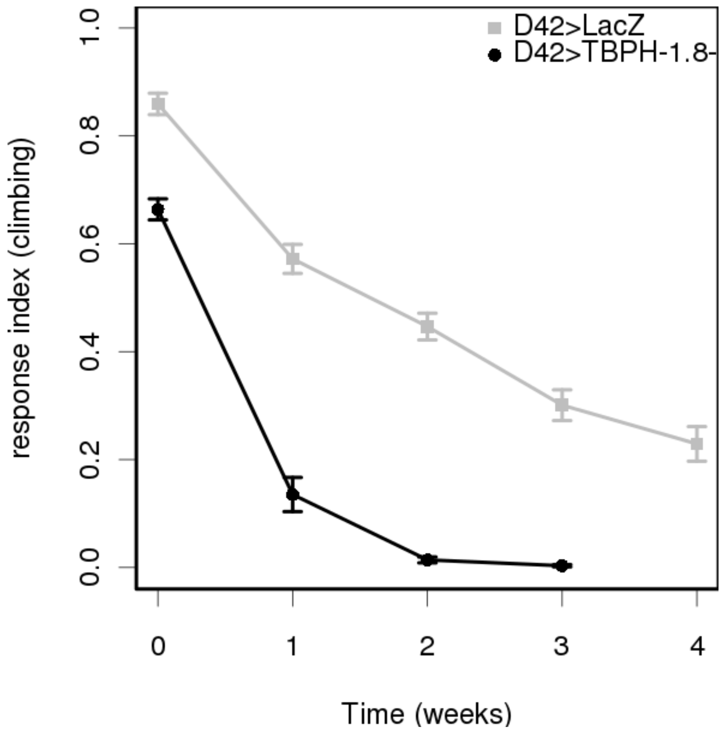

Figure S1 Overexpression of TBPH in motor-neurons causes adult climbing deficits. In the current study, these two genotypes, D42>LacZ (grey squares) and D42>TBPH (black circles) were compared to assess the effect of overexpression of TBPH in motorneurons. At each time point assayed, TBPH expression caused a significantly reduced ability to climb compared to controls ($p<0.01$) Climbing assays were performed as described in (Benzer, 1967).
