# Supplementary material for: Comparison of Parallel High-Throughput RNA Sequencing Between Knockout of TDP-43 and Its Overexpression Reveals Primarily Nonreciprocal and Nonoverlapping Gene Expression Changes in the Central Nervous System of Drosophila
Source: G3 (Bethesda). 2012 Jul 1;2(7):789–802. doi: 10.1534/g3.112.002998 (PMC3385985; doi:10.1534/g3.112.002998)
Supplement: Supporting Information [file supp_2.7.789_TableS1.pdf]

Table S1 Gene Expression changes in G2 mutants, rescued genes only.

Flybase_ID	Entrez_ID	A1	G2	Ratio	Direction	p_value	adj_P_Val
Hsp70Aa	48581	-0.62	4.02	25.06	Up	0.047647	NA
Mur18B	32912	-1.82	2.29	17.30	Up	0.00162	8.28E-10
CG33191	251270	-1.82	1.78	12.16	Up	0.004289	NA
CG42393	7354474	-0.82	2.78	12.14	Up	0.006049	1.93E-06
CG6372	38986	-1.29	2.03	10.00	Up	0.046938	3.25E-13
CG3984	41821	5.00	1.97	8.17	Down	0.006599	5.05E-22
CG7084	42606	-0.10	2.86	7.75	Up	0.01693	7.90E-10
CG6912	41820	4.73	1.86	7.33	Down	0.009779	5.93E-28
CG31663	33363	6.25	3.73	5.73	Down	0.000233	1.87E-58
Ude	42953	-0.52	1.93	5.47	Up	0.041034	NA
CG12496	31226	0.72	2.80	4.20	Up	0.040112	0.038228
CG31115	318597	2.32	0.32	4.00	Down	0.034834	0.001154
mwh	38131	0.15	2.11	3.89	Up	0.011536	0.000107
stops	43683	2.71	0.81	3.74	Down	0.020156	1.58E-05
Cyp12c1	40037	0.68	2.58	3.73	Up	0.008916	0.000168
Ddc	35190	6.02	4.20	3.52	Down	8.03E-05	1.08E-26
CG32204	317913	5.18	6.86	3.20	Up	0.000261	2.76E-26
Ance-4	35909	0.71	2.37	3.17	Up	0.049128	0.003194
CG12355	2768954	1.20	2.84	3.12	Up	0.040599	0.101571
CG1924	32180	1.28	2.91	3.09	Up	0.022844	NA
CG4546	41922	0.72	2.30	2.99	Up	0.018114	0.000446
Rpt6R	43635	0.48	2.06	2.98	Up	0.036028	0.038104
CG9812	37709	1.79	3.35	2.96	Up	0.00975	0.000145
CG10869	33337	0.48	2.04	2.94	Up	0.015332	0.010392
CG5491	43227	3.39	4.89	2.83	Up	0.000506	4.47E-12
CG6678	42581	2.75	1.27	2.79	Down	0.037883	0.096767
CG32195	317907	2.66	4.09	2.71	Up	0.006231	2.63E-08
Obp99a	43488	8.83	7.45	2.61	Down	9.84E-05	4.35E-31
lbl	42541	0.90	2.24	2.53	Up	0.020222	0.043097
dro5	38409	2.53	3.83	2.48	Up	0.038687	0.000212
CG1681	32299	2.48	3.77	2.45	Up	0.005266	7.21E-05
CG4839	34348	0.79	2.05	2.40	Up	0.000429	0.236896
CG7800	40963	2.39	3.62	2.34	Up	0.024575	0.000277
CG32495	318053	5.77	4.55	2.33	Down	0.022823	NA
plx	40703	3.64	2.43	2.32	Down	0.013398	1.84E-08
FucTB	34260	1.84	0.63	2.31	Down	0.044129	NA
CG5704	48613	2.13	0.98	2.22	Down	0.022554	0.168479
asparagine-synthetase	2768965	5.34	6.47	2.19	Up	6.04E-05	9.41E-12
GS	32775	6.04	4.93	2.15	Down	0.002856	NA

CG4210	41832	1.87	0.77	2.14	Down	0.009741	NA
CG14082	40088	3.62	2.54	2.12	Down	0.009672	0.00206
MtnA	41202	5.93	4.87	2.09	Down	0.033243	1.46E-10
hkb	40549	2.42	3.48	2.09	Up	0.025104	0.041523
Fuca	3772574	4.59	5.65	2.09	Up	0.011602	1.74E-08
CG11714	3772566	4.59	5.65	2.09	Up	0.011602	NA
stv	39518	3.83	4.89	2.08	Up	0.020985	2.71E-06
RpS5b	41807	3.39	4.42	2.04	Up	0.019594	2.59E-05
CG32442	318031	3.49	2.46	2.04	Down	0.023831	0.018108
GstD3	48336	4.00	2.98	2.03	Down	0.020253	0.000432
CG12994	50350	1.66	2.67	2.01	Up	0.028956	0.23397
CG4660	31542	3.70	4.70	1.99	Up	0.002318	6.40E-05
lama	38610	5.79	4.81	1.98	Down	0.000906	1.09E-09
CG12003	38183	2.47	1.49	1.97	Down	0.041798	0.096767
CG42640	44513	2.23	1.27	1.95	Down	0.036189	0.110598
CG7386	38719	2.23	3.20	1.95	Up	0.017625	0.055547
CG32845	318243	1.16	2.13	1.95	Up	0.044242	NA
mRpS11	42061	2.63	3.59	1.94	Up	0.032741	0.088316
CG10425	43043	3.38	4.29	1.89	Up	0.038887	0.023524
CG13142	34434	1.66	2.56	1.87	Up	0.047215	NA
CG32857	318252	3.12	4.02	1.87	Up	0.014244	NA
ry	41605	2.91	3.81	1.86	Up	0.001791	0.085004
Skeletor	3771796	3.97	3.08	1.85	Down	0.01303	NA
Skeletor	3772559	3.97	3.08	1.85	Down	0.01303	NA
CG8132	41121	4.00	3.11	1.85	Down	0.010685	0.000117
Sox21a	39567	2.27	3.14	1.83	Up	0.014648	0.226839
CG13897	38090	4.70	5.52	1.76	Up	0.005451	4.57E-05
Sp7	40918	4.48	5.26	1.72	Up	0.003129	0.001035
Cyp6v1	33056	4.32	3.54	1.71	Down	0.037145	0.000839
mRpS18A	326141	3.59	4.37	1.71	Up	0.009219	0.03304
CG8928	32558	2.91	3.68	1.70	Up	0.023425	0.224879
CG7607	39263	3.26	4.03	1.70	Up	0.021684	0.001233
CG1545	32036	2.98	3.75	1.70	Up	0.022481	0.185298
rtv	32056	1.97	1.21	1.69	Down	0.02246	NA
CG32687	31980	2.58	3.33	1.68	Up	0.047176	0.116604
CG14210	32958	3.40	4.15	1.68	Up	0.008163	0.321152
CG11562	33200	2.61	3.36	1.67	Up	0.004382	0.204857
wgn	32849	3.95	4.69	1.67	Up	0.001873	0.002382
plx	40704	7.39	6.66	1.65	Down	0.024184	1.84E-08
CG5793	42505	2.78	3.49	1.64	Up	0.039913	0.138717
NFAT	32321	6.80	6.09	1.64	Down	0.005507	4.63E-07
CG15822	38273	3.86	3.15	1.63	Down	0.007874	0.009904

CG34325	32656	3.93	4.64	1.63	Up	0.001944	0.118087
B-H1	32724	3.95	3.25	1.63	Down	0.03526	0.039318
Fibp	40163	3.17	3.87	1.63	Up	0.042017	0.331298
CG4484	39055	4.72	4.02	1.62	Down	0.006238	0.000303
CG12582	40524	5.05	5.75	1.62	Up	0.00123	0.000821
CG1265	38517	3.24	2.54	1.62	Down	0.022864	0.065374
CG9095	32447	1.67	2.36	1.61	Up	0.004679	NA
CG8038	38889	3.68	3.00	1.61	Down	0.000686	0.074307
CG33786	3772640	4.30	4.98	1.61	Up	0.034986	NA
CG33785	3772344	4.30	4.98	1.61	Up	0.034986	NA
CG10898	41384	2.75	3.43	1.61	Up	0.047605	0.085004
mTerf3	40279	2.84	3.52	1.60	Up	0.022521	0.115059
wun2	53558	4.32	4.99	1.59	Up	0.001048	0.012494
ventrally-expressed-protein-D	117331	2.50	1.84	1.59	Down	0.019978	NA
CG42235	43136	4.94	5.60	1.58	Up	0.031781	0.001678
mRpL33	50381	2.21	2.87	1.57	Up	0.015111	NA
CG12728	31526	3.38	4.03	1.57	Up	0.033918	NA
Ac76E	40180	3.65	4.29	1.56	Up	0.010835	0.012359
CG5973	34023	1.57	2.21	1.56	Up	0.034923	NA
CG30392	246587	3.46	4.10	1.56	Up	0.018509	NA
CG31547	40663	6.85	7.50	1.56	Up	0.004023	0.000924
CG3308	42527	4.90	5.54	1.56	Up	0.03303	0.009532
Mdr49	36428	4.14	4.78	1.56	Up	0.01267	0.101641
Tsp42Ej	35620	4.07	4.70	1.55	Up	0.035375	0.296582
DNApol-eta	40438	3.72	4.34	1.54	Up	0.034143	0.24497
CG17292	34152	2.66	3.28	1.54	Up	0.033903	NA
CG33502	2768875	3.47	4.09	1.53	Up	0.000915	NA
CG12177	32326	3.07	3.68	1.53	Up	0.014878	0.100583
CG8326	32738	3.78	4.39	1.53	Up	0.024056	0.11879
CG9114	32460	4.17	4.78	1.52	Up	0.038583	0.033143
UGP	39065	4.73	5.33	1.52	Up	0.046125	0.009532
CG32432	40310	6.07	5.47	1.52	Down	0.013973	0.000259
Scp2	42015	2.76	2.16	1.51	Down	0.016838	NA
CG12746	40672	3.97	4.57	1.51	Up	0.025353	0.055679
CG11577	40084	5.20	5.79	1.51	Up	0.006348	0.014408
CG1927	38262	4.54	5.14	1.51	Up	0.004388	0.151389
Rh50	38589	3.76	4.35	1.51	Up	0.040207	0.006029
spn-A	43577	4.13	3.54	1.50	Down	0.049609	0.159309
CG16863	34784	3.52	2.94	1.50	Down	0.003112	0.155887
Ada1-2	318992	3.34	3.92	1.50	Up	0.017295	NA
xmas-1	44660	3.80	4.37	1.49	Up	0.018407	0.116528

TBPH	37781	6.97	6.41	1.48	Down	0.024987	1.52E-05
Ir62a	3885628	2.52	1.96	1.47	Down	0.031439	NA
CG32281	317953	3.30	3.86	1.47	Up	0.049016	0.265832
Ilp2	39150	5.48	4.92	1.47	Down	0.03675	0.001233
Cdk7	31441	4.64	5.19	1.47	Up	0.008875	0.08987
CHMP2B	38599	3.68	4.24	1.47	Up	0.043362	0.224078
CG9896	37652	2.47	3.02	1.46	Up	0.006961	NA
CG6171	41872	4.95	5.50	1.46	Up	0.004081	0.24497
RpI135	33210	4.99	5.54	1.46	Up	0.017992	0.033508
CG4984	37017	4.37	4.92	1.46	Up	0.033847	NA
PHDP	37788	1.61	2.15	1.46	Up	0.0264	NA
Syx13	39485	5.37	4.84	1.45	Down	0.012964	0.081678
run	33059	5.58	6.11	1.45	Up	2.08E-05	0.021595
CG32786	318210	3.49	4.02	1.45	Up	0.021204	NA
CG32372	38845	6.22	6.75	1.45	Up	0.029087	0.009431
neb	35293	5.09	5.63	1.45	Up	0.007342	0.02346
CG10949	35310	4.03	4.56	1.44	Up	0.012155	0.194738
CG18210	32469	4.27	3.75	1.44	Down	0.027657	0.03304
dpr13	3885598	4.53	5.06	1.44	Up	0.006411	0.210277
Hsp83	38389	11.29	11.82	1.44	Up	0.03774	0.000277
CG34106	4379891	2.33	2.85	1.44	Up	0.019715	NA
CG42668	42411	3.58	3.06	1.43	Down	0.030606	NA
CG32549	32822	6.03	6.55	1.43	Up	0.019216	0.046914
CG6044	37558	4.24	3.72	1.43	Down	0.006123	0.06999
form3	3346238	5.17	5.68	1.43	Up	0.003591	0.072992
CG34163	5740407	1.72	2.24	1.43	Up	0.036284	NA
f-cup	41677	3.91	4.42	1.43	Up	0.019635	0.339684
ECSIT	40732	3.83	4.34	1.43	Up	0.029851	0.221934
RNaseX25	38885	4.86	5.36	1.42	Up	0.020624	0.047892
Hr78	40378	5.95	6.45	1.42	Up	0.020004	0.028848
CG11910	43074	6.79	6.29	1.41	Down	0.011207	0.00198
CG12182	38324	4.00	4.50	1.41	Up	0.029207	0.296582
CG8315	36758	3.74	4.23	1.40	Up	0.023182	0.154832
Spc25	41585	3.77	4.25	1.40	Up	0.042351	NA
Amph	36383	4.41	4.88	1.39	Up	0.043932	0.275556
PGRP-LE	32534	3.85	4.32	1.39	Up	0.021957	0.299592
CG17739	36333	6.04	5.57	1.39	Down	0.01765	0.096767
stck	40999	5.16	5.63	1.39	Up	0.003959	NA
fog	33148	5.76	6.23	1.38	Up	0.003711	0.020909
CG10069	37427	5.20	5.67	1.38	Up	0.019111	0.08987
CG42399	33176	4.45	4.92	1.38	Up	0.045165	0.135094
chrb	39284	8.40	7.93	1.38	Down	0.006531	0.000302

CG1236	40708	5.09	5.55	1.38	Up	0.001213	0.19408
CG10702	35181	4.74	5.20	1.38	Up	0.004616	0.039449
CG15353	50191	5.16	4.70	1.38	Down	0.038835	0.001233
Timp	41248	2.59	3.05	1.37	Up	0.006019	NA
CG3735	37803	4.15	4.61	1.37	Up	0.032263	0.150456
CG17691	3355069	4.46	4.92	1.37	Up	0.039391	0.173441
Tsp66E	39017	5.74	6.20	1.37	Up	0.005103	0.104675
Ast-CC	34538	3.81	3.35	1.37	Down	0.025275	NA
Shawl	5740840	4.19	3.74	1.36	Down	0.022376	0.253844
sll	42115	4.86	5.31	1.36	Up	0.028613	0.104111
blow	35694	5.70	6.14	1.36	Up	0.004801	0.058488
CG9123	32462	5.17	5.61	1.36	Up	0.002958	0.237545
CG4400	32243	5.75	6.19	1.36	Up	0.009638	0.075906
CG5116	43081	4.65	5.09	1.36	Up	0.019542	0.081445
CG33214	40382	7.36	6.91	1.36	Down	0.013977	0.001006
CG12065	31798	6.94	7.38	1.36	Up	6.94E-05	0.03304
CG7772	32776	2.31	2.75	1.36	Up	0.034363	NA
CG5532	37761	5.17	4.73	1.36	Down	0.049171	0.013653
Mocs2	43017	3.62	4.06	1.36	Up	0.037099	NA
mirr	39441	5.94	6.37	1.35	Up	0.011402	0.039449
exo70	38959	4.80	5.23	1.35	Up	0.021261	0.289507
PCID2	39306	5.12	4.69	1.35	Down	0.005642	0.116084
CG33199	326329	5.51	5.94	1.35	Up	0.021231	NA
Atg5	31666	4.70	5.13	1.35	Up	0.038091	0.052119
CG8229	35898	5.51	5.94	1.35	Up	0.01926	0.008315
CG12576	33135	6.29	6.72	1.35	Up	0.000659	0.065903
scb	36692	5.94	6.37	1.34	Up	0.040915	0.022813
CG12104	38187	4.46	4.88	1.34	Up	0.023133	NA
CG17385	36603	5.90	6.33	1.34	Up	0.025258	NA
ATPsyn-Cf6	42759	6.27	5.85	1.34	Down	0.034014	0.170544
CG7277	33777	5.25	5.67	1.34	Up	0.00203	0.26852
NfI	43782	5.34	5.76	1.34	Up	0.008671	0.224607
Lerp	43223	5.40	5.82	1.34	Up	0.017676	0.132346
CG7582	43492	2.76	3.17	1.33	Up	0.030215	NA
Csl4	34548	3.47	3.88	1.33	Up	0.008374	NA
Klp3A	31240	6.71	6.30	1.33	Down	0.010035	0.016135
CG5021	39025	5.07	5.48	1.33	Up	0.004589	0.226839
CG2126	43748	3.35	3.76	1.33	Up	0.012453	NA
CG4098	39854	4.73	5.14	1.33	Up	0.022651	NA
CG5147	40002	5.29	4.88	1.33	Down	0.039857	0.048322
CG10508	40342	6.54	6.95	1.33	Up	0.014882	0.014425
Syx6	40373	5.32	5.73	1.33	Up	0.032081	

kat-60L1	40715	6.08	6.49	1.33	Up	0.013325	0.160573
Fer1	2768661	4.67	5.08	1.33	Up	0.010187	0.191272
CG15651	37375	4.87	5.27	1.32	Up	0.034791	NA
CG5590	43325	6.43	6.03	1.32	Down	0.005916	0.016695
Jra	36057	5.19	5.59	1.32	Up	0.030928	0.168479
VhaM9.7-b	40389	4.82	5.22	1.32	Up	0.047265	NA
CG34127	40912	5.09	5.49	1.32	Up	0.037106	0.20893
CG13108	34240	4.72	5.12	1.32	Up	0.013656	NA
SelR	41309	4.98	5.38	1.31	Up	0.01165	NA
Nep4	42449	7.41	7.01	1.31	Down	0.000923	0.00991
CG11180	37330	4.83	5.22	1.31	Up	0.014102	0.295161
CG3011	31524	6.37	6.76	1.31	Up	0.047205	0.135586
Spn6	49803	4.54	4.92	1.31	Up	0.045003	0.329516
CG8116	41035	3.81	3.43	1.31	Down	0.021762	NA
CG7188	38936	6.13	6.51	1.30	Up	0.029443	0.13033
CG6565	34749	4.97	5.35	1.30	Up	0.025928	0.26508
SIFR	42530	4.19	3.81	1.30	Down	0.026354	NA
CG16753	38365	3.91	4.29	1.30	Up	0.044282	NA
salr	34568	6.38	6.76	1.30	Up	0.021516	0.151237
CG8379	41060	5.23	5.61	1.30	Up	0.01499	NA
CG9531	33902	3.75	3.37	1.30	Down	0.021172	NA
cact	34969	6.25	6.63	1.30	Up	0.007955	0.29018
egl	37757	5.00	5.37	1.30	Up	0.044811	NA
CG3368	43292	6.85	7.22	1.30	Up	0.00078	0.204857
pit	42595	6.03	6.40	1.30	Up	0.0126	0.209738
CG5181	34018	5.43	5.06	1.30	Down	0.04082	0.116528
SAK	40384	5.33	5.70	1.29	Up	0.015908	NA
crim	39321	3.44	3.81	1.29	Up	0.021965	NA
dve	37546	5.96	6.33	1.29	Up	0.005206	0.233345
Eip71CD	39675	5.12	4.75	1.29	Down	0.013615	0.09237
Sh	32780	5.82	6.18	1.29	Up	0.042161	0.020026
wda	42750	4.30	4.66	1.29	Up	0.048334	NA
mRpL14	31222	4.13	4.49	1.29	Up	0.042329	NA
Tollo	44497	5.31	4.94	1.29	Down	0.007832	0.169634
dpr2	3346227	2.44	2.80	1.28	Up	0.010273	NA
magu	36048	5.05	4.69	1.28	Down	0.006403	NA
CG11317	43676	5.81	6.16	1.28	Up	0.012135	0.135288
CG7339	39310	4.35	4.70	1.28	Up	0.013541	NA
CG13248	40254	6.18	5.83	1.28	Down	0.031411	0.065502
Nelf-E	38982	4.70	5.05	1.28	Up	0.022342	0.350458
pain	37985	5.17	5.52	1.27	Up	0.001303	NA
CG10189	35234	3.33	3.68	1.27	Up	0.003837	NA

CanB	44317	6.85	7.20	1.27	Up	0.03154	0.288419
Pk61C	38017	7.66	7.31	1.27	Down	0.0238	0.009904
att-ORFA	42429	3.81	4.16	1.27	Up	0.020656	NA
CG7745	36210	4.52	4.86	1.27	Up	0.0468	NA
crl	44054	5.35	5.69	1.27	Up	0.018134	NA
CG31999	43777	4.89	4.55	1.27	Down	0.019124	0.058488
CG40045	3355079	6.54	6.88	1.27	Up	0.042339	NA
bbc	36496	6.75	7.09	1.26	Up	0.045139	0.224078
foi	38976	6.60	6.94	1.26	Up	0.003194	0.24497
CG6751	36150	5.97	6.31	1.26	Up	0.017457	0.204569
Fbxl4	32378	5.17	4.83	1.26	Down	0.035628	0.296298
CG12413	43382	3.76	3.42	1.26	Down	0.03864	NA
CG3253	37861	4.34	4.68	1.26	Up	0.031798	NA
Fdh	41311	7.08	7.41	1.26	Up	0.032958	0.182525
CG42458	2768945	4.19	3.85	1.26	Down	0.02702	NA
CG17574	36415	4.32	4.64	1.26	Up	0.041039	NA
CG34195	37018	2.70	3.03	1.26	Up	0.046011	NA
CG3726	31525	4.98	5.31	1.25	Up	0.037443	NA
CG1402	31687	4.32	4.65	1.25	Up	0.031937	NA
CG12822	35737	5.62	5.94	1.25	Up	0.014699	NA
Sulf1	53437	5.80	6.12	1.25	Up	0.016031	0.143232
CG31915	319025	4.58	4.89	1.25	Up	0.011425	NA
CG5288	39031	4.98	5.30	1.25	Up	0.034309	NA
Exn	39901	5.44	5.76	1.25	Up	0.009464	0.325554
CG11191	35765	5.57	5.88	1.24	Up	0.036687	NA
ldbr	38900	5.46	5.78	1.24	Up	0.040951	NA
Aats-cys	36784	5.68	6.00	1.24	Up	0.009876	NA
CG32528	32990	5.08	5.39	1.24	Up	0.048799	NA
Psi	36889	8.59	8.28	1.24	Down	0.018049	0.072881
Hydr2	33532	6.17	6.48	1.24	Up	0.037415	NA
REG	32274	7.23	7.54	1.24	Up	0.003733	0.331982
Scgalpha	34135	4.77	5.08	1.24	Up	0.008772	NA
CG34260	5740551	4.62	4.93	1.24	Up	0.023719	NA
CG12262	38864	6.58	6.27	1.24	Down	0.010739	0.045152
hd	40642	3.68	3.99	1.24	Up	0.027412	NA
CG9436	35586	4.11	4.41	1.24	Up	0.040524	NA
Syt14	40544	4.97	4.66	1.23	Down	0.038823	0.204857
CG10366	35258	4.71	5.01	1.23	Up	0.009626	NA
CG17754	31873	7.02	6.72	1.23	Down	0.009864	0.13033
CG5823	42105	5.42	5.72	1.23	Up	0.021641	0.248311
Hmr	31988	5.83	6.13	1.23	Up	0.018436	NA
CG42337	40335	4.52	4.81	1.23	Up	0.011569	0.273226

XRCC1	31451	4.83	5.12	1.23	Up	0.007842	0.311549
Hsc70-5	36583	7.54	7.83	1.23	Up	0.013538	NA
Lip4	34450	6.02	6.31	1.22	Up	0.024495	NA
Rala	31332	5.59	5.88	1.22	Up	0.036386	NA
Vps45	41153	4.33	4.62	1.22	Up	0.026277	NA
CG6712	34631	5.20	5.49	1.22	Up	0.037521	NA
Sdic1	43984	4.54	4.83	1.22	Up	0.016682	NA
ush	33225	5.51	5.79	1.22	Up	0.015133	NA
CG3560	32586	5.28	5.56	1.22	Up	0.012233	NA
CG1789	31812	4.25	4.53	1.22	Up	0.027686	NA
MED1	40403	7.85	7.57	1.22	Down	0.00734	0.178288
Arc1	36595	8.89	8.61	1.22	Down	0.047083	0.071523
nvy	37886	6.71	6.99	1.21	Up	0.016199	NA
CG6287	34554	6.07	6.35	1.21	Up	0.047581	NA
ergic53	44679	6.79	7.06	1.21	Up	0.033977	NA
Aats-lys	31904	7.72	8.00	1.21	Up	0.008459	NA
Clic	32349	6.14	6.41	1.21	Up	0.007097	NA
CG8602	38808	6.51	6.24	1.21	Down	0.04403	0.168858
CG5343	34429	4.78	5.05	1.21	Up	0.003521	NA
CG34371	37679	7.44	7.17	1.21	Down	0.019022	0.059006
Ggamma30A	45234	8.33	8.60	1.21	Up	0.00659	NA
CG13784	34003	5.94	6.21	1.20	Up	0.016125	NA
l(1)G0289	31964	7.20	7.47	1.20	Up	0.019807	NA
baz	32703	6.22	5.95	1.20	Down	0.003991	0.065272
klg	42707	7.51	7.78	1.20	Up	0.021615	NA
Aats-val	45783	6.91	7.18	1.20	Up	0.012694	NA
Rab26	40359	5.53	5.79	1.20	Up	0.001393	NA
CG9636	40967	7.15	7.42	1.20	Up	0.029257	0.347539
AP-1gamma	31842	7.05	7.31	1.20	Up	0.049497	NA
MAPk-Ak2	44573	6.49	6.75	1.20	Up	0.027776	NA
CG11396	40296	4.48	4.22	1.20	Down	0.042361	NA
MED11	40042	4.84	4.58	1.20	Down	0.011481	0.236896
NPFR1	40754	3.84	4.10	1.20	Up	0.003066	NA
Gbeta5	31744	5.58	5.83	1.19	Up	0.015182	NA
RpS14a	47218	9.39	9.65	1.19	Up	0.040083	0.317467
geminin	35563	6.30	6.55	1.19	Up	0.015139	NA
Mer	32979	4.83	5.08	1.19	Up	0.001263	NA
grau	45871	4.99	5.24	1.19	Up	0.005611	NA
CG1637	32019	6.27	6.52	1.19	Up	0.02527	NA
GalNAc-T2	32836	6.26	6.52	1.19	Up	0.037906	NA
CG15618	33001	6.13	5.88	1.19	Down	0.02534	0.292735
p	41025	6.81	7.06	1.19	Up	0.004703	NA

CG11926	33689	4.85	4.60	1.19	Down	0.024696	NA
CAP-D2	43491	7.16	6.92	1.19	Down	0.048453	NA
CG5214	41360	7.47	7.22	1.19	Down	0.035739	0.104912
CG12592	41263	5.93	6.18	1.18	Up	0.035484	0.067867
7B2	40644	7.40	7.64	1.18	Up	0.022045	NA
CG8378	36299	6.57	6.81	1.18	Up	0.014082	NA
CG12239	31509	7.65	7.41	1.18	Down	0.026647	0.116528
Hnf4	44544	3.97	3.73	1.18	Down	0.009187	NA
CG32226	40229	7.51	7.27	1.18	Down	0.005332	0.189433
CG4577	33291	7.88	7.64	1.18	Down	0.008905	0.157082
HspB8	32955	6.44	6.68	1.18	Up	0.002243	NA
scf	38145	6.65	6.89	1.18	Up	0.00263	NA
dlg1	32083	8.02	7.78	1.18	Down	0.005125	0.182336
Sod2	36878	6.64	6.40	1.18	Down	0.035369	0.264135
CG6506	32766	4.20	3.96	1.18	Down	0.011712	NA
CG5871	42518	6.94	6.71	1.18	Down	0.02985	0.285009
CG14812	31137	3.76	3.99	1.18	Up	0.020684	NA
dome	32976	7.24	7.47	1.18	Up	0.018949	NA
RpS14b	47219	8.54	8.77	1.17	Up	0.001696	NA
CG3004	31903	6.15	5.92	1.17	Down	0.015639	NA
pgant2	33556	6.67	6.44	1.17	Down	0.024948	NA
CG6769	32770	5.52	5.75	1.17	Up	0.001558	NA
ferrochelatase	43757	5.04	5.27	1.17	Up	0.01943	NA
Adam	36037	5.74	5.97	1.17	Up	0.007047	NA
CG6512	39922	6.99	7.22	1.17	Up	0.025557	NA
xmas-2	44271	6.39	6.17	1.17	Down	0.000928	NA
Hop	33202	8.16	8.39	1.17	Up	0.036117	NA
Aats-thr	45784	8.20	8.43	1.17	Up	0.024972	NA
CG42390	42600	5.20	5.43	1.17	Up	0.000484	NA
CG8841	36336	5.74	5.96	1.17	Up	0.014236	NA
CG15646	32501	5.27	5.49	1.17	Up	0.009272	NA
CG15111	37200	5.84	6.07	1.17	Up	0.038562	NA
ham	35135	7.35	7.13	1.17	Down	0.042123	0.308393
CG9346	37381	6.01	5.79	1.17	Down	0.023752	NA
CG9253	35379	6.54	6.76	1.16	Up	0.0026	NA
Smox	31738	8.31	8.53	1.16	Up	0.044535	NA
CG31635	33928	6.49	6.71	1.16	Up	0.016871	NA
CG13995	33851	5.87	6.09	1.16	Up	0.036236	NA
CG3719	44736	5.16	5.38	1.16	Up	0.019642	NA
CG17337	35507	6.10	6.32	1.16	Up	0.016465	NA
boi	31229	5.09	4.87	1.16	Down	0.010207	NA
Adar	31130	6.21	5.99	1.16	Down	0.043778	NA

CG34317	5740224	5.15	5.36	1.16	Up	0.02048	NA
CG7950	43579	5.15	5.36	1.16	Up	0.02048	NA
Rrp1	33500	8.13	8.34	1.16	Up	0.003718	NA
shrb	35933	6.73	6.94	1.16	Up	0.041807	NA
CG1553	35730	6.63	6.84	1.16	Up	0.044575	NA
Ykt6	31706	6.18	6.39	1.16	Up	0.008528	NA
Vrp1	37521	5.87	6.08	1.16	Up	0.033444	NA
CG11448	31090	6.91	7.12	1.16	Up	0.025539	NA
rb	31381	6.77	6.97	1.15	Up	0.003607	NA
CG9171	33807	4.87	4.66	1.15	Down	0.042627	NA
Syt4	40876	8.53	8.74	1.15	Up	0.029357	NA
CG9132	2768881	6.06	6.27	1.15	Up	0.005226	NA
RpL18A	36985	10.07	10.28	1.15	Up	0.014954	NA
sti	39429	8.03	7.83	1.15	Down	0.015908	0.301703
Nlp	43560	8.82	9.03	1.15	Up	0.044512	NA
SIDL	41833	5.71	5.51	1.15	Down	0.032601	NA
tinc	42148	8.65	8.85	1.15	Up	0.041053	NA
Vinc	31201	6.38	6.59	1.15	Up	0.009122	NA
spin	45380	6.98	7.18	1.15	Up	0.025716	NA
CG33181	318916	5.73	5.93	1.15	Up	0.008862	NA
CG33096	326251	6.28	6.08	1.15	Down	0.013788	NA
sax	35731	5.76	5.96	1.15	Up	0.045167	NA
disco-r	64875	5.76	5.56	1.15	Down	0.021436	0.334537
ns4	35338	5.51	5.71	1.15	Up	0.001301	NA
CG16896	37977	5.52	5.72	1.15	Up	0.041874	NA
CG34353	5740590	5.56	5.76	1.15	Up	0.041968	NA
Bap55	36956	7.10	7.29	1.14	Up	0.004659	NA
Sema-1b	37007	7.24	7.05	1.14	Down	0.041922	0.285009
Hsc70-4	41840	12.16	12.36	1.14	Up	0.036502	NA
CG11927	33687	5.67	5.47	1.14	Down	9.57E-05	0.320212
Elp3	33649	5.19	5.38	1.14	Up	0.04858	NA
MED19	39987	6.64	6.46	1.14	Down	0.044932	NA
RpS15Aa	44150	9.45	9.64	1.14	Up	0.026626	NA
Sap-r	43662	9.59	9.77	1.14	Up	0.033072	NA
CG9170	32563	7.45	7.64	1.14	Up	0.027964	NA
CG2915	35754	7.05	7.23	1.14	Up	0.025429	NA
Hph	40633	5.89	6.08	1.14	Up	0.032973	NA
CG1518	33082	8.13	8.32	1.14	Up	0.013822	NA
CG7185	38937	9.06	8.87	1.14	Down	0.026995	0.205585
CG12424	36673	7.46	7.27	1.14	Down	0.019694	0.210774
Sesn	37755	5.02	5.20	1.14	Up	0.028161	NA
Sesn	37755	5.02	5.20	1.14	Up	0.028161	NA

CG6049	40369	5.80	5.62	1.13	Down	0.043121	0.298784
Sam-S	48552	8.15	8.33	1.13	Up	0.022739	NA
CG14213	32966	7.46	7.64	1.13	Up	0.043031	NA
CG11504	43557	6.56	6.38	1.13	Down	0.005324	NA
Ssrp	37767	8.13	8.31	1.13	Up	0.005496	NA
Rca1	33959	5.99	6.17	1.13	Up	0.036156	NA
CG4502	34002	6.72	6.89	1.13	Up	0.04501	NA
Set2	32301	7.61	7.44	1.13	Down	0.02468	NA
CG12484	37310	6.14	5.97	1.13	Down	0.043498	NA
ecd	38291	6.50	6.33	1.13	Down	0.021521	NA
RnpS1	41147	7.38	7.20	1.13	Down	0.037634	NA
Surf4	41864	6.17	6.34	1.13	Up	0.004833	NA
CG4293	31001	5.52	5.69	1.13	Up	0.019972	NA
skpA	31016	7.46	7.63	1.12	Up	0.044819	NA
Rtc1	32338	4.34	4.51	1.12	Up	0.042441	NA
CG4674	41320	5.09	5.25	1.12	Up	0.046093	NA
twin	42880	7.40	7.56	1.12	Up	0.037373	NA
CG31855	318983	5.24	5.40	1.12	Up	0.012446	NA
gfzf	40858	6.69	6.86	1.12	Up	0.007355	NA
CG5745	42498	5.43	5.60	1.12	Up	0.042093	NA
CG15744	2768909	7.41	7.25	1.12	Down	0.012221	NA
dod	33111	6.69	6.85	1.12	Up	0.049093	NA
KdelR	34427	6.91	7.07	1.12	Up	0.035286	NA
CG9147	33855	4.66	4.82	1.12	Up	0.025106	NA
CG10347	32141	5.37	5.53	1.12	Up	0.047009	NA
CG10627	39434	5.75	5.59	1.12	Down	0.009976	NA
CG5946	39336	5.72	5.87	1.11	Up	0.037411	NA
Taf1	40813	7.85	7.70	1.11	Down	0.042935	0.311162
l(3)05822	47260	6.45	6.60	1.11	Up	0.020439	NA
CG14782	31105	6.68	6.83	1.11	Up	0.015917	NA
CG8372	34121	5.33	5.18	1.11	Down	0.04695	NA
Ef1alpha100E	43736	11.11	11.25	1.11	Up	0.018765	NA
RpS13	34149	9.84	9.98	1.10	Up	0.04153	NA
Smg6	42994	6.41	6.56	1.10	Up	0.017922	NA
CG6724	34488	5.41	5.55	1.10	Up	0.027683	NA
128up	36288	5.86	6.00	1.10	Up	0.034727	NA
Tcp-1zeta	32518	8.46	8.59	1.10	Up	0.031597	NA
grp	34993	7.80	7.93	1.10	Up	0.043522	NA
CG6236	41857	6.55	6.68	1.10	Up	0.029679	NA
Rab35	33014	7.30	7.43	1.10	Up	0.036234	NA
CG9705	39875	8.22	8.35	1.09	Up	0.041692	NA
Nup154	34527	6.75	6.88	1.09	Up	0.009063	NA

bur	45830	6.84	6.97	1.09	Up	0.023329	NA
CG2025	32143	6.86	6.73	1.09	Down	0.018124	NA
CG7878	40959	6.45	6.57	1.09	Up	0.045138	NA
CG7332	32885	6.45	6.57	1.09	Up	0.005166	NA
Nap1	37798	8.69	8.81	1.09	Up	0.032857	NA
RpL7A	31588	10.76	10.88	1.08	Up	0.027734	NA
wuho	31566	6.22	6.33	1.08	Up	0.025997	NA
Cctgamma	42029	8.66	8.77	1.08	Up	0.041267	NA
Ptpmeg	38059	7.67	7.78	1.08	Up	0.022273	NA
D1	41095	9.80	9.70	1.07	Down	0.048673	NA
Tom7	35899	5.45	5.35	1.07	Down	0.028487	NA
Synd	42467	6.15	6.25	1.07	Up	0.030868	NA
opa	40605	6.25	6.33	1.06	Up	0.024776	NA
Su(var)3-9	41843	7.94	8.01	1.05	Up	0.045205	NA
CG8223	41045	8.15	8.21	1.04	Up	0.018312	NA

Terms and abbreviations: Gene_Identifier, the flybase symbol of the corresponding differentially expressed gene, A1_mean, the mean expression level of A1 p-element revertant controls, G2_mean, the mean expression level of TBPH[G2] allele (homozygous), Ratio, the fold-change of expression in G2 compared to A1, Direction, the direction of the change, p_value, the genesifter p value (see methods for parameters), edgeR p value adjusted for multiple hypothesis testing (Benjamini & Hochberg 1995). NA, the gene was not differentially expressed using edgeR analysis.
