# Supplementary material for: Comparison of Parallel High-Throughput RNA Sequencing Between Knockout of TDP-43 and Its Overexpression Reveals Primarily Nonreciprocal and Nonoverlapping Gene Expression Changes in the Central Nervous System of Drosophila
Source: G3 (Bethesda). 2012 Jul 1;2(7):789–802. doi: 10.1534/g3.112.002998 (PMC3385985; doi:10.1534/g3.112.002998)
Supplement: Supporting Information [file supp_2.7.789_TableS2.pdf]

Table S2 Gene Expression changes in D42>TBPH

Flybase ID	Entrez	LacZ	TBPH	Ratio	Direction	P Value	adj_P_Val
Tpr2	45556	-1.30	3.08	20.91	Up	0.000449	NA
CG17669	37125	-0.97	3.37	20.31	Up	0.00117	2.24E-16
CG3984	41821	4.40	0.21	18.27	Down	0.000125	1.17E-24
Cyp4p2	35946	3.45	-0.43	14.67	Down	0.000221	NA
CG12607	38547	2.18	-1.62	13.99	Down	0.000269	4.53E-09
Magi	41820	4.26	0.59	12.75	Down	0.000447	2.13E-30
snoRNA:Psi28S-2263	40157	3.37	-0.10	11.04	Down	0.012306	NA
bgm	45524	1.65	-1.62	9.65	Down	0.002793	2.82E-08
CR40734	5740371	-1.30	1.47	6.83	Up	0.049309	NA
CR41548	5740812	-0.44	2.31	6.74	Up	0.038967	NA
CR41539	5740187	-0.64	2.05	6.44	Up	0.033255	NA
CR41544	5740694	0.16	2.71	5.84	Up	0.024901	NA
CG42382	40957	-0.77	1.66	5.4	Up	0.026403	0.007291
CG30015	41822	2.05	-0.32	5.18	Down	0.018011	NA
Pepck	37131	3.78	1.51	4.83	Down	0.032896	8.35E-15
CG14277	34129	1.90	-0.10	3.99	Down	0.006754	0.005358
brp	42344	2.69	0.71	3.95	Down	0.012885	2.45E-09
CG42306	40588	1.82	3.79	3.93	Up	0.01895	1.32E-11
CG6701	42443	-0.11	1.86	3.91	Up	0.003875	0.015887
Catsup	37712	3.32	1.53	3.46	Down	0.006132	0.000188
Nplp4	50190	1.14	2.85	3.28	Up	0.035451	0.026685
Msp-300	35199	0.23	1.87	3.13	Up	0.000972	0.121946
CG30187	246509	2.35	0.77	2.99	Down	0.035893	0.40095
Dhc36C	35061	2.31	0.78	2.89	Down	0.007782	0.31854
CG14946	34627	2.57	1.10	2.78	Down	0.014636	0.00177
CG2976	42294	3.28	1.82	2.75	Down	0.024819	NA
Gug	39621	1.02	2.46	2.72	Up	0.019709	NA
CG4892	53502	2.60	4.02	2.66	Up	0.022874	NA
CG13897	38090	4.70	3.31	2.61	Down	0.011887	1.71E-06
phr	35735	5.76	4.43	2.51	Down	0.001317	2.45E-14
CG31728	34731	2.99	1.70	2.43	Down	0.045172	0.098217
CG8814	42049	4.51	3.23	2.43	Down	0.04415	9.15E-07
Ir62a	3885628	3.09	1.81	2.42	Down	0.026837	NA
CG13082	35244	1.13	2.40	2.41	Up	0.018851	0.118273
CG31797	32767	2.73	1.48	2.39	Down	0.000822	0.059971
Gdi	39567	2.95	1.72	2.34	Down	0.020901	NA
CG3788	37644	3.09	4.29	2.3	Up	0.015163	7.55E-06
snoRNA:Me28S-A576	40701	0.49	1.69	2.29	Up	0.010014	NA
Doc3	40266	4.38	3.21	2.26	Down	0.00105	2.96E-05
s-cup	36371	3.08	1.90	2.26	Down	0.023767	NA

CG42796	41017	2.64	1.47	2.25	Down	0.027202	NA
CG34458	40868	4.54	5.70	2.25	Up	0.028068	6.22E-11
CG31643	43126	2.86	1.69	2.24	Down	0.042358	0.343689
5740512	5740512	0.86	2.02	2.23	Up	0.038367	NA
CG6424	32419	3.42	2.26	2.22	Down	0.01045	0.021966
Ady43A	44750	4.90	6.00	2.15	Up	0.006933	NA
CG33271	32084	0.67	1.73	2.1	Up	0.034625	NA
cni	53522	1.46	2.53	2.09	Up	0.031436	NA
stl	37619	1.93	2.96	2.04	Up	0.048928	0.077571
CG42801	4379858	3.04	2.03	2	Down	0.016104	NA
for	47878	1.89	2.89	2	Up	0.028341	0.332967
Gadd45	35646	1.32	2.30	1.97	Up	0.017649	NA
CG5160	34015	1.25	2.21	1.96	Up	0.040538	NA
akirin	40427	4.16	3.20	1.95	Down	0.000369	0.00666
CG14471	35525	2.30	1.34	1.94	Down	0.038649	0.53413
CG12911	36081	3.51	4.46	1.93	Up	0.023607	0.00128
Egfr	39566	8.20	7.25	1.93	Down	0.043681	6.81E-14
CG8552	41202	6.81	7.75	1.92	Up	0.026908	1.64E-10
CG13272	35013	2.27	1.33	1.92	Down	0.007575	0.392587
Socs36E	43583	2.40	1.48	1.9	Down	0.018836	NA
CG42700	36330	2.01	1.09	1.9	Down	0.017414	NA
CG8679	40205	6.63	5.73	1.87	Down	0.004442	NA
Catsup	37709	2.77	1.87	1.86	Down	0.000189	0.362167
Chd1	39048	4.61	3.72	1.86	Down	0.009781	NA
CG14502	42055	2.60	3.48	1.84	Up	0.033918	0.420993
CG5830	39748	7.49	6.61	1.84	Down	0.012054	4.55E-09
CG16741	40728	2.55	3.42	1.83	Up	0.03618	0.069552
Gmd	39238	3.55	2.69	1.82	Down	0.002921	NA
nopo	37083	2.79	1.93	1.82	Down	0.048664	NA
CR18854	32148	6.95	6.09	1.82	Down	0.009063	2.93E-07
Dap160	39662	2.93	2.10	1.78	Down	0.007585	NA
Gal	31761	2.74	3.57	1.78	Up	0.014134	0.116775
CG34398	5740462	3.96	3.13	1.77	Down	0.015492	0.05469
CG9951	41766	2.66	1.84	1.76	Down	0.042753	NA
TotX	31193	3.51	2.70	1.75	Down	0.028059	0.102051
mir-307-as	42879	2.33	1.52	1.75	Down	0.002714	NA
lectin-21Cb	31271	5.95	6.75	1.74	Up	0.007816	NA
CG15674	37463	2.53	1.74	1.73	Down	0.00619	NA
Acox57D-p	37445	2.06	2.84	1.73	Up	0.03783	0.191724
Ir31a	31718	5.04	4.25	1.73	Down	0.028432	0.001299
CG3732	40297	1.82	1.03	1.72	Down	0.03864	NA
osp	44110	5.32	6.10	1.72	Up	0.001689	1.17E-06
CG12594	41416	6.25	5.48	1.7	Down	0.002161	7.55E-06

Cbp53E	40422	2.96	3.73	1.7	Up	0.007211	0.125953
CG13966	35284	1.78	2.53	1.68	Up	0.047131	NA
CG18853	246511	4.43	3.68	1.68	Down	0.009838	NA
mir-278	42945	3.81	4.55	1.67	Up	0.007221	0.002298
gammaTub37C	42271	2.33	1.59	1.67	Down	0.046855	NA
Fibp	41819	3.72	2.98	1.67	Down	0.025113	NA
Adgf-E	37551	1.28	2.02	1.67	Up	0.03402	NA
salm	36991	4.12	3.39	1.66	Down	0.010389	0.126957
CG30392	246587	4.34	3.61	1.66	Down	0.000586	0.015333
Obp44a	45326	8.19	7.46	1.66	Down	0.013578	1.34E-06
CG42866	42866	3.23	3.95	1.65	Up	0.001948	NA
sec31	35877	7.89	7.17	1.65	Down	0.006275	NA
Herp	39854	5.24	4.52	1.64	Down	0.000345	0.003947
CG15100	50388	4.59	3.88	1.64	Down	0.020081	NA
Mip	39933	4.29	3.58	1.64	Down	0.021901	0.062798
CG42750	35094	3.15	2.43	1.64	Down	0.007505	NA
CG1827	35989	3.48	4.19	1.64	Up	0.016807	0.054096
E2f2	39263	3.18	3.88	1.64	Up	0.046982	1.66E-05
Fbp2	34259	4.94	4.24	1.63	Down	0.046694	0.003947
RpL27A	41092	2.13	2.82	1.62	Up	0.03443	0.384406
pdm2	32441	1.88	2.57	1.61	Up	0.032088	0.4678
l(2)05714	46066	4.16	4.84	1.6	Up	0.010277	0.048475
CG8740	35866	3.26	2.58	1.6	Down	0.003126	NA
mRpS2	33688	4.41	3.74	1.59	Down	0.023328	0.420993
CG42268	42268	3.40	4.07	1.59	Up	0.031103	0.273177
CG3394	37887	2.19	2.86	1.59	Up	0.006181	0.228537
l(2)35Bg	40916	2.98	3.65	1.59	Up	0.017085	NA
CG9140	40424	7.10	6.44	1.58	Down	0.004544	5.30E-05
Tom20	43235	3.39	2.73	1.58	Down	0.035311	0.451935
CG5946	31249	3.83	3.16	1.58	Down	0.048722	NA
CG32640	318135	3.52	4.18	1.57	Up	0.026336	NA
Ast	42947	5.00	4.35	1.56	Down	0.041968	0.007838
Zasp52	36740	3.20	2.56	1.56	Down	0.048659	NA
membrin	38614	3.33	2.69	1.56	Down	0.029299	NA
CG13800	38305	4.81	4.17	1.56	Down	0.040561	0.351735
CR30009	39517	3.23	2.59	1.56	Down	0.047788	NA
CG7366	41710	3.84	4.48	1.55	Up	0.017267	NA
v(2)k05816	31502	4.48	3.85	1.55	Down	0.017021	NA
ventrally-expressed-protein-D	117331	2.47	3.11	1.55	Up	0.014476	NA
Hen1	36301	1.64	2.27	1.55	Up	0.010345	NA
CG9932	32469	4.87	4.24	1.55	Down	0.044803	0.118082
CG8950	43751	7.70	7.08	1.54	Down	0.001276	0.000272

Pp2A-29B	32028	4.51	3.88	1.54	Down	0.049109	0.182235
RpL40	37227	3.17	2.55	1.54	Down	0.041095	NA
CG7264	50001	2.14	2.76	1.54	Up	0.007733	NA
CG1273	38522	2.53	1.91	1.53	Down	0.036546	NA
calypso	41384	3.20	2.58	1.53	Down	0.044129	NA
Mis12	38762	3.13	2.52	1.53	Down	0.047486	NA
tacc	41502	2.71	3.32	1.53	Up	0.007768	NA
CG16970	34740	6.21	5.59	1.53	Down	0.030011	0.00666
CG13575	38352	5.52	4.91	1.53	Down	0.020993	NA
Sin3A	39317	5.18	5.79	1.53	Up	0.025495	0.000283
Mitf	3885647	4.27	4.88	1.53	Up	0.006326	NA
cenG1A	48336	3.79	4.40	1.52	Up	0.048313	0.070744
Hr51	36702	3.13	2.53	1.52	Down	0.023511	NA
CG7777	36237	4.01	3.40	1.52	Down	0.001274	0.040622
imd	38957	3.85	3.25	1.52	Down	0.001311	NA
CG15828	44002	3.00	3.60	1.52	Up	0.017302	NA
CG8086	34131	5.08	4.48	1.51	Down	0.049406	0.186158
Rpp30	31237	3.17	2.58	1.51	Down	0.035714	NA
CR14578	40059	6.08	6.67	1.51	Up	0.017083	0.00013
tim	32401	4.40	3.80	1.51	Down	0.035079	0.142297
CG9510	34681	4.78	4.19	1.51	Down	0.010824	0.098217
CG14593	35535	3.45	2.86	1.51	Down	0.003644	NA
Ret	32930	7.54	6.96	1.5	Down	0.026735	0.003947
CG1814	35984	4.86	4.27	1.5	Down	0.033477	0.405891
CG31344	31344	4.68	4.09	1.5	Down	0.010613	NA
Indy	40049	5.74	5.15	1.5	Down	0.015006	NA
Idgf3	34981	6.97	6.39	1.5	Down	9.21E-05	0.001386
CG33213	338395	4.37	3.79	1.5	Down	0.031199	0.290773
A16	32468	6.30	5.72	1.5	Down	0.026148	0.026147
CG5924	34307	3.30	3.88	1.49	Up	0.045936	0.405891
CG8475	40430	3.88	3.31	1.49	Down	0.049432	0.262212
Tango1	42835	4.05	4.62	1.48	Up	0.02415	NA
CG10341	31429	3.56	2.99	1.48	Down	0.011545	NA
CG1969	43530	4.00	3.44	1.48	Down	0.009633	NA
CG1265	38517	2.60	3.17	1.48	Up	0.01375	NA
CG17036	34679	3.39	3.95	1.48	Up	0.018873	0.384406
CaBP1	41616	6.59	6.03	1.47	Down	0.020286	NA
GABA-B-R1	34878	4.43	3.87	1.47	Down	0.033725	NA
CG9948	41626	4.17	3.61	1.47	Down	0.040022	NA
JIL-1	43551	5.16	5.71	1.46	Up	0.002952	0.011397
CR32385	42503	4.13	4.67	1.46	Up	0.006713	0.170104
mdy	32610	4.13	3.59	1.46	Down	0.010821	0.331745
daw	33474	5.73	5.19	1.46	Down	0.001721	0.102708

CG33339	33339	4.54	4.01	1.45	Down	0.000285	NA
snmRNA:128	3771829	3.35	2.82	1.45	Down	0.037279	NA
santa-maria	41317	4.46	3.93	1.44	Down	0.020717	0.234793
CG6201	42339	4.19	4.72	1.44	Up	0.033606	0.125953
CG34370	50405	3.93	3.41	1.43	Down	0.015952	NA
CaBP1	39962	7.99	7.47	1.43	Down	0.048012	0.004571
dpp	33432	3.99	3.47	1.43	Down	0.044814	0.347364
CG12187	38337	5.29	4.78	1.43	Down	0.011323	0.509133
CG13330	36501	6.46	5.95	1.43	Down	0.002787	NA
CG9319	35337	4.06	4.58	1.43	Up	0.04172	NA
CG32500	2768879	3.81	3.30	1.43	Down	0.001814	NA
CG32857	318252	3.65	3.14	1.42	Down	0.01842	NA
CG31729	31729	5.50	4.99	1.42	Down	0.033985	0.254171
Catsup	37708	4.92	5.43	1.42	Up	0.007056	0.026985
CG3407	33606	3.81	4.31	1.42	Up	0.043826	0.267378
CG13901	45706	2.92	2.42	1.41	Down	0.041665	NA
tim	38501	4.82	4.32	1.41	Down	0.028118	NA
CG5660	39023	3.87	3.37	1.41	Down	0.038606	NA
ush	42440	3.76	4.25	1.41	Up	0.046118	0.43007
CG31954	31543	6.80	6.31	1.4	Down	0.044103	NA
botv	38793	7.02	6.53	1.4	Down	0.036859	NA
Ppcdc	37882	5.27	4.78	1.4	Down	0.006221	NA
Pmi	3772100	5.06	4.57	1.4	Down	0.002972	NA
CG13868	37302	7.52	7.03	1.4	Down	0.009833	0.026985
BicC	41473	4.43	3.95	1.4	Down	0.007827	NA
aret	34648	6.21	5.73	1.39	Down	0.010663	NA
FucTC	3355171	3.58	3.11	1.39	Down	0.047457	NA
Gmd	42351	3.70	4.18	1.39	Up	0.02754	NA
Socs44A	35786	5.44	4.96	1.39	Down	0.010176	0.378194
fusl	33765	4.13	3.66	1.39	Down	0.040061	NA
mir-184	42925	5.25	4.77	1.39	Down	0.044097	NA
CG10543	41340	6.04	5.57	1.38	Down	0.044889	NA
CG7120	38954	4.88	4.41	1.38	Down	0.018226	0.427281
CG6043	34732	3.88	3.41	1.38	Down	0.009849	NA
prom	42310	7.14	6.67	1.38	Down	0.010623	NA
CG9314	40339	5.61	5.15	1.38	Down	0.030702	0.389559
CG9130	39611	3.82	4.28	1.38	Up	0.028969	0.267378
osp	34850	6.09	5.63	1.37	Down	0.01311	0.099178
Jheh2	41355	3.82	4.27	1.37	Up	0.018599	0.194671
EndoG	43293	2.90	3.36	1.37	Up	0.026149	NA
mbl	36945	7.63	7.18	1.37	Down	0.017529	0.020587
CG3376	37884	6.27	5.82	1.37	Down	0.030479	0.12581
Fuca	3772574	5.49	5.04	1.37	Down	0.047797	0.40095

CG11714	3772566	5.49	5.04	1.37	Down	0.047797	NA
Hydr1	35930	4.93	4.48	1.37	Down	0.041762	NA
Pkc53E	40124	5.67	6.11	1.36	Up	0.000282	0.110654
Cf2	33692	6.39	5.94	1.36	Down	0.020281	0.419474
CG1675	36022	4.33	3.88	1.36	Down	0.013017	NA
Mmp2	38523	7.15	6.70	1.36	Down	0.030197	0.125953
CG42266	42266	4.46	4.90	1.36	Up	0.00995	0.188392
CG8486	34112	6.93	6.49	1.36	Down	0.049041	0.2488
CG4133	36570	3.70	4.14	1.36	Up	0.005257	0.527191
FucTD	44126	1.92	2.36	1.36	Up	0.040167	NA
PGRP-LD	3771920	4.22	3.78	1.35	Down	0.00332	NA
CG31688	40999	5.60	5.16	1.35	Down	0.041068	0.317133
CG42750	42750	4.70	4.27	1.35	Down	0.010674	0.429966
CG11912	40524	5.71	5.28	1.35	Down	0.006572	0.288112
hig	35949	7.60	7.17	1.34	Down	0.013471	0.113908
rok	43916	7.02	6.60	1.34	Down	0.022776	0.18697
CG5446	34655	5.39	5.81	1.34	Up	0.000201	0.444587
Ugt36Bc	32606	4.90	4.48	1.34	Down	0.045213	0.311492
CG31251	318645	3.34	3.77	1.34	Up	0.043324	NA
CG17742	38787	6.04	5.62	1.34	Down	0.014831	0.289351
ck	36656	5.26	4.84	1.34	Down	0.04025	NA
CG11180	37330	4.69	5.11	1.34	Up	0.049615	0.339929
dpr3	3346208	4.29	3.87	1.34	Down	0.010102	NA
CG15643	32489	3.61	3.19	1.33	Down	0.015692	0.542998
cpx	32490	5.39	4.97	1.33	Down	0.002076	0.431609
Rx	41835	4.60	4.18	1.33	Down	0.025915	NA
NHP2	44005	4.82	5.23	1.33	Up	0.047945	NA
rig	32233	6.35	5.94	1.33	Down	0.002674	0.408399
blue	39939	5.06	4.64	1.33	Down	0.035186	NA
Shawl	32113	4.54	4.95	1.33	Up	0.043883	0.102578
CG13784	34003	6.18	5.77	1.33	Down	0.022977	0.223418
CG8678	35396	4.87	4.46	1.33	Down	0.013731	0.529443
GC	38194	5.63	6.03	1.33	Up	0.019519	0.312127
mre11	34565	4.97	4.57	1.33	Down	0.04395	0.376261
CG1695	33046	5.84	5.44	1.32	Down	0.023696	0.165713
DNApol-gamma35	3772064	5.32	5.72	1.32	Up	0.045621	NA
CG33649	3772218	5.32	5.72	1.32	Up	0.045621	NA
tweek	53586	5.52	5.12	1.32	Down	0.005346	0.529944
tutl	38657	3.50	3.90	1.32	Up	0.045415	NA
CG9008	34779	3.79	3.39	1.32	Down	0.005822	NA
snmRNA:357	3772300	7.13	7.53	1.32	Up	0.03277	NA
CG17082	3355133	5.43	5.83	1.32	Up	0.048565	0.311492
CG34381	31743	3.11	2.71	1.32	Down	0.041236	NA

hk	41578	3.25	2.85	1.32	Down	0.041486	NA
Skeletor	3771796	3.67	3.27	1.32	Down	0.033858	NA
Skeletor	3772559	3.67	3.27	1.32	Down	0.033858	NA
CG31714	34341	4.98	4.58	1.31	Down	0.047241	NA
Oatp26F	33927	3.66	3.27	1.31	Down	0.023559	NA
CG7741	36208	4.20	4.60	1.31	Up	0.046852	NA
rdo	35077	4.57	4.18	1.31	Down	0.037158	NA
mfas	41455	8.19	7.80	1.31	Down	0.0272	0.081752
Prosalpha1	45780	6.51	6.12	1.31	Down	0.014568	NA
Rab14	44870	5.19	5.57	1.31	Up	0.000291	0.104046
CG10664	38885	5.02	4.63	1.31	Down	0.036964	0.467953
CG6520	36967	5.20	5.58	1.31	Up	0.046791	0.161705
ine	33659	4.76	4.38	1.3	Down	0.024958	NA
d	31610	5.06	4.67	1.3	Down	0.003864	NA
CG10249	36668	5.94	5.56	1.3	Down	0.035681	NA
rdgBbeta	39582	3.14	2.76	1.3	Down	0.042324	NA
srpk79D	41113	3.69	3.31	1.3	Down	0.033283	0.467325
CG2976	33682	5.13	4.75	1.3	Down	0.003785	0.295955
CG3975	38629	7.62	7.99	1.3	Up	0.01812	NA
CG34353	5740590	5.77	5.40	1.3	Down	0.047338	NA
CG10431	35157	4.24	4.62	1.3	Up	0.040184	NA
TepII	45970	4.62	4.25	1.29	Down	0.005991	NA
CG34380	40323	5.20	4.83	1.29	Down	0.028805	NA
Rgk2	37132	4.40	4.03	1.29	Down	0.015394	NA
CG32815	318224	6.06	5.69	1.29	Down	0.008855	0.439892
CG13993	43682	4.78	4.41	1.29	Down	0.017918	0.434994
snRNA:U2:38ABa	32882	4.58	4.21	1.29	Down	0.017974	NA
CG14590	48903	4.53	4.90	1.29	Up	0.01625	NA
Smc5	326215	5.30	5.67	1.29	Up	0.049318	NA
ics	38735	5.08	5.45	1.29	Up	0.037363	0.496872
CG3021	53546	4.66	4.30	1.29	Down	0.005782	NA
CG34371	32922	4.53	4.16	1.29	Down	0.006553	NA
dpr9	2768670	5.76	5.39	1.29	Down	0.040939	NA
CG32039	317834	3.16	3.53	1.29	Up	0.040899	NA
CG11455	39331	4.47	4.83	1.29	Up	0.017375	0.509969
CG10338	35793	4.98	5.34	1.28	Up	0.013648	NA
CG15270	34886	6.12	5.76	1.28	Down	0.015258	0.191724
d4	42505	3.21	3.57	1.28	Up	0.029713	NA
Lrr47	34449	4.12	3.76	1.28	Down	0.048854	NA
CG31772	33601	6.31	5.95	1.28	Down	0.047919	0.31614
CG33631	31934	3.06	3.41	1.28	Up	0.01364	NA
dpr12	50320	5.63	5.27	1.28	Down	0.00499	NA
CG31953	31953	7.15	6.79	1.28	Down	0.002421	NA

CG33923	38930	3.29	3.64	1.27	Up	0.018837	NA
CG17912	35004	7.30	6.95	1.27	Down	0.031361	0.431609
CG4945	38845	6.54	6.19	1.27	Down	0.03219	NA
Alg10	32076	4.88	4.53	1.27	Down	0.018449	NA
sec71	34785	6.66	6.31	1.27	Down	0.039804	NA
Ir41a	38817	6.34	5.99	1.27	Down	0.00139	NA
Ca-alpha1D	42045	4.56	4.90	1.27	Up	0.010761	NA
CG3570	37934	3.73	4.08	1.27	Up	0.019854	NA
CG12214	36072	5.72	5.38	1.27	Down	0.038534	0.466225
bft	34639	5.92	6.27	1.27	Up	0.025019	0.267378
fau	41994	4.00	3.66	1.27	Down	0.0243	NA
CG6614	38752	7.13	6.79	1.27	Down	0.005838	0.428626
Pde1c	34594	6.48	6.13	1.27	Down	0.009011	0.392587
CG15630	31970	5.00	4.66	1.27	Down	0.003348	0.503575
Aldh-III	41571	5.06	4.72	1.27	Down	0.023438	NA
nxf2	41644	5.80	5.46	1.26	Down	0.008273	NA
CG30460	36924	6.03	5.70	1.26	Down	0.029185	NA
ACXD	33110	6.88	6.54	1.26	Down	0.010771	0.383025
CG31244	31244	5.16	4.82	1.26	Down	0.049993	NA
CG1648	40794	6.19	5.85	1.26	Down	0.031008	NA
CG8617	36594	5.51	5.17	1.26	Down	0.021017	NA
CG42389	34987	7.42	7.09	1.26	Down	0.039772	0.35522
Neu3	3772109	6.75	7.08	1.26	Up	0.021074	NA
CG34393	33534	3.74	3.41	1.26	Down	0.038898	NA
CG9510	3771738	3.66	3.33	1.26	Down	0.048218	NA
CG9515	3771965	3.66	3.33	1.26	Down	0.048218	NA
CG10880	42726	5.46	5.14	1.26	Down	0.010068	0.33168
yellow-c	34879	4.11	4.44	1.25	Up	0.016643	NA
Acp53Ea	37382	6.34	6.01	1.25	Down	0.014166	0.472786
CG33123	53472	7.55	7.23	1.25	Down	0.000752	0.509133
zuc	246582	6.60	6.28	1.25	Down	0.014265	NA
CG33116	32625	7.43	7.11	1.25	Down	0.013976	0.302478
CG10492	35177	6.96	6.65	1.25	Down	0.038244	0.54266
Ip259	41376	5.23	4.91	1.25	Down	0.002889	NA
ush	33225	5.58	5.26	1.25	Down	0.045819	0.529944
Atf-2	37978	7.61	7.30	1.24	Down	0.016068	0.43007
Fmrf	36147	7.26	6.94	1.24	Down	0.033813	0.376025
sr	42402	5.94	5.63	1.24	Down	0.046221	NA
CG10912	40265	4.72	4.41	1.24	Down	0.012446	0.451935
l(2)tid	43506	5.79	5.47	1.24	Down	0.042428	0.431609
Slbp	43448	5.33	5.02	1.24	Down	0.011306	NA
Acp26Aa	37218	5.50	5.19	1.24	Down	0.020264	NA
mRpL17	43278	4.39	4.70	1.24	Up	0.049459	NA

Pkg21D	40147	5.11	4.79	1.24	Down	0.010338	NA
CG17273	32788	6.01	5.70	1.24	Down	0.009054	NA
Gef26	31798	7.04	6.73	1.24	Down	0.024021	0.451935
wb	32970	7.38	7.07	1.24	Down	0.029728	NA
CG31778	31565	6.97	6.67	1.24	Down	0.015478	NA
CG31646	40948	5.77	5.46	1.24	Down	0.010022	NA
CG1317	38300	7.13	6.83	1.24	Down	0.004766	NA
CG9641	40659	5.29	4.99	1.23	Down	0.001328	NA
Spred	36643	7.92	7.62	1.23	Down	0.022701	0.355773
CG33145	41403	5.60	5.30	1.23	Down	0.024149	0.296358
ACXB	33108	6.91	6.61	1.23	Down	0.009807	NA
dp	31964	7.48	7.18	1.23	Down	0.032315	NA
Adh	36580	5.94	6.24	1.23	Up	0.026749	0.392587
CG3558	48421	6.80	6.50	1.23	Down	0.040649	NA
Ptpmeg	41636	6.53	6.23	1.23	Down	0.025797	0.455822
lectin-22C	42295	6.14	5.84	1.23	Down	0.01827	NA
Hmx	42110	5.93	5.64	1.23	Down	0.004251	NA
CG17660	33347	6.48	6.78	1.23	Up	0.004263	0.244993
CG33932	338392	6.18	5.88	1.23	Down	0.002773	NA
Rpp20	3772007	6.18	5.88	1.23	Down	0.002773	NA
CG1516	39627	4.92	4.62	1.23	Down	0.042946	NA
CG4577	33291	8.17	7.87	1.23	Down	0.02946	0.472786
Zasp52	38978	9.89	9.60	1.23	Down	0.041866	NA
CG42303	42303	3.70	3.41	1.22	Down	0.040607	NA
FK506-bp2	41441	6.47	6.18	1.22	Down	0.015847	NA
hipk	39732	7.12	6.83	1.22	Down	0.032088	0.320696
CG9330	40131	6.59	6.30	1.22	Down	0.049176	NA
CG5028	118436	6.53	6.25	1.22	Down	0.021803	NA
crol	45931	6.51	6.22	1.22	Down	0.013911	NA
Sos	34912	5.49	5.21	1.22	Down	0.011383	NA
svp	41491	6.25	5.97	1.22	Down	0.019667	NA
Ire1	42358	6.23	5.95	1.22	Down	0.049362	NA
CG1667	36016	3.27	3.55	1.22	Up	0.025731	NA
CG1134	38472	3.69	3.41	1.21	Down	0.025845	NA
RYBP	37601	6.36	6.08	1.21	Down	0.010602	NA
Hmgs	44154	6.23	5.96	1.21	Down	0.030339	NA
Iap2	32373	7.56	7.28	1.21	Down	0.013725	NA
CG6498	39686	7.84	7.56	1.21	Down	0.043239	NA
oys	37306	8.77	8.50	1.21	Down	0.049926	NA
Syt1	38716	5.60	5.32	1.21	Down	0.003163	NA
dos	32135	7.04	6.76	1.21	Down	0.024332	NA
Hrs	33565	6.75	6.48	1.21	Down	0.015042	NA
PHDP	32684	7.10	6.83	1.21	Down	0.031428	0.515358

CG13295	38692	3.53	3.80	1.21	Up	0.038774	NA
mir-2b-1	42175	6.23	5.95	1.21	Down	0.013821	NA
tau	326116	6.96	6.69	1.21	Down	0.009074	NA
CG5287	34711	3.43	3.71	1.21	Up	0.036686	NA
Nnf1b	31658	6.87	6.60	1.21	Down	0.027361	NA
Catsup	37781	7.11	7.38	1.21	Up	0.028224	NA
CG2955	38195	10.79	10.52	1.21	Down	0.003165	0.297552
pelo	34286	5.63	5.36	1.21	Down	0.02782	NA
Tom7	35899	5.32	5.59	1.21	Up	0.037214	NA
Ced-12	34633	6.11	6.38	1.21	Up	0.046891	NA
jing	37954	7.14	6.87	1.21	Down	0.017555	NA
Pka-C1	34284	8.74	8.47	1.2	Down	0.034984	NA
CG42307	7354423	5.62	5.35	1.2	Down	0.005506	NA
FKBP59	38809	5.62	5.35	1.2	Down	0.005506	NA
BtbVII	38376	7.84	7.58	1.2	Down	0.013073	0.51454
ade3	31628	5.94	5.67	1.2	Down	0.049187	NA
Trp1	39524	5.87	5.60	1.2	Down	0.005581	NA
Acer	34189	4.90	4.64	1.2	Down	0.043338	NA
shep	38605	9.40	9.14	1.2	Down	0.014671	0.522769
mir-12	33076	5.13	4.86	1.2	Down	0.02667	NA
CG3808	40074	4.55	4.29	1.2	Down	0.049816	NA
Sr-CI	38003	7.80	7.54	1.2	Down	0.000685	NA
CG42399	31225	5.98	5.72	1.2	Down	0.015245	NA
CG34452	40876	8.65	8.39	1.2	Down	0.025907	NA
alien	34225	6.10	6.36	1.2	Up	0.024126	NA
snRNA:U1:21D	31656	5.59	5.33	1.2	Down	0.044501	NA
Side	41869	4.42	4.68	1.2	Up	0.019455	0.278074
vir-1	32426	6.54	6.28	1.2	Down	0.012518	NA
spen	42345	7.25	6.99	1.2	Down	0.030805	NA
esc	35098	10.30	10.04	1.2	Down	0.03717	0.53406
CG4341	33276	7.93	7.67	1.2	Down	0.021041	0.431609
CG42271	2768892	6.32	6.06	1.2	Down	0.006285	NA
CG10866	38461	2.20	2.46	1.2	Up	0.049036	NA
aop	45467	7.58	7.33	1.19	Down	0.049269	NA
Atg1	42504	6.76	7.01	1.19	Up	0.041476	0.537672
Ntf-2r	35101	3.92	4.18	1.19	Up	0.010746	NA
CG3662	42179	5.28	5.54	1.19	Up	0.028375	0.165713
nol	32077	8.01	7.75	1.19	Down	0.027794	NA
app	39399	6.50	6.25	1.19	Down	0.018482	NA
CG1600	40687	10.58	10.32	1.19	Down	0.00691	NA
CG5543	41247	8.02	7.76	1.19	Down	0.0087	0.507438
wb	35236	5.24	5.49	1.19	Up	0.011216	NA
ns4	35338	5.78	6.03	1.19	Up	0.033897	NA

pr	41592	6.69	6.44	1.19	Down	0.048718	NA
CG2269	36056	9.26	9.01	1.19	Down	0.015012	0.451935
CG30007	38756	10.64	10.40	1.19	Down	0.001514	NA
robo3	33314	7.93	7.69	1.19	Down	0.032163	NA
snoRNA:Psi28S-1180	44018	7.55	7.30	1.19	Down	0.012109	NA
betaggt-II	40972	7.05	7.29	1.19	Up	0.039923	0.434994
dao	34891	5.48	5.23	1.19	Down	0.018119	0.293868
CG34212	40704	7.06	7.31	1.19	Up	0.038359	0.305174
Su(var)2-HP2	42954	7.73	7.48	1.18	Down	0.025537	NA
CG42404	41842	6.86	6.62	1.18	Down	0.047398	NA
CG6209	45021	4.52	4.28	1.18	Down	0.01107	NA
A16	41120	9.56	9.32	1.18	Down	0.006984	NA
CG11658	39319	4.31	4.06	1.18	Down	0.021354	NA
Smg5	34804	7.85	7.61	1.18	Down	0.012911	NA
CG42748	32976	7.42	7.18	1.18	Down	0.009628	NA
Dyb	36362	7.02	6.79	1.18	Down	0.010156	NA
iPLA2-VIA	43728	5.32	5.56	1.18	Up	0.040924	NA
ia2	31795	7.27	7.04	1.18	Down	0.004803	NA
CG42561	42561	7.61	7.38	1.18	Down	0.013864	NA
CG13284	36375	6.43	6.20	1.18	Down	0.00585	NA
CG4502	39135	6.93	6.70	1.18	Down	0.047974	NA
CG11050	42634	5.46	5.69	1.17	Up	0.02701	0.529944
Alr	32991	4.49	4.26	1.17	Down	0.017587	NA
CG9527	38420	8.67	8.44	1.17	Down	0.016645	NA
syd	43905	6.89	6.66	1.17	Down	0.042469	NA
CG31919	40249	7.80	7.57	1.17	Down	0.015213	NA
RpL24	34754	10.36	10.13	1.17	Down	0.001901	NA
CG10260	31247	7.09	6.86	1.17	Down	0.046907	NA
clumsy	32933	5.97	5.74	1.17	Down	0.038029	NA
CG17377	46158	5.88	5.65	1.17	Down	0.001968	NA
Pi3K68D	42793	6.43	6.21	1.17	Down	0.037253	NA
CG32452	318035	4.82	4.60	1.17	Down	0.029157	NA
SoxN	44275	9.52	9.30	1.17	Down	0.023553	0.439892
CG11035	43434	6.52	6.29	1.17	Down	0.037376	NA
CG31352	261629	7.86	7.63	1.17	Down	0.005146	NA
CG8001	38224	5.85	5.63	1.17	Down	0.037784	NA
CG32396	43453	5.90	5.67	1.17	Down	0.012514	NA
CG3156	30994	3.97	3.75	1.17	Down	0.024241	NA
CG13995	33851	5.92	5.70	1.17	Down	0.024341	NA
CG7879	38184	5.84	6.06	1.17	Up	0.003682	0.307188
Fbxl4	32378	5.19	4.97	1.17	Down	0.040393	NA
ken	49713	6.53	6.31	1.17	Down	0.00303	NA
tRNA:K5:84ABb	31489	4.55	4.33	1.17	Down	0.045376	NA

Catsup	37750	4.15	3.93	1.17	Down	0.037343	NA
CG4707	37935	5.38	5.15	1.17	Down	0.034506	NA
CG31665	33361	7.53	7.30	1.17	Down	0.027805	NA
CG7686	36146	7.08	6.86	1.17	Down	0.020465	NA
lwr	31701	5.09	4.87	1.17	Down	0.045416	NA
CG13330	44014	8.14	7.92	1.17	Down	0.014044	NA
CG1600	35687	8.67	8.45	1.16	Down	0.04612	NA
GRHR	33132	7.55	7.33	1.16	Down	0.024127	NA
CG9098	33840	7.31	7.10	1.16	Down	0.032015	NA
Sfp33A2	42473	7.15	6.93	1.16	Down	0.005995	NA
CG31813	31813	6.41	6.20	1.16	Down	0.04682	NA
Hex-t1	32849	4.37	4.15	1.16	Down	0.047447	NA
edl	39588	3.40	3.62	1.16	Up	0.021335	NA
CG8814	41768	6.89	6.67	1.16	Down	0.027794	NA
Acp53Ea	37381	5.90	6.11	1.16	Up	0.042258	NA
CG17129	38080	2.09	1.87	1.16	Down	0.03852	NA
debcl	32430	4.42	4.20	1.16	Down	0.01702	NA
Su(Tpl)	32217	7.00	6.79	1.16	Down	0.016797	NA
CG31357	326135	6.22	6.01	1.16	Down	0.041794	NA
CG40045	3355079	6.64	6.85	1.16	Up	0.016158	0.480176
LSm7	53562	8.46	8.25	1.16	Down	0.043653	NA
CG13343	36539	5.37	5.58	1.16	Up	0.023846	NA
snoRNA:Psi28S-2263	34568	6.57	6.36	1.16	Down	0.007577	NA
nimC4	43277	7.09	6.88	1.16	Down	0.002066	NA
Acp53Ea	37384	6.31	6.10	1.16	Down	0.005883	NA
CG8441	36792	4.26	4.47	1.16	Up	0.032233	NA
CG15765	31513	7.46	7.25	1.16	Down	0.007461	NA
aru	31290	6.04	5.83	1.16	Down	0.037909	NA
psq	36118	8.46	8.25	1.16	Down	0.009171	NA
E2f2	31523	6.42	6.21	1.16	Down	0.029643	NA
CG10253	36669	6.39	6.18	1.16	Down	0.00782	NA
Nhe2	42958	8.78	8.57	1.16	Down	0.027935	NA
CG7149	40654	9.34	9.13	1.16	Down	0.033057	NA
arr	44279	6.96	6.76	1.15	Down	0.049788	NA
Rho1	40792	6.80	6.59	1.15	Down	0.007161	NA
CG31871	32278	6.51	6.31	1.15	Down	0.013947	NA
wah	41911	8.36	8.16	1.15	Down	0.033249	NA
CG3589	42169	8.52	8.31	1.15	Down	0.011013	NA
CG11377	37152	5.33	5.13	1.15	Down	0.021994	NA
rdo	40258	3.66	3.45	1.15	Down	0.00489	NA
Nup54	36360	5.95	5.75	1.15	Down	0.044708	NA
SCAR	34519	7.30	7.10	1.15	Down	0.019055	NA
CG1753	33081	6.22	6.02	1.15	Down	0.025507	NA

Dscam	31050	6.26	6.06	1.15	Down	0.024422	NA
CG4970	32953	10.32	10.12	1.15	Down	0.004274	NA
Cpsf160	41365	6.54	6.34	1.15	Down	0.038497	NA
Gpdh	32379	7.00	6.80	1.15	Down	0.02404	NA
CG13705	48481	5.62	5.82	1.15	Up	0.013055	NA
CG9531	32935	7.34	7.15	1.15	Down	0.015081	NA
CG12042	43901	7.76	7.56	1.15	Down	0.013469	NA
flfl	42267	8.07	7.88	1.15	Down	0.000288	NA
Acp29AB	32561	8.01	7.81	1.14	Down	0.014684	NA
CG13097	34184	5.69	5.50	1.14	Down	0.021774	NA
mdy	32611	2.89	3.09	1.14	Up	0.012586	NA
drl	41343	9.60	9.40	1.14	Down	0.043145	NA
Sec61alpha	42993	4.71	4.91	1.14	Up	0.047558	NA
CG4806	37948	5.45	5.64	1.14	Up	0.037286	NA
aop	40431	8.78	8.59	1.14	Down	0.022144	NA
crol	34592	9.92	9.72	1.14	Down	0.00821	NA
dikar	43576	7.40	7.21	1.14	Down	0.035109	NA
Fsn	33133	4.65	4.83	1.14	Up	0.044879	NA
snoRNA:Me28S-G3255b	40461	7.65	7.46	1.14	Down	0.033262	NA
Catsup	37761	5.39	5.21	1.14	Down	0.035649	NA
loqs	34751	7.57	7.39	1.13	Down	0.042821	NA
trsn	36110	5.00	5.18	1.13	Up	0.049492	NA
lbk	36788	6.73	6.55	1.13	Down	0.012045	NA
CG33969	41443	8.19	8.01	1.13	Down	0.031234	NA
CG16812	34722	6.06	5.88	1.13	Down	0.030237	NA
RpL37A	44783	8.95	8.77	1.13	Down	0.033344	NA
RpL9	45928	8.10	7.92	1.13	Down	0.014404	NA
spir	32854	7.25	7.07	1.13	Down	0.041228	NA
fusl	38943	4.77	4.95	1.13	Up	0.023626	NA
AGO1	43353	6.19	6.02	1.13	Down	0.004653	NA
Jon25Bii	35758	7.17	7.00	1.13	Down	0.015264	NA
CG12194	33685	4.66	4.84	1.13	Up	0.04748	NA
Amy-d	32536	7.10	7.27	1.13	Up	0.024985	NA
Smox	31738	8.53	8.35	1.13	Down	0.014364	NA
CG33013	41398	7.64	7.47	1.13	Down	0.003063	NA
Catsup	37862	7.18	7.01	1.13	Down	0.0036	NA
PNUTS	33526	7.34	7.17	1.13	Down	0.006367	NA
CG42741	40139	4.31	4.48	1.13	Up	0.030533	NA
CG31814	318958	4.19	4.02	1.13	Down	0.034266	NA
CG9304	39688	5.40	5.23	1.13	Down	0.007543	NA
Pvf3	31629	6.23	6.06	1.13	Down	0.039342	NA
PRL-1	34952	7.70	7.87	1.13	Up	0.047632	NA

eIF-4a	33835	11.35	11.18	1.12	Down	0.007014	NA
CG32066	39218	7.05	6.88	1.12	Down	0.031178	NA
sca	36411	6.76	6.59	1.12	Down	0.03484	NA
tRNA:D2:69F	32125	6.54	6.38	1.12	Down	0.011039	NA
CG8833	39535	5.00	4.83	1.12	Down	0.005782	NA
oaf	33435	5.82	5.99	1.12	Up	0.045374	NA
I-2	39156	5.55	5.38	1.12	Down	0.024126	NA
gprs	36862	6.31	6.15	1.12	Down	0.044979	NA
Rca1	38700	7.15	6.99	1.12	Down	0.006886	NA
Poxn	41721	7.89	8.05	1.12	Up	0.037118	0.536973
Dscam	31044	4.55	4.39	1.12	Down	0.020911	NA
emb	34167	8.48	8.64	1.12	Up	0.030563	NA
mir-2c	42936	6.08	5.92	1.11	Down	0.010046	NA
stai	35627	5.26	5.42	1.11	Up	0.003471	NA
Thiolase	32690	7.75	7.60	1.11	Down	0.046568	NA
l(2)37Cb	42808	5.35	5.19	1.11	Down	0.025098	NA
Dek	47906	8.03	8.19	1.11	Up	0.036215	NA
aub	47384	5.77	5.62	1.11	Down	0.017217	NA
CG33464	2768843	8.14	7.99	1.11	Down	0.030078	NA
CG4669	41025	6.78	6.93	1.11	Up	0.040794	NA
tsh	35430	7.04	6.89	1.11	Down	0.023726	NA
CG31883	32616	6.58	6.43	1.11	Down	0.015304	NA
Ced-12	37464	6.94	6.79	1.11	Down	0.021769	NA
Aldh	32114	4.96	4.82	1.11	Down	0.036328	NA
snoRNA:Me28S-C3227b	40739	10.69	10.55	1.11	Down	0.001874	NA
Sir2	40562	6.51	6.36	1.11	Down	0.029894	NA
gpp	31151	10.46	10.32	1.11	Down	0.025169	NA
Opbp	246618	4.79	4.64	1.11	Down	0.012645	NA
CG12567	35509	7.19	7.05	1.11	Down	0.024839	NA
CG7971	38206	9.54	9.40	1.1	Down	0.045289	NA
Acp26Ab	37205	8.45	8.32	1.1	Down	0.04559	NA
Vha16-1	44307	8.71	8.57	1.1	Down	0.014843	NA
snRNA:U5:38ABa	32881	6.06	5.92	1.1	Down	0.04218	NA
mus201	41168	6.75	6.62	1.1	Down	0.043698	NA
tRNA:CR31602	31602	6.90	6.77	1.1	Down	0.018435	NA
snoRNA:Me18S-C1831	44000	8.73	8.60	1.1	Down	0.018979	NA
Gr64c	32256	7.23	7.10	1.09	Down	0.045078	NA
CG4983	40682	6.14	6.27	1.09	Up	0.03334	NA
CG3625	40155	7.94	7.82	1.09	Down	0.01687	NA
rl	41085	6.94	7.06	1.09	Up	0.00371	NA
CG42540	38562	6.33	6.20	1.09	Down	0.006548	NA

CG11866	38400	5.98	5.86	1.09	Down	0.014074	NA
CG10151	36639	6.76	6.64	1.09	Down	0.001704	NA
CG42342	7354466	7.86	7.74	1.09	Down	0.018692	NA
AGO2	39683	8.80	8.92	1.09	Up	0.000791	NA
CG42306	31581	7.09	6.98	1.08	Down	0.010146	NA
Amy-p	32524	5.75	5.64	1.08	Down	0.000328	NA
Egfr	39899	8.02	7.90	1.08	Down	0.025891	NA
His2B:CG17949	32627	7.57	7.69	1.08	Up	0.025126	NA
CG4230	33743	6.15	6.04	1.08	Down	0.003061	NA
Pkn	35950	6.98	6.87	1.08	Down	0.045645	NA
Catsup	37876	6.19	6.30	1.08	Up	0.016836	NA
bur	39099	7.49	7.38	1.08	Down	0.038098	NA
Trl	2768981	9.00	8.89	1.07	Down	0.038693	NA
cutlet	44637	4.27	4.37	1.07	Up	0.045913	NA
CG12907	44548	10.85	10.75	1.07	Down	0.021864	NA
alpha4GT1	38744	6.80	6.70	1.07	Down	0.045622	NA
CkIIalpha-i3	43130	6.60	6.50	1.07	Down	0.013431	NA
crc	32941	7.86	7.76	1.07	Down	0.001983	NA
lama	42265	7.94	7.85	1.07	Down	0.039376	NA
RpL19	37995	10.77	10.68	1.07	Down	0.039261	NA
Thor	32557	6.39	6.30	1.07	Down	0.00368	NA
snoRNA:Me28S-G2596	5740396	6.94	7.03	1.07	Up	0.04682	NA
snmRNA:184	3772671	6.94	7.03	1.07	Up	0.04682	NA
CG1371	36053	7.32	7.40	1.06	Up	0.04553	NA
CG17612	39935	6.37	6.29	1.05	Down	0.039049	NA
zf30C	34292	8.07	8.00	1.05	Down	0.048358	NA
cg	36571	8.51	8.46	1.04	Down	0.031872	NA
CG7248	40933	7.19	7.15	1.03	Down	0.011376	NA

Terms and abbreviations: Gene_Identifier, the flybase symbol of the corresponding differentially expressed gene, control_mean, the mean expression level of D42>LacZ, overexpression_mean, the mean expression level of D42>TBPH, Ratio, the fold-change of expression in D42>TBPH compared to D42>LacZ, Direction, the direction of the change, p_value, the genesifter p value (see methods for parameters), edgeR p value, the p value calculated in edgeR for the corresponding gene, also adjusted for multiple hypothesis testing (Benjamini & Hochberg, 1995). NA, the gene was not differentially expressed using edgeR analysis.
