# Supplementary material for: Comparison of Parallel High-Throughput RNA Sequencing Between Knockout of TDP-43 and Its Overexpression Reveals Primarily Nonreciprocal and Nonoverlapping Gene Expression Changes in the Central Nervous System of Drosophila
Source: G3 (Bethesda). 2012 Jul 1;2(7):789–802. doi: 10.1534/g3.112.002998 (PMC3385985; doi:10.1534/g3.112.002998)
Supplement: Supporting Information [file supp_2.7.789_TableS3.pdf]

Table S3 List of 26 genes whose expression changed in both loss-of-function (LOF) and gain-of-function (GOF) genotypes and that contained putative TBPH binding sites.

Gene_Identifier	LOF	Ratio	p_value	GOF	Ratio	p_value
CG9812	+	2.96	0.00975	-	1.86	0.000189
Irc	+	2.67	0.010363	-	2.43	0.04415
wgn	+	1.67	0.001873	-	1.16	0.047447
plx	-	1.65	0.024184	+	1.19	0.038359
CG10249	+	1.56	0.030863	-	1.3	0.035681
CCHa2r	+	1.45	0.035987	-	1.51	0.003644
ltl	+	1.45	0.029087	-	1.27	0.03219
CG18210*	-	1.44	0.027657	-	1.55	0.044803
CG17211	-	1.39	0.008802	+	1.27	0.025019
stck	+	1.39	0.003959	-	1.35	0.041068
CG12065*	+	1.36	6.94E-05	-	1.24	0.024021
Tbh	+	1.3	0.009052	-	1.73	0.028432
CG3902	+	1.25	0.0101	+	1.51	0.017083
ush	+	1.22	0.015133	-	1.25	0.045819
CG13784	+	1.2	0.016125	-	1.33	0.022977
l(1)G0289	+	1.2	0.019807	-	1.23	0.032315
CG1578	+	1.2	0.030473	-	1.21	0.024332
CG9009	-	1.19	0.033497	-	1.2	0.012518
cora	+	1.19	0.00021	-	1.1	0.04559
CG4577	-	1.18	0.008905	-	1.23	0.02946
dome	+	1.18	0.018949	-	1.18	0.009628
Smox	+	1.16	0.044535	-	1.13	0.014364
CG13995	+	1.16	0.036236	-	1.17	0.024341
CG13928	-	1.16	0.011025	-	1.21	0.003165
Hmgs	+	1.15	0.022268	-	1.21	0.030339
CG34353	+	1.15	0.041968	-	1.3	0.047338

LOF Ratio, TBPH[G2] knockout expression relative to control A1 strain; GOF Ratio, D42-GAL4>UAS-TBPH expression relative to D42-GAL4>UAS-LacZ.
