# Supplementary material for: Comparison of Parallel High-Throughput RNA Sequencing Between Knockout of TDP-43 and Its Overexpression Reveals Primarily Nonreciprocal and Nonoverlapping Gene Expression Changes in the Central Nervous System of Drosophila
Source: G3 (Bethesda). 2012 Jul 1;2(7):789–802. doi: 10.1534/g3.112.002998 (PMC3385985; doi:10.1534/g3.112.002998)
Supplement: Supporting Information [file supp_2.7.789_TableS4.pdf]

Table S4 Entrez gene identifiers for all genes used in Ontology analyses.

A: Entrez identifiers of DE in mutant , rescued or partially rescued, contains TBPH bind site (181 genes)

37981,3772221,42541,31001,37555,36340,39441,43662,33814,35554,36673,33135,32976,32080,36616,35731,43895,39106,31105,44736,42059,38490,39990,44936,37196,32469,318243,40403,33386,37546,36862,44544,43844,37788,37435,39485,37786,648
75,41059,39583,31964,37940,33225,37892,36930,39056,38273,31542,34554,42891,39017,37012,42581,31965,3772232,38976,38468,39284,33363,45040,37781,31408,32349,43563,47260,42015,41843,43265,47173,32009,41121,42477,37070,35181,33292,42
226,34826,32703,33807,40382,43579,39567,31767,38845,40170,38959,39530,43178,2768881,34093,37798,40835,32994,31201,31157,31795,32943,3346227,40633,32243,40666,37882,41605,33783,42429,35911,37401,34003,43800,37886,31842,40059,3204
5,48581,39311,38104,38195,35286,44317,36782,36383,31955,43950,44154,36896,44573,42049,40102,32321,37583,40229,40590,33291,31525,41360,40342,34002,45012,39054,31332,39860,42880,47767,3772312,36232,36048,35494,42747,39903,34114,325
79,40605,31130,31016,32135,33148,44497,39390,3346207,40330,38610,35620,40754,43765,32037,31718,35730,37119,31212,37661,36634,32083,32087,32273,33299,38484,40310,32822,32724,31980,39389,38255,42105,34969,42216,32447,34767,33953,43
560,40955,40999,35765,43676,38545,43756,37709,37652,43440,34170,43630,36030,326116,43223,33851,318721,31214,34835,42467,318916,41320,34639,34803,39543,42881,41266,32836,3346167,3346238,35507,32849,42547,41309,31873,32217,42707,3
6712,39132,53425,44679,33991,33765,36694,40912,45880,5740840,31451,44660,42702,36395,37755,117369,38017,39518,37161,41864,38895,40205,42066,35997,44258,39901,37132,31798,45380,40359,31309,31381,37310,37011,39049,37567,40959,5344
6,41674,37757,31839,30991,31612,47729,40305,41035,42527,3346150,36595,31402,3885573,31386,40549,42940,31588,3885598,31792,36681,50350,33928,5740318,32656,5740349,31591,34560,35737,50253,42832,38994,32576,33638,38434,32955,36248,
40879,44297,40922,36084,40335,42600,31687,42595,34181,32799,36513,32775,32098,37679,40056,31490,33283,41095,36415,44150,37205,43683,45922,34068,41677,36753,38059,42455,5740224,39090,39730,32337,35135,41840,33260,35535,39065,4313
6,37521,42530,45234,3771985,40876,32780,42411,40715,39954,32343,36496,40703,40704,37007,37419,32056,34993,41045,36603,43562,42196,38262,33495,36488,38222,32123,35444,35697,36121,38731,31441,32426,5740176,3771796,3772559,33014,31
666,31738,45875,36668,31229,34287,33556,35293,39873,39875,317913,53437,36526,38589,40180,35768,37129,42148,44359,48311,2768945,42836,318743,43197,41248,5740590,41062,5740551,4379891

Conserved with Sephton *et al*., (2011, 109 genes):

34731,42049,39567,40701,36371,41017,37619,4379858,47878,34015,40427,35525,36330,40205,37709,39748,32148,5740462,42879,31718,35284,41819,35094,35866,40424,43235,36740,38305,31502,36301,32469,43751,32028,34740,38352,39317,36237,34
131,40059,32401,35535,32930,35984,31344,40049,32468,42835,31429,41616,34878,43551,33339,42339,39962,33432,38337,31729,37882,41473,34648,33765,38954,34732,39611,34850,36945,37884,33692,38523,34112,44126,40999,35949,43916,32606,334
6208,32233,39939,34003,35396,38194,38657,34779,3355133,41578,3771796,3772559,34341,36208,35077,41455,31610,36668,38629,5740590,45970,37132,43682,48903,2768670,50320,31953,35004,38845,36072,34639,34594,31970,41644,36924,40794,365
94,34987,3772109,33534,3771738,3771965,35177,41376,33225,37978,36147,42402,31798,32970,31565,40948,36643,33108,31964,48421,42295,42110,33291,38978,42303,39732,45931,34912,41491,37601,44154,32373,39686,38716,32135,326116,31658,37
781,38195,34286,37954,34284,38376,31628,38605,31225,40876,32426,33276,2768892,45467,42504,32077,39399,41247,41592,36056,38756,33314,40704,41842,39319,34804,32976,36362,43728,31795,42561,43905,40249,31247,44275,261629,38224,43453,
30994,33851,37935,33361,44014,33132,42473,31813,32849,39588,32217,53562,43277,37384,31513,31290,36118,31523,42958,44279,40792,32278,41911,42169,37152,34519,33081,31050,32953,48481,43901,42267,32561,41343,40431,34592,40461,36788,4
1443,34722,45928,32854,43353,35758,32536,31738,41398,33526,318958,34952,36411,36862,38700,34167,42936,35627,32690,47906,35430,32616,32114,31151,35509,37205,44307,41168,31602,44000,32256,40155,41085,38562,7354466,31581,39899,3374
3,35950,39099,2768981,44548,38744,43130,32941,37995,36571

B: DE in overexpression, contains TBPH binding site (270 genes)

34731,42049,39567,40701,36371,41017,37619,4379858,47878,34015,40427,35525,36330,40205,37709,39748,32148,5740462,42879,31718,35284,41819,35094,35866,40424,43235,36740,38305,31502,36301,32469,43751,32028,34740,38352,39317,36237,34
131,40059,32401,35535,32930,35984,31344,40049,32468,42835,31429,41616,34878,43551,33339,42339,39962,33432,38337,31729,37882,41473,34648,33765,38954,34732,39611,34850,36945,37884,33692,38523,34112,44126,40999,35949,43916,32606,334
6208,32233,39939,34003,35396,38194,38657,34779,3355133,41578,3771796,3772559,34341,36208,35077,41455,31610,36668,38629,5740590,45970,37132,43682,48903,2768670,50320,31953,35004,38845,36072,34639,34594,31970,41644,36924,40794,365
94,34987,3772109,33534,3771738,3771965,35177,41376,33225,37978,36147,42402,31798,32970,31565,40948,36643,33108,31964,48421,42295,42110,33291,38978,42303,39732,45931,34912,41491,37601,44154,32373,39686,38716,32135,326116,31658,37
781,38195,34286,37954,34284,38376,31628,38605,31225,40876,32426,33276,2768892,45467,42504,32077,39399,41247,41592,36056,38756,33314,40704,41842,39319,34804,32976,36362,43728,31795,42561,43905,40249,31247,44275,261629,38224,43453,
30994,33851,37935,33361,44014,33132,42473,31813,32849,39588,32217,53562,43277,37384,31513,31290,36118,31523,42958,44279,40792,32278,41911,42169,37152,34519,33081,31050,32953,48481,43901,42267,32561,41343,40431,34592,40461,36788,4
1443,34722,45928,32854,43353,35758,32536,31738,41398,33526,318958,34952,36411,36862,38700,34167,42936,35627,32690,47906,35430,32616,32114,31151,35509,37205,44307,41168,31602,44000,32256,40155,41085,38562,7354466,31581,39899,3374
3,35950,39099,2768981,44548,38744,43130,32941,37995,36571

Conserved (85 genes):

40424,38352,32930,35984,42835,34878,39962,34850,36945,33692,38523,35949,43916,32233,38194,36208,5740590,48903,35004,34594,3772109,37978,32970,36643,31964,48421,42303,39732,45931,34912,41491,44154,39686,326116,37781,37954,34284,3
1628,2768892,42504,41247,38756,39319,43728,42561,43905,40249,31247,261629,38224,30994,44014,42473,39588,32217,43277,31290,44279,40792,32278,31050,43901,32561,34592,36788,45928,32854,35758,31738,41398,34167,42936,32616,31151,3550
9,37205,31602,32256,41085,31581,39899,35950,43130,37995,36571

C: Controls (7897 genes)

45524,35351,35946,42257,41259,39626,317923,31503,33274,38015,32419,37302,43671,47878,39529,40916,31162,317915,32034,40020,3771872,3772576,31674,34191,34383,38495,38818,39096,43820,45880,33292,37644,33635,40963,40268,37991,36348,
38638,318129,33324,37131,43227,38470,35211,43126,32778,36595,36838,33659,42787,41403,41031,318098,42339,37015,43593,36532,38473,35911,39795,37941,37590,43184,36806,33749,34894,36615,31641,38180,318135,41339,43177,43067,41036,320
36,41640,41368,43413,38432,246458,246608,50442,41160,37929,41155,41520,32610,34627,39333,42002,40345,43183,39075,33474,33874,32199,38058,37128,32327,34317,33569,318956,261621,32592,39232,31358,32401,31923,34037,39020,32399,37469,
36596,34679,43440,37004,37709,31408,43136,41831,5740766,326156,34127,36865,39582,35913,5740276,38741,32299,34819,43867,41297,32329,32148,45879,31591,42788,5740455,34565,318136,37614,4379858,318160,37295,35047,42426,261611,36573,
35380,3772574,3772566,40205,32035,37458,42581,40004,35735,36958,44887,42970,42432,31898,246587,3772393,3772088,33839,37182,35708,41202,326170,36740,40266,34038,38583,37534,33349,43583,36011,31340,34732,36016,32154,37229,39740,36
244,41887,38484,38501,43895,42530,45326,50350,32285,7354475,50001,35886,33657,38434,261626,39056,33934,37619,317953,38296,38297,33432,41513,39752,36601,42049,37435,40088,32087,33170,39748,43243,249663,39165,317907,40612,32353,50
044,31111,43275,31650,35745,31656,39071,35013,37949,33200,42981,33727,261623,42930,5740424,39954,35989,36924,40666,45396,39543,42455,3772646,3771806,36243,32488,326116,5740275,3354987,41308,38507,44013,36634,34786,36586,39055,31
8825,317857,35875,36849,50466,3346150,317852,38719,37940,246442,44058,34673,45875,35090,33641,38410,39264,37575,36501,7354376,7354377,7354378,7354381,31103,34322,40598,37545,40071,40918,39048,43530,35746,41518,33726,34260,33534,
38183,41767,317877,43551,41967,38972,33718,39049,39991,35513,41134,42893,37227,35417,39077,37748,41851,35104,318799,250710,34827,31099,41452,42697,43756,326264,42940,36076,35940,32283,246651,41144,38942,40051,39396,3355069,57401
95,42361,32694,33386,31417,59239,34117,39567,37106,38994,36957,38663,44098,34639,36081,41919,41221,35396,40083,34289,41033,40333,3772042,34611,37083,32680,37399,36292,32821,37166,40450,31718,41473,39231,34176,45780,31094,34351,41
252,43043,5740596,38443,34878,37887,40897,39517,39085,42335,32326,37294,40524,31136,41605,318631,38751,38813,39422,36668,246386,37560,41222,34530,32426,30972,31858,40492,41248,32093,38954,37822,31251,38610,44910,41070,38290,3606
0,36528,39237,38517,35264,33695,31402,43429,42676,35061,32672,41238,41112,35231,42560,36550,35622,40287,31743,33047,246573,41982,35687,5740325,31212,42242,41381,37219,42683,42860,39317,33540,42723,38104,37147,36009,37548,36753,45
588,39106,32558,38315,40608,59261,41044,32874,36965,42701,38472,44010,37663,326141,37137,34181,41821,34067,32469,246595,41278,40732,318251,31497,46391,34477,2768870,38518,36396,40102,43582,42216,318573,5740503,40713,31385,34623,
46386,34237,41894,40059,42832,40279,49090,40085,32724,43748,36588,41136,3346141,36651,40922,32072,31021,31198,2768992,3346208,246620,42801,41469,40816,35126,31391,42294,4379889,34064,42523,34087,3885628,2768998,246514,44297,404
91,117366,33613,32767,38390,42576,43014,38869,33022,2768668,42280,31055,318992,43825,43318,36368,34023,38889,326109,318203,41746,140439,32296,31695,31461,43414,40579,34645,318930,40001,38305,42890,39138,35180,32053,49077,246420,
36427,40031,2768965,42078,40575,45706,33354,35284,37223,33812,4379917,32476,43707,38252,50381,246533,38235,34152,318260,36776,47253,42783,40180,35221,46085,38454,37999,32665,35125,44057,7354414,50395,35153,32098,35620,43805,3591
6,318252,3355048,3772540,36862,41148,42527,38364,34652,40156,37376,40721,32064,40373,34952,31526,34792,35656,31843,59178,34528,42134,37641,41394,34106,42851,40486,34981,50402,50149,246511,37028,31480,32980,32354,31606,40153,4282
9,37697,42762,246582,34440,32620,34598,41191,33931,35554,34039,42477,35291,31326,42356,40422,43104,31729,38906,3346202,41742,42390,38337,38502,39854,33953,32272,53558,41626,3346192,46032,33530,35984,32447,42295,34961,42702,43324,
35003,37025,36017,38535,39139,31949,31857,39316,39550,36379,2768718,31186,42061,32549,37134,2769000,31777,37567,38012,35271,34490,32748,35704,39389,43514,40703,39871,3355016,31550,35309,36392,37968,31189,36284,37648,35238,43506,
43608,37445,53446,30988,42045,34090,39504,32251,41863,43326,31509,34184,39262,43002,39383,3772691,3771923,41157,37453,42929,31070,33284,48373,39306,32504,37985,38731,41017,34485,36248,41726,39409,31520,31935,5740349,59163,32485,
318031,39461,32626,34577,41758,34505,31702,7354433,31761,34434,30983,39238,33308,33600,34839,41644,5740879,5740394,34625,33347,33705,318224,40429,41820,43577,2769004,35353,3885598,31169,3346207,37342,36712,41450,37783,33476,409
31,41771,38957,37191,41937,40619,33175,32850,32079,44831,37944,36757,44858,3346227,43256,32135,5740347,44258,42620,31056,41396,36197,36909,32532,33700,326201,50003,44002,37429,33230,36364,33556,50170,36268,41480,35369,41300,3904
4,41384,32244,43483,35987,42003,31614,42564,41387,Entrez_ID,45524,35351,35946,42257,41259,39626,317923,31503,33274,38015,32419,37302,43671,47878,39529,40916,31162,317915,32034,40020,3771872,3772576,31674,34191,34383,38495,38818,3
9096,43820,45880,33292,37644,33635,40963,40268,37991,36348,38638,318129,33324,37131,43227,38470,35211,43126,32778,36595,36838,33659,42787,41403,41031,318098,42339,37015,43593,36532,38473,35911,39795,37941,37590,43184,36806,33749,
34894,36615,31641,38180,318135,41339,43177,43067,41036,32036,41640,41368,43413,38432,246458,246608,50442,41160,37929,41155,41520,32610,34627,39333,42002,40345,43183,39075,33474,33874,32199,38058,37128,32327,34317,33569,318956,26
1621,32592,39232,31358,32401,31923,34037,39020,32399,37469,36596,34679,43440,37004,37709,31408,43136,41831,5740766,326156,34127,36865,39582,35913,5740276,38741,32299,34819,43867,41297,32329,32148,45879,31591,42788,5740455,34565,
318136,37614,4379858,318160,37295,35047,42426,261611,36573,35380,3772574,3772566,40205,32035,37458,42581,40004,35735,36958,44887,42970,42432,31898,246587,3772393,3772088,33839,37182,35708,41202,326170,36740,40266,34038,38583,37
534,33349,43583,36011,31340,34732,36016,32154,37229,39740,36244,41887,38484,38501,43895,42530,45326,50350,32285,7354475,50001,35886,33657,38434,261626,39056,33934,37619,317953,38296,38297,33432,41513,39752,36601,42049,37435,4008
8,32087,33170,39748,43243,249663,39165,317907,40612,32353,50044,31111,43275,31650,35745,31656,39071,35013,37949,33200,42981,33727,261623,42930,5740424,39954,35989,36924,40666,45396,39543,42455,3772646,3771806,36243,32488,326116,
5740275,3354987,41308,38507,44013,36634,34786,36586,39055,318825,317857,35875,36849,50466,3346150,317852,38719,37940,246442,44058,34673,45875,35090,33641,38410,39264,37575,36501,7354376,7354377,7354378,7354381,31103,34322,40598
,37545,40071,40918,39048,43530,35746,41518,33726,34260,33534,38183,41767,317877,43551,41967,38972,33718,39049,39991,35513,41134,42893,37227,35417,39077,37748,41851,35104,318799,250710,34827,31099,41452,42697,43756,326264,42940,36
076,35940,32283,246651,41144,38942,40051,39396,3355069,5740195,42361,32694,33386,31417,59239,34117,39567,37106,38994,36957,38663,44098,34639,36081,41919,41221,35396,40083,34289,41033,40333,3772042,34611,37083,32680,37399,36292,3
2821,37166,40450,31718,41473,39231,34176,45780,31094,34351,41252,43043,5740596,38443,34878,37887,40897,39517,39085,42335,32326,37294,40524,31136,41605,318631,38751,38813,39422,36668,246386,37560,41222,34530,32426,30972,31858,404
92,41248,32093,38954,37822,31251,38610,44910,41070,38290,36060,36528,39237,38517,35264,33695,31402,43429,42676,35061,32672,41238,41112,35231,42560,36550,35622,40287,31743,33047,246573,41982,35687,5740325,31212,42242,41381,37219,4
2683,42860,39317,33540,42723,38104,37147,36009,37548,36753,45588,39106,32558,38315,40608,59261,41044,32874,36965,42701,38472,44010,37663,326141,37137,34181,41821,34067,32469,246595,41278,40732,318251,31497,46391,34477,2768870,38
518,36396,40102,43582,42216,318573,5740503,40713,31385,34623,46386,34237,41894,40059,42832,40279,49090,40085,32724,43748,36588,41136,3346141,36651,40922,32072,31021,31198,2768992,3346208,246620,42801,41469,40816,35126,31391,4229
4,4379889,34064,42523,34087,3885628,2768998,246514,44297,40491,117366,33613,32767,38390,42576,43014,38869,33022,2768668,42280,31055,318992,43825,43318,36368,34023,38889,326109,318203,41746,140439,32296,31695,31461,43414,40579,34
645,318930,40001,38305,42890,39138,35180,32053,49077,246420,36427,40031,2768965,42078,40575,45706,33354,35284,37223,33812,4379917,32476,43707,38252,50381,246533,38235,34152,318260,36776,47253,42783,40180,35221,46085,38454,37999,
32665,35125,44057,7354414,50395,35153,32098,35620,43805,35916,318252,3355048,3772540,36862,41148,42527,38364,34652,40156,37376,40721,32064,40373,34952,31526,34792,35656,31843,59178,34528,42134,37641,41394,34106,42851,40486,34981
,50402,50149,246511,37028,31480,32980,32354,31606,40153,42829,37697,42762,246582,34440,32620,34598,41191,33931,35554,34039,42477,35291,31326,42356,40422,43104,31729,38906,3346202,41742,42390,38337,38502,39854,33953,32272,53558,41
626,3346192,46032,33530,35984,32447,42295,34961,42702,43324,35003,37025,36017,38535,39139,31949,31857,39316,39550,36379,2768718,31186,42061,32549,37134,2769000,31777,37567,38012,35271,34490,32748,35704,39389,43514,40703,39871,33
55016,31550,35309,36392,37968,31189,36284,37648,35238,43506,43608,37445,53446,30988,42045,34090,39504,32251,41863,43326,31509,34184,39262,43002,39383,3772691,3771923,41157,37453,42929,31070,33284,48373,39306,32504,37985,38731,41
017,34485,36248,41726,39409,31520,31935,5740349,59163,32485,318031,39461,32626,34577,41758,34505,31702,7354433,31761,34434,30983,39238,33308,33600,34839,41644,5740879,5740394,34625,33347,33705,318224,40429,41820,43577,2769004,35
353,3885598,31169,3346207,37342,36712,41450,37783,33476,40931,41771,38957,37191,41937,40619,33175,32850,32079,44831,37944,36757,44858,3346227,43256,32135,5740347,44258,42620,31056,41396,36197,36909,32532,33700,326201,50003,44002
,37429,33230,36364,33556,50170,36268,41480,35369,41300,39044,41384,32244,43483,35987,42003,31614,42564,41387,34143,39726,43362,4379851,35131,37132,42391,33647,37556,117300,41272,42180,39051,41092,3885637,41455,41710,43872,39564,3
4145,32822,35261,36789,41159,42481,32799,33656,40252,31990,43408,39738,34303,42429,41124,42992,35535,36121,3355171,37126,38334,38322,32253,326171,7354403,40999,318053,41823,41877,43521,37882,318938,36012,37246,34356,35585,34226,
40137,33793,49893,32682,36688,246551,40228,40923,32076,33332,35728,32723,31193,43265,39320,33865,41625,43782,43697,40330,117369,31648,42505,40346,39065,30970,48421,40543,33299,32991,43997,3771877,3772432,39559,39294,39393,31880,
43268,35877,35681,38600,39964,326256,43504,30992,40263,36258,34873,40339,45845,38823,40288,36428,39070,40552,39444,246599,41261,37149,38036,37558,33783,42507,44750,33858,32881,34882,39907,31020,33639,43918,5740699,34752,41229,32
144,33924,5740318,32775,30994,41677,37861,42657,32792,36848,34114,2768882,38080,41994,42810,43034,34044,32849,42947,35220,43590,35255,43213,40562,43797,43780,43999,43244,33079,41032,43497,38857,5740282,36454,33780,36762,35068,42
028,36232,42310,2768908,41225,37947,33901,34018,3772603,3772383,43155,38362,3772100,43353,43843,42750,318246,2768976,44446,36488,38911,34543,33765,2768945,41149,40128,40309,37413,40997,34429,33085,41579,37514,38192,48440,42503,3
7890,41807,41002,32468,40669,31498,35901,35094,31572,39667,32267,38895,31502,34171,50320,36383,35832,318096,33501,42206,43193,38917,32813,40871,33844,39566,35310,42622,35525,39999,34109,43098,39774,40348,45285,33826,35043,41420,40016,3
0991,38622,40935,40430,39530,32348,43936,31463,44510,42670,37036,32676,31794,37954,36466,31436,33461,37849,37834,34115,33571,3355011,31249,36976,35878,33190,39486,33179,31558,36051,35322,40833,31536,32075,40745,37937,36656,40555,
37531,38900,36626,35299,2768977,47852,42465,43070,318003,45884,39947,32216,39004,46637,42547,33363,41345,38720,40363,32619,39382,33515,33377,35546,33927,43009,35567,35568,33606,32864,42645,39518,38102,40720,36272,246637,39514,32
166,43149,32489,39516,40356,42014,30979,44498,31168,39078,43777,39129,35111,41768,38693,37520,3355084,42551,246534,42589,37708,39581,33520,42920,31237,36547,47065,50416,40056,3355132,35480,35828,31766,34251,38147,41318,38666,409
53,3772376,35004,38273,45248,326246,44432,43453,41445,40512,39539,43747,42210,38614,36333,35768,36536,38121,32581,31896,34304,2768877,37563,38445,40427,37905,5740462,38496,33759,44392,48571,42588,31943,39989,37419,39156,32958,38
331,326235,50068,38003,34259,41809,39950,31507,34779,40271,38461,41708,37807,36920,38095,41428,42835,36084,37017,50302,33028,34395,44018,33221,5740840,32375,49241,38941,3771779,36868,40961,32397,32994,32081,38879,40009,31728,359
96,36127,43347,36829,37107,38089,31469,33923,42126,326217,34749,36705,39730,42066,43040,246389,41502,40860,43069,40671,41290,32293,35174,33351,37089,318751,41027,34116,45908,246473,39916,36135,42634,40160,40681,42112,36279,31963,
42018,41676,192528,37129,35888,35077,41822,42271,39068,32545,36283,36057,41030,46717,2769003,37232,38810,36246,43015,38182,33909,7354423,38809,33487,39777,39221,42166,41250,40335,43450,40124,43081,35604,42694,34068,32921,37888,4
1547,35885,40400,41121,31005,40042,38522,39252,38615,38657,326180,43630,42245,37037,40826,40019,36438,33908,318986,35933,36652,35689,318035,35898,37555,36202,47384,32342,40061,43875,35941,35694,33133,34634,32788,38407,38029,3699
1,31002,35910,34122,2769005,43092,40551,40644,40706,31178,33882,31934,35190,35424,37364,45922,5740299,33440,38594,32320,41985,326329,42350,43392,32873,45041,41772,31980,35122,35383,34406,31270,31038,31644,36770,39441,34275,35234,
36508,40282,36985,48844,38887,43033,318916,40982,44005,44227,31317,43099,45021,43904,42831,34368,31622,34386,31808,43127,43699,32967,5740816,37412,31406,38624,32319,42877,40241,42203,35124,246650,34435,41000,36030,35107,35408,57
40629,31447,34419,59128,50191,31120,35055,42396,39572,34310,3772580,3772419,36551,34947,47220,35016,38581,43786,40592,35692,41344,38976,44291,38382,43046,40599,31194,32267,39508,35571,40522,38805,3772483,34180,31010,5740446,4051
3,33117,32479,40293,31721,7354383,41254,35918,31429,40690,326208,40704,32037,31736,34591,49580,246488,31126,34998,31794,39426,40342,35773,47611,41153,36526,34621,37791,2769008,43223,3355093,33742,38085,41170,43746,36174,33959,5
0438,34837,40981,31962,38562,36814,35531,37331,35374,31434,34189,318977,42001,40640,43651,41721,33248,39476,34774,37564,35277,40928,39172,35807,34538,39098,40167,38845,31547,317891,41354,43168,39941,5740544,2768669,45249,246467,
37122,40921,37884,39675,32229,43020,43503,43819,39304,31734,35266,34186,35344,31424,41955,31339,33257,31696,37016,42340,34030,35937,48445,40340,41991,53579,37030,40002,49425,40965,43562,5740712,36481,50236,40521,3346176,36758,3
1704,31855,33277,41697,37806,38723,47173,32534,41961,33711,31701,35037,44856,41293,38596,38298,36335,35786,38955,33561,34716,41361,32753,40222,33688,41704,36533,32028,44787,40193,31831,35156,40349,41971,36375,64877,34951,4379850,
37174,32606,39643,5740224,43579,33467,2768673,5740142,41174,42351,41583,43607,41034,38463,42187,32574,43682,32151,41309,41601,43535,42160,44817,32495,35615,42289,3771796,3772559,32761,40858,39996,34680,39876,3772670,3771960,3
772109,2768920,42883,33231,33626,34560,38814,36641,35029,40374,37571,39976,36654,42300,33399,37867,37712,59150,42226,32322,33285,34483,40323,39023,43729,35699,42784,34912,40280,42004,32324,59223,41990,37879,42298,40130,35866,323
23,40873,38539,44095,36577,39682,32483,42733,37188,42268,50364,40325,42433,3771968,39034,34005,31190,30976,50266,31610,40296,43427,37355,35357,326240,43869,246452,35800,32211,42608,42162,34953,3771920,33431,33141,318833,47729,41
417,37701,34762,248194,318587,251984,32201,33814,31724,33599,40613,37964,31810,31322,35793,53431,35926,41760,31491,41009,40689,35042,32215,39097,35138,33403,33976,33291,33565,37524,33161,34908,32002,34013,37136,39652,3772165,413
06,31382,41163,44289,40147,32586,42865,32986,33361,42037,42956,43610,34121,31540,44448,41882,33957,35970,36395,37874,35158,38992,40587,37135,38101,41242,31965,38543,33935,32629,33952,37173,34963,36996,4379884,43620,41703,53433,34
355,34772,32972,47408,41650,43447,32498,36692,36886,38222,37825,36768,48973,34118,38084,38771,33776,34544,34891,40080,33861,41720,32645,35145,39390,31007,39775,37285,48312,2768881,38050,37818,318193,32120,31185,35528,35118,32865,
31266,35260,34028,32343,32857,33207,41366,7354406,7354408,7354407,40221,41941,31964,34011,42127,41826,44669,39762,35181,32011,36227,32165,37304,36713,38088,37009,5740407,38428,41302,33545,31295,43078,31967,41046,326151,41359,381
96,34412,33193,45815,40152,43490,35296,43991,32677,42807,41311,31873,39132,40988,38038,31744,35338,37332,37368,31441,39939,38181,35227,49892,31912,40471,48986,43990,40567,39336,35623,38357,33197,41192,31780,42189,45234,38306,3901
9,34091,39052,33016,38735,7354399,42359,39629,40872,31384,33703,35753,40257,39813,43029,31839,40862,48441,38464,42594,41671,44154,36905,37245,38169,3346160,37422,44007,34731,36039,34131,33855,35608,32963,37707,34893,36086,31451,3
17825,32088,36623,31380,35340,31581,38612,46035,30995,43800,38069,49803,4379885,53471,36403,39050,50169,39028,38203,318981,41207,40637,37012,36916,39933,34630,41023,37244,38520,37839,39930,40941,38515,41086,7354410,7354408,73544
11,37470,43672,41733,41866,34840,34945,36687,36593,41612,36045,35691,43500,36307,40003,32461,33391,37516,31642,42269,37815,35376,31944,5740790,42463,33975,33425,31568,35683,41040,41571,40310,30998,31320,42690,317926,32742,42362,4
1231,41448,42619,38381,33578,39458,32224,42454,36960,2768719,40254,31023,43505,36702,3771983,3772180,44783,36990,36046,41701,40404,36592,43614,2768858,38392,3772007,32726,318644,41103,43102,41521,37075,35943,50373,32281,42737,3
6778,40724,33234,41552,246439,36792,59247,40948,33644,39053,31054,32903,43831,43317,34268,36644,44054,40794,2768999,33743,39965,37181,33692,31585,42105,38233,38747,34277,32398,41407,33960,326267,42341,42292,39310,38097,2768688,4
1817,39656,42502,44830,42060,42244,41355,34414,41305,34965,41816,43689,40725,43604,33601,246602,50007,41687,48971,33519,40035,31560,34753,42669,42077,5740672,32766,31837,42519,42473,32509,41194,318566,39527,246552,31489,3772064,
3772218,38261,36541,40780,42079,39457,37141,118436,34537,44108,39101,326120,42687,33920,33401,37407,32516,38922,41549,33077,37305,43074,39079,42148,33398,31903,34633,317887,32868,43425,44936,41797,42492,34631,37119,35709,33224,3
1683,33046,31490,3355133,36694,39388,32435,38500,53436,42597,318935,31383,31682,326146,35575,42939,34138,41376,53585,42958,41989,41062,33847,35177,38302,39736,31268,34323,33609,40095,38989,41035,33294,33455,41789,35817,326199,37
71738,3771965,36887,35035,318753,38750,319028,32497,43435,41875,42109,44441,37926,35505,37187,43738,31397,31236,36754,31848,38457,31267,34307,36782,33112,250736,32636,41869,42385,37960,39364,36459,42905,36643,38629,2768687,39888

,33781,39054,40314,36240,40220,41647,31913,38034,34529,38197,40402,38201,38456,33610,40145,35060,39432,44315,35193,39879,34110,41288,33902,40258,35256,35781,31013,3772182,40509,36157,33222,45816,318103,318682,31806,47942,39293,40
649,37161,34003,3771966,3772677,31205,34701,42859,42964,34720,39690,43006,43373,31613,42954,32382,38346,36496,36253,35880,40620,318790,43570,3355116,7354421,31155,32449,36109,35382,32531,34089,42491,5740281,40389,42925,38087,389
44,36889,5740205,43499,31081,38325,42821,32943,37306,41351,39406,326115,41419,34879,36041,31603,40695,36156,32591,42497,319046,44748,41364,31139,32507,36457,53425,37218,39093,38716,32009,317932,40054,36743,35073,39066,36107,3672
5,31915,43436,43789,37198,5740536,32526,41575,38533,36954,37776,37918,42885,38545,35986,31045,38379,41825,40679,41694,35253,47895,37770,32698,39041,32387,36507,44390,3772069,3772329,40944,38411,42185,33734,35715,39880,31950,4192
0,5740408,42178,40714,32317,35222,32988,34169,3885631,318133,40265,48775,3772015,3772216,32279,43199,39204,39919,43489,34282,41189,40163,38733,41659,34453,35023,37044,37652,3771829,39332,33686,40359,43231,36037,35402,47767,37627
,41503,35452,33822,35684,40718,40411,318947,34624,38936,33650,43382,35711,38503,36682,47594,43230,5740414,318595,34250,34357,31361,33564,37761,31639,43913,43713,42726,42918,35920,318249,33118,44110,37587,4379919,36919,43357,4119
5,42843,32597,39363,42192,32294,41425,35386,36529,33717,31158,40945,35419,34966,33529,33757,31344,40097,36080,42026,43563,42633,35259,59173,37836,42919,31835,326139,32982,43757,38601,40159,45320,36475,34449,40358,32202,42366,3998
5,43827,37536,41203,38303,43616,38344,35127,3355160,32973,53502,33473,46456,42993,43169,32916,5740235,33103,32587,42508,43466,38526,43533,41401,41784,48903,33469,42894,41850,43417,42637,39415,37535,33194,2768853,35149,43262,4833
6,43292,43160,39067,31815,37703,38602,39852,326148,44529,34264,38605,41542,39017,32662,38715,37034,43547,31217,39654,43814,36209,43018,43100,39479,37185,40719,43448,42393,34718,31222,41083,32529,39027,40638,35315,33226,42817,3319
6,33114,32686,34315,35179,33033,37978,38964,32885,39549,34930,31731,34374,31899,33255,34132,33007,39671,41362,32102,35537,37988,41133,35934,38224,42254,33000,43423,37591,38695,3772300,41217,35091,42752,37871,31738,5740323,34373,3
7444,43105,38353,43438,41868,33532,37209,32446,39234,34736,35370,35761,31586,40176,38504,40893,38369,37749,31601,33524,49713,41446,317898,35737,41176,43529,42728,32791,43502,35969,34985,41538,39588,33135,40260,33932,36799,33992,3
1145,43669,33655,45460,39135,36128,42389,40925,53428,31745,33356,42230,33948,31143,31244,32290,43916,37431,40177,35390,41804,38161,34286,31348,35559,31807,36717,35712,44279,41204,34393,39987,39137,36513,34803,3885647,38352,43495,
33526,37296,38928,39768,39547,33341,41951,35953,36563,36914,33241,42291,33563,35442,45840,5740590,31035,38286,31605,53569,32679,42937,32312,40036,33119,31089,37068,42195,43652,32242,38433,37449,37410,31732,33282,41126,35159,4231
3,39679,33076,40139,39944,31171,37277,32341,43473,34426,35865,31841,32107,47718,40384,42058,38455,33346,33434,46662,32969,44030,37375,36772,42973,35511,35355,40149,36881,40863,42805,31388,37164,32334,31221,42221,42543,34982,41263
,36963,40975,35738,31717,38430,39860,2768674,33680,38756,35688,33132,32572,32989,43508,38019,50058,42520,31697,44046,33951,42302,42414,43555,32506,31801,33541,38345,31083,33714,42357,31612,31119,32674,37155,37730,31826,36048,3701
4,36146,33521,39284,35892,32358,318636,43093,36269,38451,32008,43724,36447,38760,32113,31589,38148,43735,38532,43008,41550,53442,36984,42900,32212,39552,36038,326165,37533,32625,42325,32230,43982,41313,42996,38807,38178,35286,415
33,32520,38816,44339,32888,41104,31525,246496,38103,43345,38229,38377,45785,43004,35198,42462,33498,42934,37981,36594,42850,31500,32300,32295,33648,31110,42279,39557,246410,33128,32183,42590,34568,38116,37196,32013,47219,36560,39
717,35785,36499,246459,32063,37973,34969,43690,35297,3885643,46201,33516,31866,37463,38155,32437,40557,246421,31524,34445,36606,39847,34095,41190,40912,35257,35083,32568,35364,43228,45525,31016,246618,35944,40998,32047,42754,368
30,32222,246629,33210,33191,39133,50253,37896,39917,3355164,4379834,31735,34380,35098,42518,117414,38730,37040,3355136,31157,43179,2768843,34450,42897,38787,36150,31318,41819,36079,2768879,42561,34385,34622,32480,40938,43552,504
03,35375,40073,35671,40134,43901,38389,32976,31881,42380,36285,32335,32050,43236,42834,41161,39410,41709,32940,34747,36696,43792,38677,40181,40111,41927,37385,41266,46008,41911,40300,40665,34784,31739,35192,39554,37935,47757,3679
4,36810,35540,41636,44233,32523,33643,35347,31851,31681,33298,33914,33396,37047,35609,38801,36273,44127,32593,43399,43391,38534,38514,32790,45883,39450,38478,36357,43783,35744,35697,36825,35490,36652,42030,42031,39314,43492,35121
,38969,42243,31596,246445,37323,49805,37637,2768916,43580,35891,33973,40687,3771973,39625,39894,32259,33304,35248,40959,38779,37462,43042,31401,32688,43767,43532,33369,32350,41060,33236,34097,39551,32768,39212,43170,42449,43828,3
7916,42936,35673,33627,34311,42999,318210,39108,43892,39521,39693,46194,35560,34715,43830,31142,41304,38808,38185,32080,31945,35488,38890,41597,33254,42515,40643,35985,31869,41931,35957,43720,36681,3355135,35483,39260,36583,3932
5,31518,37476,34787,40336,37152,37817,33704,43794,35343,37205,36400,39119,34274,49953,40994,33017,38076,42652,36497,34889,41258,36281,34065,40169,39076,46719,43128,31399,32337,43290,31513,31335,41343,39157,39979,59170,36685,35116
,31418,32014,32142,43349,40305,33677,40700,32722,35085,38656,35799,42376,32217,32764,35522,43952,34519,34397,38313,38208,42586,37996,41528,38643,31293,43293,326216,34170,36423,318212,33421,44254,34154,35971,43719,42592,36252,4111
3,36683,38374,42440,4379867,32637,37919,39734,40231,33466,31012,39228,39948,318872,32494,46140,47272,37862,34804,34432,33373,32954,42055,36159,33233,41938,39611,43095,32407,43691,47091,36985,37325,3346235,44861,35420,41459,48805,
47186,53434,44125,38492,35664,43238,32226,42866,42101,40131,34754,246580,34159,32554,41578,31921,43811,53586,39337,32607,37931,35000,32023,34714,35879,31667,53472,33823,34193,35228,37288,40368,36290,40831,41120,38846,39043,42394,
41522,326255,34885,36967,39691,31386,44543,36104,37994,42128,43656,39610,39524,40861,41778,41053,50225,42386,35232,34480,318991,36720,38736,38734,32909,36237,38232,33388,35109,33807,38785,32661,33899,31342,34805,42201,31208,4062
4,43809,41228,46260,49505,35324,44433,40357,37786,37804,42852,33963,38469,34997,31593,33427,40410,42943,41179,37393,38542,49968,33045,33900,33212,31977,33999,38763,37950,36205,34001,42747,40507,33110,42059,32962,32612,32039,38721
,38995,41878,34636,38901,40294,41855,34111,40851,36951,44965,37301,32608,34433,37808,41434,34398,36955,326186,43325,40974,34228,38343,34921,39878,31665,43197,39259,34847,32789,36860,35405,41672,42960,38055,48481,32924,38883,36746
,42504,31887,41247,33139,40459,40453,38397,34004,38413,42358,41788,41071,34618,34242,41656,31098,318843,40967,5740757,43493,34096,50246,41182,42086,47080,35928,318221,39843,37124,33353,41935,47121,42466,36485,34616,33263,36956,35
954,41026,41114,31331,35695,42267,41184,35395,35974,35988,35100,39217,32670,35147,50356,41437,39628,40934,32737,31247,41436,41014,37482,33424,33379,39630,35113,40000,39118,41045,31604,43751,35197,31914,33972,42626,42428,36552,352
18,32841,261634,37702,261631,43003,34996,38421,2768981,44785,39722,39375,46158,35194,36719,33562,34939,33018,35682,40126,251690,34324,36108,36986,32007,326234,33504,38424,36138,36376,39735,34442,38885,37859,36398,36856,38934,384
23,39439,37875,42464,39302,44793,40715,36589,44724,34826,33567,41744,39254,42836,246653,42141,34155,35128,39746,43235,31582,42169,42630,246652,53437,38262,31009,326193,38628,40553,40081,33214,46078,37860,37921,39267,32158,34172,3
6619,34481,38494,36022,41495,32508,40626,43905,33965,38193,32777,44702,40894,33083,318043,41840,34699,37201,43618,32598,36689,46080,37647,317815,45928,36019,318559,43359,33259,40712,43958,3885654,37033,31106,46038,41067,43077,34
608,39413,31842,39478,31387,42137,36731,38070,37084,37339,317952,40431,5740708,3772055,43844,35189,35949,31795,41073,33982,38647,42500,39153,39164,31377,38485,37741,318016,42816,42571,33503,40958,5740853,44128,33597,42290,43329,
33111,32338,32145,36911,7354471,41616,36598,33281,32546,43842,37704,43950,37936,43182,41801,42124,34750,34514,36851,43057,38714,38970,42439,50227,39650,37478,33499,42748,34527,38978,36073,45268,35144,338395,37329,35565,37728,440
59,2768909,32953,34009,31240,32576,40045,36948,40852,37570,35337,44250,33602,36061,48535,39826,50296,43222,31239,38136,42191,38446,44451,35757,40791,248102,37892,35140,38195,34831,32430,43573,40792,36321,42062,41827,32282,38259,3
6492,40190,31121,42764,36068,37628,43348,41785,36295,33857,34924,35298,40879,31597,35053,31708,32514,33237,36415,42689,32938,33983,36102,43103,117419,44260,32640,31805,39645,34548,36574,38915,39759,36144,40664,38646,31631,35267,5
0139,34626,2768951,36025,37803,33367,35756,35426,37467,31090,44620,59218,33198,33283,317935,37216,32518,41193,37401,32406,53444,43517,32770,41743,33227,35155,31588,40399,42600,33689,2768716,32196,32038,42820,35931,31811,32408,31
8079,34677,34962,42572,33849,2768892,43550,42842,33182,33134,34504,40583,34433,32288,33653,38956,38081,31420,46091,43978,42266,38945,35200,40256,37589,32729,32701,31044,36764,34740,42452,42789,50250,38732,32902,36040,40490,37427,
35262,43528,38059,34993,3355120,44672,42662,40800,48785,38063,41377,32703,43774,2768670,42265,39243,37809,37794,43240,40566,32515,34610,38637,44271,35900,41125,35932,40369,36053,41805,39025,32624,318578,33136,33690,35680,39399,2
61620,32537,318600,39213,35381,3885607,326173,35590,33034,31176,33260,33005,41447,32045,32033,39858,35627,38110,37019,36747,43400,50323,44653,40129,35848,36173,39122,44353,40565,38017,33049,32622,36498,35293,40582,42721,43355,39
430,39935,34408,45329,43557,32755,34073,32140,41185,34120,34722,45827,40451,41168,38355,42027,38516,35930,41873,39014,36028,4379907,4379908,41042,246638,39434,3772124,31147,35539,37927,42813,37601,41641,3771751,53556,38982,33739
,40343,41056,46074,49809,38873,40663,42174,40096,7354371,39158,34708,38047,41404,33198,36961,40605,37035,38360,33172,39657,35993,35966,31363,31818,42871,40559,41847,31140,34669,35555,32055,35747,35044,37021,41100,40936,37938,3863
5,42985,34683,35963,33167,43572,42758,42342,40132,37894,38530,3772349,3772641,33343,44100,39694,3885646,40786,31658,33056,42990,37330,36064,37781,37824,43017,38819,44885,37249,40324,33137,36436,33220,36603,41395,35895,41154,3675
9,34287,43019,44573,41838,32660,34594,40960,35288,37925,36168,42498,33883,38888,2768677,43242,36670,40548,40048,3355114,37203,38206,38013,49228,41608,41526,38987,33738,50251,34137,49701,42587,41689,7354380,31153,39150,37414,3592
4,40191,38528,36362,32613,32164,318990,41741,326198,36386,43558,40589,39535,41770,42802,33229,33305,42046,326213,40303,44531,40138,318841,32258,32563,33276,42703,36311,37400,4379861,38100,34135,42593,41443,35917,33853,40273,4234
3,31628,39969,42136,44811,41438,42770,4379817,45318,53473,30975,42215,43428,38378,32373,32149,38109,33235,39875,31724,43594,39536,32898,39724,40437,2768661,38865,35849,37979,33105,32241,40062,39206,43561,32569,38505,41940,31874
7,42882,39255,32116,42828,40240,36210,34488,43384,37397,33043,326119,38669,33654,47082,39251,42735,37078,34284,46234,192507,40970,42283,40174,39104,31373,40360,37261,41842,35929,31346,39180,45368,41340,31907,53562,39513,36111,339
87,44791,31598,38367,42175,31225,31097,33184,40754,34404,31657,50133,35123,37372,3355130,32471,42402,41137,38698,41839,33651,318082,38640,43404,42903,42516,31537,38742,42757,48552,3885575,318231,35564,32169,32605,39099,318077,40
172,37858,43852,45339,42258,41109,40654,40187,42020,44160,43159,38961,44660,34892,44183,36820,32162,32269,34354,35172,40717,41783,38700,38376,34688,41960,44497,38697,42595,38255,31392,34950,40540,39820,3772183,3771756,326245,383
95,38490,38864,31430,31562,43178,34785,31228,33265,40034,43754,33809,36788,32104,39750,37430,5740674,38561,42051,318095,32551,35662,39903,37914,31086,41150,46243,41776,50363,34648,37832,34503,40781,37992,59212,38336,36338,43481,4
0383,44360,39751,34709,35084,2768997,31011,318161,39749,33732,39480,39856,318599,41857,35899,39686,35938,34738,41205,326135,39685,31764,50405,33805,42000,39689,3772476,38487,36995,42840,59246,34409,43283,38488,326232,33938,73544
66,37633,37546,41942,34387,35795,40438,41603,38057,38373,43277,3771951,3772262,39069,41074,3355118,47906,326158,36773,35097,40162,33027,36514,32340,38243,42209,36766,44544,42033,42397,7354473,59260,37071,38033,38300,40049,37934,
32410,31104,326161,43148,41215,37643,31608,39833,38796,32240,38031,31904,40790,32278,37451,33836,36014,41029,40622,38062,40996,41551,37820,64874,31166,33240,39758,44029,35336,318259,38020,31310,36464,33825,34070,43768,32934,4264
1,32671,40932,31521,39914,41427,38648,36239,35397,38001,31523,38769,32886,41983,326192,40178,44554,38593,5740156,41896,44870,41525,37618,326127,37659,3355013,35754,32918,32604,5740700,39086,33943,43164,32596,32866,31230,35702,39
240,38794,42272,34437,40876,35923,42806,42881,39972,32462,38363,36118,40843,39095,47396,5740362,40597,34158,41876,40332,43328,31804,37699,31499,32819,43984,35952,37632,3772719,3771946,32522,43031,43730,37415,38418,43346,38187,35
041,43772,34974,42509,35730,36282,32933,31874,37335,36125,32349,50428,34675,41013,41899,43694,31812,35378,36133,38046,37384,40472,35850,36797,32780,53573,31181,44001,39659,35975,37602,33418,41558,2768660,33204,41022,35331,36585,4
2125,41837,34495,38221,39934,44522,41598,42179,31567,32208,31970,37178,39684,45031,38913,39997,40590,36566,33840,37912,36160,33409,35219,36123,35030,34421,31713,41835,31611,36171,40015,3346237,41871,36420,44701,31174,40074,40361,
42545,38068,47781,43515,39998,326214,35430,318680,35215,36374,35072,38323,31022,34183,32730,40189,39809,38342,34496,43437,40610,38499,41849,35842,45969,37561,33502,39141,42436,31687,33458,7354476,50188,31253,45784,41162,36540,38
375,3355134,40281,40495,35066,36027,34378,40946,41068,36945,36570,36546,37475,40506,41740,36912,31245,39721,48448,34881,37381,44228,32328,43398,32400,31535,31870,35154,40558,31319,31856,32815,36981,31660,34126,35732,37895,42858,
40377,37072,36001,246497,31127,32204,40044,35591,40014,40686,48782,5740551,34320,36271,37966,33761,34362,32330,31163,36372,43946,38611,32771,44786,41423,252479,41398,44275,31771,31577,39883,41844,31571,31260,39147,38692,41341,43
79886,40064,34707,31741,42239,117332,33195,31376,31292,38194,43491,43559,32937,326143,33693,45343,38400,44263,32882,31840,32872,39404,37178,38667,317946,45739,40577,41433,33125,38718,36639,37020,36821,31259,37581,32228,40053,324
78,40787,43812,42005,37344,33219,38219,34681,42143,35524,37768,33835,38905,37723,31150,318706,45906,43576,39321,31543,40326,32552,319048,42490,31684,35677,32457,38694,38786,31042,2768667,41390,33158,40308,42129,31905,31775,36760,
35955,35501,42891,33782,36727,31165,38749,32501,40378,31988,41260,32505,33611,37154,33838,32379,33646,33967,4379854,42927,35882,39250,31071,318857,32315,36240,42876,37873,32683,36360,39522,30996,41147,38493,45338,33989,33348,316
09,32970,3772560,3771728,38151,43130,47869,40683,33140,35478,41374,38540,34231,38655,37754,35102,31026,36908,41993,33649,39770,44155,41456,43835,39268,31647,43546,32854,43660,34321,42646,41675,45398,39484,37946,38258,35837,42839,
41707,59215,32977,41269,39766,48632,41178,38963,38820,35365,246461,42642,33979,38228,40386,39083,35532,37554,42183,42615,39387,47285,319025,34975,41865,33775,33607,39829,37729,37076,37585,36299,40904,33716,33256,46020,40699,4068
2,38006,33301,37063,37767,50008,39111,32594,40508,31948,33179,35507,35787,32027,3885573,37795,40789,42857,34167,44011,42525,39966,36697,31184,46415,40950,36224,317878,31309,3355079,42647,36997,43300,41363,36809,37455,34771,39013,
36655,39769,37967,45683,34196,42663,318911,36921,35362,37664,35762,39438,36926,39936,31817,42285,117353,43637,43927,2768875,42899,39613,35411,44843,41406,40515,39448,41091,32573,32935,31942,37450,39624,3346238,192509,31156,37011
,42749,33303,326128,35784,33939,42064,43856,38260,37511,46040,38079,36054,38979,39400,41638,38649,38086,32124,43673,39844,50392,31483,31160,31618,37785,35713,33402,40844,40195,32776,31798,32109,47894,43215,38529,42370,41118,43241
,34352,31381,50005,36750,41458,318743,42146,47998,42632,31678,36363,39080,40668,32738,42181,34396,39026,31107,326259,34783,31979,34906,38844,31574,36857,240672,34371,36617,42549,33988,37537,41690,35002,42880,33684,42888,32890,318
97,32541,41342,32579,36793,326206,43028,31792,40981,31976,31557,36736,42089,37849,44484,38943,40302,38023,35236,35898,64880,38248,46006,40388,39720,32273,33645,3772312,40189,40168,43517,31351,38227,45668,35527,36722,33921,32987,3607
2,37751,33834,44096,33109,36137,39818,40549,45360,31953,45307,31991,34516,41262,41138,31213,41646,32979,34646,34422,39688,38256,36337,41090,38159,31148,40084,43775,33715,36763,35588,39899,43862,31052,31659,35674,42299,32046,31177
,35453,39270,34428,250735,46027,43676,43153,42624,31175,39561,32585,42371,39874,40723,246457,7354379,33905,41591,38037,38654,3772082,36578,251900,37757,38093,34329,44703,50060,42691,34674,37662,42110,41085,36732,35778,41180,3888
0,31449,40617,36579,43693,318206,33560,38831,37833,41592,44889,326121,34021,39394,40694,31364,34300,35529,50335,41080,43459,36527,38324,47769,36147,36411,35268,35379,31940,2768940,39082,317928,31224,39619,43344,37168,40659,41399,
36442,37932,32631,326205,39772,35311,36309,31987,32635,32946,33215,31844,40933,38959,38176,38051,36749,39263,43163,318824,40711,36698,38438,36676,33397,41268,39683,43469,44355,42553,33604,40436,42309,33964,42617,33170,44014,3487
4,43788,38349,38398,38806,43518,42413,38613,34119,246507,34392,32971,34107,43488,32782,35672,40087,39765,33552,39593,34062,41141,31625,43664,32472,34557,326228,44131,38599,35961,41962,43718,43766,39862,41319,40547,35045,38067,360
56,32544,37500,36748,43923,37117,41461,46058,7354422,42501,5740442,35660,3771985,43943,36408,32448,41206,37018,32595,34550,4379891,45783,36568,41674,31706,32700,42445,40262,42742,42665,41749,36020,32368,36448,34549,4379860,40242
,42846,31747,38177,40487,36397,37989,36432,41165,32458,38326,36706,34263,36255,31553,3772280,3772012,42944,36680,35995,41862,34734,36632,43271,31215,37987,38788,37479,53578,34312,49636,40596,53439,39437,35071,36082,44259,32928,40
670,42282,32910,41628,37733,43750,33307,41541,37811,31475,45399,34405,40433,41569,4379911,32292,37640,35142,42935,39958,37805,37706,42364,40642,37090,34524,37116,41365,43556,38420,41271,31496,40030,34112,40661,36304,47457,36544,4
0092,37334,35678,2768996,33131,43167,37070,36303,34541,38174,32539,41043,317813,43315,42093,49260,36059,41084,43609,31043,38096,32721,40709,31101,38930,36831,53445,41880,42475,32395,37661,36289,36495,41848,32482,36669,37923,3468
4,33293,31833,40929,40105,38523,33289,36541,43713,35240,39483,33777,32716,32777,36203,40059,43507,41570,42874,40641,32820,32277,35425,41158,32150,35391,33019,37062,44437,31393,37144,40653,33991,35050
,31095,31772,32456,42986,31638,326226,32763,33928,31082,41122,33338,43180,41883,33015,31359,38477,43001,37069,40739,36031,36426,42496,37378,33268,41992,34992,43727,40660,36208,32878,326122,40716,39366,40403,41347,33166,31989,3778
0,31544,38744,246605,34423,43471,36494,33435,32615,38226,45064,32005,32245,40391,41836,45976,37029,36826,31448,37842,37238,33225,34222,37379,37073,32163,33636,35486,35499,38149,3346167,31350,32984,39397,32638,41957,38332,7354429,
31700,42220,47763,33094,40165,33629,44173,41058,35316,38002,37734,41939,32101,37679,37586,35176,37328,32589,41282,41431,32114,40633,43833,37310,39265,41370,45871,43838,39940,36393,44643,2768680,36239,40693,36975,32115,3189
18,38981,34245,246682,32691,41279,34294,39902,32955,45931,41078,34283,34775,38338,35342,40164,40127,35070,33245,44540,34372,36042,31037,38429,32133,33977,35925,32073,32094,34595,40581,42922,42672,43087,34542,37886,34710,38983,402
32,41718,40859,33339,319043,32119,40186,44326,41181,32851,32752,34790,32057,40155,39961,40983,38150,38283,36927,39986,32684,42065,32992,31542,39608,37924,41201,31813,40270,37200,43981,43725,36918,32352,36050,42173,31997,38598,317
67,31615,32836,44097,38447,34573,32475,32136,34256,34546,43444,33910,36545,43233,39663,32966,38589,34781,35146,31698,53580,33736,39161,39943,36784,41917,35959,39377,34877,32100,36846,31834,35398,43041,37065,31096,34420,42360,3492
7,31776,34500,36199,40401,41317,34767,38022,43611,38321,41845,39882,33156,34748,47216,44307,43810,40449,32111,59172,39835,43795,32936,33465,45970,40385,31405,41532,34156,36761,3355124,31204,33850,39253,42467,35890,38173,32268,370
07,46326,36044,42273,326197,42460,33518,317859,34079,37290,33911,33760,41745,38513,32090,40842,41921,43472,40545,36542,37769,45247,30973,43374,42150,32547,41226,40060,38199,45655,39612,35788,40907,35485,39745,43267,3355163,42526,
49427,37437,37471,41491,44548,33389,34860,36143,41087,40264,31792,34241,44129,36306,250811,35569,39647,38674,39386,34700,37746,31751,40135,43803,38489,42196,33082,38044,34327,53577,31845,32599,34713,45682,35536,33753,37466,33201,
38091,41197,31960,42470,36959,318645,35927,31141,5740565,35027,37800,41028,42208,42550,32016,36302,36876,32566,31195,37456,45380,32351,42653,32439,39218,42693,31001,39381,31473,36287,44266,35348,36280,39548,33350,32434,32440,329
83,42186,37079,37433,43617,43354,39873,37377,41081,43765,36384,42498,33077,48311,36838,42567,35454,43358,42844,43739,47260,41747,43565,32803,37782,36915,38065,40785,38884,34024,38527,46187,59151,3355143,32981,36134,43101,37006,31
211,35919,33059,261607,44505,43192,40505,40857,39877,42991,37043,34197,36358,39587,33744,35057,36562,32548,31906,33051,32732,42931,37042,40710,41766,36555,35509,47218,39210,31955,42139,2769001,36035,40239,35444,37464,35563,43808,
48071,34403,32974,34592,41460,41294,43023,36645,38074,44272,35789,35339,37172,31144,45574,38007,41320,41673,44819,33496,32275,39408,41391,40585,38135,35653,44172,31459,36974,40576,31474,31879,39175,35748,35440,32786,43319,36576,
40234,40171,38997,37864,43758,33470,39570,36640,40541,41167,33471,35570,3355158,38603,40602,36103,39329,35500,34338,40966,3771905,3772679,33862,33314,32502,47762,37846,39596,42510,41498,33712,40158,45250,35594,45269,36580,33638,
34069,40692,40291,31679,35526,3355089,32243,33916,40464,35991,43401,36381,44728,42531,39301,37116,38595,42803,33774,36699,45894,34539,41617,32646,33372,44438,35001,45401,39455,38800,251645,38184,40457,32071,32584,39446,39331,354
00,32205,37367,37635,32510,34778,38175,34240,40517,35956,31908,42493,39764,40544,37228,32321,41139,38740,37402,32652,35052,32463,43700,33002,43717,31214,39821,3772396,39440,34794,40390,42521,317913,44978,34903,42087,31425,34140,
43165,43166,32262,31130,35847,40249,31793,5740176,42406,36129,32112,35741,44238,35292,3771984,3772658,3771897,41244,32440,37221,43456,37130,35652,34360,35995,39757,4379844,31809,3355125,40224,37386,32256,36229,37237,43276,35921,
31265,41256,38073,39319,34479,40980,34441,39202,32941,3354925,32919,45786,43125,32473,32157,38144,35157,43734,33808,31816,43823,40795,44149,326257,32860,41289,2768961,43096,45374,32754,44801,41930,42812,33264,43310,326177,41860,
39842,38931,31362,318563,34364,5740472,318867,261629,39737,41095,38157,45680,3355129,42314,7354432,39990,33026,41864,33390,36612,37646,32564,42165,37532,41859,3355138,39491,47765,37481,40593,42412,35881,43217,44747,34359,40556,3
772133,34185,36154,34302,38590,34416,38696,42015,32715,32274,41981,33509,33896,34571,35583,39445,38060,45316,32601,33269,32611,33845,31746,35586,32001,38186,53567,31493,37417,30985,32425,36767,38113,34215,41870,40652,41657,37953,
34925,39978,318958,36730,36131,37387,39560,2768685,31781,53438,32887,41746,53348,37260,38504,35547,38711,41578,43773,48380,37784,38893,43764,36988,40272,34175,37521,41562,33966,41054,37
005,32106,37465,37727,41143,34979,34276,31079,41173,32209,41907,43906,32225,40146,38968,31030,34559,42499,40144,37872,41177,36110,45467,42177,39340,33640,41235,42679,40398,39669,31017,36978,41145,35624,41995,246397,32968,33025,36
297,43710,36840,42544,32678,41802,40295,42987,37588,3355126,33462,32239,41296,35576,326166,31456,38291,42368,39655,40947,31394,40196,39558,42707,32656,32123,47877,32908,3772640,3772344,33270,36455,37140,39443,31664,33918,32000,3
8249,37272,43005,39417,42845,38257,37389,7354437,34134,43434,36564,34032,35254,32428,42218,33024,35258,33851,31374,32097,40955,43588,34751,31632,31187,37165,43312,37235,31992,33881,39537,33608,37411,37202,37837,37123,37995,42972,
39454,43569,7354420,2768948,38412,31926,40415,31347,39435,37336,40973,32869,31442,41441,36587,34080,39732,31666,37177,33971,44679,318909,246627,43442,35649,41800,32796,33981,53435,31802,47399,3771726,3772080,3772213,34358,57402
50,36310,37066,36288,36329,37840,37970,37038,43924,31329,41779,38765,31838,33512,5740557,7354430,44000,37080,42076,42863,40414,34179,41965,40217,32513,34192,41757,36674,42994,34967,31487,31006,31957,38812,43351,37199,40461,36278,
35195,40987,43385,31129,43787,40530,37230,37993,43158,37443,31762,34281,35922,38327,34896,318207,44851,37841,33710,3772221,3772232,34655,33778,42811,34540,31257,38230,41540,31663,246596,32556,40542,33904,37790,35779,40063,38372,
39594,33906,35512,43476,38078,41350,261329,35306,43944,39311,40850,36791,43826,50050,43284,40835,32467,43736,36058,37801,36170,42484,32474,39955,42862,38160,2768852,42285,41714,37638,39198,43218,42435,41798,33247,429
50,41353,32429,37583,35265,31353,36726,39725,31760,37434,192508,39338,49424,49297,34425,38427,43157,35278,35595,40292,7354428,36571,47283,49638,31555,41072,40448,38254,43836,36233,37292,41852,43377,43633,33962,39651,33441,53560,4
1107,34757,40848,34142,33014,2768848,35790,32725,43415,35325,41082,3355010,36238,40673,5740668,35387,38218,41102,326251,33554,42778,43044,32846,37360,36678,32616,35135,35031,43873,32663,40434,246538,33854,40421,117365,38661,3157
8,34363,42824,40529,38028,38512,43928,36913,33974,33423,37552,40456,31170,43864,38971,5740513,246485,31265,39716,42363,32741,31798,37787,45325,31031,34394,41006,35759,44391,43291,33003,36521,37893,53565,31803,37171,37755,36621,3
5690,42795,36431,32318,32207,32167,32143,45961,42664,44324,35130,31846,44027,46009,32557,41599,50417,37718,39962,40940,43735,45301,36130,37215,39834,39910,38468,40331,39031,5740131,38932,34829,36506,34735,41910,39429,41112,369
93,43586,38757,40229,34796,39849,42447,39136,42971,39846,41059,37254,34642,39274,34987,35263,3346209,39744,39102,35610,40962,39145,246512,34970,31243,34139,37731,48387,48228,31379,43332,318828,40351,43330,318568,31290,35385,3274
5,38146,326172,35693,36419,39670,34780,39864,36774,42437,40301,42696,43654,192506,37562,35359,33202,31646,32263,38301,31154,39845,37247,39160,37850,42047,34842,37851,38216,33628,37580,45233,42048,40179,34099,45775,40696,35054,437
23,38984,37142,42596,32797,39402,36259,32083,31958,3772305,3772101,50472,37977,39431,35685,43799,39956,43039,42879,36989,34292,33306,33619,39901,251610,43737,37826,32623,42793,41524,33300,42753,35173,34487,34361,36158,35893,353
26,35323,33528,34148,34000,36896,35523,33477,33148,43912,34149,32734,44052,42671,32455,41151,32213,32582,33001,38652,59216,35295,53581,36614,32664,39405,40327,36365,31050,40290,42256,42591,43000,36769,41479,37792,33107,43741,3982
7,5740203,44132,33414,38168,41891,41025,41952,38821,39433,33081,43791,35933,32573,33681,31554,46155,35574,35696,32948,36554,41470,37081,37143,31669,43289,37523,42899,33859,41079,39035,31357,34413,32939,43441,32877,31229,49816,400
90,44205,42984,32666,35654,38580,35026,33903,42145,35305,32907,32078,33098,42736,40514,36463,3355144,35801,42867,31072,33819,38304,32470,41346,34614,37298,39523,38077,37239,36460,40094,31201,38459,37636,38422,39644,39855,35213,47
900,36015,3771934,3772186,38660,42345,41968,50082,35151,37778,39142,33772,33218,31422,31423,43402,42199,32855,38391,318890,31349,3772539,35184,40371,46156,42240,318211,40382,40093,36539,33203,42322,34876,34350,43079,40675,36373,
3885567,45959,39385,326184,326155,32602,31624,35203,35743,50459,39493,42963,33443,31254,42761,34928,3355131,39173,43132,39569,36005,2768883,33492,40250,33493,40546,42237,5740435,41397,39616,31261,41589,36263,43531,31151,43331,34
955,39540,38859,42029,33468,38099,37810,39168,40813,7354465,40201,41867,43352,36964,43484,40047,39226,34153,32740,44915,38223,33824,43513,40424,31579,36569,32316,40698,39697,40133,318059,34325,31527,33898,318164,35101,37289,3122
0,34293,2768886,36361,41416,38904,33837,38793,40225,34696,40708,37668,39159,34160,32757,38415,41200,40846,36795,35939,33394,32153,32889,36653,45382,34956,41208,48224,44115,36277,34980,41566,42034,32787,38341,41310,117331,44496,34
157,53443,33658,39583,42680,42171,36854,42379,42638,33185,44087,36176,38385,37948,40395,35731,37922,42734,41020,34632,37798,42896,30971,43333,36142,34844,39018,38568,42875,48311,33769,43266,42411,42853,326237,7354424,36673,37322,
43541,31849,317860,326157,39904,31000,39103,34665,42446,39032,32141,246578,37156,34388,38898,36609,40032,32993,34719,39350,40439,33162,41372,37176,43938,45280,42995,32015,42253,5740528,34765,32690,246513,43554,326142,32195,38444
,37838,31332,43709,42524,39763,41432,32257,40188,40628,40782,41925,37517,42861,38630,40924,36200,35578,34742,39866,34333,35979,32378,31773,40647,39459,33944,42682,34569,37959,33165,31872,40569,31759,39266,45757,41756,35596,39687,
34002,38748,32233,38752,38881,37774,59253,42982,43816,3355127,36808,41773,48450,32493,33555,42822,34313,36465,45881,42825,36087,31620,38966,39090,31902,39309,39747,36286,41375,33258,44446,41166,33505,31600,36491,32524,41585,4027
8,36242,38937,36406,36421,33687,43016,41913,32459,34098,36561,33035,47730,5740526,46066,5740845,34431,37603,37480,43698,36262,32769,42327,39474,36855,42434,42444,46068,34058,32852,33495,319033,326252,43278,41373,37356,35329,4425
1,33460,37852,35733,41230,43841,38811,39889,35028,37395,45278,36992,39214,39505,33795,36953,35749,43386,40623,35170,32947,42848,39453,32530,32069,40972,35758,35418,35475,117368,41627,42945,32221,39174,41123,40055,35796,36565,4407
1,42115,36445,38287,34526,41286,326210,35421,31192,33232,31630,32049,35843,42517,40173,42898,40657,39376,38214,42874,38645,32627,44150,44900,31014,31617,31003,35679,40091,35279,252554,32019,38153,3772179,3772427,39143,34910,3430
9,40814,41843,32588,38541,33513,33048,47141,42103,35223,34551,38142,42130,37712,41295,42142,38565,32492,317823,42140,33500,36339,34564,33062,40630,32746,35314,31680,38246,38726,38909,7354472,39475,37843,35667,246656,50002,42952,4
2392,37857,43162,42979,36241,40870,40528,36440,33180,42804,42453,34402,33682,38231,38027,35771,326168,41602,31062,34984,43439,42849,33295,42601,32070,41089,42819,33488,32920,40458,33758,39244,3772655,3772367,43229,41997,32838,35
584,33676,42182,43824,5740252,44086,35504,36771,36675,40462,31800,43012,35056,41830,38755,44111,40680,36319,43771,39460,36703,31649,34954,37876,34367,40616,33171,39988,36264,39980,43974,36405,39456,36033,33370,31004,36824,35051,3
9477,37542,43350,32017,38886,36461,44226,34427,38396,7354398,34619,40847,38154,47251,42906,5740389,35129,251466,37631,37869,41943,32793,39424,32616,36049,39151,43387,35977,31015,37148,7354425,40993,31470,31517,39436,39313,41833,
35816,40560,35225,34063,35577,31607,35742,45821,43749,33930,37337,32132,43327,33770,33897,37214,34314,37403,38026,38207,32687,32125,31722,39367,35765,34370,31248,44261,37549,34341,35552,2768944,41152,34177,41349,44736,31911,3639
0,39653,5740396,3772671,37559,42808,33852,35246,33262,31146,31466,40150,32567,44359,49045,41038,53557,40676,33288,36462,38519,38824,32871,34721,47746,38916,32540,31137,43822,42478,36122,37823,42656,38521,44668,38644,36999,43566,3
1105,32490,41360,44317,31179,7354434,39365,38198,31799,318785,41055,39195,33489,261613,41719,31256,43692,45826,33725,41679,40563,31533,42904,35335,53561,34377,33176,41872,32561,35241,40140,35780,34326,39258,31118,41738,53566,374
18,32533,37573,40269,2768929,38452,40170,64875,40141,42204,37579,42854,5740593,42286,41227,42841,38491,39811,117294,32371,32511,31458,38244,40639,39425,41959,40396,40248,192535,32565,31890,33444,31027,38365,35783,43549,43650,500

70,42738,3346177,39241,41666,40157,39428,37082,40412,43294,3346206,42369,36425,32042,34582,36966,37617,41670,39501,34280,40046,32751,32464,32743,37515,44021,36377,39091,44207,44118,43462,39922,38863,35998,43449,34411,39222,42759,
39485,34968,37639,44039,31707,41367,34745,38347,35445,34086,37382,39368,41587,34957,40656,36043,34835,38795,42025,43661,32056,35327,48309,53563,32818,34976,40043,33968,39502,43829,41531,40672,49168,39753,39555,42082,37557,33685,3
9403,40447,42249,41273,38815,34136,37961,43288,40707,39107,40949,44235,41861,32021,33864,39881,40527,36444,43728,41187,47249,37121,33863,34554,36775,31354,43798,39776,37179,34613,38045,326215,35089,42431,36409,41834,34886,38204,3
9089,36584,49804,36718,42826,44839,34384,32930,326150,38762,37233,40952,38891,42743,43560,33108,32765,33986,43083,39857,34686,34012,40697,34875,53554,40678,38082,252671,33721,42649,35239,31481,42695,34484,35289,41219,31450,34330,
31404,31662,42438,34883,43839,41611,33922,42618,38205,39121,42809,43568,49779,36270,34102,40299,37775,38912,35168,35864,42946,38799,32875,33843,43770,41270,38090,34066,35237,35614,42933,38288,37461,38717,34658,39322,35648,37962,4
1392,38399,41094,40261,39810,38156,34486,326152,39520,37204,32639,36336,38094,40233,31075,42063,37866,41615,40143,34353,326153,31740,41648,46121,39884,35701,40079,35846,42057,36658,38668,33261,32122,37965,42838,35330,43426,33514,
39970,41543,35997,35287,33178,3355139,32603,33213,32630,36382,43740,32543,35099,43832,36201,40684,37146,3355145,35119,43080,32168,43091,32536,44072,33374,39447,42056,37528,42091,38388,36340,44274,31519,33536,33486,32517,32394,31
355,34788,33392,32853,34166,34793,43216,38897,35897,31566,318983,40554,44642,41140,42957,35169,32617,32024,42306,35112,38137,38817,40815,32922,41316,31629,36387,31393,31149,38914,37358,43574,42872,34187,42942,50391,46015,40432,45
830,41737,41973,31565,35813,44409,41209,33709,43196,35150,40259,36903,2768836,42661,42869,34999,49070,38021,32301,41565,32095,38974,39169,318236,50105,35686,35207,31703,36018,40733,41457,40793,38032,42184,37750,40607,33507,38798,
35235,42928,33211,35492,38145,2768865,3885565,33044,34593,34888,34656,42158,39374,35384,41265,53546,39420,32442,35851,37447,33445,326262,43548,

A: List of all genes whose expression was rescued or partially rescued in TBPH/dTDP43 mutants by GAL4/UAS expression of TBPH under control of the endogenous promoter. “Conserved”: sublist of orthologs of mammalian TDP targets. B: List of differentially expressed genes in TBPH overexpression experiment. C: Pooled list of control genes from A1 and D42-GAL4>UAS-LacZ expressing strains.
