# Supplementary material for: Comparison of Parallel High-Throughput RNA Sequencing Between Knockout of TDP-43 and Its Overexpression Reveals Primarily Nonreciprocal and Nonoverlapping Gene Expression Changes in the Central Nervous System of Drosophila
Source: G3 (Bethesda). 2012 Jul 1;2(7):789–802. doi: 10.1534/g3.112.002998 (PMC3385985; doi:10.1534/g3.112.002998)
Supplement: Supporting Information [file supp_2.7.789_TableS5.pdf]

Table S5 Analysis of splice-junction reads in mutant *vs.* control

gene	exon junction	physical map	A1	G2	rescue	D42> LacZ	D42> TBPH	*p* all junctions	*p* alternate exon	
cac	12/13	11,848,314..50,954				4	2			\
	12/15	11,848,314..56,052				4	**0**	0.022		/
	25/26	11,867,517..8,256	4	**2**	3	2	3	0.579		\
	27/28	11,869,894..70,435	11	**3**	14	10	10	0.076	0.163	/
	31/33	11,871,595..3,657	0	**0**	1	2	3	0.183		\
	32/33	11,873,188..3,657	7	**0**	3	4	2	0.033	NA	/
Pde9	1/2	12685781..874	2	**0**	1	1	2	0.15		\
	1/9	12,685,781..723,742	10	**0**	2	4	6	0.009	NA	/
	2/3	12,686,996..7,609	0	**3**	0	0	0	0.001		\
	2/9	12,686,996..723,742	14	**6**	5	4	6	0.4	0.001	/
Unc-115a	8/9	5,523,483..629	1	1	6	1	1			\
	8/10	5,523,484..4,039	0	**5**	0	0	0	0.002	0	/
smi35a	2/3	14,187,153..8,863	0	**1**	0	0	0		0.026	\
	2/13	14,187,153..233,893	10	**3**	3	5	3	0.053		/
sno	2/3	13,090,543..629	0	**3**	0	0	0		0.001	\
	2/16	13,090,543..105,132	2	0	2	0	1			/
rok	2/3	16,520,280..602	6	3	2	1	4			\
	2/11	..30,460	1	**0**	0	0	0		NS	/
Pkn	2/3		4	**1**	1	3	0			\
	2/13		1	**1**	1	1	4			/
	2/16		21	**3**	4	4	9	0.009		/
	2/21		2	**1**	0	1	1			/
CG6509	2/3	11,509,403..499	0	**1**	0	0	0		0.006	\
	2/12	11,509,403..16,508	4	**0**	2	0	4	0.024		/
NetB	9/10	14,642,954..3,380				6	**22**	0.040		
InR	13/14	17,413,731..5,692	13	**1**	2	9	15	0.008		\
	13/16	17,413,731..28,737	1	0	0	0	0			/
CG34318	4/5					6	**0**	0.003		
CG31379	3/4	16,827,889..28,051	1	**4**	0	0	0	0.017	0.002	\
	3/8	16,827,889..30,523	19	**3**	8	19	19	0.007		/
Ark	3/4		9	**0**	3	4	4		0.012	\
	3/7		4	3	9	9	**1**		0.029	/
CkIIbeta	2/3	11,687,551..830	0	**18**	0	0	0	0.000	0.000	\

	2/9	11,687,551..94,025	6	3	2	5	4			/
Trn	11/12	6,190,657..713	14	**3**	10	6	**13**	0.001,0.018		
ham	5/6	18,776,374..471	0	**5**	0	2	0	0.006	0.000	\
	5/7	18,776,374..9,804	**4**	0	0	1	0	0.009		/

Terms and abbreviations: gene, the flybase gene symbol for the corresponding splice target, Exon-junction, reference to the numerical order of exons from left to right as they appear in the genomic sequence regardless of orientation, Physical map, the breakpoints of the alternatively spliced exons, A1 number of reads crossing the junction in control (A1) genotype, G2 number of reads *etc*. in mutant (G2) genotype, rescue, number of reads in rescue (G2-/-; TBPH-GAL4>UAS-TBPH), D42>LacZ, number of reads in D42-GAL4>UAS-LacZ controls, D42>TBPH, number of reads in D42-GAL4>UAS-TBPH overexpression, *p* all junctions, the result of a statistical test using all junction reads (not shown) from the involved gene to normalize (non-inclusive, see methods), *p* alternative exon, similar statistical test using the alternative junction reads as a normalizer (see also methods). The *p* value refers to the boldfaced, underlined junction read-count in the body of the table. If more than one *p* value is listed, reads were significant for the indicated junction in two genotypes and the p values are listed in the same order as the column order of the boldfaced and underlined genotypes. The brackets at right indicate alternative exon-junction pairs from within the same gene.
