# Supplementary material for: Comparison of Parallel High-Throughput RNA Sequencing Between Knockout of TDP-43 and Its Overexpression Reveals Primarily Nonreciprocal and Nonoverlapping Gene Expression Changes in the Central Nervous System of Drosophila
Source: G3 (Bethesda). 2012 Jul 1;2(7):789–802. doi: 10.1534/g3.112.002998 (PMC3385985; doi:10.1534/g3.112.002998)
Supplement: Supporting Information [file supp_2.7.789_TableS6.pdf]

Table S6 Differentially expressed genes with homology to human neurological disease association

Gene	Dataset	Direction	Ratio	Ortholog	e_value	rev e value	disease
Ddc	KO	Down	3.52	DDC	5.00E-175	8.00E-175	Aromatic L-amino acid decarboxylase deficiency
Tequila	OX	Down	1.86	PRSS12	5.00E-72	3.00E-72	Mental retardation
CG12728	KO	Up	1.57	NHEJ1	0.0008	0.0002	Severe combined immunodeficiency with microcephaly
CG10249	KO, OX	Up,Dn	1.56,1.3	KANK1	1.00E-67	2.00E-67	Cerebral palsy
Myo10A	OX	Down	1.54	MYO15A	0	0	Deafness
Tau	KO, OX	Up,Dn	1.45,1.21	MAPT	1.00E-24	1.00E-24	Dementia
CG3376	OX	Down	1.37	SMPD1	2.00E-120	3.00E-120	Niemann-Pick disease
CG14291	OX	Up	1.36	SGSH	3.00E-133	4.00E-133	Sanfilippo syndrome
mre11	OX	Down	1.33	MRE11A	1.00E-118	2.00E-118	Ataxia-telangiectasia-like disorder
Sh	KO	Up	1.29	KCNA1	5.00E-149	7.00E-149	Episodic ataxia/myokymia syndrome
Tsp3A	OX	Down	1.26	TSPAN7	6.00E-20	1.00E-23	Mental retardation
Spred	OX	Down	1.23	SPRED1	2.00E-19	2.00E-19	Neurofibromatosis
Htt	KO_SPLICE	Up	1.21	HTT	8.00E-20	1.00E-19	Huntington disease (3)
Mer	KO	Up	1.19	NF2	6.00E-148	7.00E-148	Meningioma
dlg1	KO	Down	1.18	DLG3	0	0	Mental retardation
CG3822	OX	Down	1.16	GRIK2	0	0	Mental retardation
Rst	OX	Down	1.16	KIRREL3	8.00E-63	4.00E-63	Mental retardation
Cac	KO_SPLICE, OX_SPLICE	Up,Dn	1.15,1.1	CACNA1S	0	0	Hypokalemic periodic paralysis
Sd	OX	Up	1.13	TEAD1	3.00E-128	1.00E-128	Sveinsson choreoretinal atrophy
Taf1	KO	Down	1.11	TAF1	0	0	Dystonia-Parkinsonism
Cap	OX	Up	1.08	SMC3	0	0	Cornelia de Lange syndrome 3
Marf	OX	Down	1.08	MFN2	0	0	Charcot-Marie-Tooth disease
CG31739	OX_SPLICE	Up	1.02	DARS2	1.00E-151	1.00E-151	Leukoencephalopathy with brainstem and spinal cord involvement and lactate elevation

Explanation of terms: In the column marked Dataset, KO refers to the differentially expressed genes in TBPH G2 mutant vs. control, OX to D42>TBPH vs. D42>LacZ control. KO_SPLICE and OX_SPLICE refer to differentially spliced genes in G2 vs. control and D42>TBPH vs. control, respectively. The direction and magnitude of change relative to control are given in the Direction and Ratio columns. The Human gene column is the proposed homolog/ortholog followed by its BLAST e-value (obtained from homophila, see text) and reverse blast e-value. The final column gives the human gene's disease associations.
