# Supplementary material for: Comparison of Parallel High-Throughput RNA Sequencing Between Knockout of TDP-43 and Its Overexpression Reveals Primarily Nonreciprocal and Nonoverlapping Gene Expression Changes in the Central Nervous System of Drosophila
Source: G3 (Bethesda). 2012 Jul 1;2(7):789–802. doi: 10.1534/g3.112.002998 (PMC3385985; doi:10.1534/g3.112.002998)
Supplement: Supporting Information [file supp_2.7.789_FigureS3.pdf]

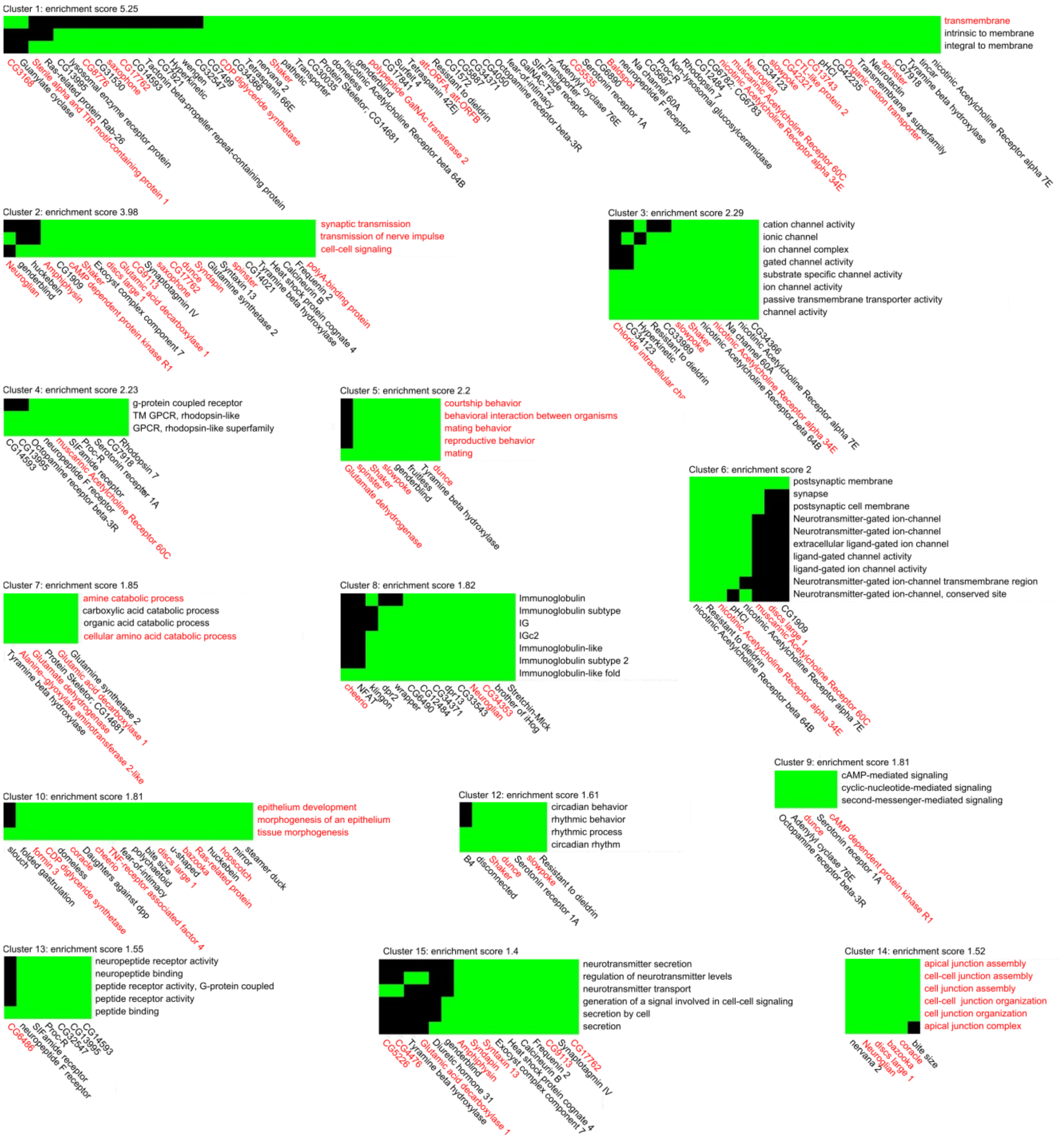

Figure S3 Clusters of annotations of DE genes from G2 TBPH homozygous mutants. The terms represented on the vertical axis co-occur in the gene sets represented on the horizontal axis, with enrichment scores reflecting the average log p-value for the group (Huang et al., 2009). Green squares indicate positive association reported between the gene and the annotation, black squares indicate no association yet reported. Red highlighted genes are orthologs of mammalian TDP targets identified by CLIP-seq (Sephton et al., 2011). Red highlighted terms were also enriched among TBPH-regulated Drosophila orthologs of vertebrate targets.
